# Enantiospecific sp^2^–sp^3^ Coupling of *ortho*‐ and *para*‐Phenols with Secondary and Tertiary Boronic Esters

**DOI:** 10.1002/anie.201710777

**Published:** 2017-11-28

**Authors:** Claire M. Wilson, Venkataraman Ganesh, Adam Noble, Varinder K. Aggarwal

**Affiliations:** ^1^ School of Chemistry University of Bristol Cantock's Close Bristol BS8 1TS UK

**Keywords:** arylation, boronic esters, C−C bond formation, phenol, synthetic methods

## Abstract

The coupling of *ortho*‐ and *para*‐phenols with secondary and tertiary boronic esters has been explored. In the case of *para*‐substituted phenols, after reaction of a dilithio phenolate species with a boronic ester, treatment with Ph_3_BiF_2_ or Martin's sulfurane gave the coupled product with complete enantiospecificity. The methodology was applied to the synthesis of the broad spectrum antibacterial natural product (−)‐4‐(1,5‐dimethylhex‐4‐enyl)‐2‐methyl phenol. For *ortho*‐substituted phenols, initial incorporation of a benzotriazole on the phenol oxygen atom was required. Subsequent *ortho*‐lithiation and borylation gave the coupled product, again with complete stereospecificity.

Phenols are ubiquitous motifs in pharmaceuticals, agrochemicals, and polymers, and are important constituents of plant metabolites possessing an array of interesting biological activities.^1^ Chiral phenolic groups, bearing benzylic stereocenters, are also found in a range of currently marketed drugs and agrochemicals (Scheme 1 a).^2^ Installation of the benzylic stereocenter would ideally be accomplished by a stereospecific cross‐coupling reaction between a halo‐phenol and a chiral organometallic reagent. However, sp^2^–sp^3^ cross‐couplings, such as the Suzuki–Miyaura reaction,^3^ of secondary and tertiary aliphatic organometallic reagents remain a significant challenge.^4^ We have worked on a conceptually different approach to such cross‐couplings and recently reported a high yielding, stereospecific method for the coupling of aryl lithium species with chiral secondary and tertiary boronic esters (Scheme 1 b).^5^, ^6^ The reaction occurs via a boronate complex that undergoes stereospecific 1,2‐migration upon activation by an electrophilic halogenating agent, with subsequent elimination of the boron and halide groups leading to the coupled product. This mode of activation proved to be highly efficient for a variety of electron‐rich heteroaromatics (e.g. furan, thiophene, indole, and benzofuran) and electron‐rich aromatics, provided they were substituted with donor groups at the *meta*‐position. The *meta* donor group and the electron‐rich boronate worked synergistically to promote an electrophilic reaction on the aromatic ring.5a,5b Positioning the donor group in the *para*‐position pushes electron density onto the sp^3^ carbon atom of the boronate complex, resulting in a selective reaction via an S_E_2 pathway, rather than an S_E_Ar pathway (e.g. Scheme 1 c, left‐hand side).5b, 7

**Scheme 1:**
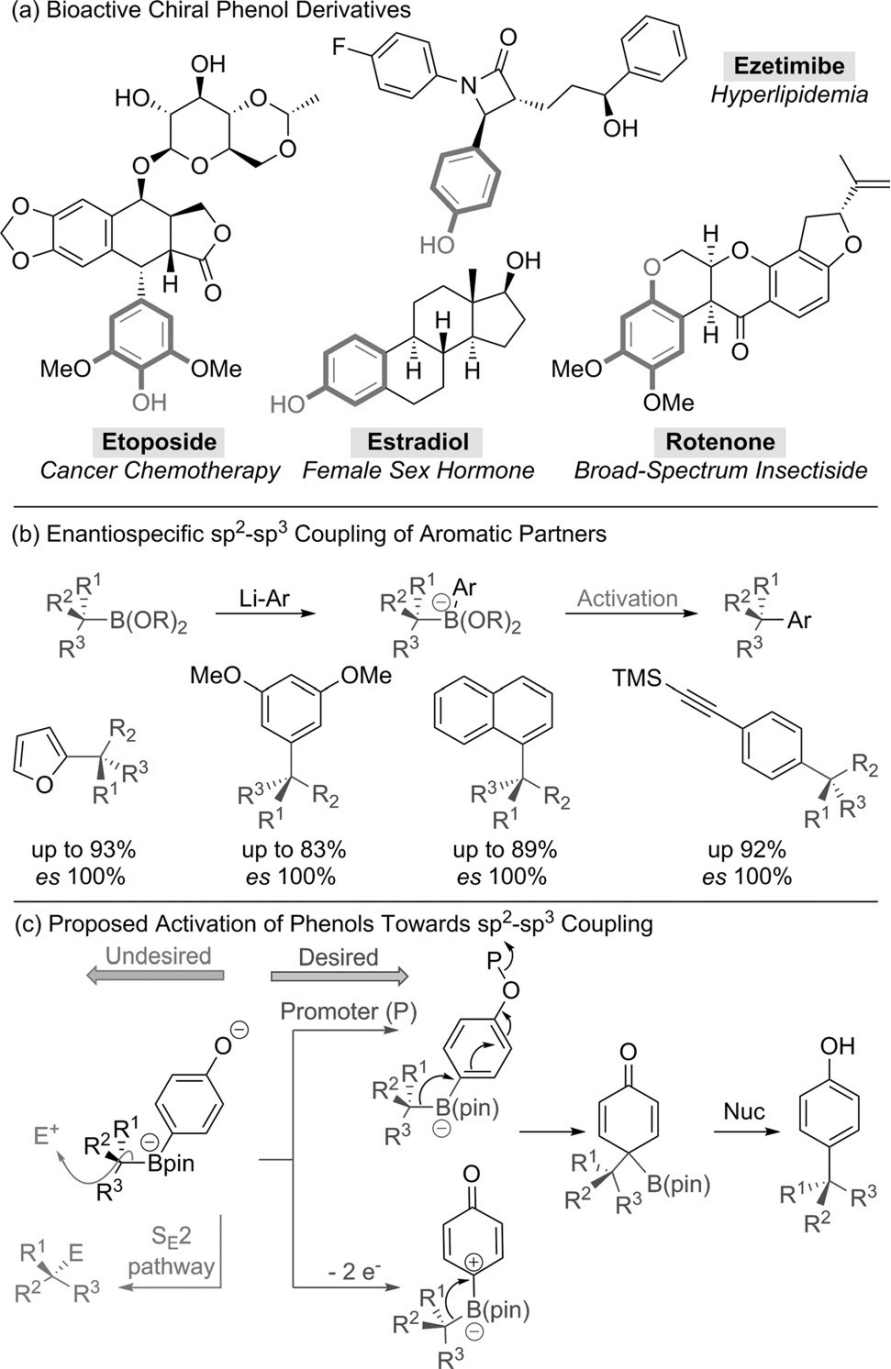
sp^2^–sp^3^ coupling of aromatic compounds and proposed coupling of phenols.

To address this limitation, we considered the counterintuitive use of the even more electron‐rich *para*‐phenolates for our coupling strategy (Scheme 1 c). Although paradoxically this should strongly promote reaction at the sp^3^ carbon (S_E_2 pathway), we reasoned that the nucleophilic properties of the phenolate oxygen would lend themselves to the identification of activating reagents that could trigger the desired 1,2‐migration of the boronate complex. Herein, we report our success in achieving this goal with two distinct methods for the stereospecific coupling of both *ortho*‐ and *para*‐substituted phenols with secondary and tertiary boronic esters.

Initially, we focused on coupling *para*‐substituted phenols. We began our studies by establishing the conditions required to promote the 1,2‐migration of the boronate complex **1 a**, which was easily prepared by reaction of boronic ester **1** with 2 equivalents of *s*BuLi. A broad range of promoters were investigated, including phenol oxidizing reagents [e.g., single‐electron oxidants: Fremy's salt, ceric ammonium nitrate, DDQ, and two‐electron oxidants: PhI(OAc)_2_)], aliphatic alcohol oxidizing reagents (e.g., Swern‐type conditions, MnO_2_), dehydrating reagents (e.g., Martin's sulfurane, Burgess reagent), phenolate alkylation reagents (e.g. Ph_3_BiF_2_), and other oxophilic reagents (e.g., Ph_3_PCl_2_, SO_2_Cl_2_). Although most were unsuccessful (see Scheme 2 and the Supporting Information for more details), we were delighted to find that Martin's sulfurane (**3**, Ph_2_S[OC(CF_3_)_2_Ph]_2_,^8^ Method A) and triphenylbismuth difluoride (**4**, Ph_3_BiF_2_,^9^ Method B) gave the desired coupling product in good yield.

**Scheme 2:**
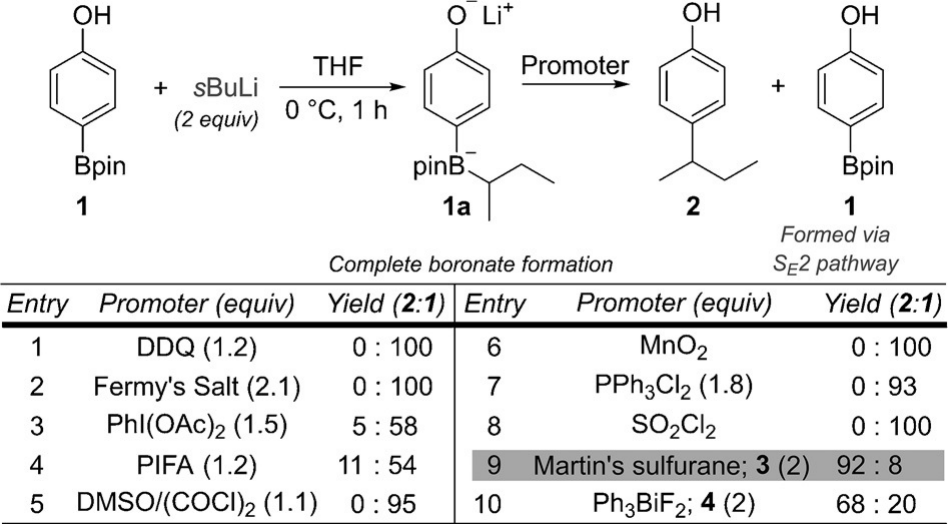
Optimization of reaction conditions. Reaction Conditions: ArBpin **1** (0.20 mmol, 1.0 equiv), *s*BuLi (0.20 mmol, 1.0 equiv), promoter (as in table above), 0 °C to RT, 16 h. Yields were determined using ^1^H NMR spectroscopy with 1,3,5‐trimethoxybenzene as an internal standard. DDQ=2,3‐dichloro‐5,6‐dicyano‐1,4‐benzoquinone; Fremy's salt=disodium nitrosodisulfonate; PIFA=phenyliodine bis(trifluoroacetate); Martin's sulfurane=bis[α,α‐bis(trifluoromethyl)benzyloxy]diphenylsulfur.

A plausible mechanism for the reaction of **1 a** with promoters **3** and **4** is presented in Scheme 3. The key step in both the cases is believed to be the functionalization of the phenolate oxygen of boronate complex **I** by the promoters **3** and **4** giving intermediates **IIa** and **IIb**, respectively. This triggers 1,2‐migration with the reduction of S^IV^ to S^II^ in **IIa** and Bi^V^ to Bi^III^ in **IIb**, leading to the quinone intermediate **III**. Subsequent rearomatization by elimination of Bpin from **III** leads to the coupled products. The success of the two reagents may be ascribed to their hindered nature, which leads to preferential reaction at the unhindered phenolate over the much more hindered boronate.

**Scheme 3:**
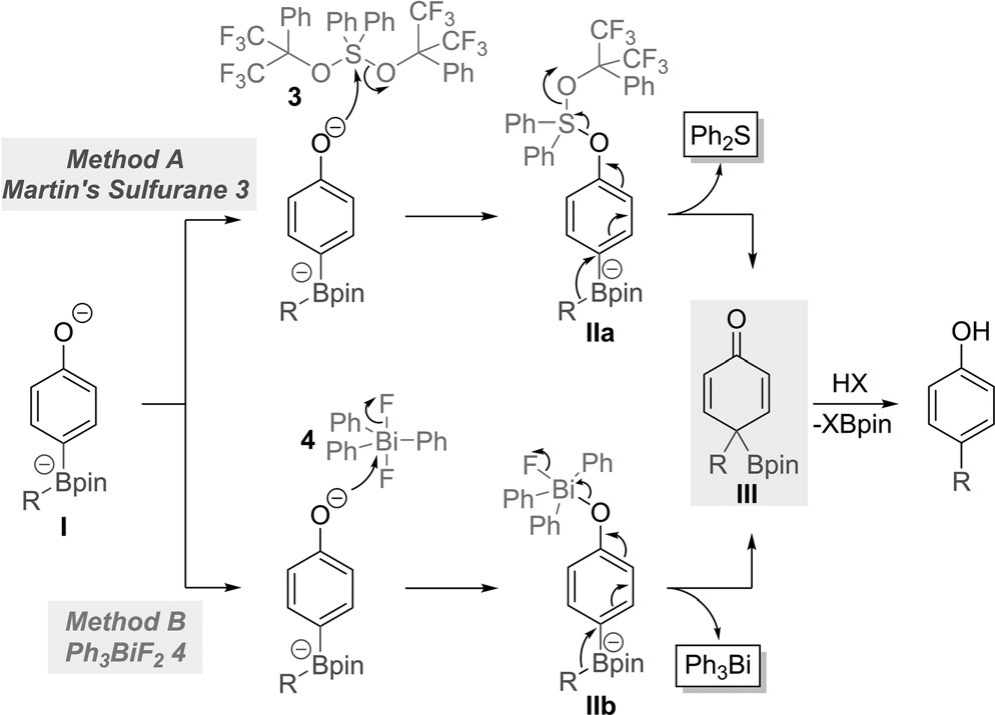
A possible mechanism for the coupling promoted by **3** and **4**.

Having established two protocols to trigger the key 1,2‐migration step, we turned our attention to linking them with in situ boronate formation from alkyl boronic esters. Dianionic boronate complex **VI** was generated by the reaction of boronic ester **6** with dilithiated aryl species **V**, which was prepared from the corresponding *para*‐bromophenols **5** through sequential deprotonation with MeLi followed by lithium–halogen exchange with *t*BuLi.^10^ The optimized coupling reaction conditions were then applied to a range of *para*‐bromophenols **5 a**–**i** (Table 1). Using benzylic boronic ester **6** as our standard, *para*‐bromophenol **5 a** gave the desired coupling product **7 a** in 62 % yield with Martin's sulfurane (**3**) as the promoter and 44 % with Ph_3_BiF_2_ (**4**). Additional methyl substituents could be introduced on the phenol at the 2‐ or 3‐positions (**7 b**, **7 c**) as well as the 2,6‐ and 3,5‐positions (**7 d**, **7 e**), albeit with a reduction in yield in the case of the sterically more demanding substrates. 1‐Naphthol could also be used in the coupling reaction (**7 f**), as could phenols bearing electron‐donating groups (OMe, **7 g**) or electron‐withdrawing groups (F, CF_3_; **7 h**, **7 i**). In most cases, Martin's sulfurane (Method A) gave higher yields than Ph_3_BiF_2_ (Method B).

**Table 1:** Substrate scope of *para*‐bromophenols for sp^2^–sp^3^ coupling with **6**.^[a]^ 
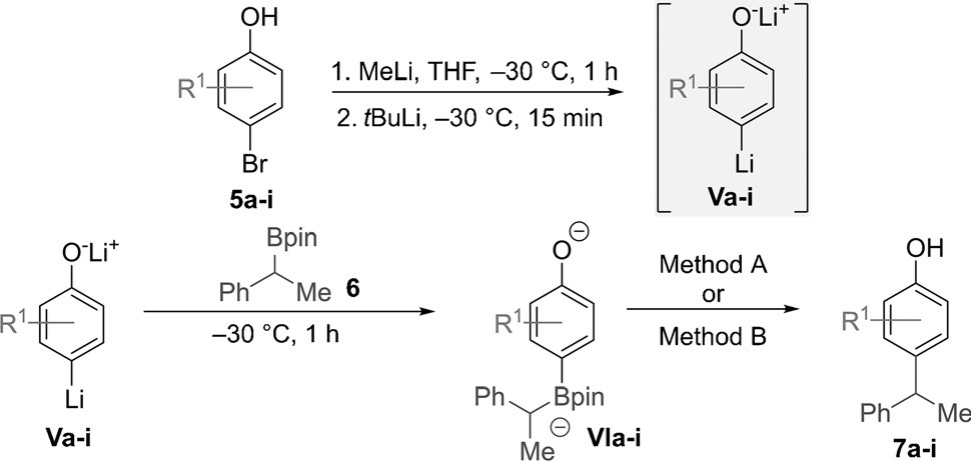

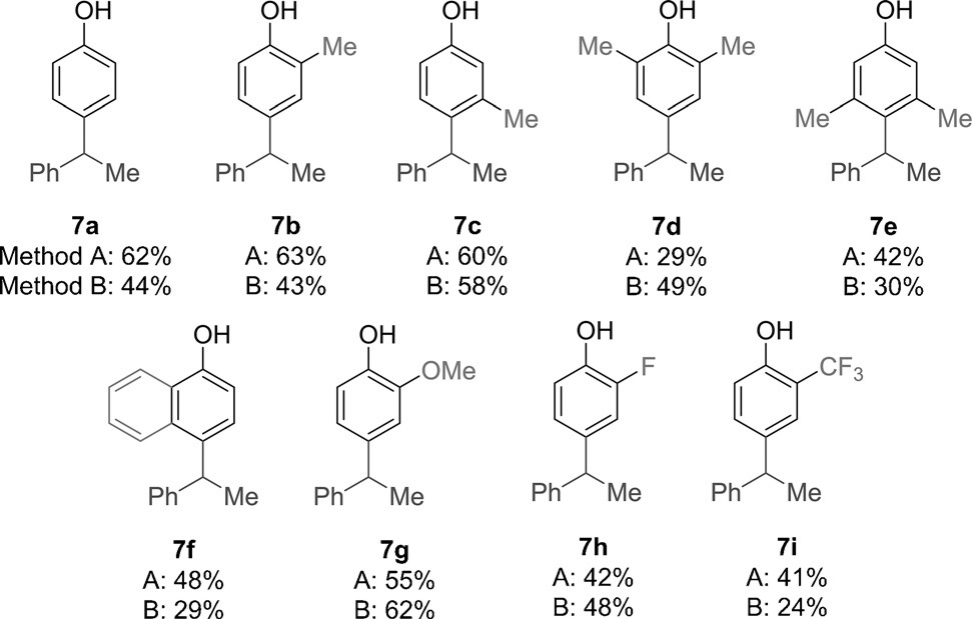

[a] Reaction conditions: Method A: ArBr (0.20 mmol, 1.25 equiv), MeLi (1.3 equiv), 1 h, *t*BuLi (2.1 equiv), 15 min, RBPin (0.16 mmol, 1.0 equiv), Martin′s sulfurane (0.20 mmol, 1.25 equiv), −30 °C, 16 h; Method B: ArBr (0.20 mmol, 1.25 equiv), MeLi (1.3 equiv), 1 h, *t*BuLi (2.1 equiv), 15 min, RBPin (0.16 mmol, 1.0 equiv), Ph_3_BiF_2_ (0.4 mmol, 2.5 equiv), −30 °C, 16 h.

Having demonstrated that a wide range of phenols could be employed in this cross‐coupling reaction, we then explored the scope of the boronic esters using Martin's sulfurane (Method A, Table 2). Primary, secondary, and tertiary boronic esters were all successfully coupled, giving high yields of the desired products with excellent enantiospecificity (*es*). Both benzylic and non‐benzylic boronic esters could be used, as well as highly hindered secondary and tertiary boronic esters. Chiral secondary boronic esters bearing alkenyl (**8 i**), cyclopropyl (**8 j**), TBS ether (**8 k**), azido (**8 l**), acetal (**8 m**), and carbamate (**8 n**) functionalities were smoothly converted to the corresponding phenol derivatives **9 i**–**n** in good yields (68–37 %) and with excellent enantiospecificity. In addition, the natural‐product‐derived boronic esters **8 o** and **8 q**, gave the desired coupling products **9 o**–**q** efficiently (58–50 %) with complete diastereospecificity.

**Table 2:** Substrate scope of chiral boronic esters for enantiospecific sp^2^–sp^3^ coupling with *para*‐bromophenols.^[a]^ 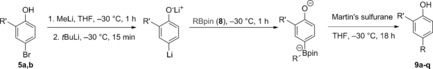

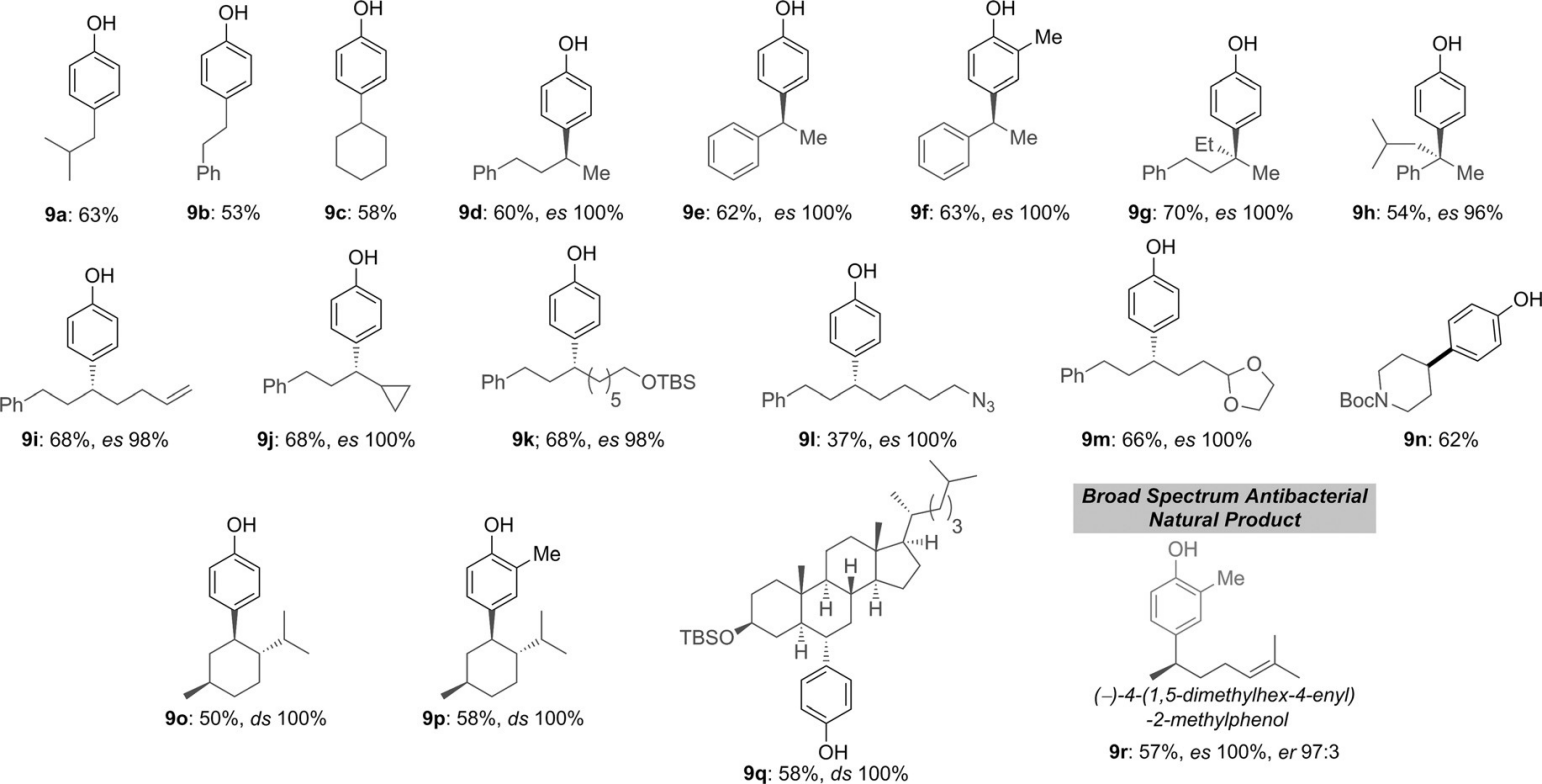

[a] Reaction conditions: ArBr (0.20 mmol, 1.25 equiv), MeLi (1.3 equiv), *t*BuLi (2.1 equiv), RBPin (0.16 mmol, 1.0 equiv), Martin′s sulfurane (0.20 mmol, 1.25 equiv), −30 °C, 16 h.

To further highlight the utility of this coupling reaction, we applied it to the synthesis of the natural product (−)‐4‐(1,5‐dimethylhex‐4‐enyl)‐2‐methylphenol (**9 r**), a compound active against a broad spectrum of gram‐positive (MIC: 16–32 μg mL^−1^) and gram‐negative bacteria including methicillin‐resistant *S. aureus* and vancomycin‐resistant *E. faecium*.^11^ Phenol **9 r** is also a key intermediate in the total synthesis of (+)‐β‐herbertenol.^12^ Treatment of enantioenriched boronic ester **8 r** 13 with dilithiated **5 b**, followed by Martin's sulfurane, gave the natural product **9 r** in 57 % yield with 100 % enantiospecificity.

Having successfully developed an enantiospecific cross‐coupling reaction for the synthesis of *para*‐substituted phenols, we wished to extend the methodology to *ortho*‐phenols. However, our attempts with *ortho*‐bromophenol **10** using the previous strategy of dilithiation/boronate complex formation and activation with Martin's sulfurane only gave a trace amount of the desired product **14 a** (Scheme 4). Although activation with Ph_3_BiF_2_ gave the coupled product in significantly higher yield (31 %), all attempts to improve upon this were unsuccessful. We believe that the increased steric demand of phenolate **11** inhibits reaction with the bulky promoters. This prompted us to investigate an alternative strategy that would avoid the requirement of a sterically hindered phenolate reacting with an activating reagent.

**Scheme 4:**
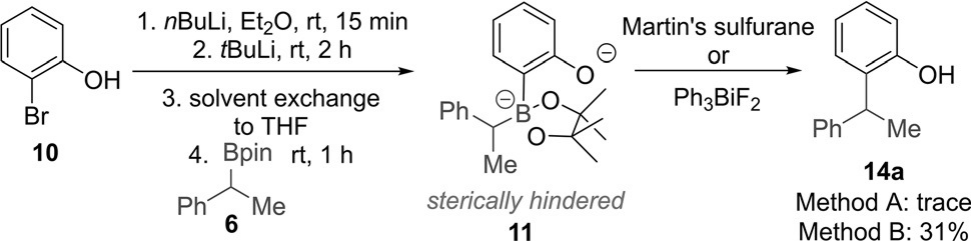
Attempts towards *ortho*‐functionalization using *ortho*‐bromophenol.

We postulated that a convenient solution to the poor reactivity of the *ortho*‐substituted phenolate would be to incorporate an efficient leaving group on the phenolic oxygen atom prior to boronate complex formation. We selected a benzotriazole (Bt) group as it can be easily introduced,^14^ is compatible with organolithium reagents,^15^ and is a good leaving group.^16^ However, we were also aware that boronate complexes possessing good leaving groups at the *ortho*‐position were prone to undergo elimination leading to benzyne formation,^17^ which is a potentially competing process.

*ortho*‐Bromophenoxybenzotriazoles **10 a**–**d** were easily prepared in one step by reaction of Ar_2_I^+^OTf^−^ with hydroxybenzotriazole (HOBt).^14^ Lithium–halogen exchange of **10 a** with *n*BuLi and treatment with boronic ester **6** gave the corresponding boronate complex (see **13**, Table 3). To our delight, upon warming to room temperature the 1,2‐migration proceeded to give the *ortho*‐functionalized phenol **14 a** in 53 % yield with 100 % *es*. Both electron‐rich (**10 b**) and electron‐deficient phenoxybenzotriazoles (**10 c**,**d**) gave the coupled products **14 b**–**d** in good yields (41–82 %). A range of chiral secondary and tertiary boronic esters were tested to explore the scope of this methodology. With simple secondary and tertiary boronic esters, 2‐bromophenoxybenzotiazole **10 a** reacted smoothly under the standard reaction conditions to give the desired products **15 a**–**d** in 22–68 % yield. The use of strongly electron‐withdrawing substituents (CF_3_) on the aromatic ring or sterically demanding tertiary pinacol boronic esters (e.g., to give **15 c**) resulted in poor yield (10–15 %) with considerable recovery of the starting boronic esters (see the Supporting Information for details). For tertiary boronic esters, exchanging the pinacol ligand for a less bulky neopentyl glycol gave the coupled product (**15 c**) in slightly improved yield (22 %). Other functionalized secondary boronic esters gave the corresponding products bearing alkenyl (**15 e**), azido (**15 f**), and carbamate (**15 h**) functionalities, as well as a menthol‐derived boronic ester (**15 g**), all with complete stereospecificity.

**Table 3:** Substrate scope of ArOBt and chiral boronic esters for enantiospecific sp^2^–sp^3^ coupling to access *ortho*‐functionalized phenols.^[a]^ 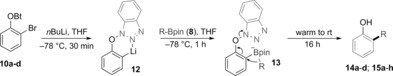

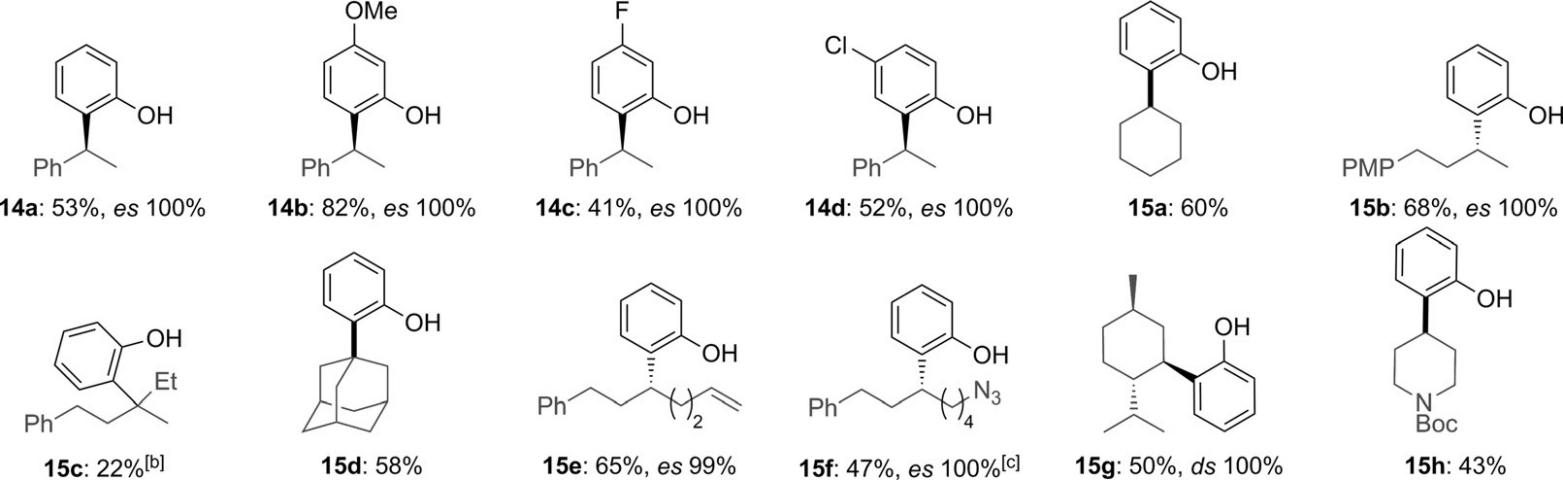

[a] Reaction Conditions: ArBr (0.30 mmol, 1.5 equiv), *n*BuLi (1.5 equiv), RBpin (0.20 mmol, 1.0 equiv), −78 °C to RT, 16 h; [b] Bneop ester was used; [c] ArBr (0.20 mmol, 1.1 equiv), *n*BuLi (1.1 equiv), RBpin (0.18 mmol, 1.0 equiv), −95 °C to RT, 16 h.

In conclusion, we have developed a general method for the enantiospecific coupling of boronic esters with *ortho*‐ and *para*‐substituted phenols. For hindered *ortho*‐substituted phenols, a pre‐incorporated leaving group (benzotriazole) was required to promote 1,2‐migration of the corresponding boronate complex. For the less hindered *para*‐substituted phenols 1,2‐migration was triggered by using Martin's sulfurane or Ph_3_BiF_2_. The substrate scope was found to be broad, with excellent functional‐group tolerance demonstrated over a range of boronic ester and phenol coupling partners. This methodology provides an important extension to the growing range of aromatic compounds that can partake in stereospecific transition‐metal‐free cross‐coupling reactions^5^ of highly hindered secondary and tertiary boronic esters and will likely find broad utility for the synthesis of challenging chiral phenol derivatives.

## Conflict of interest

The authors declare no conflict of interest.

## Supporting information

As a service to our authors and readers, this journal provides supporting information supplied by the authors. Such materials are peer reviewed and may be re‐organized for online delivery, but are not copy‐edited or typeset. Technical support issues arising from supporting information (other than missing files) should be addressed to the authors.

Supplementary
