# Supplementary material for: Enantiospecific sp2–sp3 Coupling of ortho‐ and para‐Phenols with Secondary and Tertiary Boronic Esters
Source: Angew Chem Int Ed Engl. 2017 Nov 28;56(51):16318–22. doi: 10.1002/anie.201710777 (PMC5767764; doi:10.1002/anie.201710777)
Supplement: Supplementary file 1 — Supplementary [file ANIE-56-16318-s001.pdf]

Angewandte Chemie
Eine Zeitschrift der Gesellschaft Deutscher Chemiker

Supporting Information

Enantiospecific sp^2–sp^3 Coupling of *ortho*- and *para*-Phenols with Secondary and Tertiary Boronic Esters

*Claire M. Wilson[+], Venkataraman Ganesh[+], Adam Noble, and Varinder K. Aggarwal**

anie_201710777_sm_miscellaneous_information.pdf

Table of Contents

**1.**	**General Information**	**S3**
**2.**	**Synthesis of Starting Materials**	**S5**
**3.**	**Electrophile Screening and Additional Information on Reaction Development**	**S11**
**4.**	**General Procedures**	**S16**
**5.**	**Compound Characterization Data**	**S19**
**6.**	**Reversible Boronate Complex Formation with Benzotriazoles**	**S36**
**7.**	**NMR Spectra**	**S37**
**8.**	**References**	**S75**

1. General Information

Solvents and Reagents: All air and water-sensitive reactions were carried out in flame-dried glassware under a nitrogen atmosphere using standard Schlenk manifold technique. Bulk solutions were evaporated under reduced pressure using a Büchi rotary evaporator. All solvents were commercially supplied or provided by the communal stills of the School of Chemistry, University of Bristol. Petroleum ether (pet. ether) refers to the fraction collected between 40 – 60 °C. TMEDA was distilled over CaH_2. (+)-Sparteine and (–)-sparteine were obtained from the commercially available sulfate pentahydrate salt (99%, Acros) and distilled before use. The sparteine free base readily absorbs atmospheric carbon dioxide (CO_2) and should be stored under argon/nitrogen at −20 °C in a Schlenk tube. sec-BuLi was purchased from Acros. PhLi was purchased from Sigma-Aldrich. The molarity of organolithium solutions was determined by titration using N-benzyl benzamide as an indicator. All other reagents were purchased from commercial sources and used as sold, unless noted.

Chromatography and Spectroscopy: Flash column chromatography (FCC) was carried out using fluorochem silica gel LC60A-40 (63 μm). Auto-column chromatography was carried out on a Biotage, Isolera One using Biotage SNAP cartridge KP Sil 5 g, unless otherwise stated. All reactions were followed by thin-layer chromatography (TLC) when practical, using Merck Kieselgel 60 F_{254} fluorescent treated silica which was visualised under UV light or by staining with aqueous basic potassium permanganate or phosphomolybdic acid.

^1H and ^{13}C NMR spectra were recorded using Jeol ECP(Eclipse) 300 MHz, Jeol ECS 400 MHz, Varian VNMR 400 MHz and Varian VNMR 500 MHz spectrometers. Chemical shifts (δ) are given in parts per million (ppm), and coupling constants (J) are given in Hertz (Hz). The ^1H NMR spectra are reported as follows: ppm (multiplicity, coupling constants, number of protons, assignment). Data are reported as follows: chemical shift, multiplicity (s = singlet, br s = broad singlet, d = doublet, t = triplet, q = quartet, qi = quintet, sx = sextet, sp = septet, m = multiplet, dd = doublet of doublets, etc.) and integration. NMR assignments are made according to spin systems, using two-dimensional (COSY, HSQC, HMBC) NMR spectroscopy to assist the assignment. Where an assignment could not be made unambiguously, possible assignments are listed.

High resolution mass spectra (HRMS) were recorded on a VG Analytical Autospec by Electron Ionisation (EI) or Chemical Ionisation (CI) or on a Brüker Daltonics Apex IV by Electrospray Ionisation (ESI). IR spectra were recorded on a Perkin Elmer Spectrum One FT-IR as a thin film. Only selected absorption maxima (ν_{max}) are reported in wavenumbers (cm^{-1}). Melting points were recorded in degrees Celsius (°C), using a Kofler hot-stage microscope apparatus and are reported uncorrected. Optical rotation ($[\alpha]_D^T$) was measured on a

Bellingham and Stanley Ltd. ADP220 polarimeter and is quoted in (° ml)(g dm)$^{-1}$. Chiral HPLC was performed on a HP Agilent 1100 with a Chiralpak columns and monitored by DAD (Diode Array Detector). Chiral SFC was performed on a Waters TharSFC system using a Diacel Chiralpak columns (4.6 m × 250 mm × 5 µm) and monitored by DAD (Diode Array Detector). GC-MS was performed on an Agilent 7820A using a HP-5MS UI column (30 m x 0.25 mm x 0.25 µm).

Naming of compounds: Compound names are those generated by ChemBioDraw 15.0 software (PerkinElmer), following the IUPAC nomenclature.

Solution of Martin's sulfurane: Due its hydroscopic nature, a solution of Martin's sulfurane was employed in this chemistry. This solution was made by emptying the contents of a full (5 g) bottle of Martin's sulfurane reagent into a pre-weighed dry schlenk tube. Dry THF was added to the schlenk tube to make a 0.5 M solution, based on the weight of Martin's sulfurane reagent in the schlenk tube. This solution was found to be active for months when stored at room temperature under N_2 atmosphere.

2. Synthesis of Starting Materials

Synthesis of Boronic Ester Synthesis

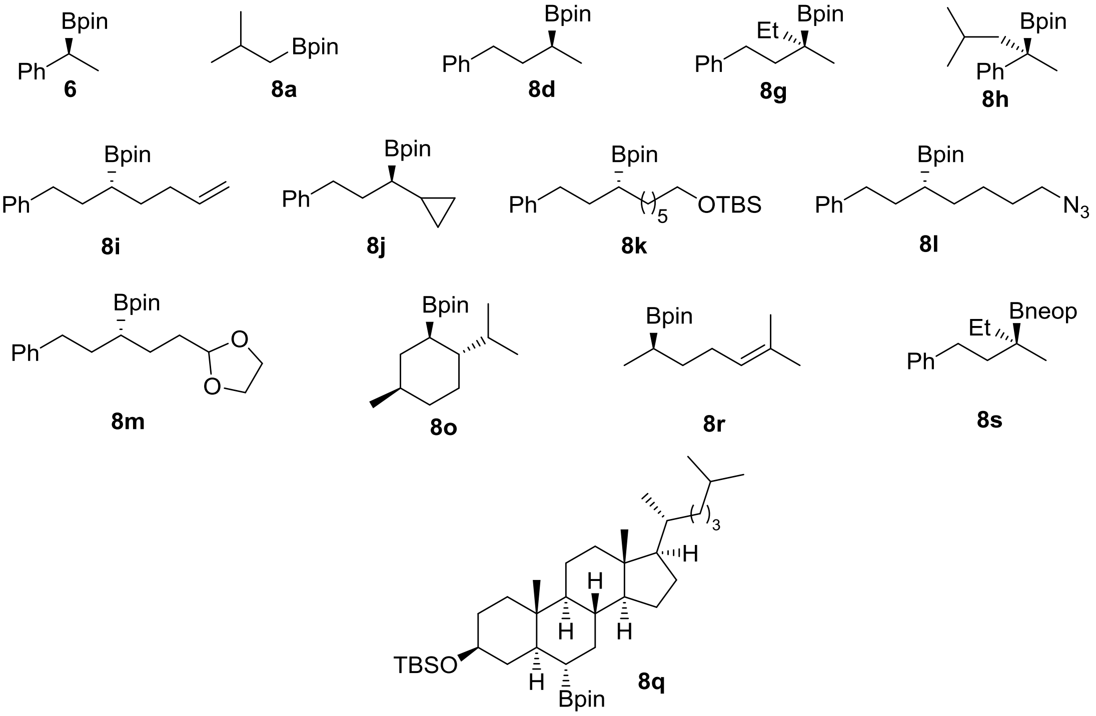

Please see the references below for the synthesis and determination of the enantiomeric excesses (where applicable) of boronic esters **6** and **8a-8j**. Substrates available from commercial sources (**8b-8c** and **8n**) are not listed here.

6: (*S*)-4,4,5,5-tetramethyl-2-(1-phenylethyl)-1,3,2-dioxaborolane[2]

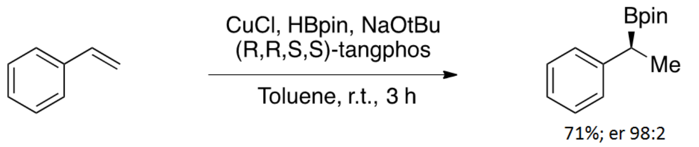

8a: 2-isobutyl-4,4,5,5-tetramethyl-1,3,2-dioxaborolane[4]

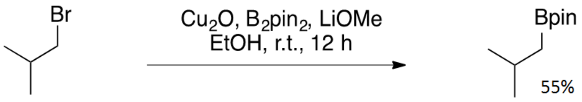

8d: (*S*)-4,4,5,5-tetramethyl-2-(4-phenylbutan-2-yl)-1,3,2-dioxaborolane[3]

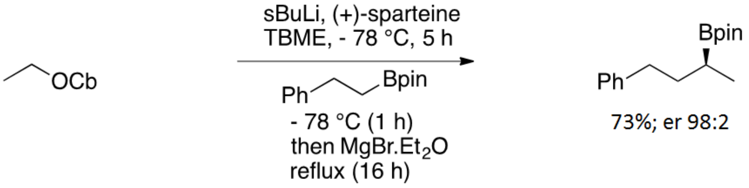

8g: (*S*)-4,4,5,5-tetramethyl-2-(3-methyl-1-phenylpentan-3-yl)-1,3,2-dioxaborolane[7]

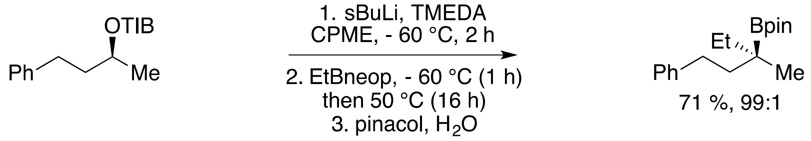

8h: (*S*)-4,4,5,5-tetramethyl-2-(4-methyl-2-phenylpentan-2-yl)-1,3,2-dioxaborolane[5]

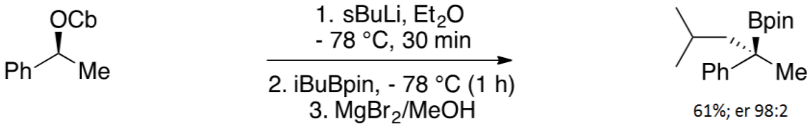

8i: (*R*)-4,4,5,5-tetramethyl-2-(1-phenylhept-6-en-3-yl)-1,3,2-dioxaborolane[1]

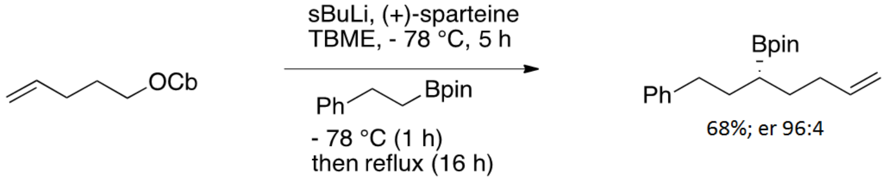

8j: (*S*)-2-(1-cyclopropyl-3-phenylpropyl)-4,4,5,5-tetramethyl-1,3,2-dioxaborolane[1]

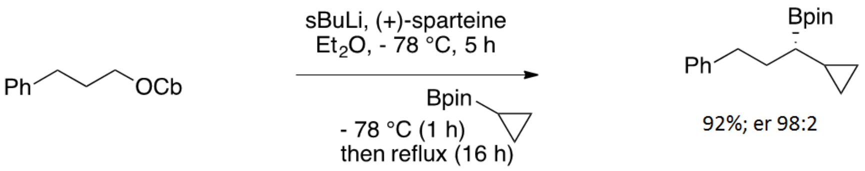

8k: (*S*)-*tert*-butyldimethyl((9-phenyl-7-(4,4,5,5-tetramethyl-1,3,2-dioxaborolan-2-yl)nonyl)oxy)silane[1b]

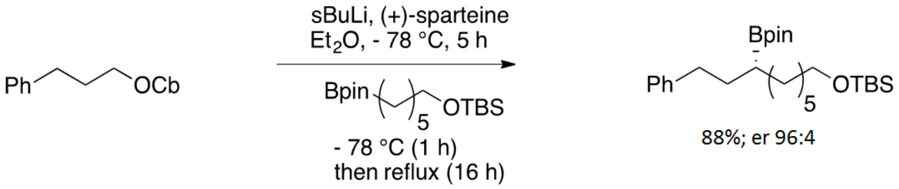

8l: (*R*)-4,4,5,5-tetramethyl-(7-azido-1-phenylheptyl)-1,3,2-dioxaborolane

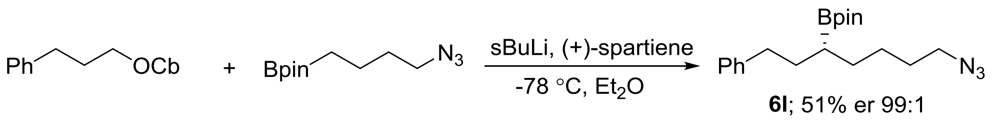

8m: (*S*)-4,4,5,5-tetramethyl-(1-(1,3-dioxolan-2-yl)-5-phenylpentyl)-1,3,2-dioxaborolane

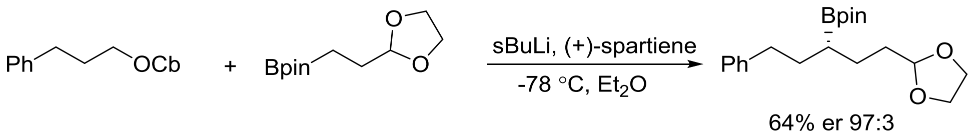

8o: 2-((1*R*,2*R*,5*R*)-2-isopropyl-5-methylcyclohexyl)-4,4,5,5-tetramethyl-1,3,2-dioxaborolane[6]

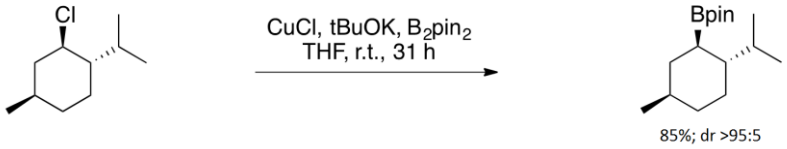

8q: 4-(4,4,5,5-tetramethyl-(5*R*,6*S*)-3-O-(tert-Butyldimethylsilyl)cholestr-6-yl)- 1,3,2-dioxaborolane

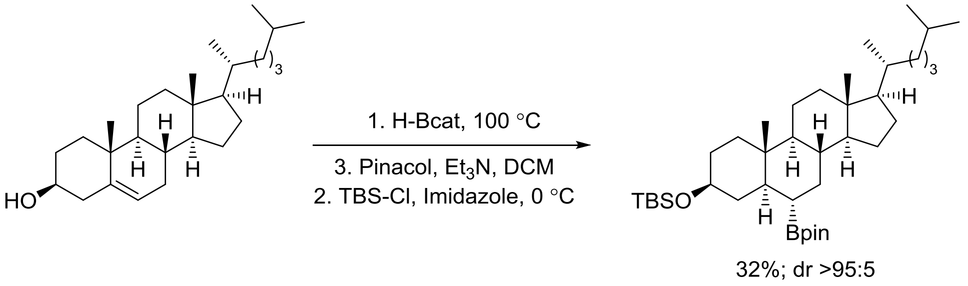

8r: (*R*)-4,4,5,5-tetramethyl-2-(6-methylhept-5-en-2-yl)-1,3,2-dioxaborolane[8]

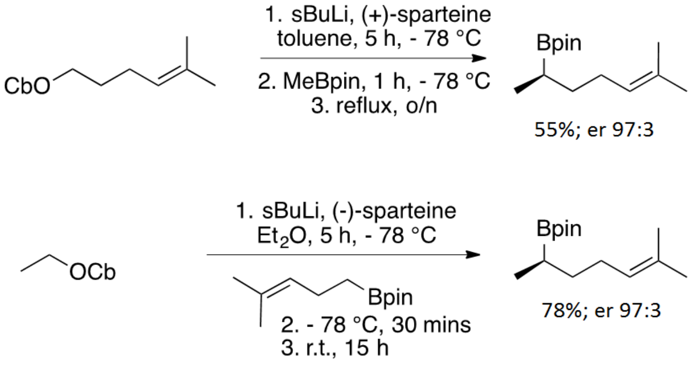

8s: (*S*)-5,5-dimethyl-2-(3-methyl-1-phenylpentan-3-yl)-1,3,2-dioxaborinane[7]

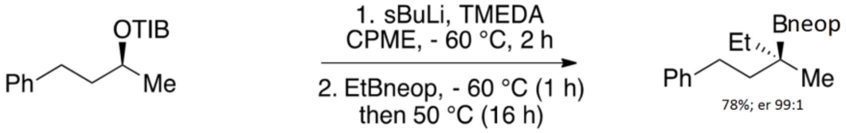

Procedure for the Synthesis of Iodonium Triflates

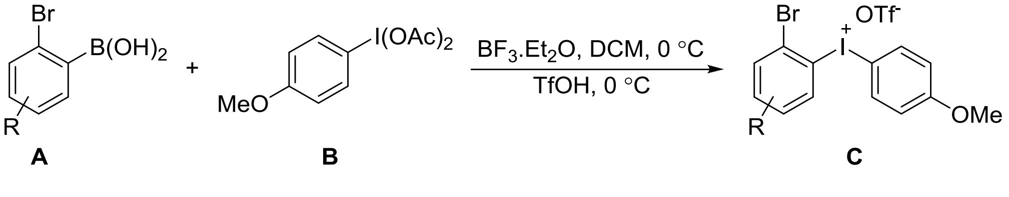

To a stirred suspension of boronic acid (**A**, 25 mmol; 1.0 equiv.) in DCM (50 mL) under N_2 at 0 °C was added $BF_3.Et_2O$ (3.5 mL; 28 mmol; 1.1 equiv.) and the reaction was stirred for 1 h at 0 °C. Then, a solution of anisyl iodoacetate (**B**, 9.24 g; 26.3 mmol; 1.05 equiv.) in DCM (15 mL) was added dropwise to the reaction mixture at 0 °C and stirred at rt for a further 1 h. The mixture was cooled again to 0 °C and TfOH (2.5 mL; 28 mmol; 1.1 equiv.) was added dropwise. After stirring for 10 min, the reaction mixture was concentrated in vacuo and passed through a pad of silica using 5% MeOH/DCM solution (150 mL) as eluent. The filtrate was evaporated to dryness to give the desired iodonium triflate (**C**).

(2-Bromo-5-methoxyphenyl)(4-methoxyphenyl)iodonium trifluoromethanesulfonate (S1)

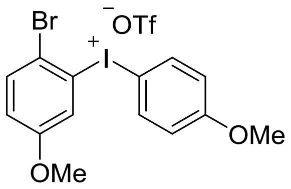

The starting boronic acid (1.00 g; 4.35 mmol) was reacted according to the above general procedure to afford the title compound (1.47 g; 61%) as white solid. ^{1}H NMR (500 MHz, acetonitrile-d_3) 8.15 – 8.04 (m, 2H), 7.78 (d, *J* = 8.9 Hz, 1H), 7.72 (d, *J* = 2.9 Hz, 1H), 7.15 (dd, *J* = 8.9, 2.9 Hz, 1H), 7.13 – 7.04 (m, 2H), 3.87 (s, 3H), 3.86 (s, 3H); ^{13}C NMR (126 MHz, acetonitrile-d_3) 163.4, 160.3, 137.9, 134.2, 123.5, 122.2, 121.1 (q, *J* = 320 Hz), 119.7, 119.3, 118.1, 116.5, 102.0, 56.2, 55.7; ^{19}F NMR (283 MHz, acetonitrile-d_3) -79.17; HRMS (ESI$^+$) mass calculated for $[M]^+$ $C_{14}H_{13}BrIO_2^+$ requires m/z 418.9138, found m/z 418.9141.

(2-Bromo-5-fluorophenyl)(4-methoxyphenyl)iodonium trifluoromethanesulfonate (S2)

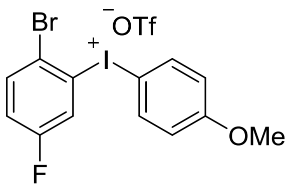

The starting boronic acid (1.00 g; 4.57 mmol) was reacted according to the above general procedure to afford the title compound (2.2 g; 86%) as white solid. ^{1}H NMR (500 MHz, acetonitrile-d_3) 8.16 – 8.05 (m, 2H), 8.00 – 7.89 (m, 2H), 7.40 (ddd, *J* = 8.8, 8.0, 2.9 Hz, 1H), 7.16 – 7.07 (m, 2H), 3.89 (s, 3H); ^{13}C NMR (126 MHz, acetonitrile-d_3) δ 163.6, 161.6 (d, *J* = 254.4 Hz), 138.1, 135.1 (d, *J* = 8.0 Hz), 125.2 (d, *J* = 26.7 Hz), 122.1 (d, *J* = 22.5 Hz), 121.7 (d, *J* = 3.9 Hz), 120.9 (q, *J* = 320 Hz), 119.2 (d, *J* = 8.2 Hz), 118.2, 102.2, 55.8; ^{19}F NMR (377 MHz, acetonitrile-d_3) δ -73.97, -105.02 (td, *J* = 7.7, 5.1 Hz). HRMS (ESI$^+$) mass calculated for $[M]^+$ $C_{13}H_{10}BrFIO^+$ requires m/z 406.8938, found m/z 406.8949.

(2-Bromo-4-chlorophenyl)(4-methoxyphenyl)iodonium trifluoromethanesulfonate (S3)

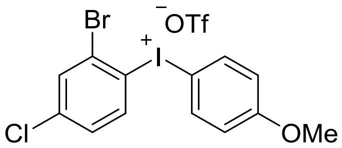

The starting boronic acid (1.00 g; 4.27 mmol) was reacted according to the above general procedure to afford the title compound (2.2 g; 86%) as white solid. ^{1}H NMR (500 MHz, $CDCl_3$) 8.06 (dd, *J* = 15.2, 8.9 Hz, 3H), 7.95 (d, *J* = 2.3 Hz, 1H), 7.50 (dd, *J* = 8.7, 2.3 Hz, 1H), 7.07 (d, *J* = 9.1 Hz, 2H), 3.85 (s, 3H); ^{13}C NMR (126 MHz, $CDCl_3$) 168.8, 145.4, 144.3, 143.3, 139.1, 136.4, 133.1, 126.3 (q, *J* = 320 Hz), 123.5, 122.8, 107.4, 61.1; ^{19}F NMR (283 MHz, acetonitrile-d_3) -79.21; HRMS (ESI$^+$) mass calculated for $[M]^+$ $C_{13}H_{10}BrClIO^+$ requires m/z 422.8643, found m/z 422.8647.

Procedure for the Synthesis Arylbenzotriazoles

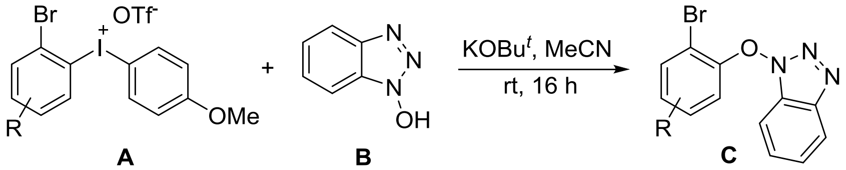

To a stirred suspension of hydroxybenzotriazole (HOBt, **B**, 243 mg; 1.80 mmol; 1.00 equiv.) in MeCN (2.0 mL) under N_2 at rt was added solid KOBu^t (202 mg; 1.80 mmol; 1.00 equiv.) and stirred for 10 min at rt. Then, solid iodonium triflate (**A**, 1.05 equiv.) was added to the reaction mixture in one portion and stirred. After 16 h, the reaction was quenched by the addition of water (20 mL) and diluted with DCM (20 mL). The layers were separated and the aqueous layer was extracted with DCM (3 x 20 mL). The combined organic layers were washed with brine (30 mL), dried over $MgSO_4$, filtered and concentrated under vacuum. The crude material was purified by flash column chromatography on silica gel to afford **C**.

Compound **10a** was prepared following the literature report.[9]

1-(2-Bromo-5-methoxyphenoxy)-1H-benzo[*d*][1,2,3]triazole 10b

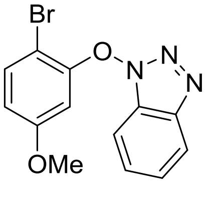

The starting iodonium triflate (1.7 g; 3.0 mmol) was reacted according to the above general procedure to afford the title compound (622 mg; 65%) as white solid.

R_f (10% EtOAc/pet. ether): 0.5; IR (film) ν_{max}/cm^{-1}: 3304, 1592, 1266, 1037, 963, 765, 740; ^{1}H NMR (500 MHz, $CDCl_3$) 8.08 (dd, *J* = 8.4, 0.8 Hz, 1H), 7.65 – 7.58 (m, 1H), 7.58 – 7.49 (m, 2H), 7.44 (ddt, *J* = 8.5, 6.9, 0.8 Hz, 1H), 6.64 (ddd, *J* = 8.8, 2.7, 0.5 Hz, 1H), 6.35 – 6.26 (m, 1H), 3.63 (s, 3H); ^{13}C NMR (126 MHz, $CDCl_3$) 160.2, 155.9, 143.3, 134.1, 128.9, 127.5, 125.1, 120.4, 112.1, 108.9, 102.5, 99.9, 55.7; HRMS (ESI$^+$) mass calculated for [M+Na]$^+$ $C_{13}H_{10}BrN_3NaO_2$ requires m/z 341.9849, found m/z 341.9851.

1-(2-Bromo-5-fluorophenoxy)-1H-benzo[*d*][1,2,3]triazole 10c

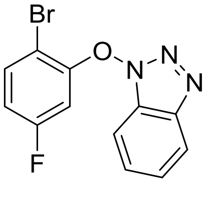

The starting iodonium triflate (2.1 g; 3.8 mmol) was reacted according to above general procedure to afford the title compound (696 mg; 59%) as white solid.

Mpt: 92-95 °C; R_f (5% EtOAc/pet. ether): 0.4; IR (film) ν_{max}/cm^{-1}: 3093, 1412, 1321, 1127, 1076, 740; ^{1}H NMR (500 MHz, $CDCl_3$) 8.05 (dd, *J* = 8.4, 0.9 Hz, 1H), 7.59 (dd, *J* = 8.9, 5.6 Hz, 1H), 7.56 – 7.49 (m, 2H), 7.41 (ddd, *J* = 8.4, 6.3, 1.7 Hz, 1H), 6.79 (ddd, *J* = 8.8, 7.6, 2.7 Hz, 1H), 6.41 (dd, *J* = 9.0, 2.7 Hz, 1H); ^{13}C NMR (126 MHz, $CDCl_3$) 162.1 (d, *J* = 250.4 Hz), 155.9 (d, *J* = 9.8 Hz), 143.2, 134.7 (d, *J* = 8.9 Hz), 129.2, 127.4, 125.3, 120.5, 113.8 (d, *J* = 22.4 Hz), 108.6, 103.9 (d, *J* = 28.1 Hz),

103.8 (d, J = 4.0 Hz); ^{19}F NMR (377 MHz, acetonitrile-d_3) δ -105.02 (td, J = 7.7, 5.1 Hz). HRMS (ESI$^+$) mass calculated for $[M+Na]^+$ $C_{12}H_7BrFN_3NaO$ requires m/z 329.9649, found m/z 329.9649.

1-(2-Bromo-4-chlorophenoxy)-1H-benzo[*d*][1,2,3]triazole 10d

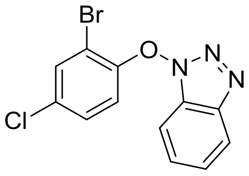

The starting iodonium triflate (1.85 g; 3.23 mmol) was reacted according to the above general procedure to afford the title compound (730 mg; 70%) as white solid.

R_f (5% EtOAc/pet. ether): 0.4; IR (film) ν_{max}/cm^{-1} 3007, 1477, 1037, 740; ^{1}H NMR (400 MHz, $CDCl_3$) 8.02 (dt, J = 8.4, 0.9 Hz, 1H), 7.61 (d, J = 2.4 Hz, 1H), 7.57 – 7.45 (m, 2H), 7.38 (ddd, J = 8.1, 6.6, 1.4 Hz, 1H), 7.11 (dd, J = 8.9, 2.4 Hz, 1H), 6.63 (d, J = 8.9 Hz, 1H); ^{13}C NMR (126 MHz, $CDCl_3$) 154.3, 143.3, 133.5, 131.5, 129.0, 128.9, 127.4, 125.3, 120.5, 116.6, 110.4, 108.7; HRMS (ESI$^+$) mass calculated for $[M+Na]^+$ $C_{12}H_7BrClN_3NaO$ requires m/z 345.9353, found m/z 345.9357.

3. Electrophile Screening and Additional Information on Reaction Development

Table 1: Screening of promotors using the model reaction.

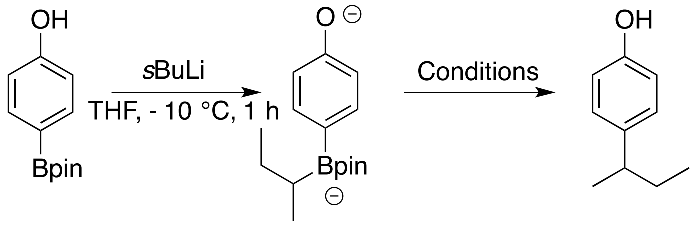

Reagent (equiv.)	Reaction Conditions	Result[a]
**Alcohol Oxidation**		
DMSO (1.5), $(COCl)_2$ (1.1)	THF, - 78 °C to r.t.,20 h	No product detected
DMSO (1.5), $(COCl)_2$ (1.1)	CH_2Cl_2, - 78 °C to r.t., 20 h	No product detected
NCS (5), Me_2S (4.6)	toluene, 0 °C to r.t., 17 h	No product detected
SO_3.pyr (3), DMSO (xs.)	CH_2Cl_2, 0 °C to r.t., 20 h	No product detected
CrO_3 (3.0), H_2SO_4	H_2O, 0 °C to r.t., 0 °C to r.t.	No product detected
SO_3.pyr (3) in xs DMSO	DMSO, 0 °C to r.t., 20 h	No product detected
NCS (1), Me_2S (1.4)	toluene, 0 °C to r.t., 0 °C to r.t.	No product detected
**Dehydrating Agent**		
Martin's sulfurane (2.5)	CH_2Cl_2, 0 °C to r.t., 1 h	Product 69% NMR yield P:SM = 84:16
Burgess reagent (1.5)	THF, 0 °C to r.t., 20 h	No product detected
$(PNCl_2)_3$ (1.5)	THF, 0 °C to r.t., 20 h	No product detected
**Phenol Oxidation**		
ceric ammonium nitrate (2.0)	H_2O, 0 °C to r.t., 20 h	No product detected
2,3-dichloro-5,6-dicyano-1,4-benzoquinone (DDQ, 1.2)	THF, 0 °C to r.t., 20 h	No product detected
Fremy's Salt (2.1)	H_2O, 0 °C to r.t., 0 °C to r.t.	No product detected
**Iodine Reagents**		
I_2 (5), 20 h	THF, 0 °C to r.t., 20 h	No product detected
phenyliodine bis(trifluoroacetate) (1.2)	THF, 0 °C to r.t., 20 h	Product = 11% NMR yield P:SM = 17:83
phenyliodine diacetate (1.5)	THF, 0 °C to r.t., 20 h	Product = 5% NMR yield P:SM = 8:92
phenyliodine diacetate (1.2)	THF, 0 °C to r.t., 20 h	Product = 4% NMR yield P:SM = 7:93

I_2 (1.2), 20 h	THF, 0 °C to r.t., 20 h	Product = 8% NMR yield<br>P:SM = 10:90
**Miscellaneous**		
Sulfuryl chloride (2.6)	THF, 0 °C to r.t., 20 h	No product detected
Thionyl Chloride (2.8)	THF, 0 °C to r.t., 20 h	No product detected
$Pd(OAc)_2$ (1.0)	THF, 0 °C to r.t., 20 h	No product detected
PPh_3Cl_2	THF, 0 °C to r.t., 20 h	No product detected
MnO_2 (1.5)	THF, 0 °C to r.t., 20 h	No product detected
MnO_2 (26), 20 h	THF, 0 °C to r.t., 20 h	No product detected
PPh_3Cl_2 (1.8)	CH_2Cl_2, 0 °C to r.t., 20 h	No product detected
ox-TEMPO-BF_4 (1)	THF, 0 °C to r.t., 20 h	No product detected
Ph_3BiF_2 (1.7)	THF, 0 °C to r.t., 20 h	Product 68% NMR yield<br>P:SM = 78:23
Ph_3BiCl_2 (1.7)	THF, 0 °C to r.t., 20 h	No product detected

[a] NMR yield determined using 1,3,5-trimethoxybenzene as an internal standard. Product:SM (P:SM) ratio determined by GCMS.

Table 2: Martin's sulfurane optimization: Effect of solvent in model reaction .

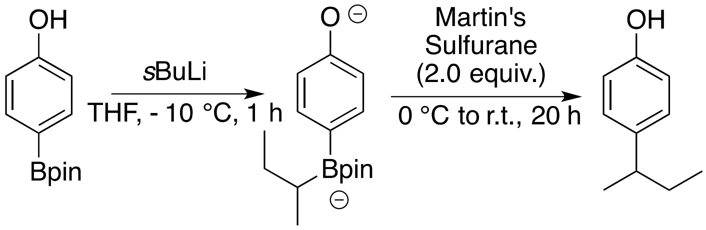

Solvent	**NMR yield (%)**	**GCMS (P:SM)**	*Solvent*	**NMR yield (%)**	**GCMS (P:SM)**
THF	69	74:26	*Toluene*	22	32:68
1,4-dioxane (with THF)	51	71:29	*DMF*	54	51:49
Et_2O	29	39:61	*CH_3CN*	42	39:61
Et_2O (with THF)	29	39:61	*DCE*	29	45:54
MTBE	21	23:77	*$CHCl_3$*	25	47:53
MTBE (with THF)	48	61:39	*DCM*	38	69:31

Table 3: Martin’s sulfurane optimization: Effect of stoichiometry in model reaction.

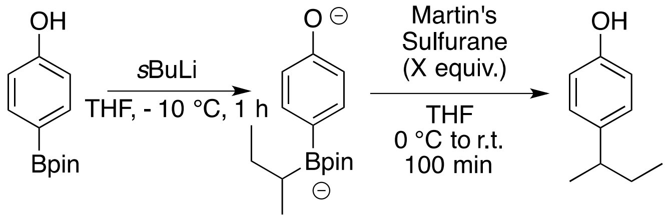

Equivalents (X)	NMR yield (%)	GCMS (P:SM)
1.0	31	61:39
1.25	46	69:31
1.5	73	85:15
1.75	96	97:3
2.0	92	92:8

Table 4: Martin’s sulfurane optimisation. Effect of temperature in model reaction.

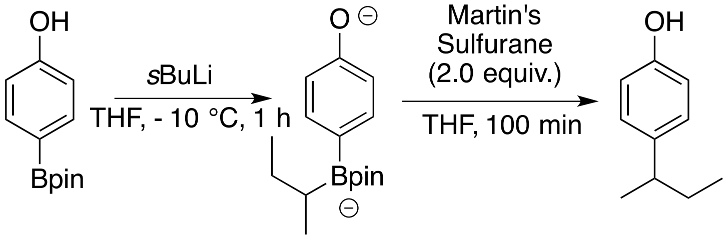

Temperature	NMR yield (%)	GCMS (P:SM)
r.t	61	79:21
0 °C	62	90:10
0 °C*	37	67:33
50 °C	41	64:36
-30 °C^	89	97:3
-30 °C to 0 °C^	90	97:3
-78 °C^	50	94:6

* MS added in two portions

^ Martin’s sulfurane (1.75 equiv.), 30 mins

Table 5: Martin's sulfurane optimisation. Effect of stoichiometry in the reaction with CyBpin.

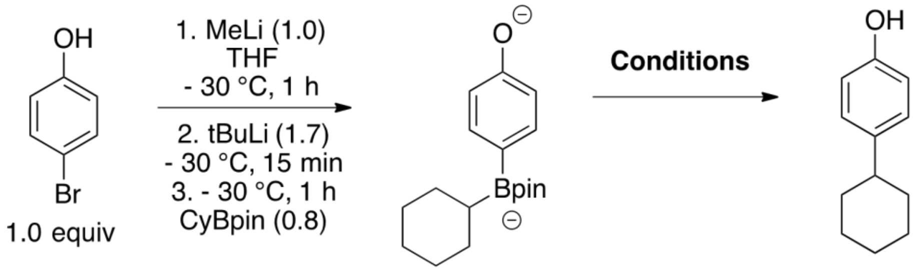

Equivalents Martin's sulfurane[a]	NMR yield (%)
2.25	6
1.75	13
1.5	20
1.25	39
1.0	53

[a] Martin's sulfurane, THF (0.18 M), 0 °C to r.t., 18 h

Table 6: Martin's sulfurane optimization: Effect of concentration in the reaction with CyBpin.

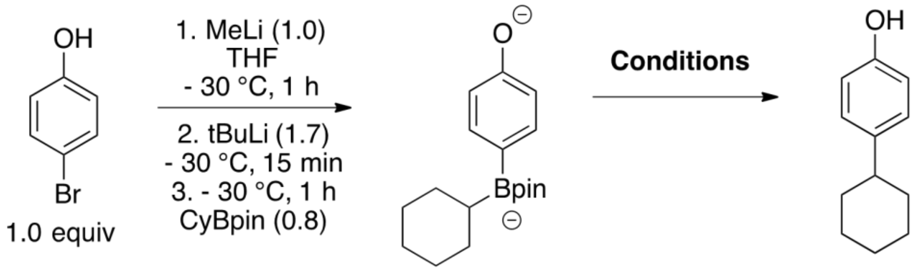

Equivalents Martin's sulfurane[b]	NMR yield (%)
1.1	54
**1.0**	**62 (58% isolated)**
0.8	45
1.0 - Concentration = 0.25 M	49
1.0 - **Concentration = 0.18 M**	62
1.0 - Concentration = 0.1 M	59
1.0 - Slow addition of reagent	59

[b] Martin's sulfurane, THF (0.18 M), -30 °C to r.t., 18 h

Table 7: Martin's sulfurane optimization: Effect of base stoichiometry in the reaction with CyBpin.

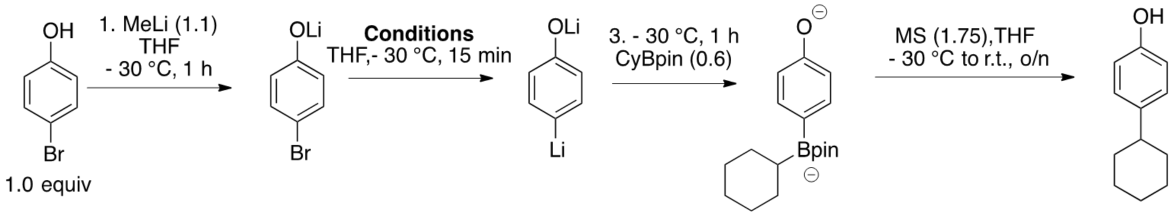

tBuLi equivalents	NMR yield (%)
2.0	55
1.7	74
1.5	64
**Equivalents of TMEDA**[a]	**NMR Yield (%)**
3.0	12
2.0	22
1.0	19
0.0	22

[a] Using 2.0 equivalents tBuLi

Table 8: Ph_3BiF_2 optimisation: Screening different conditions for the reaction CyBpin.

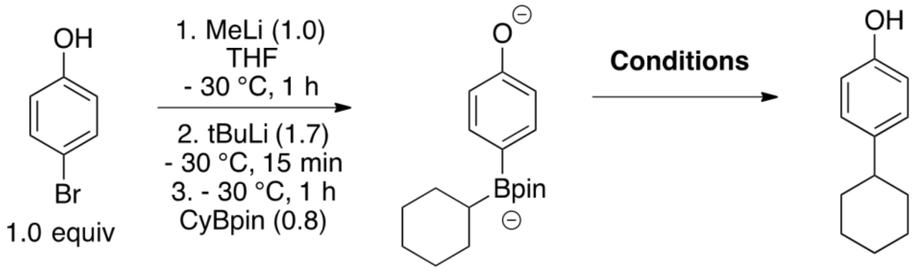

Ph_3BiF_2 equivalents[a]	NMR yield (%)
2.5	55
**2.1**	**63**
1.5	15
1.1	14
**Conditions**[b]	**NMR Yield (%)**
0 °C to r.t. (solution)	53
0 °C to r.t. (solid)	60
**Concentration**[c]	**NMR yield (%)**
0.25 M	46
0.14 M	55
0.1 M	53

[a] 30 °C, 90 mins. [b] Using 2.1 equiv. Ph_3BiF_2 [c] Using 2.1 equiv. Ph_3BiF_2 at -30 °C for 90 mins

4. General Procedures

<u>General Procedure A</u>: C(sp^2)-C(sp^3) coupling of boronic esters with phenol using Martin's sulfurane

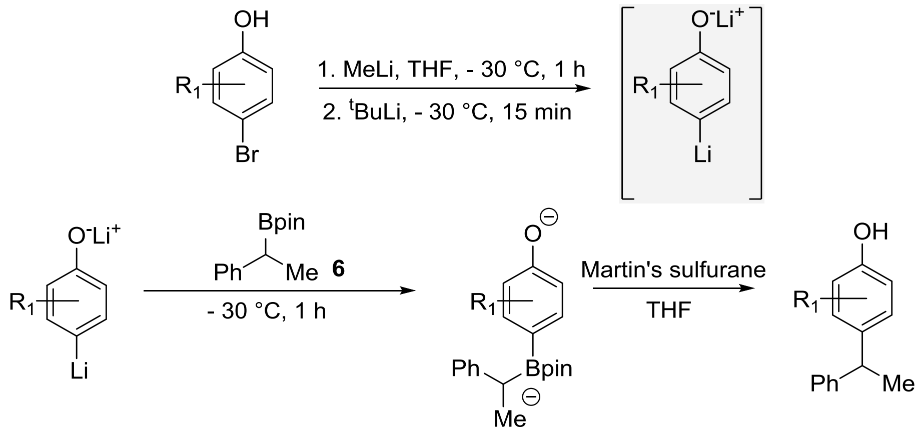

A solution of phenol (0.200 mmol, 1.25 equiv.) in THF (0.8 mL, 0.2 M) was cooled to –30 °C and treated with MeLi (0.130 mL, 0.208 mmol 1.30 equiv., 1.6 M in Et_2O), and the mixture was stirred at –30 °C for 1 h. To the resulting solution, *t*BuLi (0.2 mL, 0.34 mmol, 2.1 equiv., 1.7 M in pentane) was added. The resulting suspension was stirred vigorously for 15 minutes at –30 °C. The boronic ester (0.16 mmol, 1.0 equiv.) was added dropwise neat or as a solution in THF (0.4 M). The mixture was stirred at –30 °C for 1 h at which point ^{11}B NMR spectroscopy showed complete formation of the 'ate' complex [^{11}B NMR (96 MHz, THF) ~ 8 ppm]. A solution of Martin's sulfurane (0.400 mL, 0.5 M in THF, 0.200 mmol, 1.25 equiv.) was added at –30 °C. After 18 h at –30 °C, saturated aqueous NH_4Cl solution (10 mL) was added. The reaction mixture was diluted with EtOAc (20 mL). The layers were separated and the aqueous layer was extracted with EtOAc (3 x 20 mL). The combined organic layers were washed with brine (30 mL), dried over $MgSO_4$, filtered and concentrated under vacuum. The crude material was purified by automated flash column chromatography on silica gel (Biotage SNAP KP – 5 g) eluting with a slow gradient of pet. ether:EtOAc (100:0 to 95:0).

General Procedure B: $C(sp^2)$-$C(sp^3)$ coupling of boronic esters with phenol using Ph_3BiF_2

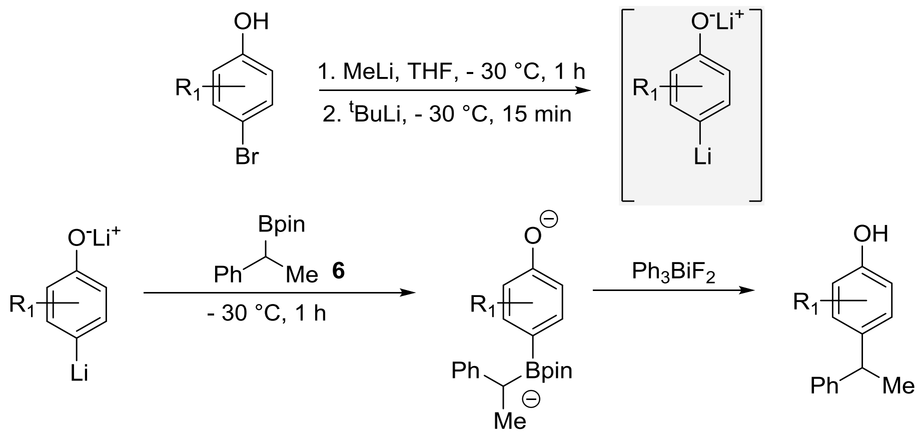

A solution of phenol (0.200 mmol, 1.25 equiv.) in THF (0.8 mL, 0.2 M) was cooled to –30 °C and treated with MeLi (0.130 mL, 0.208 mmol, 1.30 equiv., 1.6 M in Et_2O), and the mixture was stirred at –30 °C for 1 h. To the resulting solution, *t*BuLi (0.20 mL, 0.34 mmol, 2.1 equiv., 1.7 M in pentane) was added. The resulting suspension was stirred vigorously for 15 minutes at –30 °C. The boronic ester (0.16 mmol, 1.0 equiv.) was added dropwise neat or as a solution in THF (0.4 M). The mixture was stirred at –30 °C for 1 h at which point ^{11}B NMR spectroscopy showed complete formation of the 'ate' complex [^{11}B NMR (96 MHz, THF) ~ 8 ppm]. Ph_3BiF_2 (190 mg, 0.400 mmol, 2.50 equiv.) was added at –30 °C. After 18 h at –30 °C, saturated aqueous NH_4Cl solution (10 mL) was added. The reaction mixture was diluted with EtOAc (20 mL). The layers were separated and the aqueous layer was extracted with EtOAc (3 x 20 mL). The combined organic layers were washed with brine (30 mL), dried over $MgSO_4$, filtered and concentrated under vacuum. The crude material purified by automated flash column chromatography on silica gel (Biotage SNAP KP – 5 g) eluting with a slow gradient of pet. ether:EtOAc (100:0 to 95:0).

<u>General Procedure C</u>: C(sp^2)-C(sp^3) coupling of boronic esters with ArOBt

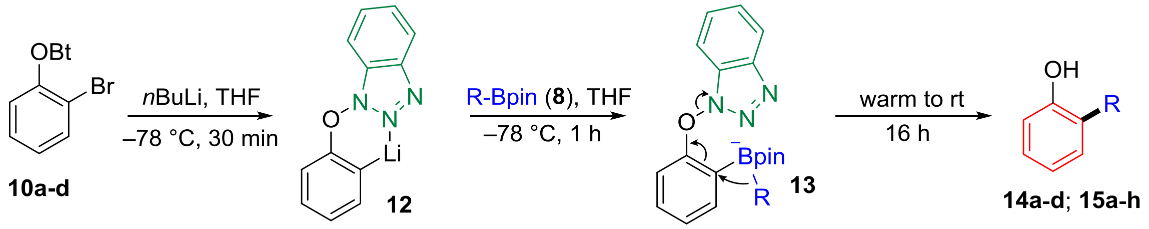

A solution of *o*-Br-ArOBt (0.200 mmol, 1.25 equiv.) in THF (0.8 mL, 0.2 M) was cooled to –78 °C and treated with *n*BuLi (0.125 mL, 0.200 mmol 1.25 equiv., 1.6 M in Et_2O), and the mixture was stirred at –78 °C for 30 min. To this mixture a solution of boronic ester (0.16 mmol, 1.0 equiv.) in THF (0.8 mL) was added dropwise. The mixture was stirred at –78 °C for 1 h at which point ^{11}B NMR spectroscopy showed complete formation of the 'ate' complex [^{11}B NMR (96 MHz, THF) ~ 8 ppm]. Then, the reaction mixture was allowed to warm slowly to rt. After 18 h, the reaction was quenched with 3 N aqueous HCl (3 mL). The reaction mixture was diluted with EtOAc (20 mL) and washed with water. The layers were separated and the aqueous layer was extracted with EtOAc (3 x 20 mL). The combined organic layers were washed with brine (30 mL), dried over Na_2SO_4, filtered and concentrated under vacuum. The crude material purified by flash column chromatography on silica gel to afford the desired product.

5. Compound Characterization Data

4-(1-Phenylethyl)phenol (**7a/9e**)

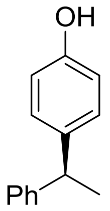

The starting boronic ester (0.16 mmol) was reacted according to General Procedure A (20 mg; 62%, 100% es) and General Procedure B (14 mg; 44%, 100% es), to afford the title compound as yellow wax. The spectral data matched that previously reported in the literature.[10]

$[\alpha]_D^{23}$ = + 5.8 (c 0.5, $CHCl_3$); R_f (15% EtOAc/pet. ether): 0.24; IR (film) ν_{max}/cm^{-1}: 3529, 3354, 2967, 2926, 1613, 1512, 1377, 1238, 1066, 834; ^{1}H NMR (400 MHz, $CDCl_3$) 7.34 – 7.15 (m, 5H), 7.09 (dd, *J* = 8.5, 1.9 Hz, 2H), 6.75 (dd, *J* = 8.5, 1.9 Hz, 2H), 4.86 (s, 1H), 4.10 (q, *J* = 7.2 Hz, 1H), 1.62 (d, *J* = 7.2 Hz, 3H); ^{13}C NMR (101 MHz, $CDCl_3$) 153.7, 146.8, 138.8, 128.8, 128.4, 127.6, 126.0, 115.2, 44.0, 22.1; HRMS (EI^+) mass calculated for $[M]^+$ $C_{14}H_{14}O$ requires m/z 198.1045, found m/z 198.1041. The er was determined by SFC [chiralpak IB, 10% of hexane/isopropanol 50/50, 125 bar, 4.0 mL/min, 40 °C, t (major) = 8.9 min, t (minor) = 9.5 min] to be 98:2 (100% es).

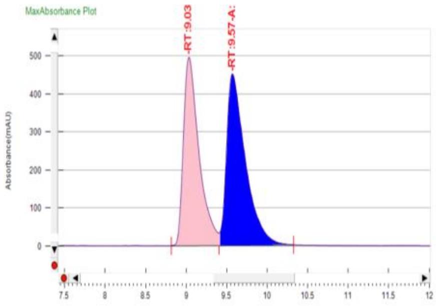

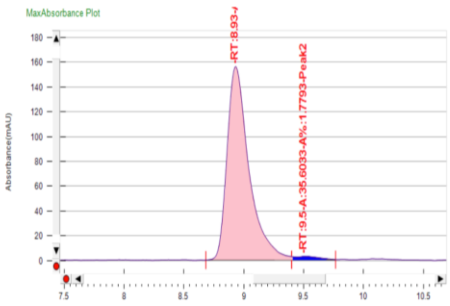

Peak Information

Peak No	% Area	Area	Ret. Time	Height	Cap. Factor
1	47.7131	6577.8537	9.03 min	495.0629	0
2	52.2869	7208.4207	9.57 min	450.2555	0

Peak Information

Peak No	% Area	Area	Ret. Time	Height	Cap. Factor
1	98.2207	1965.354	8.93 min	156.291	8932.2
2	1.7793	35.6033	9.5 min	2.6732	9498.8667

2-Methyl-4-(1-phenylethyl)phenol (**7b/9f**)

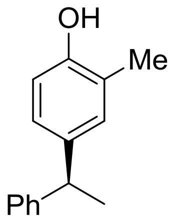

The starting boronic ester (0.16 mmol) was reacted according to General Procedure A (21 mg; 63%, 100% es) and General Procedure B (15 mg; 43%, 100% es), to afford the title compound as yellow oil. The spectral data matched that previously reported in the literature.[10]

$[\alpha]_D^{23}$ = +10.1 (c 0.7, $CHCl_3$); R_f (15% EtOAc/pet. ether): 0.28; IR (film) ν_{max}/cm^{-1}: 3412, 2969, 1520, 1394, 1255, 1061, 850; ^{1}H NMR (400 MHz, $CDCl_3$) 7.31 – 7.15 (m, 5H), 6.98 (d, *J* = 2.0 Hz, 1H), 6.93 (m, 1H), 6.69 (d, *J* = 8.2 Hz, 1H), 4.58 (s, 1H), 4.07 (q, *J* = 7.2 Hz, 1H), 2.21 (s, 3H), 1.61 (d, *J* = 7.2 Hz, 3H). The er was determined by SFC [chiralpak IC, 5% of hexane/isopropanol 50/50, 125 bar, 4.0 mL/min, 40 °C, t (minor) = 10.7 min, t (major) = 11.8 min] to be 98:2 (100% es).

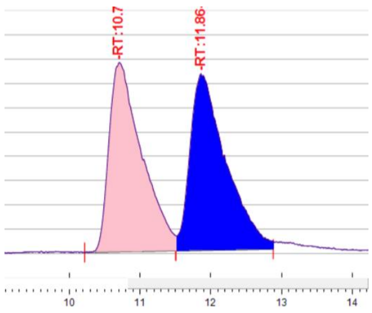

Peak Information

Peak No	% Area	Area	Ret. Time	Height	Cap. Factor
1	49.3565	12491.192	10.71 min	389.6902	10708.5
2	50.6435	12816.9019	11.86 min	363.0454	11858.4167

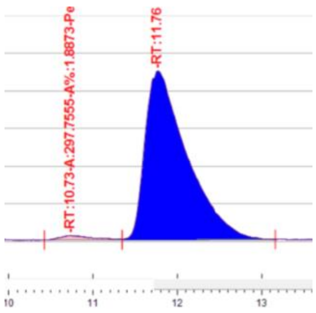

Peak Information

Peak No	% Area	Area	Ret. Time	Height	Cap. Factor
1	1.8873	297.7555	10.73 min	11.4609	10731.8333
2	98.1127	15479.4364	11.76 min	449.263	11761.7667

3-Methyl-4-(1-phenylethyl)phenol (**7c**)

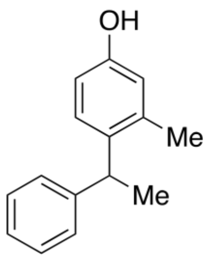

The starting boronic ester (0.16 mmol) was reacted according to General Procedure A (21 mg; 60%) and General Procedure B (19 mg; 58%), to afford the title compound as off-white solid.

Mpt: 124-126 °C; R_f (15% EtOAc/pet. ether): 0.22; IR (film) ν_{max}/cm^{-1}: 3334, 2968, 2900, 1608, 1500, 1451, 1258, 1060, 818; ^{1}H NMR (400 MHz, $CDCl_3$) 7.30 – 7.23 (m, 2H), 7.20 – 7.13 (m, 4H), 6.68 (dd, *J* = 8.3, 2.8 Hz, 1H), 6.64 (d, *J* = 2.8 Hz, 1H), 4.72 (s, 1H), 4.25 (q, *J* = 7.2 Hz, 1H), 2.18 (s, 3H), 1.59 (d, *J* = 7.2 Hz, 3H); ^{13}C NMR (101 MHz, $CDCl_3$) 153.6, 146.7, 137.9, 136.4, 128.4, 127.9, 127.6, 125.8, 117.3, 112.6, 40.4, 22.4, 19.9; HRMS (EI$^+$) mass calculated for $[M]^+$ $C_{15}H_{16}O$ requires m/z 212.1201, found m/z 212.1207.

2,5-Dimethyl-4-(1-phenylethyl)phenol (**7d**)

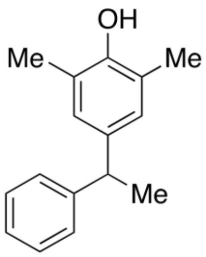

The starting boronic ester (0.16 mmol) was reacted according to General Procedure A (10 mg; 29%) and General Procedure B (18 mg; 49%), to afford the title compound as yellow oil. The spectral data matched that previously reported in the literature.[10]

R_f (15% EtOAc/pet. ether): 0.34; IR (film) ν_{max}/cm^{-1}: 3666, 3573, 2970, 2904, 1395, 1248, 1061, 870; ^{1}H NMR (400 MHz, $CDCl_3$) 7.32 – 7.21 (m, 4H), 7.21 – 7.15 (m, 1H), 6.84 (s, 2H), 4.51 – 4.46 (m, 1H), 4.04 (q, *J* = 7.2 Hz, 1H), 2.23 – 2.20 (m, 6H), 1.60 (d, *J* = 7.2 Hz, 3H).

3,5-Dimethyl-4-(1-phenylethyl)phenol (7e)

The starting boronic ester (0.16 mmol) was reacted according to General Procedure A (15 mg; 42%) and General Procedure B (11 mg; 30%), to afford the title compound as colourless waxy oil.

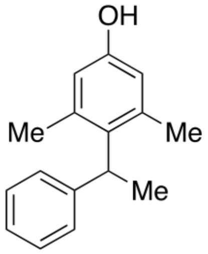

R_f (15% EtOAc/pet. ether): 0.19; IR (film) ν_{max}/cm^{-1}: 3358, 2972, 2902, 1650, 1540, 1394, 1247, 1061, 870; ^{1}H NMR (400 MHz, $CDCl_3$) 7.29 – 7.25 (m, 2H), 7.18 – 7.16 (m, 3H), 6.50 (s, 2H), 4.59 (q, *J* = 7.3 Hz, 1H), 4.50 (s, 1H), 2.10 (s, 6H), 1.65 (d, *J* = 7.3 Hz, 3H); ^{13}C NMR (101 MHz, $CDCl_3$) 153.2, 145.6, 138.4, 128.2, 126.9, 125.4, 115.8, 100.1, 37.5, 21.3, 17.1; HRMS (EI^+) mass calculated for $[M]^+$ $C_{16}H_{18}O$ requires m/z 226.1358, found m/z 226.1347.

4-(1-Phenylethyl)naphthalen-1-ol (7f)

The starting boronic ester (0.16 mmol) was reacted according to General Procedure A (19 mg; 48%) and General Procedure B (12 mg; 29%), to afford the title compound as red oily wax. The spectral data matched that previously reported in the literature.[11]

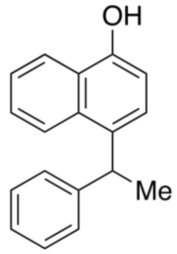

R_f (15% EtOAc/pet. ether): 0.2; IR (film) ν_{max}/cm^{-1}: 3389, 2961, 2923, 2853, 1656, 1588, 1450, 1378, 1263, 760; ^{1}H NMR (400 MHz, $CDCl_3$) 8.21 (d, *J* = 9.3 Hz, 1H), 7.97 (d, *J* = 9.3 Hz, 1H), 7.46 – 7.41 (m, 2H), 7.26 - 7.22 (m, 5H), 7.15 (d, *J* = 7.0 Hz, 1H), 6.81 (d, *J* = 7.0 Hz, 1H), 5.18 (s, 1H), 4.83 (q, *J* = 7.1 Hz, 1H), 1.73 (d, *J* = 7.1 Hz, 3H).

2-Methoxy-4-(1-phenylethyl)phenol (7g)

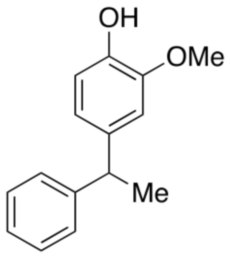

The starting boronic ester (0.16 mmol) was reacted according to General Procedure A (20 mg; 55%) and General Procedure B (23 mg; 62%), to afford the title compound as yellow oil.

R_f (15% EtOAc/pet. ether): 0.12; IR (film) ν_{max}/cm^{-1}: 3673, 3527, 3417, 2966, 2902, 1519, 1413, 1260, 1051, 799; ^{1}H NMR (400 MHz, $CDCl_3$) 7.34 – 7.13 (m, 5H), 6.84 (dd, *J* = 8.2, 1.2 Hz, 1H), 6.77 – 6.72 (m, 1H), 6.71 – 6.66 (m, 1H), 5.46 (s, 1H), 4.08 (q, *J* = 7.2 Hz, 1H), 3.82 (s, 3H), 1.61 (d, *J* = 7.2 Hz, 3H); ^{13}C NMR (101 MHz, $CDCl_3$) 146.8, 146.5, 143.9, 138.5, 128.4, 127.6, 126.1, 120.2, 114.2, 110.5, 55.9, 44.5, 22.2; HRMS (EI^+) mass calculated for $[M]^+$ $C_{15}H_{16}O_2$ requires m/z 228.1150, found m/z 228.1160.

2-Fluoro-4-(1-phenylethyl)phenol (**7h**)

The starting boronic ester (0.16 mmol) was reacted according to General Procedure A (15 mg; 42%) and General Procedure B (17 mg; 48%), to afford the title compound as colorless wax.

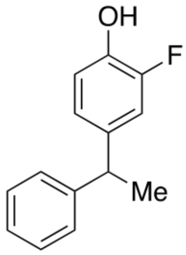

R_f (15% EtOAc/pet. ether) 0.21; IR (film) ν_{max}/cm^{-1}: 3666, 3410, 2971, 1602, 1518, 1437, 1234, 1060, 870; ^{1}H NMR (400 MHz, $CDCl_3$) 7.32 – 7.26 (m, 2H), 7.22 – 7.16 (m, 3H), 6.95 – 6.87 (m, 3H), 5.01 (s, 1H), 4.07 (q, *J* = 7.3 Hz, 1H), 1.60 (d, *J* = 7.3 Hz, 3H); ^{13}C NMR (101 MHz, $CDCl_3$) 151.0 (d, *J* = 237 Hz), 146.1, 141.6 (d, *J* = 14.3 Hz), 139.7 (d, *J* = 4.7 Hz), 128.6, 127.6, 126.3, 123.9 (d, *J* = 3.1 Hz), 117.0, 114.7 (d, *J* = 18.5 Hz), 44.0, 22.0; ^{19}F NMR (377 MHz, $CDCl_3$) -140.8 (m); HRMS (EI$^+$) mass calculated for [M]$^+$ $C_{14}H_{13}OF$ requires m/z 216.0950, found m/z 216.0955.

4-(1-Phenylethyl)-2-(trifluoromethyl)phenol (**7i**)

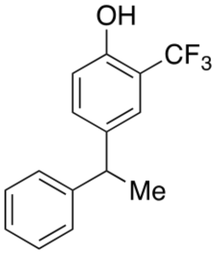

The starting boronic ester (0.16 mmol) was reacted according to General Procedure A (18 mg; 41%) and General Procedure B (10 mg; 24%), to afford the title compound as colourless oil.

R_f (15% EtOAc/pet. ether): 0.16; IR (film) ν_{max}/cm^{-1}: 3524, 2932, 1621, 1509, 1437, 1316, 1121, 1053, 904, 698; ^{1}H NMR (400 MHz, $CDCl_3$) 7.36 (s, 1H), 7.32 – 7.26 (m, 2H), 7.25 (m, 4H), 6.86 (d, *J* = 8.4 Hz, 1H), 5.43 (s, 1H), 4.12 (q, *J* = 7.2 Hz, 1H), 1.61 (d, *J* = 7.2, 3H); ^{13}C NMR (101 MHz, $CDCl_3$) 151.8, 145.8, 139.0, 132.8, 128.6, 127.6, 126.4, 125.6 (q, *J* = 4.9 Hz), 123.0, 117.9, 116.2 (d, *J* = 30 Hz), 43.9, 22.0; ^{19}F NMR (377 MHz, $CDCl_3$) -60.6; HRMS (EI$^+$) mass calculated for [M]$^+$ $C_{15}H_{13}OF_3$ requires m/z 266.0918, found m/z 266.0913.

4-Isobutylphenol (**9a**)

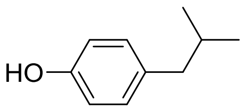

The starting boronic ester (0.16 mmol) was reacted according to General Procedure A (63% NMR yield) to afford the title compound as oil. The spectral data matched with the commercially available sample.[12]

R_f (10% EtOAc/pet. ether): 0.30; ^{1}H NMR (400 MHz, $CDCl_3$) δ 7.29 – 7.09 (m, 5H), 7.06 (d, *J* = 8.2 Hz, 2H), 6.71 (d, *J* = 8.6 Hz, 2H), 4.66 (s, 1H), 2.02 (d, *J* = 3.4 Hz, 2H), 1.59 – 1.43 (m, 1H), 0.73 (d, *J* = 8.8 Hz, 6H).

4-Phenethylphenol (**9b**)

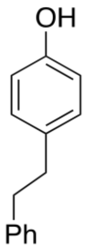

The starting boronic ester (0.16 mmol) was reacted according to General Procedure A (17 mg; 53%), to afford the title compound as white amorphous solid/wax. The spectral data matched that previously reported in the literature.[13]

R_f (15% EtOAc/pet. ether): 0.20; IR (film) ν_{max}/cm^{-1}: 3471, 2981, 2934, 1737, 1373, 1236, 1045; ^{1}H NMR (400 MHz, $CDCl_3$) 7.22 – 7.16 (m, 2H), 7.14 – 7.06 (m, 3H), 7.00 – 6.93 (m, 2H), 6.70 – 6.63 (m, 2H), 4.55 (s, 1H), 2.84 – 2.74 (m, 4H).

4-Cyclohexylphenol (**9c**)

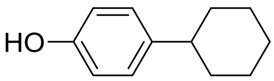

The starting boronic ester (0.16 mmol) was reacted according to General Procedure A (16 mg; 58%) to afford the title compound as off-white solid. The spectral data matched with the commercially available sample.

R_f (10% EtOAc/pet. ether): 0.30; ^{1}H NMR (400 MHz, $CDCl_3$) δ 7.11 (d, *J* = 8.4 Hz, 2H), 6.73 (d, *J* = 8.4 Hz, 2H), 4.60 (s, 1H), 2.45-2.41 (m, 1H), 1.91 – 1.37 (m, 10H).

(*S*)-4-(4-Phenylbutan-2-yl)phenol (**9d**)

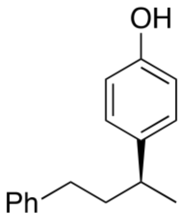

The starting boronic ester (0.16 mmol) was reacted according to General Procedure A (22 mg; 60%, 100% es) to afford the title compound as yellow oil.

$[\alpha]_D^{23}$ = +24 (c 0.3, $CHCl_3$); R_f (15% EtOAc/pet. ether): 0.24; IR (film) ν_{max}/cm^{-1}: 3317, 2969, 2925, 1519, 1398, 1255, 1061, 890; ^{1}H NMR (400 MHz, $CDCl_3$) 7.28 – 7.22 (m, 3H), 7.18 – 7.15 (m, 1H), 7.14 – 7.10 (m, 2H), 7.06 (dd, *J* = 8.4, 2.0 Hz, 1H), 6.80 – 6.75 (dd, *J* = 8.4, 2.0 Hz, 2H), 4.69 (s, 1H), 2.66 (sx, *J* = 7.2 Hz, 1H), 2.53 – 2.46 (m, 2H), 1.89 – 1.83 (m, 2H), 1.23 (d, *J* = 7.2 Hz, 3H); ^{13}C NMR (101 MHz, $CDCl_3$) 153.7, 142.7, 139.7, 128.5, 128.4, 128.2, 125.7, 115.3, 40.3, 38.8, 34.0, 22.8; HRMS (EI$^+$) mass calculated for $[M]^+$ $C_{16}H_{18}O$ requires m/z 226.1358, found m/z 226.1351. The er was determined by SFC [chiralpak IA, 20% of hexane/isopropanol 50/50, 125 bar, 2.0 mL/min, 40 °C, t (minor) = 14.9 min, t (major) = 15.81 min] to be 96:4 (100% es).

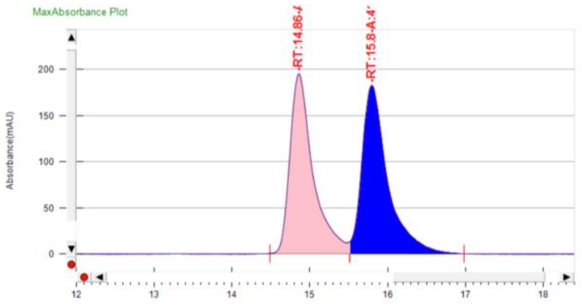

Peak Information

Peak No	% Area	Area	Ret. Time	Height	Cap. Factor
1	49.0601	4016.9263	14.86 min	194.5102	14858.2
2	50.9399	4170.847	15.8 min	181.8057	15794.8

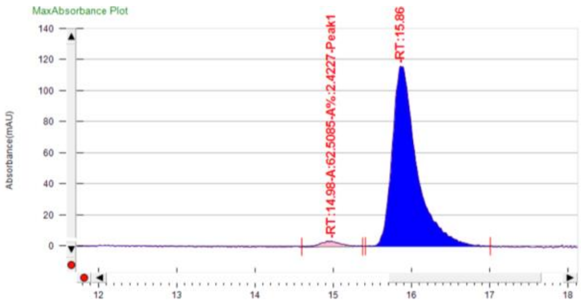

Peak Information

Peak No	% Area	Area	Ret. Time	Height	Cap. Factor
1	2.4227	62.5085	14.98 min	3.4737	14974.8667
2	97.5773	2517.6065	15.86 min	115.8786	15854.8

(*S*)-4-(3-Methyl-1-phenylpentan-3-yl)phenol (9g)

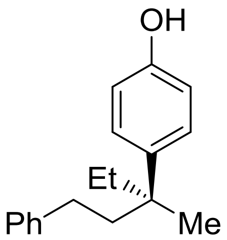

The starting boronic ester (0.16 mmol) was reacted according to General Procedure A (70%, 100% es) to afford the title compound as yellow oil

$[\alpha]_D^{23}$ = -41 (c 0.9, $CHCl_3$); R_f (15% EtOAc/pet. ether): 0.27; IR (film) ν_{max}/cm^{-1}: 3358, 3026, 2965, 1611, 1513, 1495, 1227, 1180, 827; ^1H NMR (400 MHz, $CDCl_3$) 7.28 – 7.18 (m, 4H), 7.19 – 7.13 (m, 1H), 7.13 – 7.07 (m, 2H), 6.84 – 6.79 (m, 2H), 4.73 (s, 1H), 2.44 (td, *J* = 13.0, 5.3 Hz, 1H), 2.26 (td, *J* = 13.0, 4.3 Hz, 1H), 1.97 (td, *J* = 13.0, 4.3 Hz, 1H), 1.87 – 1.54 (m, 3H), 1.33 (s, 3H), 0.71 (td, *J* = 7.5, 1.2 Hz, 3H); ^{13}C NMR (101 MHz, $CDCl_3$) 153.2, 143.4, 139.7, 128.4, 128.4, 127.8, 125.6, 115.0, 45.5, 40.8, 35.9, 31.0, 23.6, 8.8; HRMS (EI^+) mass calculated for $[M]^+$ $C_{18}H_{22}O$ requires m/z 254.1671, found m/z 254.1677. The er was determined by SFC [chiralpak IA, 10% of hexane/isopropanol 50/50, 125 bar, 4.0 mL/min, 40 °C, t (major) = 15.7 min, t (minor) = 17.9 min] to be 99:1 (100% es).

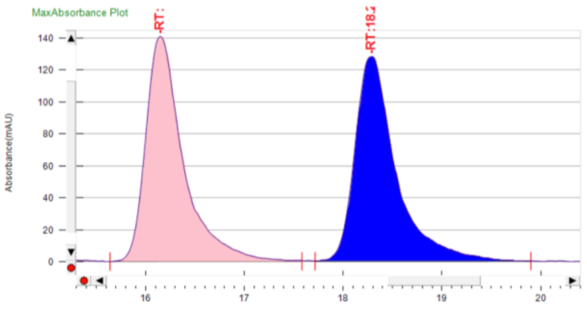

Peak Information

Peak No	% Area	Area	Ret. Time	Height	Cap. Factor
1	50.1388	3702.745	16.15 min	140.5426	16148.7667
2	49.8612	3682.2466	18.29 min	127.9842	18290.4

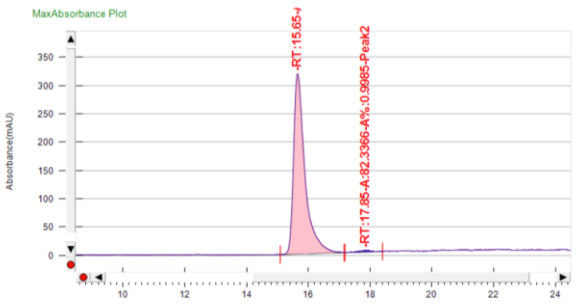

Peak Information

Peak No	% Area	Area	Ret. Time	Height	Cap. Factor
1	99.0015	8163.5679	15.65 min	318.7639	15648.7667
2	0.9985	82.3366	17.85 min	3.3125	17848.7333

(*R*)-4-(4-Methyl-2-phenylpentan-2-yl)phenol (9h)

The starting boronic ester (0.16 mmol) was reacted according to General Procedure A (54%, 96% es) and General Procedure B (33%, 98% es) to afford the title compound as yellow oil.

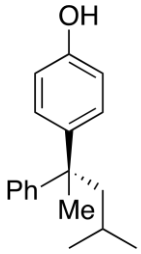

$[\alpha]_D^{23}$ = +80 (c 0.1, $CHCl_3$); R_f (15% EtOAc/pet. ether): 0.15; IR (film) ν_{max}/cm^{-1}: 3350, 2957, 2868, 1612, 1511, 1376, 1258, 1179, 830; ^1H NMR (400 MHz, $CDCl_3$) 7.27 – 7.17 (m, 4H), 7.18 – 7.12 (m, 1H), 7.08 – 7.04 (m, 2H), 6.77 – 6.66 (m, 2H), 4.66 (s, 1H), 2.02 (dd, *J* = 5.0, 0.8 Hz, 2H), 1.62 (s, 3H), 1.52 (tq, *J* = 6.7, 5.0 Hz, 1H), 0.73 (d, *J* = 6.7 Hz 3H), 0.72 (d, *J* = 6.7 Hz, 3H); ^{13}C NMR (101 MHz, $CDCl_3$) 153.3, 150.5, 142.6, 128.8, 127.9, 127.5, 125.6, 114.7, 50.6, 46.1, 28.4, 25.3, 25.3, 24.8; HRMS (EI^+) mass calculated for $[M]^+$ $C_{18}H_{22}O$ requires m/z 254.1671, found m/z 254.1661. The er was determined by HPLC [chiralpak IA with guard, 5% of isopropanol in hexane, 210 nm, 1.0 mL/min, 25 °C, t (major) = 7.8 min, t (minor) = 8.7 min] to be 97:3 (98% es).

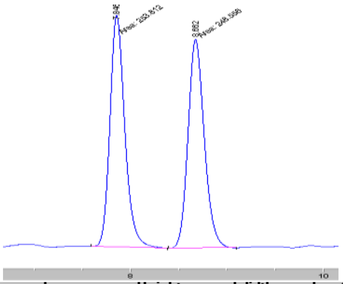

#	Time	Area	Height	Width	Area%	Symmetry
1	7.846	253.8	23.5	0.1802	50.523	0.819
2	8.662	248.6	21.2	0.1959	49.477	0.842

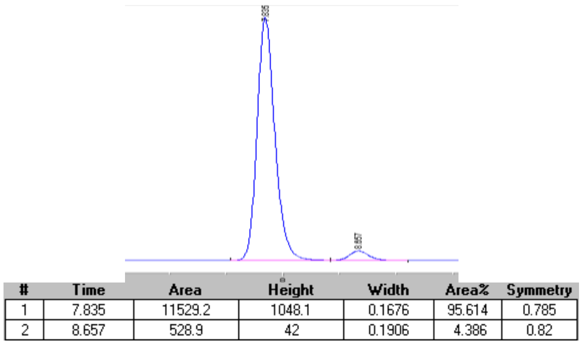

#	Time	Area	Height	Width	Area%	Symmetry
1	7.835	11529.2	1048.1	0.1676	95.614	0.785
2	8.657	528.9	42	0.1906	4.386	0.82

(*S*)-4-(1-Phenylhept-6-en-3-yl)phenol (9i)

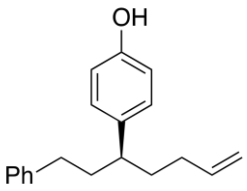

The starting boronic ester (0.16 mmol) was reacted according to General Procedure A (68%, 98% es) to afford the title compound as colourless viscous oil.

$[\alpha]_D^{23}$ = -5.5 (c 1.1, CH_2Cl_2); R_f (15% EtOAc/pet. ether): 0.30; IR (film) ν_{max}/cm^{-1}: 3341, 2922, 2854, 1612, 1512, 1452, 1224, 909, 830, 698 ^{1}H NMR (400 MHz, $CDCl_3$) 7.28 – 7.23 (m, 2H), 7.19 – 7.15 (m 1H), 7.15 – 7.07 (m, 2H), 7.07 – 7.02 (m, 2H), 6.82 – 6.78 (m, 2H), 5.76 (ddt, *J* = 16.9, 9.7, 6.7 Hz, 1H), 4.99 – 4.87 (m, 2H), 4.81 (s, 1H), 2.53 – 2.42 (m, 3H), 1.98 – 1.81 (m, 4H), 1.75 – 1.67 (m, 1H), 1.66 – 1.59 (m, 1H); ^{13}C NMR (101 MHz, $CDCl_3$) 153.8, 142.7, 138.9, 137.4, 128.9, 128.4, 128.3, 125.7, 115.3, 114.5, 44.2, 38.8, 36.4, 33.9, 31.8; HRMS (EI$^+$) mass calculated for $[M]^+$ $C_{19}H_{22}O$ requires m/z 266.1671, found m/z 266.1665. The er was determined by SFC [chiralpak IB, 10% of hexane/isopropanol 50/50, 125 bar, 2.0 mL/min, 40 °C, t (major) = 18.9 min, t (minor) = 19.9 min] to be 95:5 (98% es).

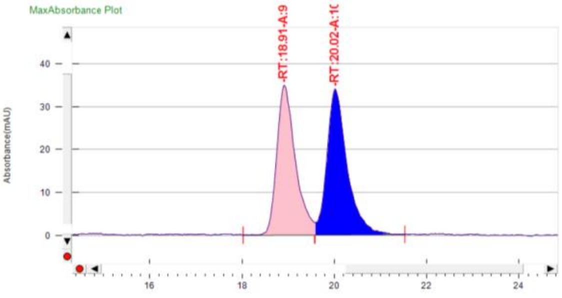

Peak Information

Peak No	% Area	Area	Ret. Time	Height	Cap. Factor
1	48.7676	974.599	18.91 min	34.9149	18907.05
2	51.2324	1023.8579	20.02 min	33.9955	20015.3667

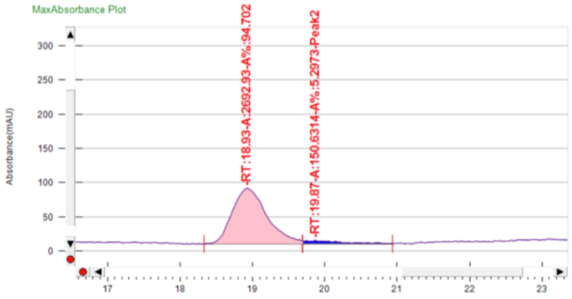

Peak Information

Peak No	% Area	Area	Ret. Time	Height	Cap. Factor
1	94.7027	2692.93	18.93 min	81.2157	18932.05
2	5.2973	150.6314	19.87 min	4.7465	19865.3667

(*S*)-4-(1-Cyclopropyl-3-phenylpropyl)phenol (9j)

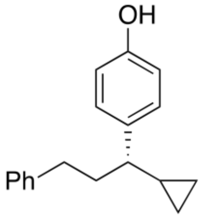

The starting boronic ester (0.16 mmol) was reacted according to General Procedure A (28 mg; 68%, 100% es) to afford the title compound as colourless oil.

$[\alpha]_D^{23}$ = +2 (c 0.9, $CHCl_3$); R_f (15% EtOAc/pet. ether): 0.26; IR (film) ν_{max}/cm^{-1}: 3350, 3025, 2999, 2919, 1597, 1511, 1490, 1222, 1015, 823; ^{1}H NMR (400 MHz, $CDCl_3$) 7.29 - 7.25 (m, 2H), 7.20 – 7.16 (m, 1H), 7.13 (d, *J* = 7.4 Hz, 2H), 7.11 – 7.07 (m, 2H), 6.85 – 6.78 (m,

2H), 4.76 (d, J = 15.9 Hz, 1H), 2.53 (ddd, J = 9.4, 6.8, 3.3 Hz, 2H), 2.18 – 1.94 (m, 2H), 1.77 (td, J = 9.4, 5.6 Hz, 1H), 0.96 (qt, J = 8.3, 4.7 Hz, 1H), 0.62 – 0.54 (m, 1H), 0.36 (ddd, J = 12.8, 9.1, 4.7 Hz, 1H), 0.19 (dq, J = 9.6, 4.7 Hz, 1H), 0.05 (dq, J = 9.6, 4.7 Hz, 1H); ^{13}C NMR (101 MHz, $CDCl_3$) 153.8, 142.8, 137.9, 128.8, 128.5, 128.4, 125.7, 115.2, 49.6, 38.4, 33.8, 17.9, 5.6, 3.7; HRMS (EI$^+$) mass calculated for $[M]^+$ $C_{18}H_{20}O$ requires m/z 252.1514, found m/z 252.1504. The er was determined by SFC [chiralpak IC, 10% of hexane/isopropanol 50/50, 125 bar, 4.0 mL/min, 40 °C, t (major) = 9.2 min, t (minor) = 10.6 min] to be 98:2 (100% es).

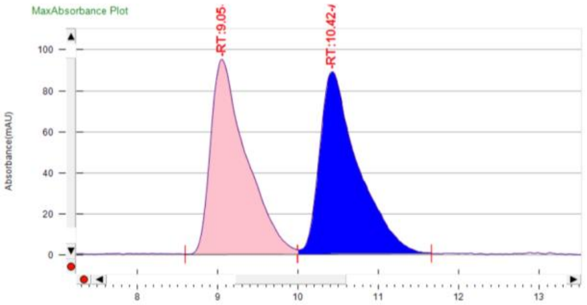

Peak Information

Peak No	% Area	Area	Ret. Time	Height	Cap. Factor
1	49.8106	2918.7507	9.05 min	95.1676	9048.8667
2	50.1894	2940.9483	10.42 min	88.8952	10423.85

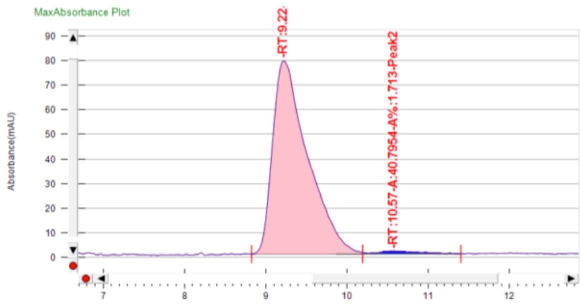

Peak Information

Peak No	% Area	Area	Ret. Time	Height	Cap. Factor
1	98.287	2340.7031	9.22 min	78.6581	9223.8667
2	1.713	40.7954	10.57 min	1.2015	10573.85

(*S*)-4-(9-((*tert*-Butyldimethylsilyl)oxy)-1-phenylnonan-3-yl)phenol (9k)

The starting boronic ester (0.16 mmol) was reacted according to General Procedure A (50 mg; 68%, 98% es) to afford the title compound as off-white viscous oil.

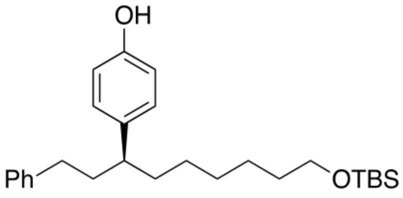

$[\alpha]_D^{23}$ = +53 (c 1.9, CH_2Cl_2); R_f (15% EtOAc/pet. ether): 0.30; IR (film) ν_{max}/cm^{-1}: 3337, 2927, 2855, 1513, 1256, 1096, 832, 775; ^{1}H NMR (400 MHz, $CDCl_3$) 7.25 – 7.19 (m, 2H), 7.19 – 7.13 (m, 1H), 7.13 – 7.09 (m, 2H), 7.05 – 7.00 (m, 2H), 6.81 – 6.76 (m, 2H), 5.15 (s, 1H), 3.63 - 3.56 (m, 3H), 2.85 – 2.77 (m, 1H), 2.49 – 2.42 (m, 3H), 1.98 – 1.89 (m, 1H), 1.87 – 1.73 (m, 2H), 1.55 – 1.45 (m, 5H), 1.36 – 1.30 (m, 2H), 0.90 (s, 9H), 0.05 (s, 6H); ^{13}C NMR (101 MHz, $CDCl_3$) 153.7, 142.8, 138.0, 128.8, 128.5, 128.3, 125.7, 115.2, 71.5, 63.4, 44.8, 38.9, 37.3, 33.9, 32.9, 29.6, 27.6, 26.1, 18.5, -5.1; HRMS (ESI$^+$) mass calculated for $[M+Na]^+$ $C_{27}H_{42}NaO_2Si$ requires m/z 449.2846, found m/z 449.2843. The er was determined by SFC [chiralpak IB, 10% of hexane/isopropanol 50/50, 125 bar, 4.0 mL/min, 40 °C, t (minor) = 12.8 min, t (major) = 13.9 min] to be 95:5 (98% es).

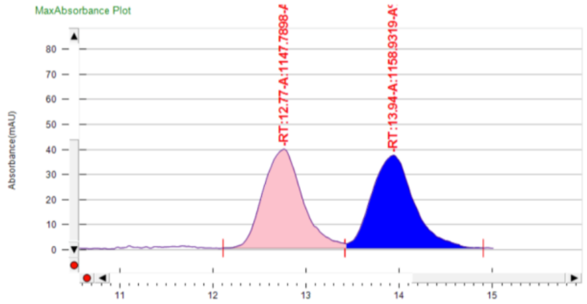

Peak Information

Peak No	% Area	Area	Ret. Time	Height	Cap. Factor
1	49.7585	1147.7898	12.77 min	39.5093	12765.4833
2	50.2415	1158.9319	13.94 min	37.0338	13940.4667

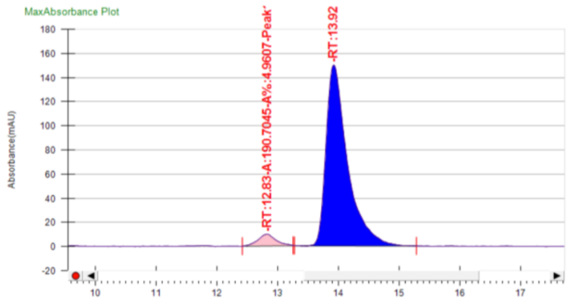

Peak Information

Peak No	% Area	Area	Ret. Time	Height	Cap. Factor
1	4.9607	190.7045	12.83 min	9.6052	12832.15
2	95.0393	3653.5661	13.92 min	149.4934	13915.4667

(*R*)-4-(7-Azido-1-phenylheptan-3-yl)phenol (**9l**)

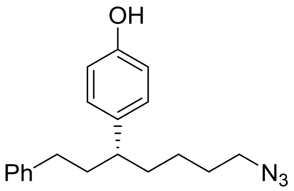

The starting boronic ester (0.16 mmol) was reacted according to General Procedure A (18 mg; 37%, 100% es) to afford the title compound as colourless viscous oil. $[\alpha]_D^{23}$ = +3.75 (c 0.8, $CHCl_3$); R_f (15% EtOAc/pet. ether): 0.40; IR (film) ν_{max}/cm^{-1}: 3676, 3396, 2925, 2096, 1512, 1241, 1066, 832; ^{1}H NMR (500 MHz, $CDCl_3$) 7.29 – 7.22 (m, 2H), 7.20 – 7.12 (m, 1H), 7.13 – 7.06 (m, 2H), 7.02 (d, *J* = 8.5 Hz, 2H), 6.79 (d, *J* = 8.6 Hz, 2H), 4.69 (s, 1H), 3.24 – 3.08 (m, 2H), 2.53 – 2.36 (m, 3H), 2.01 – 1.76 (m, 2H), 1.64 (m, 4H), 1.29 – 1.11 (m, 2H); ^{13}C NMR (126 MHz, $CDCl_3$) 153.7, 142.4, 137.2, 128.7, 128.3, 128.2, 125.6, 115.2, 51.3, 44.5, 38.6, 36.6, 33.7, 28.8, 24.6; HRMS (ESI$^+$) mass calculated for [M+Na]$^+$ $C_{19}H_{23}N_3NaO$ requires m/z 332.1733, found m/z 332.1736. The er was determined by SFC [chiralpak IB, 10% of hexane/isopropanol 50/50, 125 bar, 4.0 mL/min, 40 °C, t (minor) = 14.9 min, t (minor) = 16.0 min] to be 99:1 (100% es).

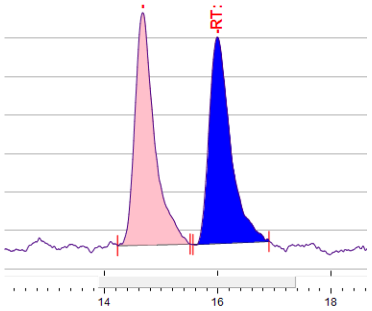

Peak No	% Area	Area	Ret. Time	Height	Cap. Factor
1	50.7693	1449.7616	14.67 min	60.474	14665.45
2	49.2307	1405.8262	15.99 min	53.7782	15990.4333

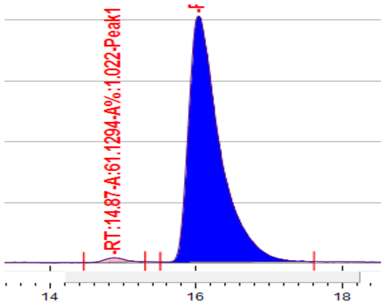

Peak No	% Area	Area	Ret. Time	Height	Cap. Factor
1	1.022	61.1294	14.87 min	3.4761	0
2	98.978	5920.1268	16.03 min	202.1148	0

(*S*)-4-(1-(1,3-Dioxolan-2-yl)-5-phenylpentan-3-yl)phenol (9m)

The starting boronic ester (0.16 mmol) was reacted according to General Procedure A (33 mg; 66%, 100% es) to afford the title compound as colourless viscous oil.

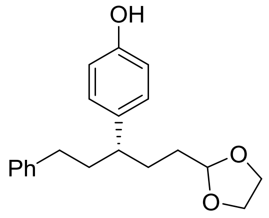

$[\alpha]_D^{23}$ = -10.2 (c 1.1, CH_2Cl_2); R_f (20% EtOAc/pet. ether): 0.30; IR (film) ν_{max}/cm^{-1}: 3677, 3371, 2924, 1513, 1224, 1133, 1029, 1833, 748; ^{1}H NMR (500 MHz, $CDCl_3$) 7.28 – 7.21 (m, 2H), 7.19 – 7.12 (m, 1H), 7.12 – 7.07 (m, 2H), 7.01 (d, *J* = 8.5 Hz, 2H), 6.75 (d, *J* = 8.5 Hz, 2H), 5.14 (s, 1H), 4.79 (t, *J* = 4.7 Hz, 1H), 4.01 – 3.73 (m, 4H), 2.55 – 2.36 (m, 3H), 1.95 (dddd, *J* = 14.5, 9.5, 7.0, 5.0 Hz, 1H), 1.82 (m, 2H), 1.63 (dtd, *J* = 13.0, 10.1, 9.5, 4.9 Hz, 1H), 1.58 – 1.42 (m, 2H); ^{13}C NMR (126 MHz, $CDCl_3$) 153.9, 142.5, 136.8, 128.7, 128.3, 128.2, 125.5, 115.2, 104.5, 64.8, 64.7, 44.5, 38.6, 33.7, 31.8, 31.1; HRMS (MALDI$^+$) mass calculated for $[M+Na]^+$ $C_{20}H_{24}O_3Na$ requires m/z 335.1618, found m/z 335.1611. The er was determined by SFC [Whelk-01, 20% of hexane/isopropanol 50/50, 125 bar, 4.0 mL/min, 40 °C, t (major) = 5.0 min, t (minor) = 5.52 min] to be 97:3 (100% es).

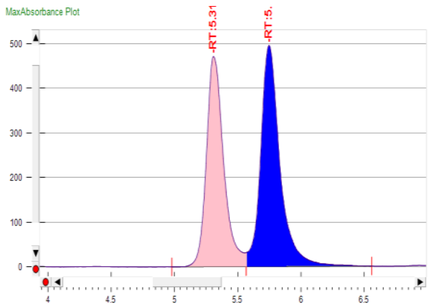

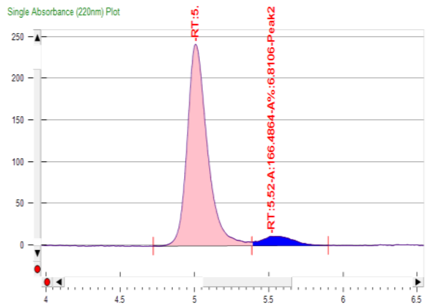

Peak No	% Area	Area	Ret. Time	Height
1	46.0456	4460.2024	5.31 min	470.8858
2	53.9544	5226.2862	5.75 min	495.3918

Peak No	% Area	Area	Ret. Time	Height
1	93.1894	2278.0485	5.01 min	241.9409
2	6.8106	166.4864	5.52 min	11.2079

***tert*-Butyl 4-(4-hydroxyphenyl)piperidine-1-carboxylate (9n)**

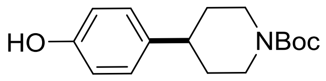

The starting boronic ester (0.16 mmol) was reacted according to General Procedure A (28 mg; 62%), to afford the title compound as colorless viscous oil. The spectral data matched that previously reported in the literature.[14] The product co-elutes with 1,1,1,3,3,3-hexafluoro-2-phenylpropan-2-ol [$Ph(CF_3)_2C$-OH], the by-product from Martin's Sulfurane.

R_f (3:1 EtOAc/pet. ether): 0.30; ^{1}H NMR (400 MHz, $CDCl_3$) 6.97 (d, *J* = 8.5 Hz, 2H), 6.73 (d, *J* = 8.6 Hz, 2H), 4.15 (d, *J* = 8.5 Hz, 2H), 2.72 (t, *J* = 12.3 Hz, 2H), 2.50 (tt, *J* = 12.1, 3.5 Hz, 1H), 1.73 (m, 2H), 1.49 (m, 2H), 1.42 (s, 9H).

4-((1*R*,2*S*,5*R*)-2-Isopropyl-5-methylcyclohexyl)phenol (**9o**)

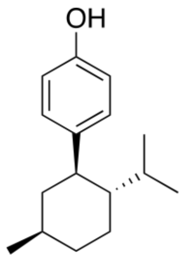

The starting boronic ester (0.16 mmol) was reacted according to General Procedure A (19 mg; 50%, >20:1 dr.) to afford the title compound as white solid.

$[\alpha]_D^{23}$ = -29 (c 0.6, $CHCl_3$); Mpt: 142-144 °C; R_f (15% EtOAc/pet. ether): 0.27; IR (film) ν_{max}/cm^{-1}: 3236, 2954, 2913, 1614, 1415, 1454, 1240, 1176, 825; ^{1}H NMR (400 MHz, $CDCl_3$) 7.05 – 6.99 (m, 2H), 6.79 – 6.72 (m, 2H), 4.69 (d, *J* = 1.5 Hz, 1H), 2.36 (td, *J* = 11.6, 3.5 Hz, 1H), 1.84 – 1.70 (m, 3H), 1.53 – 1.33 (m, 3H), 1.11 (dd, *J* = 13.8, 11.0 Hz, 2H), 1.06 – 0.97 (m, 1H), 0.89 (dd, *J* = 6.5, 1.3 Hz, 3H), 0.80 (dd, *J* = 7.0, 1.4 Hz, 3H), 0.67 (dd, *J* = 7.0, 1.4 Hz, 3H); ^{13}C NMR (101 MHz, $CDCl_3$) 153.4, 139.1, 128.6, 115.2, 47.7, 47.2, 45.7, 35.4, 33.4, 27.4, 24.7, 22.6, 21.6, 15.4; HRMS (EI$^+$) mass calculated for $[M]^+$ $C_{16}H_{24}O$ requires m/z 232.1827, found m/z 232.1824.

4-((1*R*,2*S*,5*R*)-2-Isopropyl-5-methylcyclohexyl)-2-methylphenol (**9p**)

The starting boronic ester (0.16 mmol) was reacted according to General Procedure A (23 mg; 58%, >20:1 d.r.) to afford the title compound as white wax/oil.

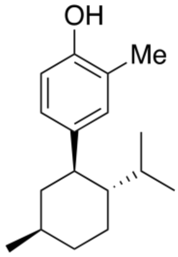

$[\alpha]_D^{23}$ = -37 (c 0.6, $CHCl_3$); R_f (15% EtOAc/pet. ether): 0.25; IR (film) ν_{max}/cm^{-1}: 3410, 2970, 2902, 1770, 1457, 1395, 1253, 1061, 896; ^{1}H NMR (400 MHz, $CDCl_3$) 6.92 (s, 1H), 6.88 (d, *J* = 8.3 Hz, 1H), 6.71 (d, *J* = 8.3 Hz, 1H), 4.65 (s, 1H), 2.38 – 2.31 (m, 1H), 2.25 (s, 3H), 1.85 – 1.71 (m, 3H), 1.54 – 1.29 (m, 3H), 1.20 – 0.97 (m, 3H), 0.90 (d, *J* = 6.4 Hz, 3H), 0.82 (d, *J* = 7.0 Hz, 3H), 0.69 (d, *J* = 6.9 Hz, 3H); ^{13}C NMR (101 MHz, $CDCl_3$) 151.7, 139.1, 130.0, 126.0, 123.5, 114.8, 47.6, 47.2, 45.8, 35.4, 33.4, 27.4, 24.7, 22.7, 21.7, 16.0, 15.4; HRMS (EI$^+$) mass calculated for $[M]^+$ $C_{17}H_{26}O$ requires m/z 246.1984, found m/z 246.1977.

4-((5*R*,6*S*)-3-*O*-(*tert*-Butyldimethylsilyl)cholestr-6-yl)phenol (**9q**)

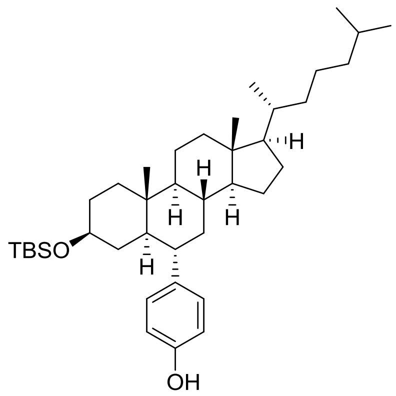

The starting boronic ester (0.16 mmol) was reacted according to General Procedure A (55 mg; 62%, >20:1 dr) to afford the title compound as colourless viscous oil.

$[\alpha]_D^{23}$ = +68.75 (c 0.8, $CHCl_3$); R_f (5% EtOAc/pet. ether): 0.30; IR (film) ν_{max}/cm^{-1}: 3353, 2929, 2866, 1514, 1251, 1081, 832, 775; ^{1}H NMR (500 MHz, $CDCl_3$) 6.96 (d, *J* = 7.9 Hz, 2H), 6.74 (d, *J* = 8.0 Hz, 2H), 4.79 (s, 1H), 3.38 (tt, *J* = 10.8, 4.6 Hz, 1H), 2.36 (td, *J* = 11.9, 3.7 Hz, 1H), 2.00 (dt, *J* = 12.6, 3.4 Hz, 1H), 1.84 – 1.62 (m, 5H), 1.58 – 0.93 (m, 24H), 0.93 – 0.82 (m, 11H), 0.77 (s, 9H), 0.67 (s, 3H), -0.11 (d, *J* = 8.2 Hz, 6H); ^{13}C NMR (126 MHz, $CDCl_3$) 153.5, 138.3, 130.7, 115.0, 72.6, 56.3, 56.2, 54.3, 49.8, 43.6, 42.5, 41.6, 40.0, 39.4, 37.5, 36.1, 36.0, 35.7, 35.3, 34.8, 31.7, 28.2,

27.9, 25.9, 24.1, 23.8, 22.8, 22.5, 21.3, 18.6, 18.2, 13.2, 12.0, -4.6, -4.8; HRMS (ESI$^+$) mass calculated for $[M+Na]^+$ $C_{39}H_{66}NaO_2Si$ requires m/z 617.4724, found m/z 617.4726.

(*R*)-2-Methyl-4-(6-methylhept-5-en-2-yl)phenol (9r)

The starting boronic ester (0.16 mmol) was reacted according to General Procedure A (57%, 100% es), to afford the title compound as pale yellow oil.

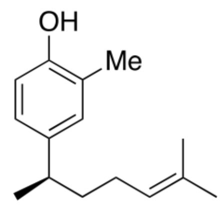

$[\alpha]_D^{23}$ = -40 (c 0.6, CH_2Cl_2); R_f (15% EtOAc/pet. ether): 0.30; IR (film) ν_{max}/cm^{-1}: 3410, 2962, 2923, 1507, 1264, 1118, 733; ^{1}H NMR (400 MHz, $CDCl_3$) 6.92 (d, *J* = 2.2 Hz, 1H), 6.88 (dd, *J* = 8.1, 2.2 Hz, 1H), 6.69 (d, *J* = 8.1 Hz, 1H), 5.12 – 5.06 (m, 1H), 4.52 (s, 1H), 2.59 (sx, *J* = 7.0 Hz, 1H), 2.23 (s, 3H), 1.87 (qi, *J* = 7.0 Hz, 2H), 1.67 (s, 3H), 1.59 – 1.51 (m, 5H), 1.19 (dd, *J* = 7.0, 0.9 Hz, 3H); ^{13}C NMR (101 MHz, $CDCl_3$) 151.8, 140.1, 131.4, 129.7, 125.5, 124.7, 123.4, 114.8, 38.7, 29.6, 26.3, 25.8, 22.7, 17.8, 16.0; HRMS (EI$^+$) mass calculated for $[M]^+$ $C_{15}H_{22}O$ requires m/z 218.1671, found m/z 218.1668. The er was determined by SFC [chiralpak IA, 5% of hexane/isopropanol 50/50, 125 bar, 2.0 mL/min, 40 °C, t (minor) = 20.2 min, t (major) = 20.9 min] to be 97:3 (100% es).

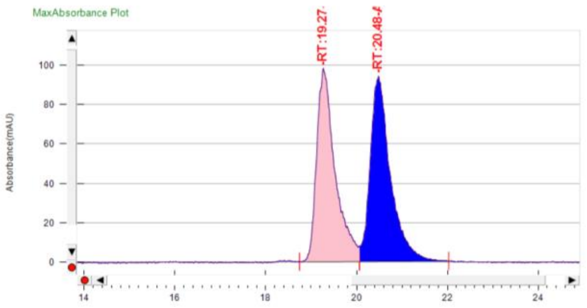

Peak Information

Peak No	% Area	Area	Ret. Time	Height	Cap. Factor
1	48.9864	2762.1247	19.27 min	98.0617	19265.4833
2	51.0136	2876.4303	20.48 min	93.9517	20482.4167

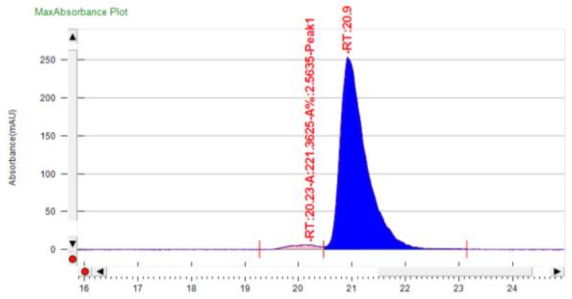

Peak Information

Peak No	% Area	Area	Ret. Time	Height	Cap. Factor
1	2.5635	221.3625	20.23 min	5.8986	20229.0333
2	97.4365	8413.6857	20.92 min	253.0587	20915.8333

(*S*)-2-(1-Phenylethyl)phenol (14a)

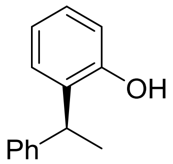

The starting boronic ester (0.20 mmol) was reacted according to General Procedure C to afford the title compound (21 mg; 53%) as colourless liquid. The spectral data matched that previously reported in the literature.[15]

$[\alpha]_D^{23}$ = -21.8 (c 1.1, $CHCl_3$); R_f (5% EtOAc/pet. ether): 0.2; IR (film) ν_{max}/cm^{-1}: 3639, 3465, 2984, 2940, 1737, 1446, 1372, 1236, 1045, 917; ^{1}H NMR (400 MHz, $CDCl_3$) 7.34 – 7.17 (m, 6H), 7.13 (td, *J* = 7.8, 1.5 Hz, 1H), 6.94 (td, *J* = 7.8, 1.5 Hz, 1H), 6.76 (dd, *J* = 7.8, 1.5 Hz, 1H), 4.62 (s, 1H), 4.37 (q, *J* = 7.2 Hz, 1H), 1.63 (d, *J* = 7.2 Hz, 3H). The er was determined by HPLC [Chiralpak IB with Guard, hexane/isopropanol 95/5, Flow: 1.0 mL/min, t (major) = 8.24 min, t (minor) = 8.75 min] to be 97:3 (100% es).

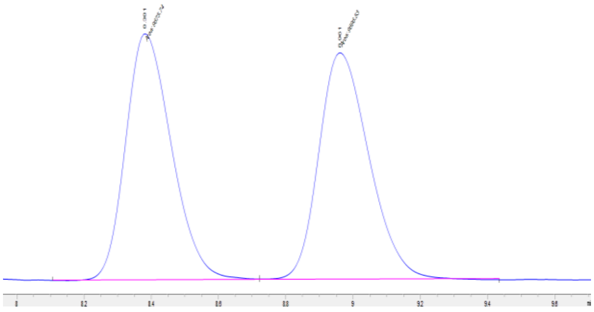

#	Time	Type	Area	Height	Width	Area%	Symmetry
1	8.381	MF	9675.7	1014.7	0.1589	49.946	0.716
2	8.961	FM	9696.5	933.6	0.1731	50.054	0.721

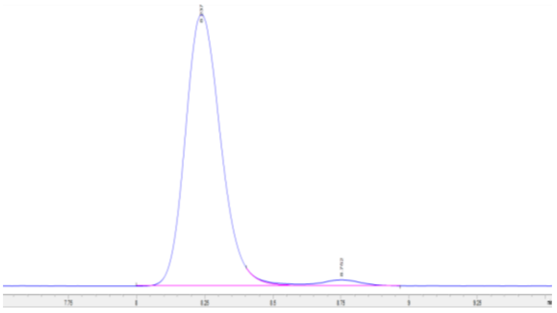

#	Time	Type	Area	Height	Width	Area%	Symmetry
1	8.237	VV R	4852.6	533.6	0.1306	96.962	0.821
2	8.752	VB E	152	12.1	0.1516	3.038	1.57

(*S*)-5-Methoxy-2-(1-phenylethyl)phenol (14b)

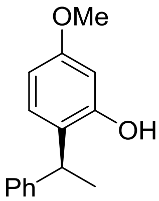

The starting boronic ester (0.20 mmol) was reacted according to General Procedure C to afford the title compound (37 mg; 82%) as colourless liquid. The spectral data matched that previously reported in the literature.[15]

R_f (10% EtOAc/pet. ether): 0.5; $[\alpha]_D^{23}$ = -18.4 (c 0.8, $CHCl_3$); IR (film) ν_{max}/cm^{-1}: 3388, 2965, 1615, 1518, 1203, 699; ^{1}H NMR (500 MHz, $CDCl_3$) 7.34 – 7.27 (m, 2H), 7.27 – 7.17 (m, 3H), 7.14 (d, J = 8.5 Hz, 1H), 6.51 (dd, J = 8.5, 2.5 Hz, 1H), 6.36 (dd, J = 2.6, 0.6 Hz, 1H), 4.64 (s, 1H), 4.26 (q, J = 7.2 Hz, 1H), 3.76 (s, 3H), 1.60 (d, J = 7.2 Hz, 3H). The er was determined by HPLC [Chiralpak IA without Guard, hexane/isopropanol 95/5, Flow: 1.0 mL/min, t (major) = 8.24 min, t (minor) = 8.75 min] to be 97:3 (100% es).

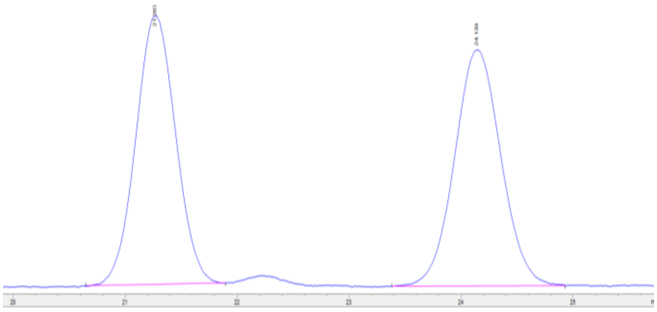

#	Time	Type	Area	Height	Width	Area%	Symmetry
1	21.263	VV R	2142.2	86.4	0.2929	49.389	0.891
2	24.139	VV R	2195.2	75.9	0.3403	50.611	0.922

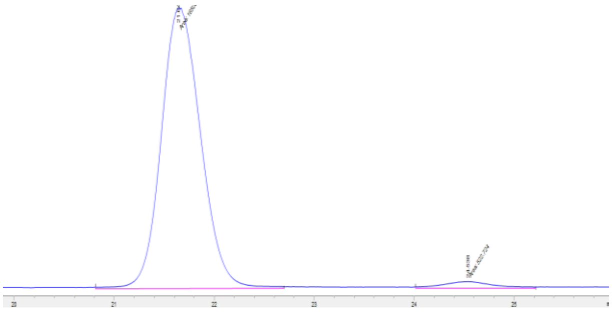

#	Time	Type	Area	Height	Width	Area%	Symmetry
1	21.643	MM	16503	602.9	0.4562	96.930	0.804
2	24.538	MM	522.7	14.2	0.6118	3.070	0.959

(*S*)-5-Fluoro-2-(1-phenylethyl)phenol (14c)

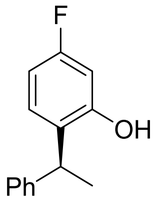

The starting boronic ester (0.20 mmol) was reacted according to General Procedure C to afford the title compound (18 mg; 41%) as colourless liquid. The spectral data matched that previously reported in the literature.[15]

R_f (5% EtOAc/pet. ether): 0.4; $[\alpha]_D^{23}$ = +6.3 (c 0.8, $CHCl_3$); IR (film) ν_{max}/cm^{-1}: 3532, 2930, 1453, 1250, 1066, 751; ^{1}H NMR (500 MHz, $CDCl_3$) 7.33 – 7.26 (m, 2H), 7.26 – 7.11 (m, 4H), 6.64 (td, *J* = 8.5, 2.5 Hz, 1H), 6.54 – 6.45 (m, 1H), 4.79 (s, 1H), 4.27 (q, *J* = 7.1 Hz, 1H), 1.60 (d, *J* = 7.2 Hz, 3H). The er

was determined by HPLC [Chiralpak IB with Guard Column, hexane/isopropanol 95/5, Flow: 1.0 mL/min, t (major) = 8.04 min, t (minor) = 9.14 min] to be 97:3 (100% es).

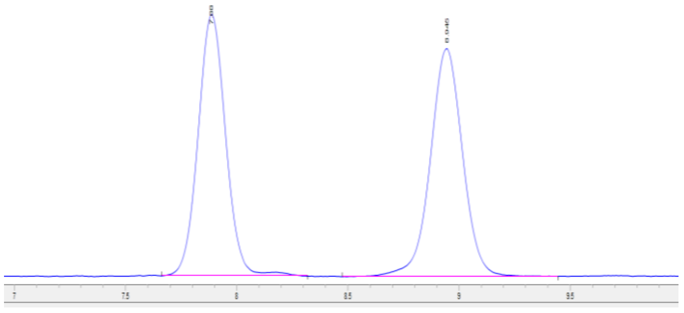

#	Time	Type	Area	Height	Width	Area%	Symmetry
1	7.886	BV R	1812.5	208.8	0.1334	49.569	0.909
2	8.945	VV R	1844.1	182	0.1552	50.431	1.016

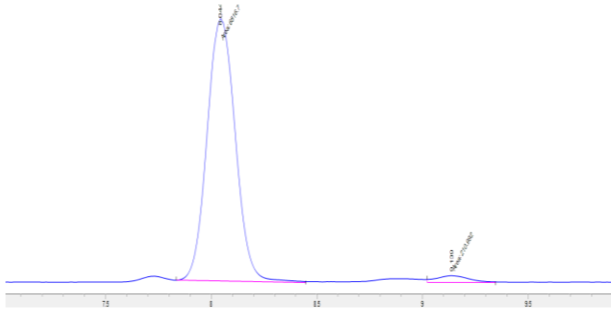

#	Time	Type	Area	Height	Width	Area%	Symmetry
1	8.044	MM	6816.7	727.3	0.1562	96.998	0.88
2	9.139	MM	211	19.2	0.183	3.002	0.848

(*R*)-4-Chloro-2-(1-phenylethyl)phenol (14d)

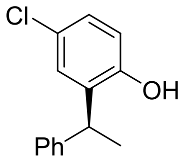

The starting boronic ester (0.20 mmol) was reacted according to General Procedure C to afford the title compound (24 mg; 52%) as colourless liquid. The spectral data matched that previously reported in the literature.[15]

R_f (5% EtOAc/pet. ether): 0.4; $[\alpha]_D^{23}$ = -21.7 (c 1.2, $CHCl_3$); IR (film) ν_{max}/cm^{-1}: 3344, 2970, 1493, 1415, 1268, 1078, 814, 699; ^1H NMR (500 MHz, $CDCl_3$) 7.34 (dd, *J* = 8.3, 6.9 Hz, 2H), 7.28 – 7.21 (m, 4H), 7.10 (dd, *J* = 8.5, 2.6 Hz, 1H), 6.71 (d, *J* = 8.5 Hz, 1H), 4.67 (s, 1H), 4.34 (q, *J* = 7.2 Hz, 1H), 1.63 (d, *J* = 7.2 Hz, 3H). The er was determined by HPLC [Chiralpak IA without Guard, hexane/isopropanol 95/5, Flow: 1.0 mL/min, t (minor) = 9.25 min, t (major) = 10.06 min] to be 97:3 (100% es).

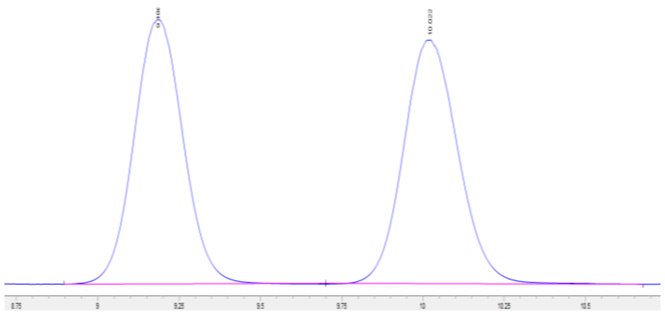

#	Time	Type	Area	Height	Width	Area%	Symmetry
1	9.186	BV R	7445.5	707.1	0.1318	49.375	0.983
2	10.022	BV R	7634.1	651	0.1403	50.625	0.933

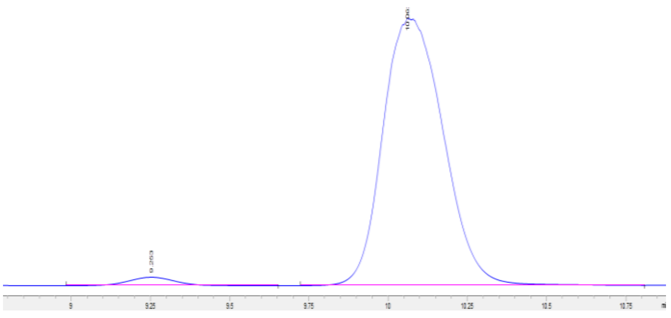

#	Time	Type	Area	Height	Width	Area%	Symmetry
1	9.253	VV R	420.3	40.6	0.1297	2.392	0.945
2	10.062	BB	17149.6	1291.7	0.1569	97.608	0.692

2-Cyclohexylphenol (15a)

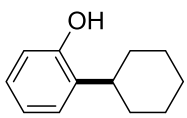

The starting boronic ester (0.20 mmol) was reacted according to General Procedure C (21 mg; 60%) to afford the title compound as gummy liquid. The spectral data matched with the commercially available sample.

R_f (10% EtOAc/pet. ether): 0.30; ^{1}H NMR (400 MHz, $CDCl_3$) δ 7.07 (dd, J = 8.4, 2 Hz, 1H), 6.83 (m, 1H), 6.78 (dd, J = 8.2, 2 Hz, 1H), 6.72 (m, 1H), 4.64 (bs, 1H), 2.50-2.43 (m, 1H), 1.91 – 1.35 (m, 10H).

(*R*)-2-(4-(4-Methoxyphenyl)butan-2-yl)phenol (**15b**)

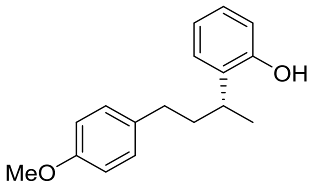

The starting boronic ester (0.20 mmol) was reacted according to General Procedure C to afford the title compound (36 mg; 68%) as colourless liquid.

R_f (5% EtOAc/pet. ether): 0.3; $[\alpha]_D^{23}$ = +6.2 (c 0.6, $CHCl_3$); IR (film) ν_{max}/cm^{-1}: 3675, 3418, 2970, 1511, 1242, 1066, 826, 752; ^{1}H NMR (500 MHz, $CDCl_3$) 7.19 (d, J = 7.6 Hz, 1H), 7.07 (m, 3H), 6.93 (t, J = 7.5 Hz, 1H), 6.82 (d, J = 8.5 Hz, 2H), 6.76 (d, J = 7.9 Hz, 1H), 4.64 (bs, 1H), 3.78 (s, 3H), 3.05 (sx, J = 6.9 Hz, 1H), 2.52 (m, 2H), 2.04 – 1.80 (m, 2H), 1.27 (d, J = 6.9 Hz, 3H); ^{13}C NMR (126 MHz, $CDCl_3$) 157.6, 152.9, 134.5, 132.8, 129.2, 127.1, 126.7, 121.0, 115.4, 113.7, 55.2, 38.9, 32.9, 31.7, 20.9; HRMS (ESI$^+$) mass calculated for $[M+Na]^+$ $C_{17}H_{20}NaO_2$ requires m/z 279.1356, found m/z 279.1360. The er was determined by HPLC [Chiralpak IA, hexane/isopropanol 95/5, Flow: 1.0 mL/min, t (major) = 16.30 min, t (minor) = 20.67 min] to be 95.5:4.5 (100% es).

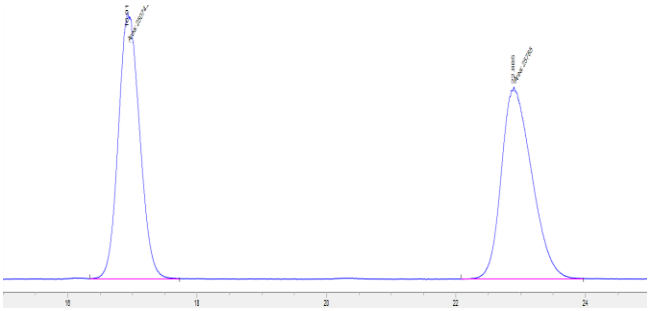

#	Time	Type	Area	Height	Width	Area%	Symmetry
1	16.915	MM	25074.2	1091.2	0.383	49.301	0.71
2	22.885	MM	25785	786.8	0.5462	50.699	0.695

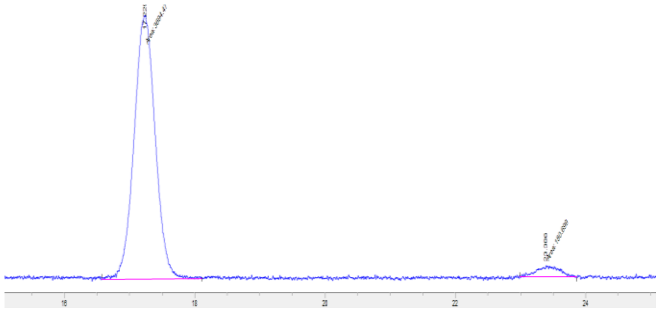

#	Time	Type	Area	Height	Width	Area%	Symmetry
1	17.229	MM	3694.5	162.1	0.3798	95.337	1.011
2	23.386	MM	180.7	7.4	0.4092	4.663	0.766

2-(3-Methyl-1-phenylpentan-3-yl)phenol (**15c**)

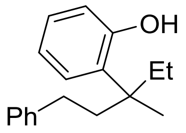

The starting racemic Bneop boronic ester **1i** (0.20 mmol) was reacted according to General Procedure C to afford the title compound (11 mg, 22%) as colourless liquid.

R_f (5% EtOAc/pet. ether): 0.6; ^{1}H NMR (500 MHz, $CDCl_3$) 7.26 – 7.18 (m, 3H), 7.17 – 7.05 (m, 4H), 6.95 – 6.85 (m, 1H), 6.70 – 6.60 (m, 1H), 4.74 (s, 1H), 2.57 – 2.37 (m, 2H), 2.27 – 2.11 (m, 2H), 1.80 (td, J = 12.5, 11.9, 4.0 Hz, 1H), 1.60 (dd, J = 13.7, 7.4 Hz, 1H), 1.41 (s, 3H), 0.69 (t, J = 7.5 Hz, 3H); ^{13}C NMR (126 MHz, $CDCl_3$) 154.0, 143.6, 132.3, 129.6, 128.3, 128.1, 127.1, 125.3, 120.5, 116.2, 42.2, 41.9, 32.7, 31.5, 24.1, 9.0; HRMS (ESI$^+$) mass calculated for $[M+Na]^+$ $C_{18}H_{22}NaO$ requires m/z 277.1563, found m/z 277.1558.

2-((3r,5r,7r)-Adamantan-1-yl)phenol (15d)

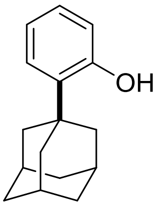

The starting boronic ester (0.20 mmol) was reacted according to General Procedure C to afford the title compound (26 mg; 58%) as colourless liquid. The spectral data matched that previously reported in the literature.[16]

R_f (5% EtOAc/pet. ether): 0.4; IR (film) ν_{max}/cm^{-1}: 3511, 2908, 2852, 1445, 1349, 1250, 756; ^{1}H NMR (400 MHz, $CDCl_3$) 7.22 (dd, *J* = 7.8, 1.6 Hz, 1H), 7.07 (td, *J* = 7.6, 1.7 Hz, 1H), 6.91 (td, *J* = 7.6, 1.3 Hz, 1H), 6.65 (dd, *J* = 7.8, 1.3 Hz, 1H), 4.71 (s, 1H), 2.17 – 2.04 (m, 9H), 1.79 (m, 6H).

(*R*)-2-(1-Phenylhept-6-en-3-yl)phenol (15e)

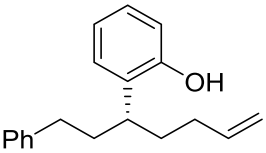

The starting boronic ester (0.20 mmol) was reacted according to General Procedure C to afford the title compound (35 mg; 65%) as colourless liquid.

$[\alpha]_D^{23}$ = +4.60 (c 1.2, $CHCl_3$); R_f (5% EtOAc/pet. ether): 0.4; IR (film) ν_{max}/cm^{-1}: 3675, 2987, 2901, 1393, 1056, 892; ^{1}H NMR (500 MHz, $CDCl_3$) 7.28 – 7.20 (m, 2H), 7.19 – 7.06 (m, 5H), 6.93 (t, *J* = 7.5 Hz, 1H), 6.77 (d, *J* = 8.0 Hz, 1H), 5.78 (ddt, *J* = 17.0, 10.2, 6.7 Hz, 1H), 5.02 – 4.82 (m, 2H), 4.57 (s, 1H), 2.95 (sx, *J* = 7.4 Hz, 1H), 2.57 – 2.40 (m, 2H), 1.96 (dq, *J* = 14.4, 7.7, 7.2 Hz, 4H), 1.76 (dq, *J* = 15.3, 7.4 Hz, 2H); ^{13}C NMR (126 MHz, $CDCl_3$) 153.6, 142.5, 138.9, 130.6, 128.3, 128.2, 127.8, 126.8, 125.6, 121.1, 115.5, 114.5, 37.4, 37.1, 35.0, 33.7, 31.5; HRMS (ESI$^+$) mass calculated for $[M]^+$ $C_{19}H_{22}NaO$ requires m/z 289.1563, found m/z 289.1566. The er was determined by HPLC [Chiralpak IA, hexane/isopropanol 95/5, Flow: 1.0 mL/min, t (major) = 9.61 min, t (minor) = 10.61 min] to be 96:4 (99% es).

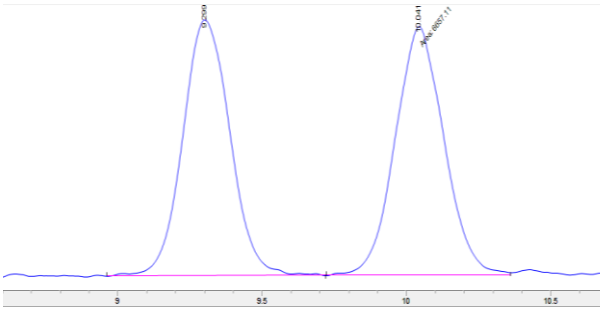

#	Time	Type	Area	Height	Width	Area%	Symmetry
1	9.299	VV R	6660.6	580.3	0.1773	50.013	0.866
2	10.041	MF	6657.1	564.2	0.1967	49.987	0.941

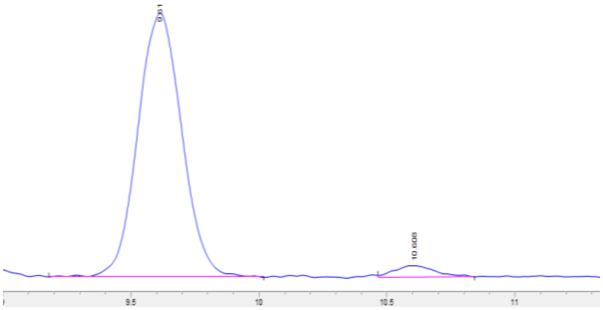

#	Time	Type	Area	Height	Width	Area%	Symmetry
1	9.613	VV R	10093.1	842.3	0.1855	95.930	0.997
2	10.608	VV R	428.2	37.6	0.1549	4.070	0.938

(*R*)-2-(7-Azido-1-phenylheptan-3-yl)phenol (15f)

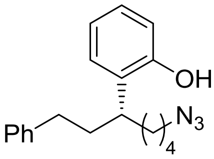

The starting boronic ester (0.20 mmol) was reacted according to General Procedure C to afford the title compound (29 mg; 47%) as colourless liquid.

R_f (5% EtOAc/pet. ether): 0.4; $[\alpha]_D^{23}$ = +6.9 (c 1.2, $CHCl_3$); IR (film) ν_{max}/cm^{-1}: 3660, 3401, 2921, 1243, 1067, 832, 740; ^{1}H NMR (500 MHz, $CDCl_3$) 7.30 – 7.25 (m, 2H), 7.23 – 7.05 (m, 5H), 6.96 (t, *J* = 7.4 Hz, 1H), 6.78 (d, *J* = 7.9 Hz, 1H), 3.21 (tq, *J* = 9.8, 5.2 Hz, 2H), 3.01 (p, *J* =

7.4 Hz, 1H), 2.59 – 2.44 (m, 2H), 1.99 (q, J = 7.6 Hz, 2H), 1.78 – 1.65 (m, 2H), 1.63 – 1.48 (m, 2H), 1.42 – 1.16 (m, 2H); ^{13}C NMR (126 MHz, $CDCl_3$) 153.6, 142.5, 130.8, 128.3, 128.2, 127.9, 126.8, 125.6, 121.1, 115.5, 51.3, 37.4, 35.4, 33.7, 29.7, 28.8, 24.5; HRMS (ESI$^+$) mass calculated for $[M+Na]^+$ $C_{19}H_{23}N_3NaO$ requires m/z 332.1733, found m/z 332.1739. The er was determined by HPLC [Chiralpak IB, hexane/isopropanol 99/1, Flow: 1.0 mL/min, t (major) = 12.07 min, t (minor) = 12.65 min] to be 99:1 (100% es).

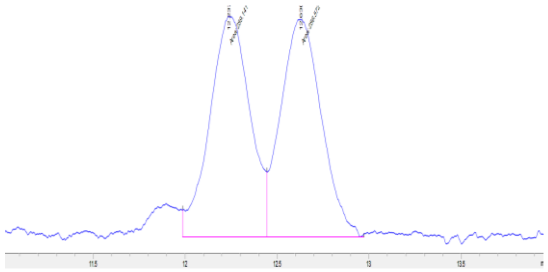

#	Time	Type	Area	Height	Width	Area%	Symmetry
1	12.237	MF	289.1	18.9	0.2553	50.223	0.898
2	12.63	FM	286.6	18.6	0.2568	49.777	0.932

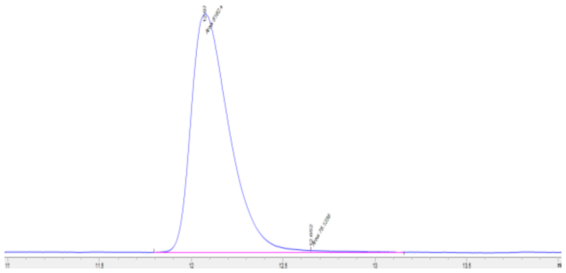

#	Time	Type	Area	Height	Width	Area%	Symmetry
1	12.071	MF	8162.4	563.5	0.2414	99.052	0.535
2	12.652	FM	78.1	5.2	0.2485	0.948	0

2-((1*R*,2*S*,5*R*)-2-Isopropyl-5-methylcyclohexyl)phenol (15g)

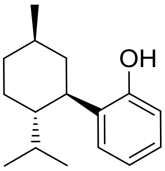

The starting boronic ester (0.20 mmol) was reacted according to General Procedure C to afford the title compound (23 mg; 50%) as colourless liquid.

R_f (5% EtOAc/pet. ether): 0.4; $[\alpha]_D^{23}$ = -13.6 (c 1.2, $CHCl_3$); IR (film) ν_{max}/cm^{-1}: 3407, 2902, 1458, 1392, 1253, 1061, 896; ^{1}H NMR (500 MHz, $CDCl_3$) 7.17 (d, J = 7.7 Hz, 1H), 7.06 (td, J = 7.6, 1.7 Hz, 1H), 6.93 (t, J = 7.5 Hz, 1H), 6.77 (d, J = 8.0 Hz, 1H), 4.71 (s, 1H), 2.94 (td, J = 12.4, 11.8, 7.4 Hz, 1H), 1.89 – 1.74 (m, 3H), 1.54 (d, J = 11.0 Hz, 3H), 1.27 – 1.15 (m, 1H), 1.12 – 0.99 (m, 2H), 0.92 (d, J = 6.5 Hz, 3H), 0.84 (d, J = 6.9 Hz, 3H), 0.72 (d, J = 6.8 Hz, 3H); ^{13}C NMR (126 MHz, $CDCl_3$) 152.7, 132.2, 127.4, 126.1, 121.0, 115.2, 46.8, 44.6, 38.2, 35.3, 33.2, 27.5, 24.7, 22.4, 21.6, 15.8; HRMS (ESI$^+$) mass calculated for $[M+Na]^+$ $C_{16}H_{24}NaO$ requires m/z 255.1719, found m/z 255.1723.

***tert*-Butyl 4-(2-hydroxyphenyl)piperidine-1-carboxylate (15h)**

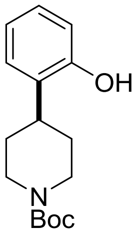

The starting boronic ester (0.20 mmol) was reacted according to General Procedure C to afford the title compound (20 mg, 43%) as colourless liquid.

R_f (30% EtOAc/pet. ether): 0.4; IR (film) ν_{max}/cm^{-1}: 3317, 2975, 2930, 1659, 1429, 1237, 1163, 752; ^{1}H NMR (500 MHz, $CDCl_3$) 7.11 (d, J = 7.6 Hz, 1H), 7.06 (t, J = 7.5 Hz, 1H), 6.88 (t, J = 7.5 Hz, 1H), 6.77 (d, J = 7.9 Hz, 1H), 5.85 (s, 1H), 4.23 (s, 2H), 3.05 (t, J = 12.1 Hz, 1H), 2.83 (s, 2H), 1.83 (m, 2H), 1.61 (m, 2H), 1.49 (s, 9H); ^{13}C NMR (126 MHz, $CDCl_3$) 155.0, 153.2, 131.8, 126.9, 126.8, 120.6, 115.2, 79.6, 44.8, 35.5, 31.6, 28.5; HRMS (ESI$^+$) mass calculated for $[M+Na]^+$ $C_{16}H_{23}NNaO_3$ requires m/z 300.1570, found m/z 300.1565.

6. Reversible Boronate Complex Formation with Benzotriazoles

With strong electron-withdrawing substituents on the aromatic ring of the aryl halide of the *ortho*-ArOBt substrates (e.g. CF_3-substituted example **S4** in the scheme below), the resultant anion **S5** from the lithium-halogen exchange is more stabilized than the boronate complex **S6**. Hence, a reversible boronate complex formation was observed. After 16 h, upon quenching the reaction with water, only a very low yield of the coupled product **S7** was obtained and the protonated phenoxybenzotriazole **S8** and boronic ester starting material **6** were isolated.

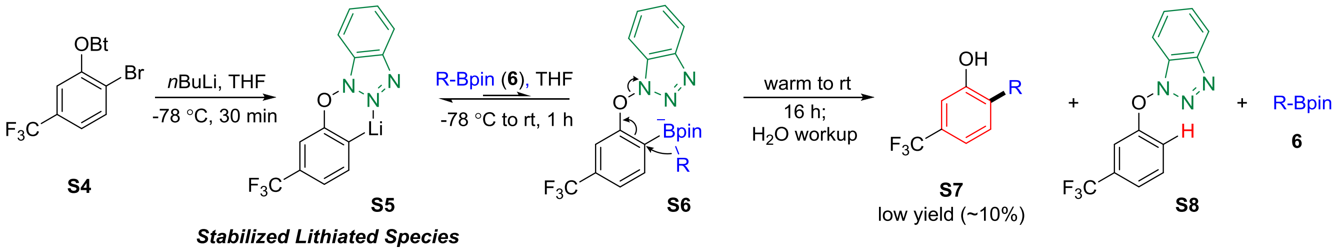

Similar low reactivity was observed with sterically hindered tertiary pinacol boronic esters such as **8g**. In this case, the reaction of aryl lithium **12** with boronic ester **8g** is, presumably, reversible due to destabilization of boronate complex **S9** as a result of the large steric hindrance. As a result, low yields of the coupled product **15c** were obtained and the protonated phenoxybenzotriazole **S9** and boronic ester starting material **8g** were isolated.

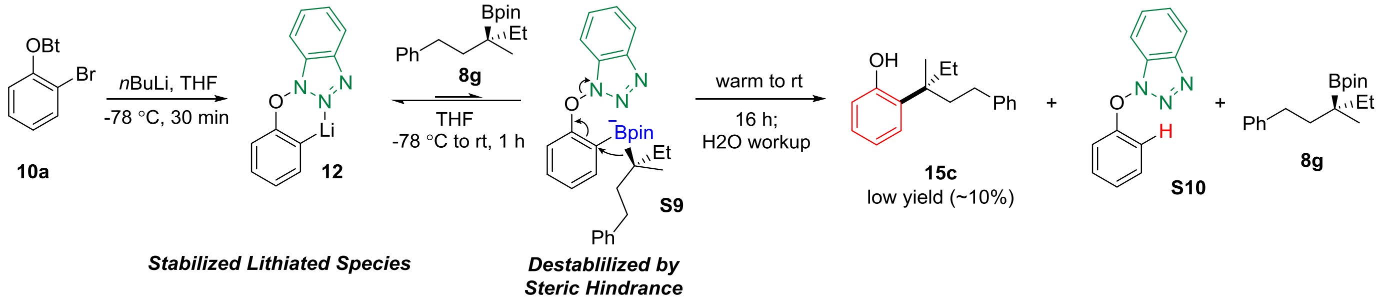

An improvement in the yield was generally observed when the lithiated species in used in excess, which is also indicative of a reversible boronate complex.

7. NMR Spectra (^{1}H, ^{13}C and ^{19}F)

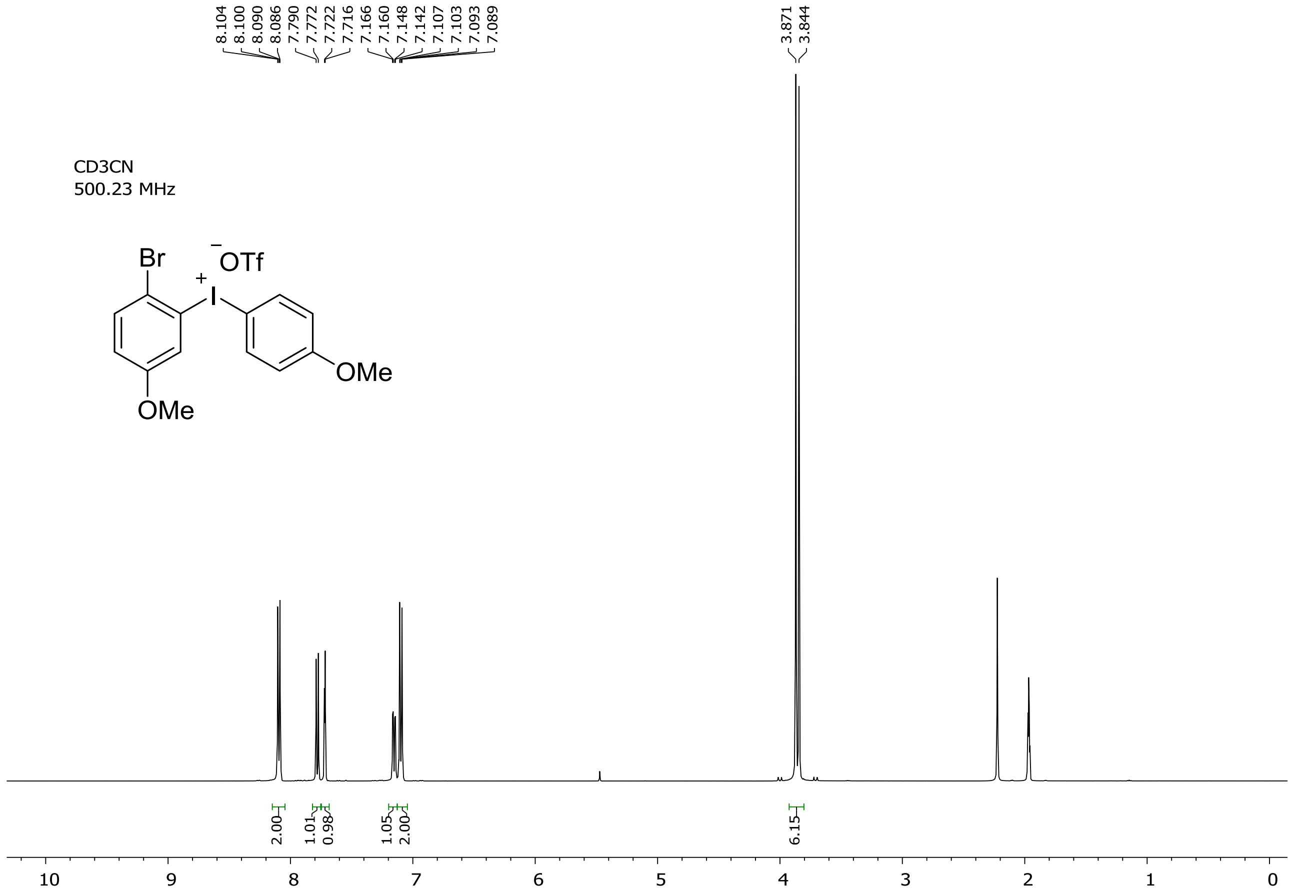

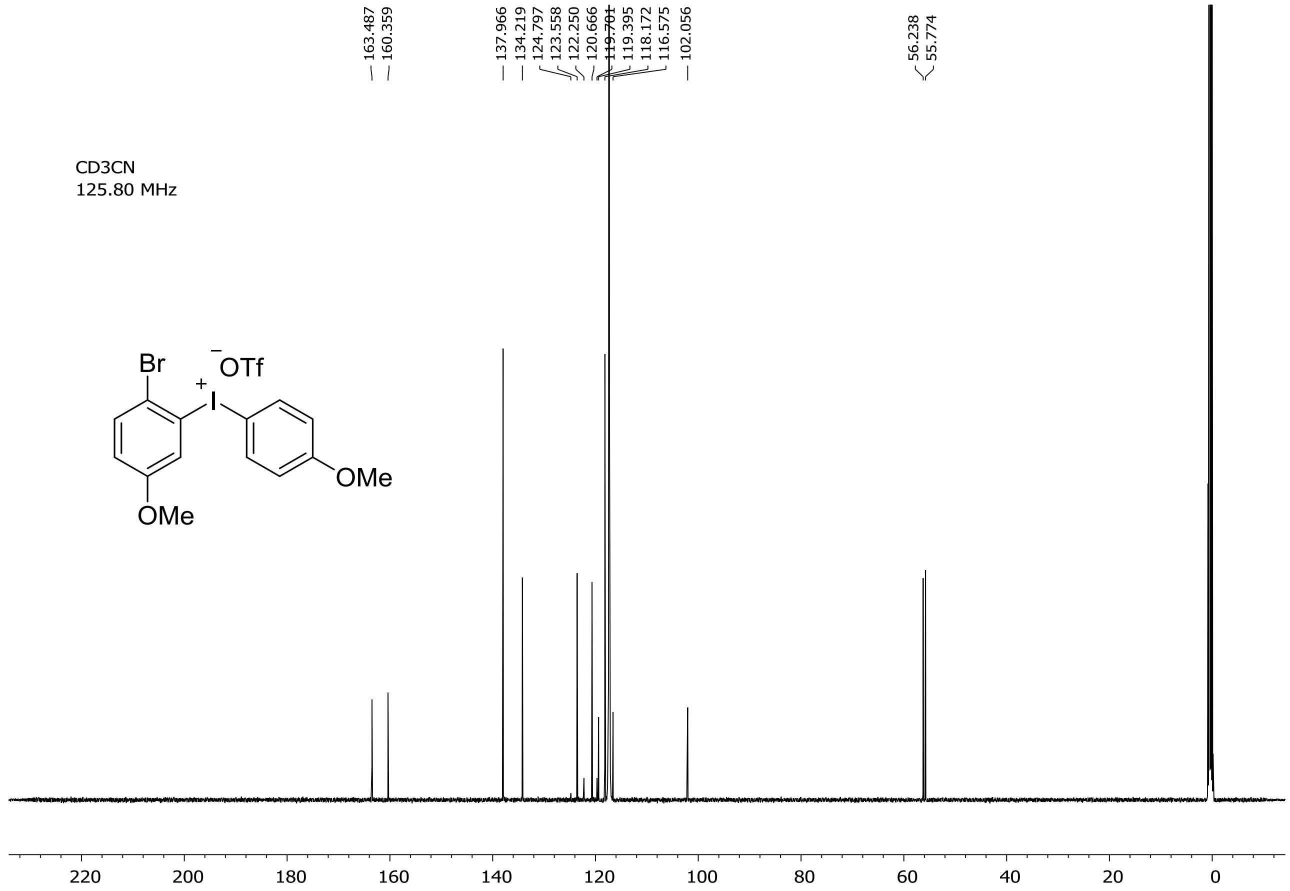

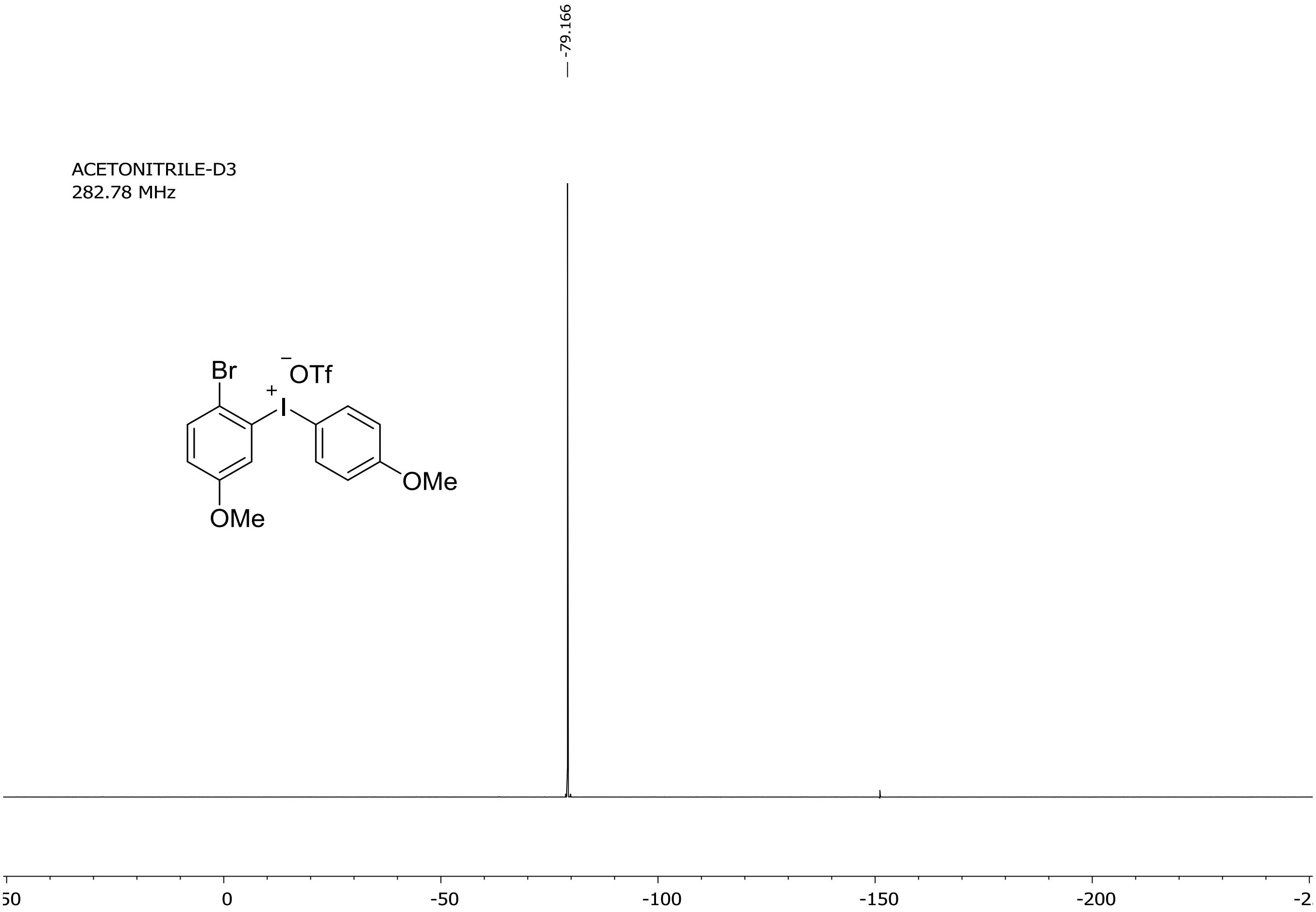
-79.166
ACETONITRILE-D3
282.78 MHz
Br
OTf
I
OMe
OMe
50
0
-50
-100
-150
-200

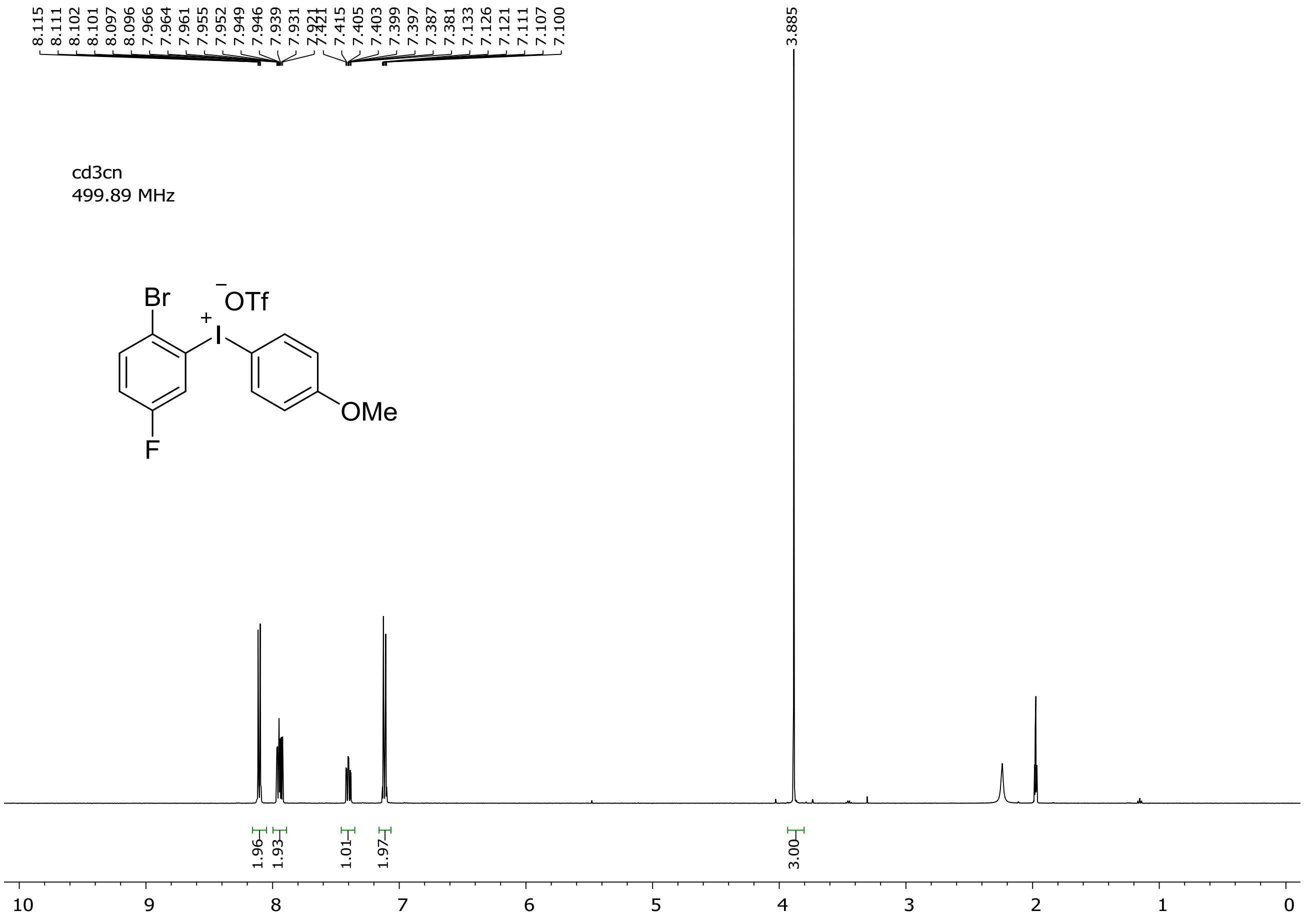
8.115
8.111
8.102
8.101
8.097
8.096
7.966
7.964
7.961
7.955
7.952
7.949
7.946
7.939
7.931
7.415
7.405
7.403
7.399
7.397
7.387
7.381
7.133
7.126
7.121
7.111
7.107
7.100
3.885
cd3cn
499.89 MHz
Br
OTf
I
OMe
F
1.96
1.93
1.01
1.97
3.00
10
9
8
7
6
5
4
3
2
1
0

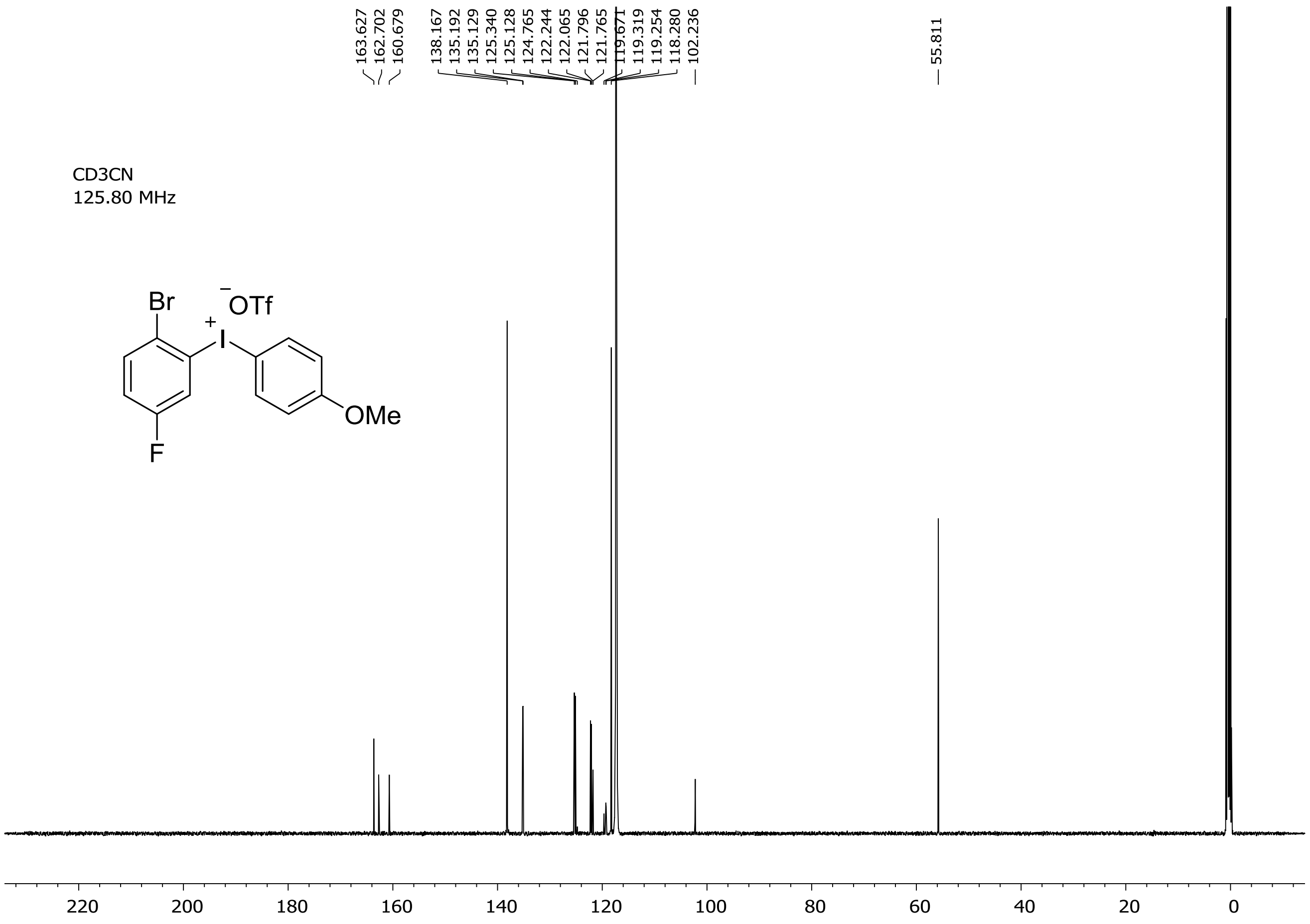
163.627
162.702
160.679
138.167
135.192
135.129
125.340
125.128
124.765
122.244
122.065
121.796
121.765
119.671
119.319
119.254
118.280
102.236
55.811
CD3CN
125.80 MHz
Br
OTf
I
OMe
F
220
200
180
160
140
120
100
80
60
40
20
0

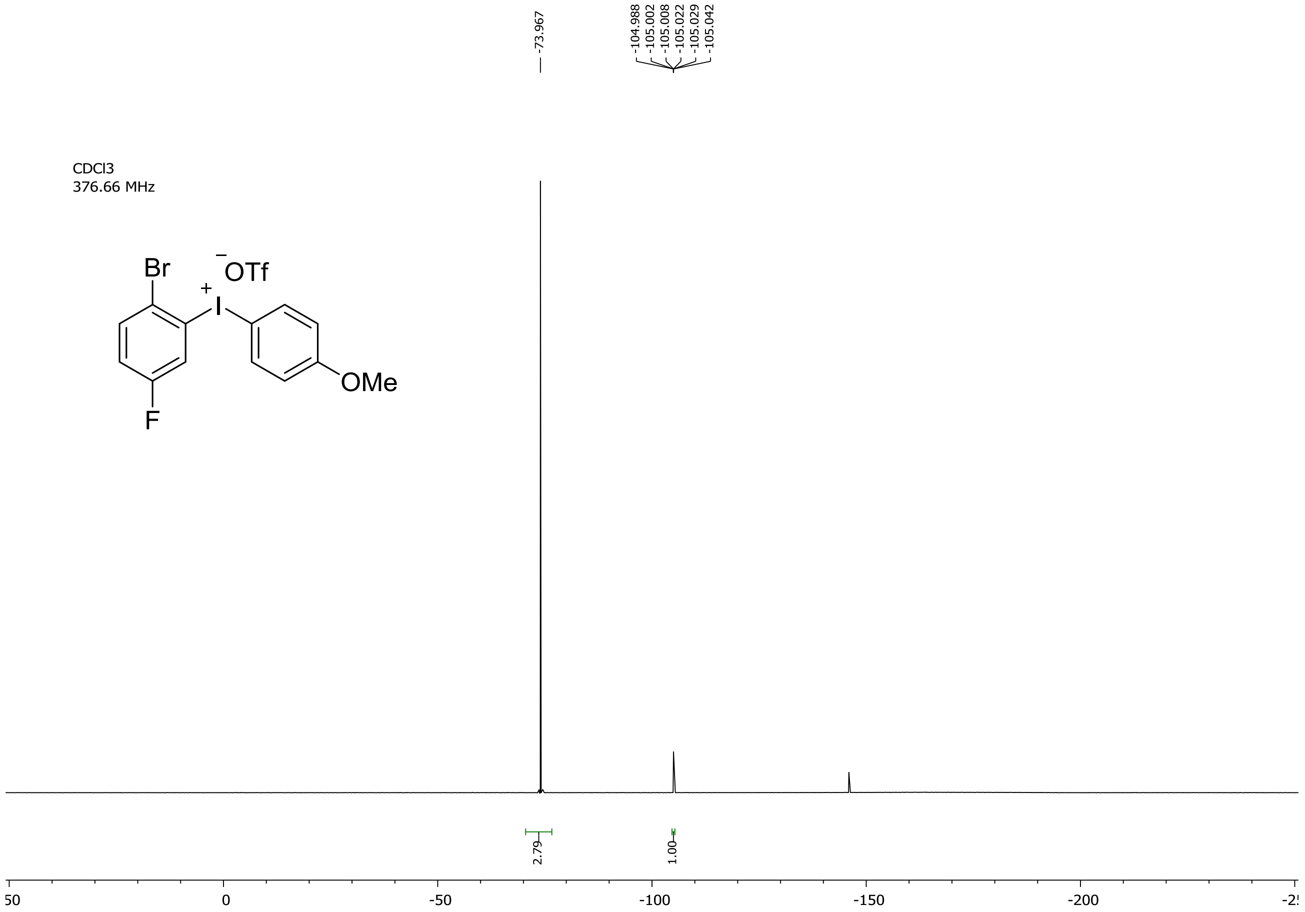
-73.967
-104.988
-105.002
-105.008
-105.022
-105.029
-105.042
CDCl3
376.66 MHz
Br
OTf
I
OMe
F
2.79
1.00
0
-50
-100
-150
-200

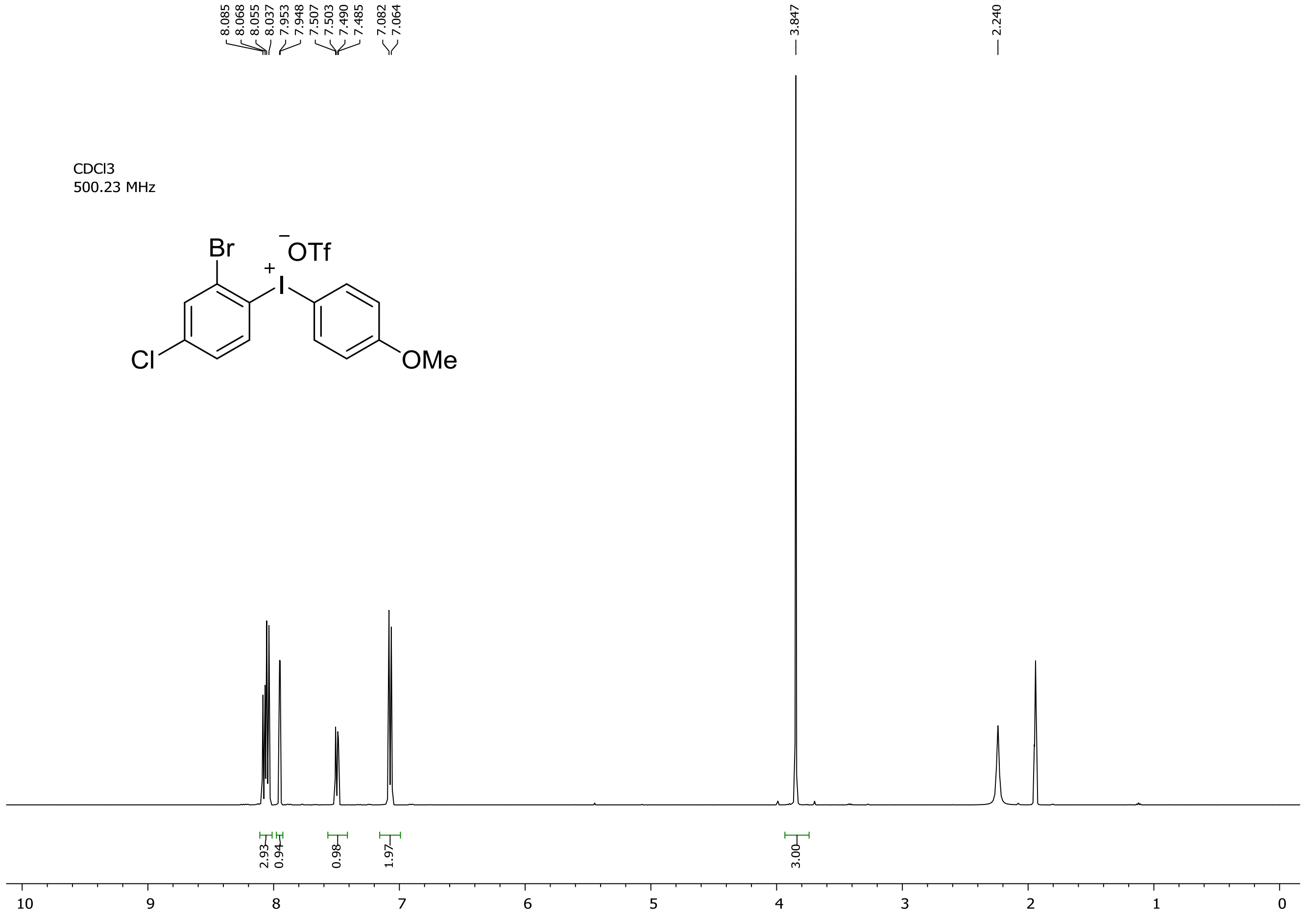
8.085
8.068
8.055
8.037
7.953
7.948
7.507
7.503
7.490
7.485
7.082
7.064
3.847
2.240
CDCl3
500.23 MHz
Br
OTf
I
Cl
OMe
2.93
0.94
0.98
1.97
3.00
10
9
8
7
6
5
4
3
2
1
0

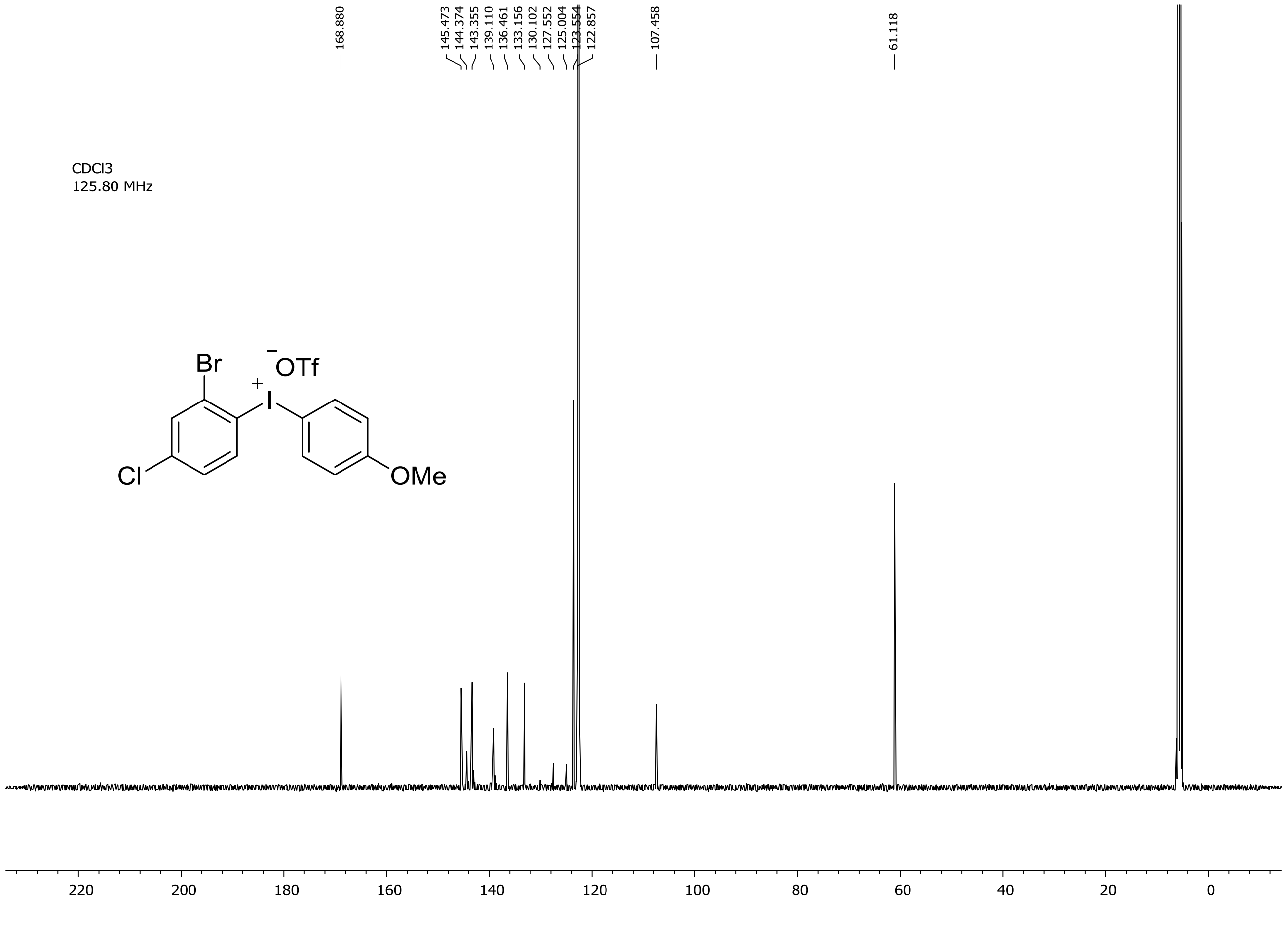
168.880
145.473
144.374
143.355
139.110
136.461
133.156
130.102
127.552
125.004
123.554
122.857
107.458
61.118
CDCl3
125.80 MHz
Br
OTf
I
Cl
OMe
220
200
180
160
140
120
100
80
60
40
20
0

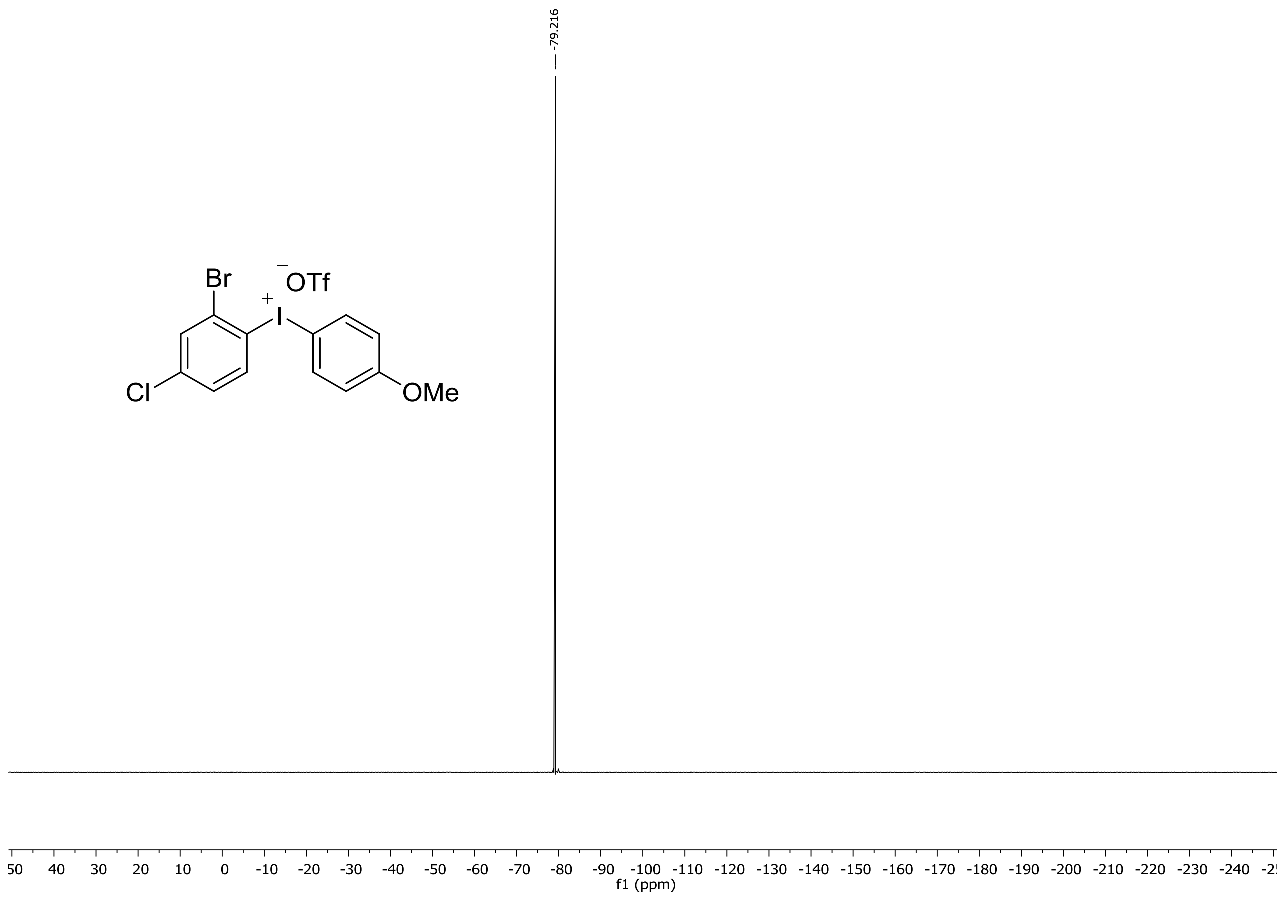
-79.216
Br
OTf
I
Cl
OMe
f1 (ppm)

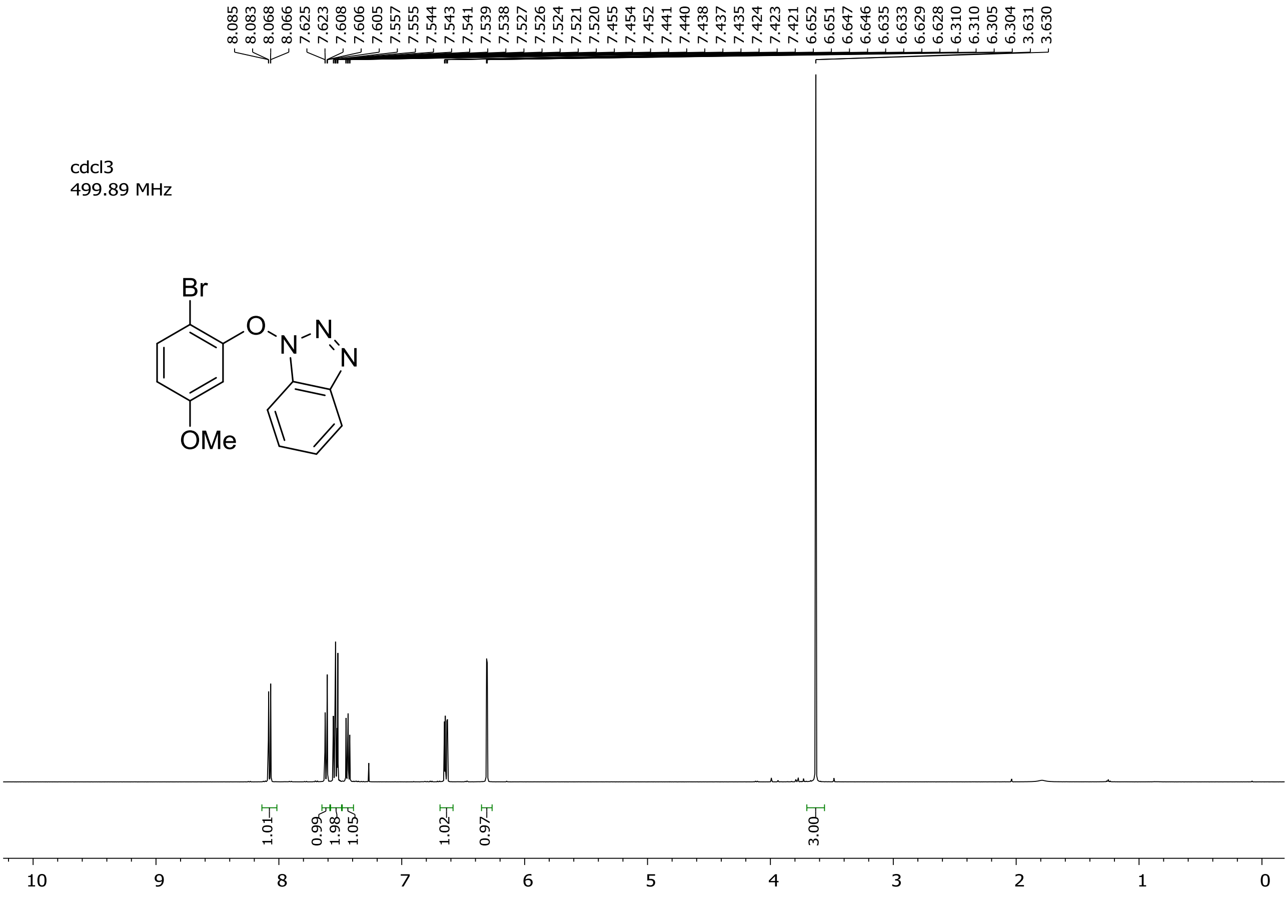
8.085
8.083
8.068
8.066
7.625
7.623
7.608
7.606
7.605
7.557
7.555
7.544
7.543
7.541
7.539
7.538
7.527
7.526
7.524
7.521
7.520
7.455
7.454
7.452
7.441
7.440
7.438
7.437
7.435
7.424
7.423
7.421
6.652
6.651
6.647
6.646
6.635
6.633
6.629
6.628
6.310
6.310
6.305
6.304
3.631
3.630
cdcl3
499.89 MHz
Br
O
N
N
N
OMe
1.01
0.99
1.98
1.05
1.02
0.97
3.00

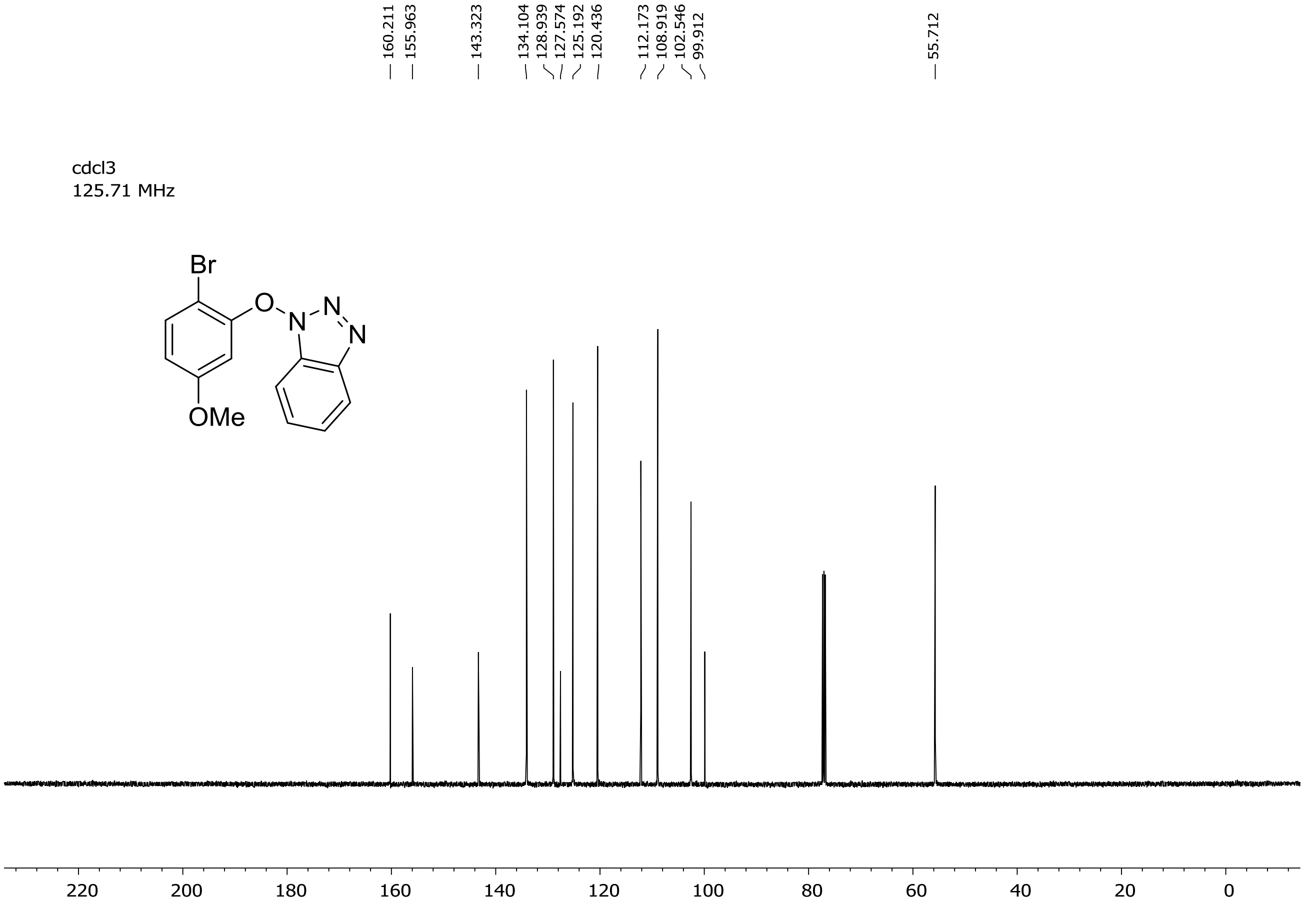
160.211
155.963
143.323
134.104
128.939
127.574
125.192
120.436
112.173
108.919
102.546
99.912
55.712
cdcl3
125.71 MHz
Br
O
N
N
N
OMe
220 200 180 160 140 120 100 80 60 40 20 0

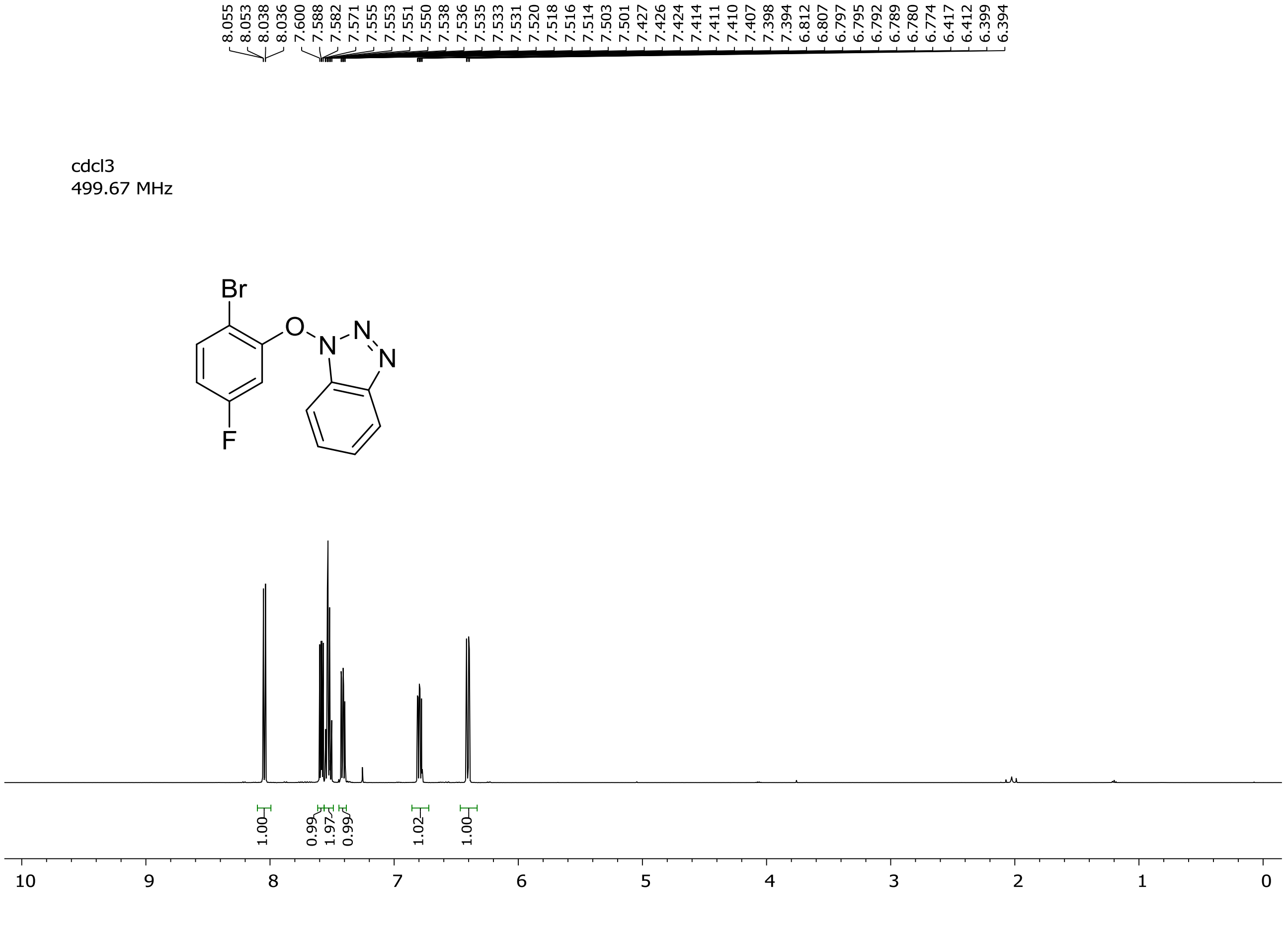
8.055
8.053
8.038
8.036
7.600
7.588
7.582
7.571
7.555
7.553
7.551
7.550
7.538
7.536
7.535
7.533
7.531
7.520
7.518
7.516
7.514
7.503
7.501
7.427
7.426
7.424
7.414
7.411
7.410
7.407
7.398
7.394
6.812
6.807
6.797
6.795
6.792
6.789
6.780
6.774
6.417
6.412
6.399
6.394
cdcl3
499.67 MHz
Br
O
N
N
N
F
1.00
0.99
1.97
0.99
1.02
1.00
10 9 8 7 6 5 4 3 2 1 0

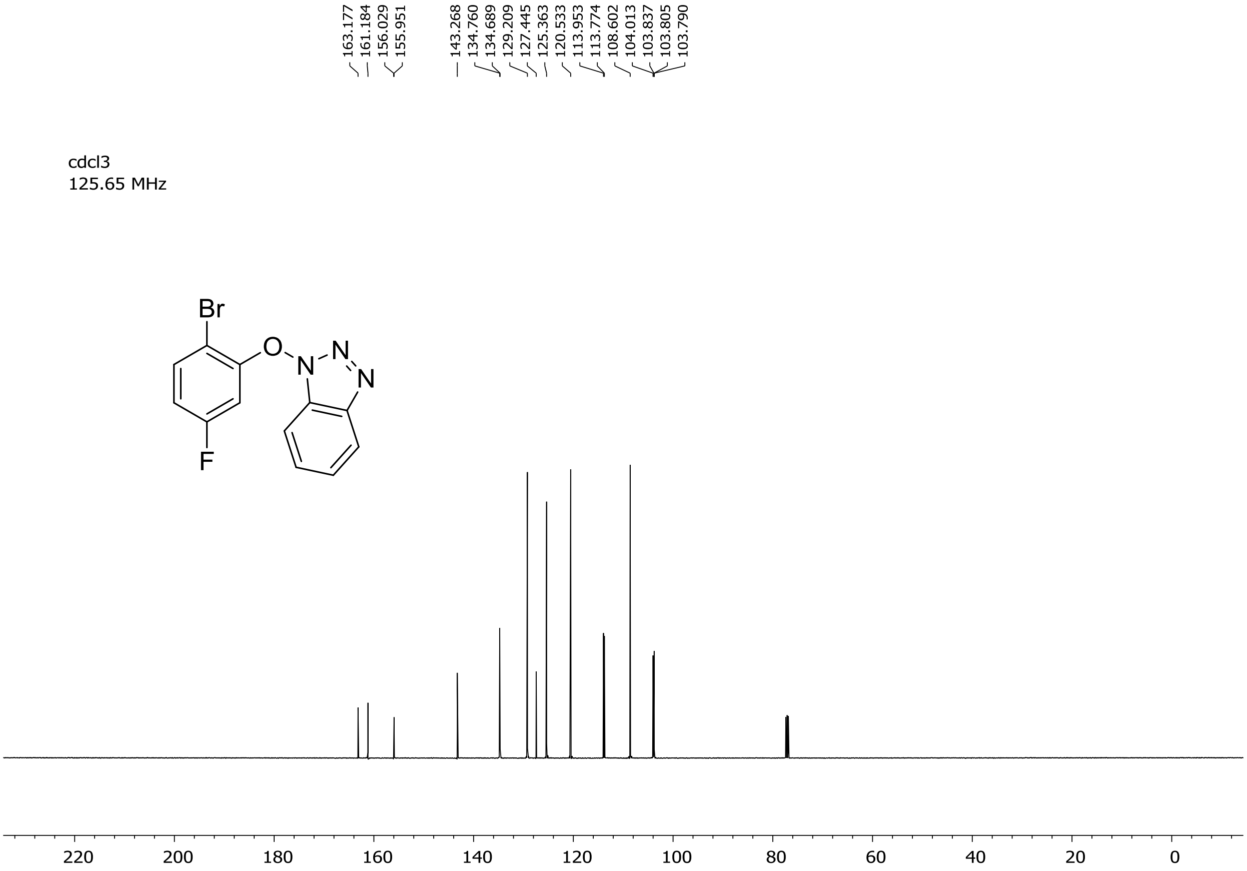
163.177
161.184
156.029
155.951
143.268
134.760
134.689
129.209
127.445
125.363
120.533
113.953
113.774
108.602
104.013
103.837
103.805
103.790
cdcl3
125.65 MHz
Br
O
N
N
N
F
220
200
180
160
140
120
100
80
60
40
20
0

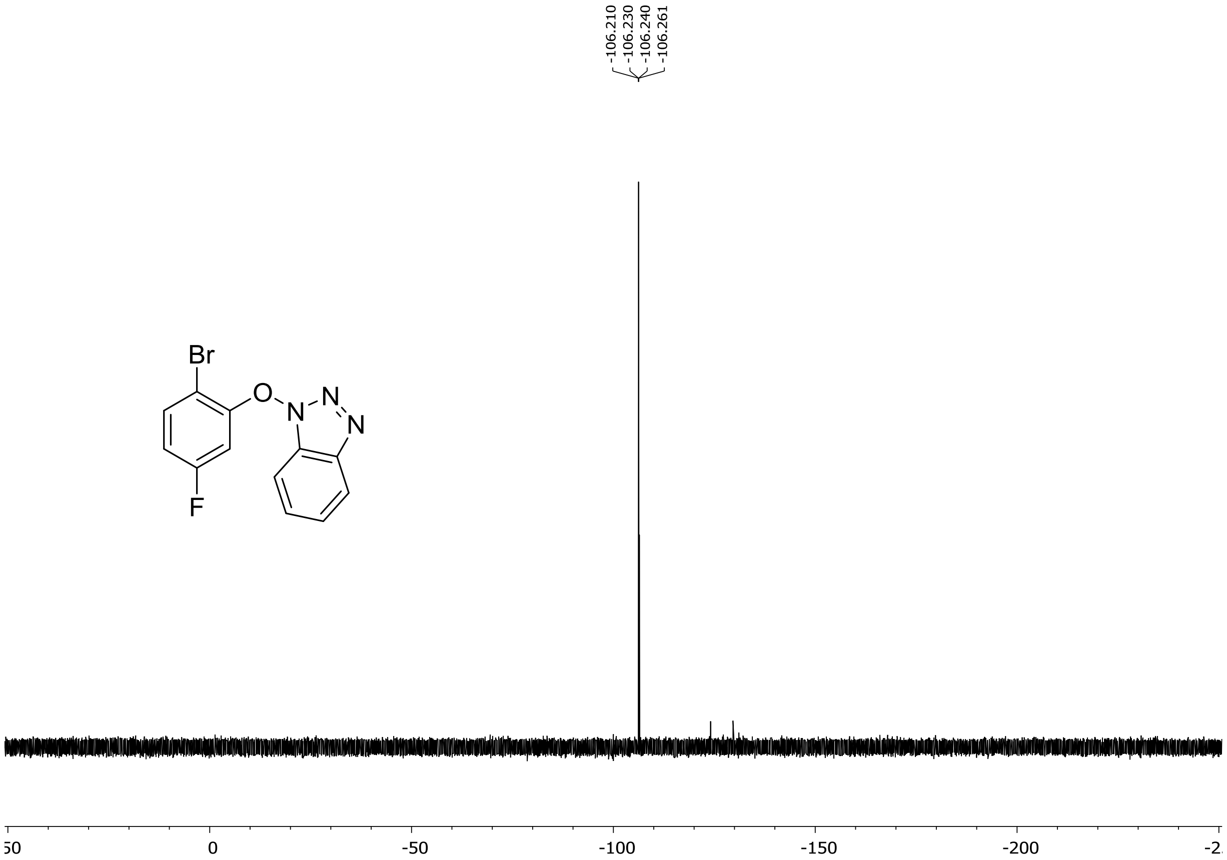
-106.210
-106.230
-106.240
-106.261
Br
O
N
N
N
F
0
-50
-100
-150
-200

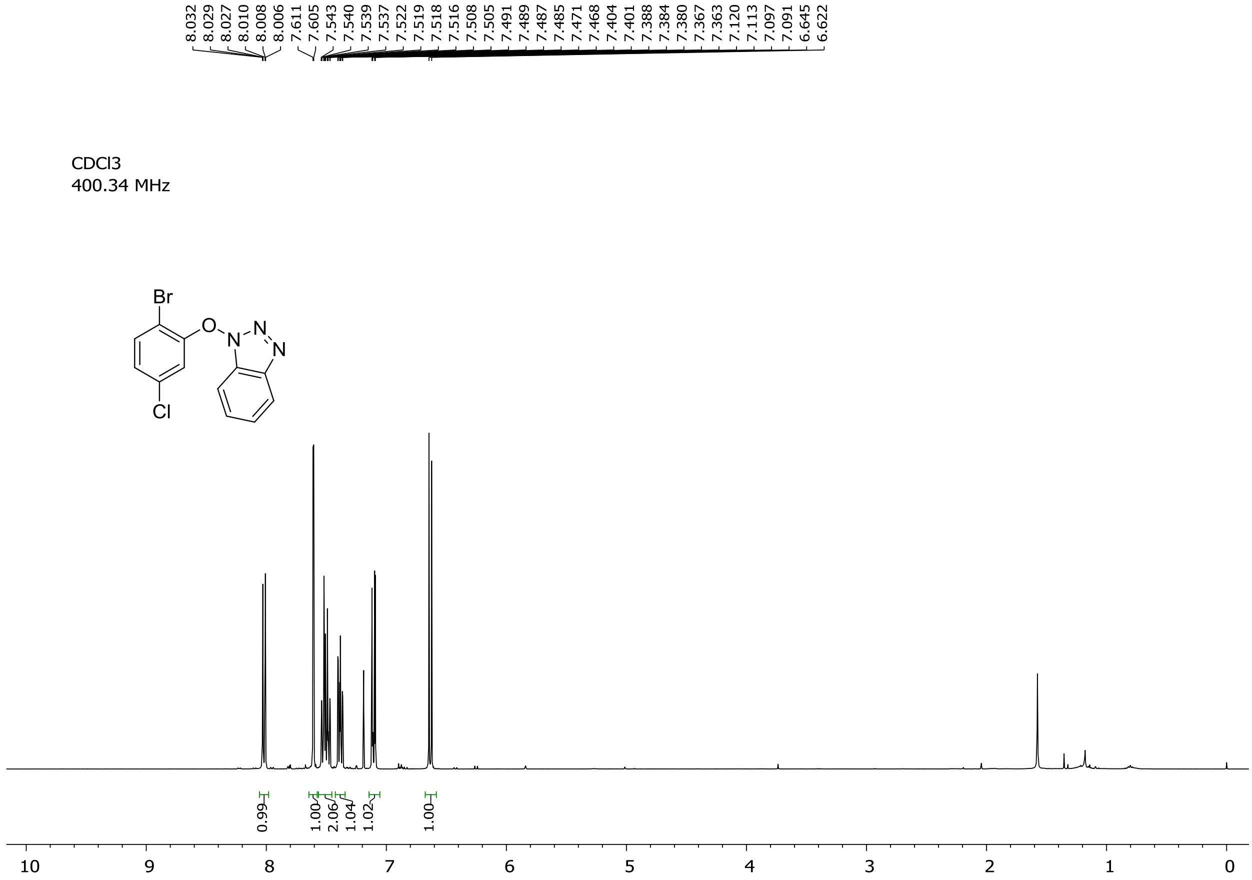
8.032 8.029 8.027 8.010 8.008 8.006 7.611 7.605 7.543 7.540 7.539 7.537 7.522 7.519 7.518 7.516 7.508 7.505 7.491 7.489 7.487 7.485 7.471 7.468 7.404 7.401 7.388 7.384 7.380 7.367 7.363 7.120 7.113 7.097 7.091 6.645 6.622
CDCl3
400.34 MHz
Br
O
N
N
N
Cl
0.99
1.00
2.06
1.04
1.02
1.00
10
9
8
7
6
5
4
3
2
1
0

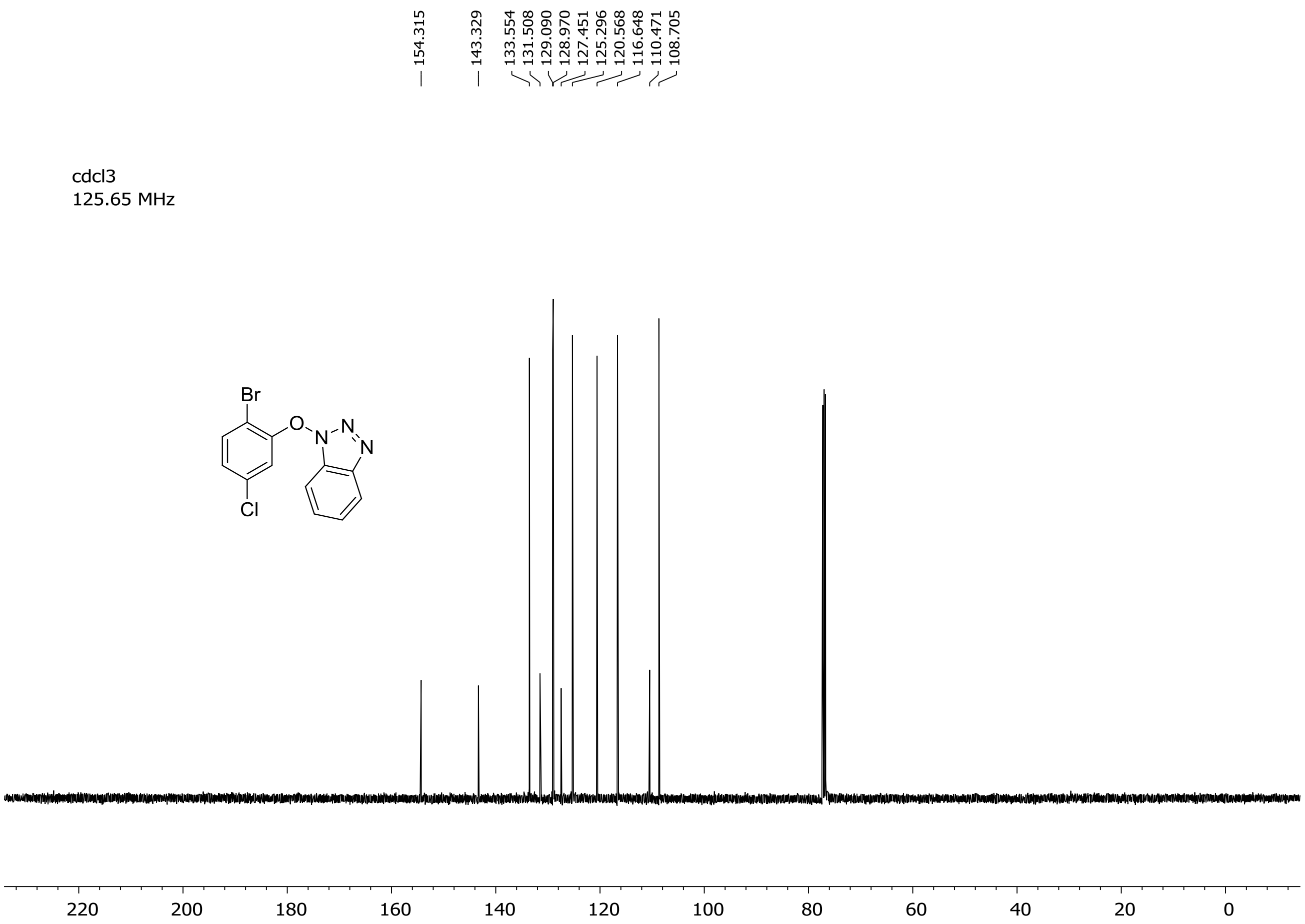
154.315
143.329
133.554 131.508 129.090 128.970 127.451 125.296 120.568 116.648 110.471 108.705
cdcl3
125.65 MHz
Br
O
N
N
N
Cl
220
200
180
160
140
120
100
80
60
40
20
0

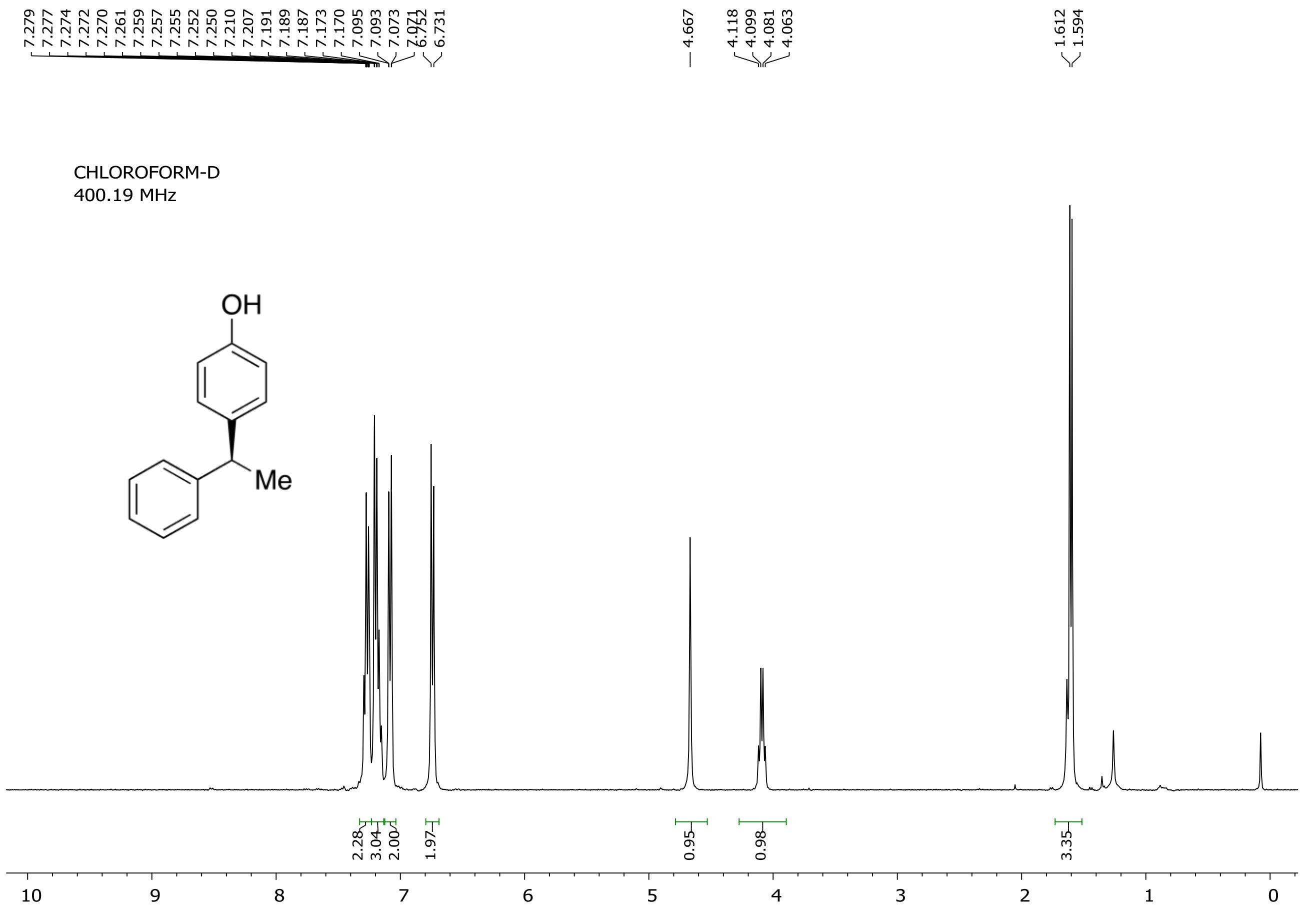

7.279
7.277
7.274
7.272
7.270
7.261
7.259
7.257
7.255
7.252
7.250
7.210
7.207
7.191
7.189
7.187
7.173
7.170
7.095
7.093
7.073
7.071
6.752
6.731
4.667
4.118
4.099
4.081
4.063
1.612
1.594
CHLOROFORM-D
400.19 MHz
OH
Me
2.28
3.04
2.00
1.97
0.95
0.98
3.35
10 9 8 7 6 5 4 3 2 1 0

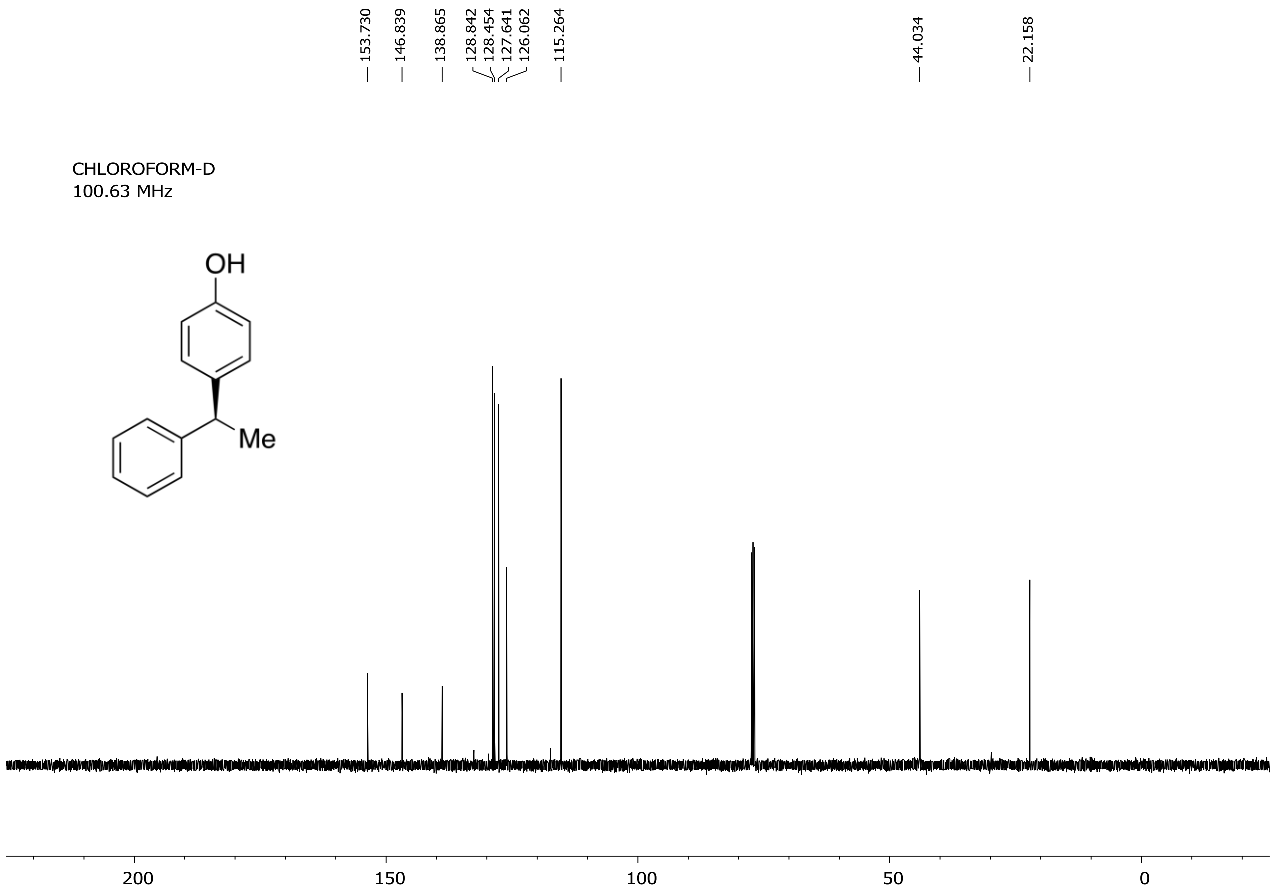

153.730
146.839
138.865
128.842
128.454
127.641
126.062
115.264
44.034
22.158
CHLOROFORM-D
100.63 MHz
OH
Me
200 150 100 50 0

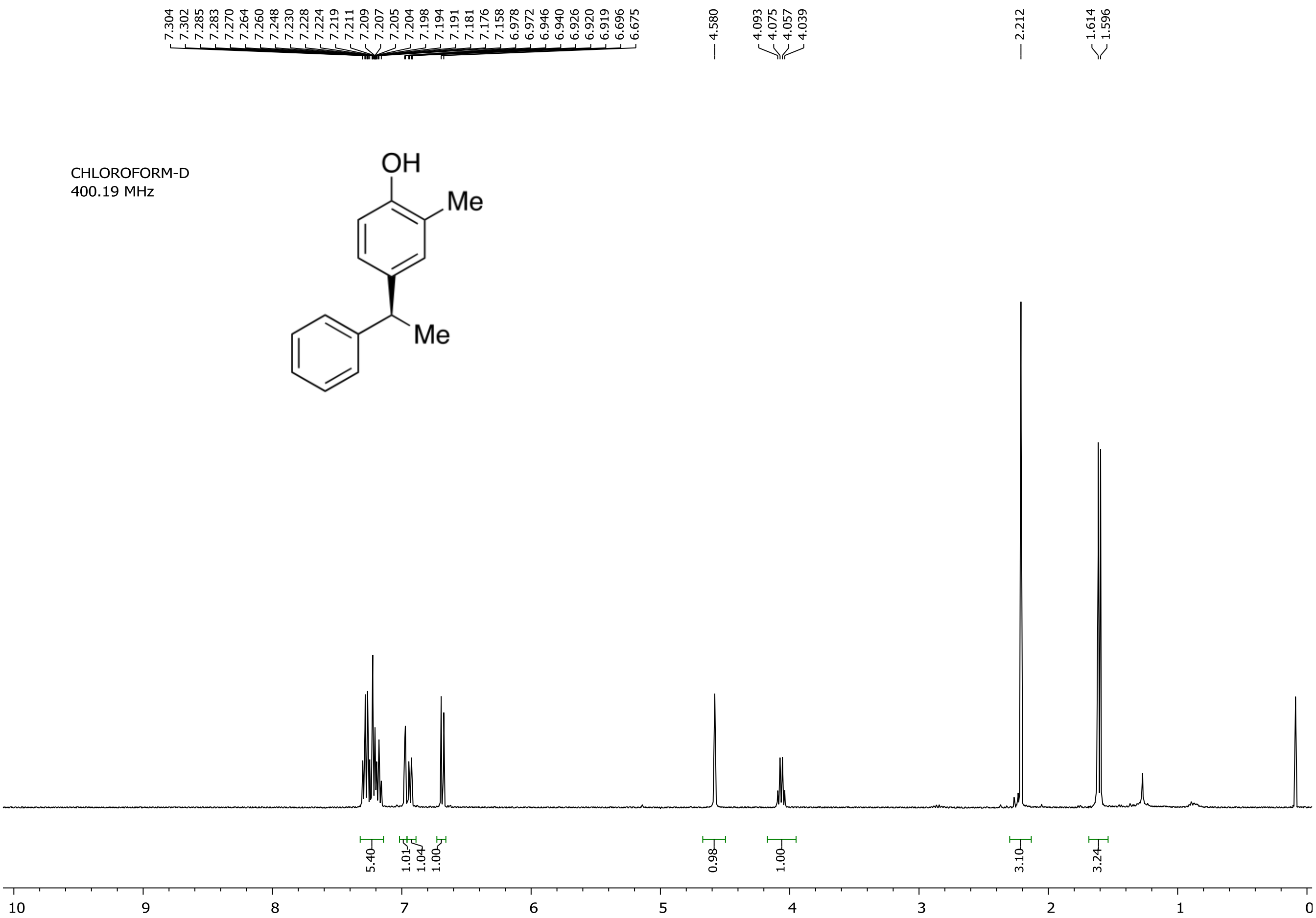
CHLOROFORM-D
400.19 MHz
OH
Me
Me

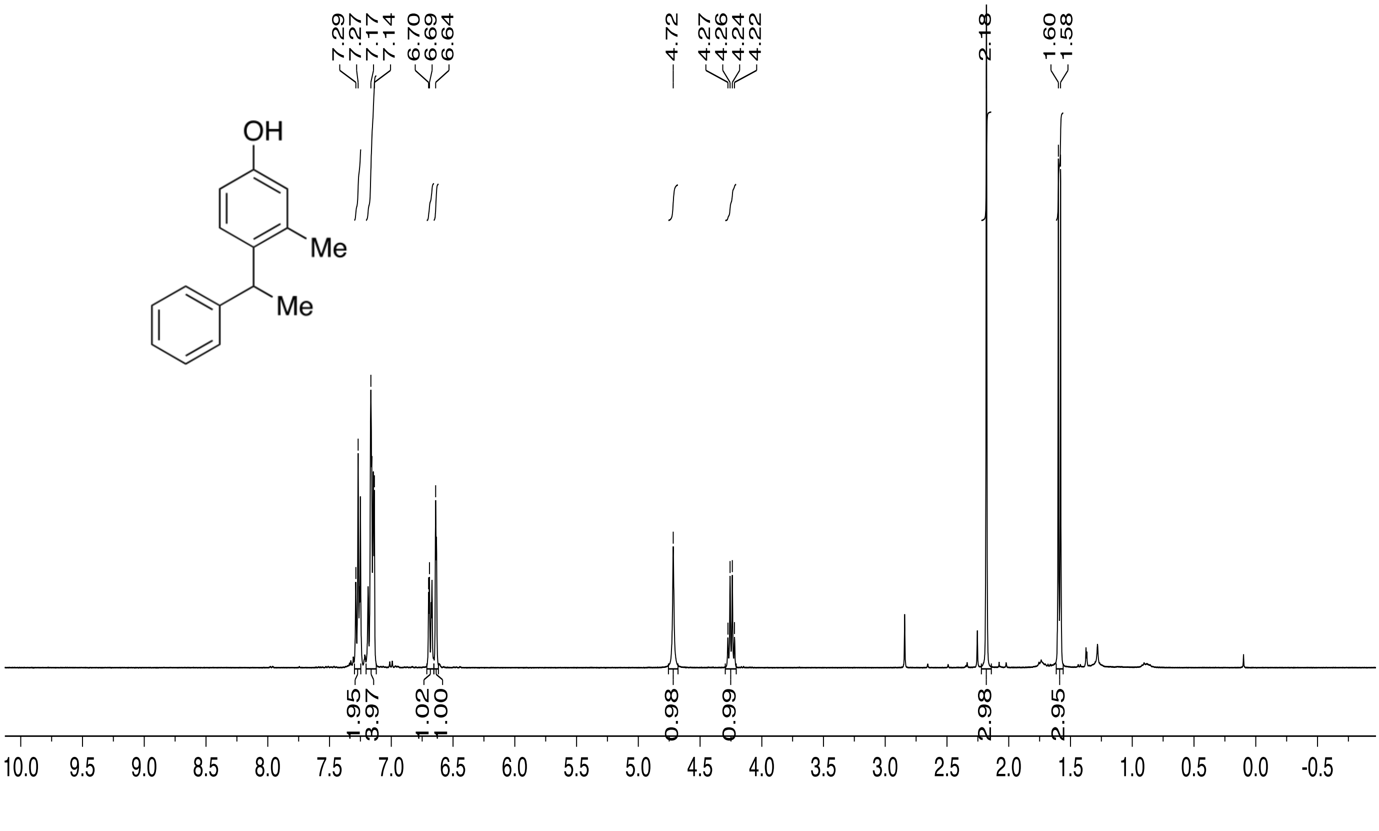
OH
Me
Me

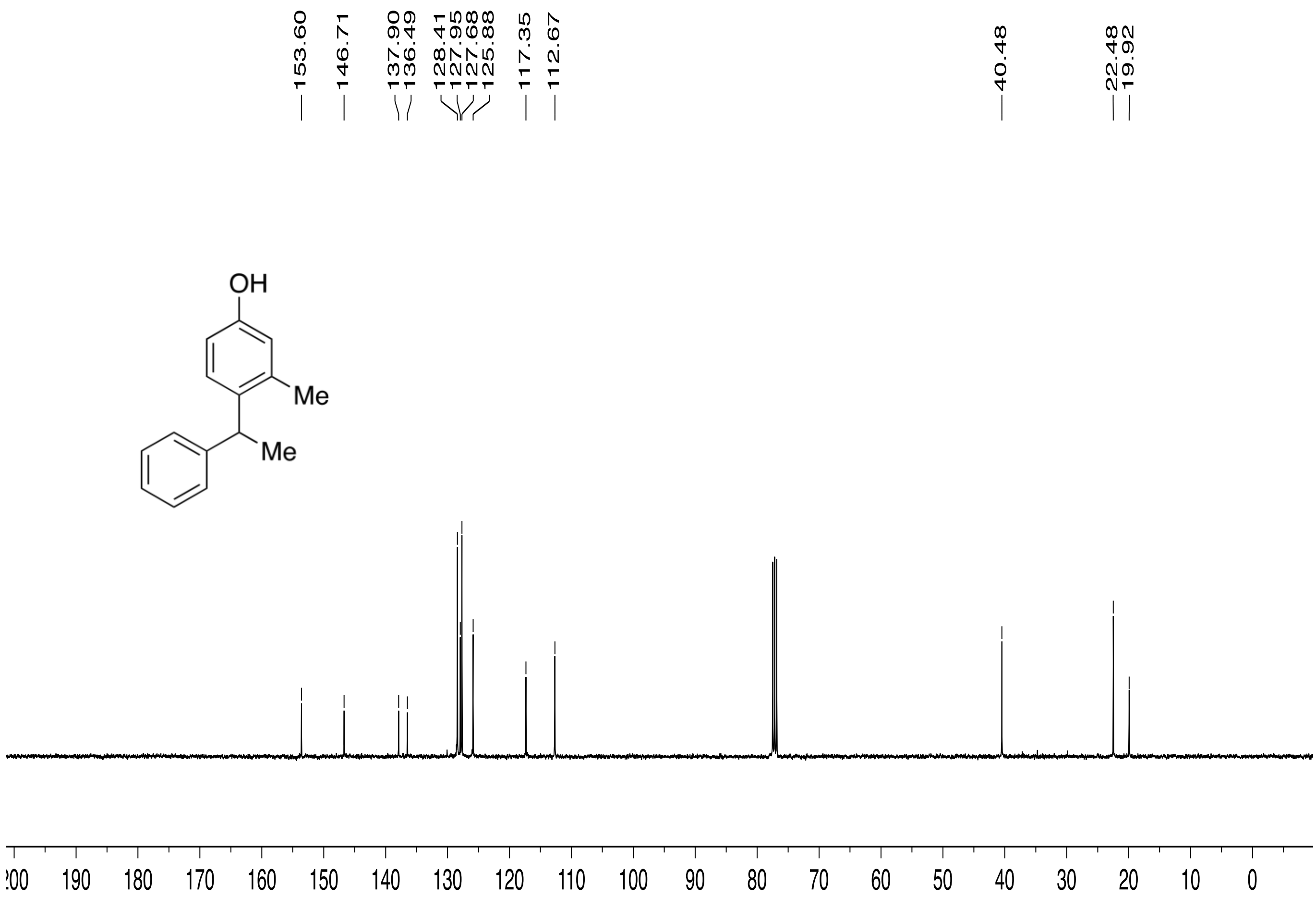

153.60
146.71
137.90
136.49
128.41
127.95
127.68
125.88
117.35
112.67
40.48
22.48
19.92
OH
Me
Me
200 190 180 170 160 150 140 130 120 110 100 90 80 70 60 50 40 30 20 10 0

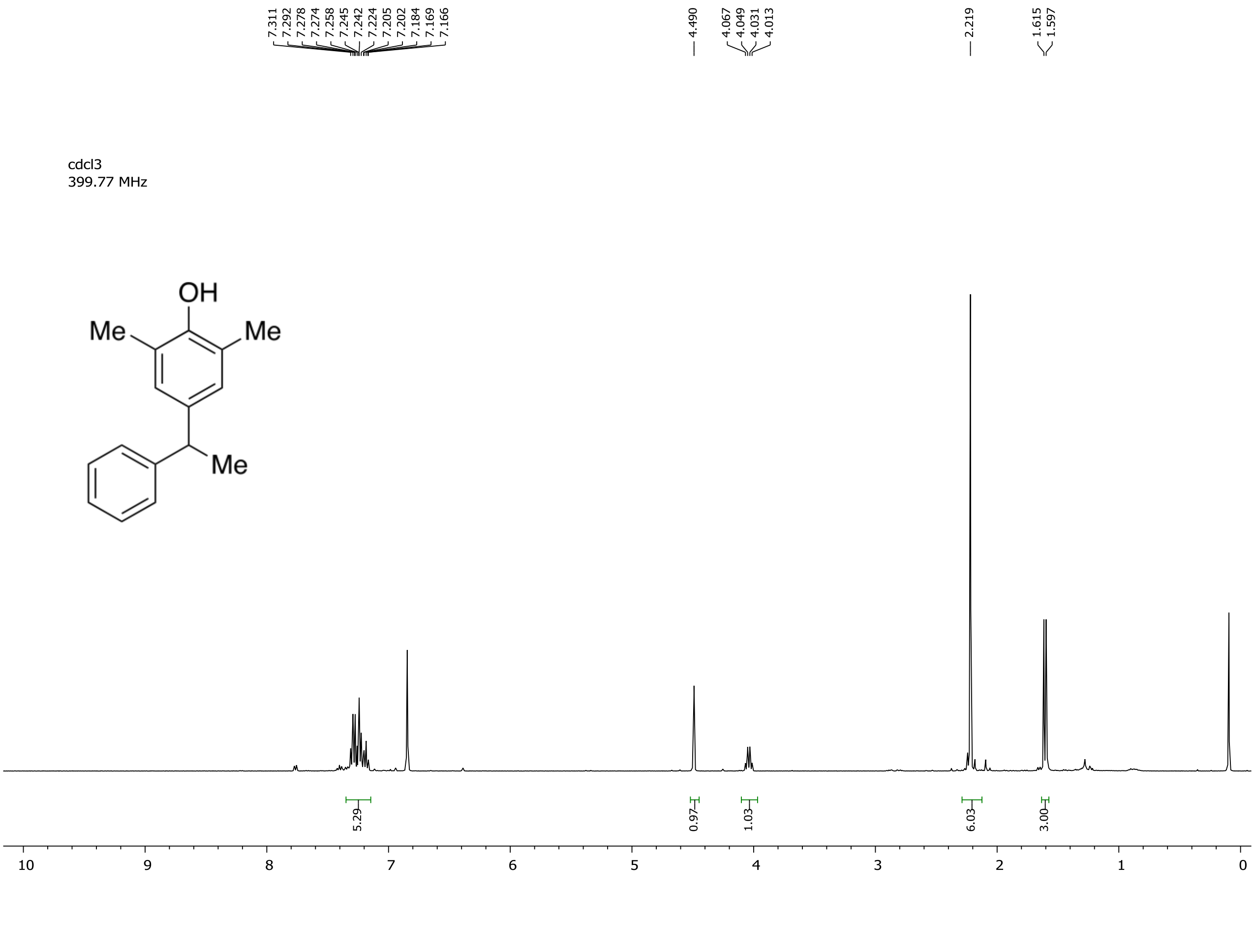

7.311 7.292 7.278 7.274 7.258 7.245 7.242 7.224 7.205 7.202 7.184 7.169 7.166
4.490
4.067 4.049 4.031 4.013
2.219
1.615 1.597
cdcl3
399.77 MHz
OH
Me
Me
Me
5.29
0.97
1.03
6.03
3.00
10 9 8 7 6 5 4 3 2 1 0

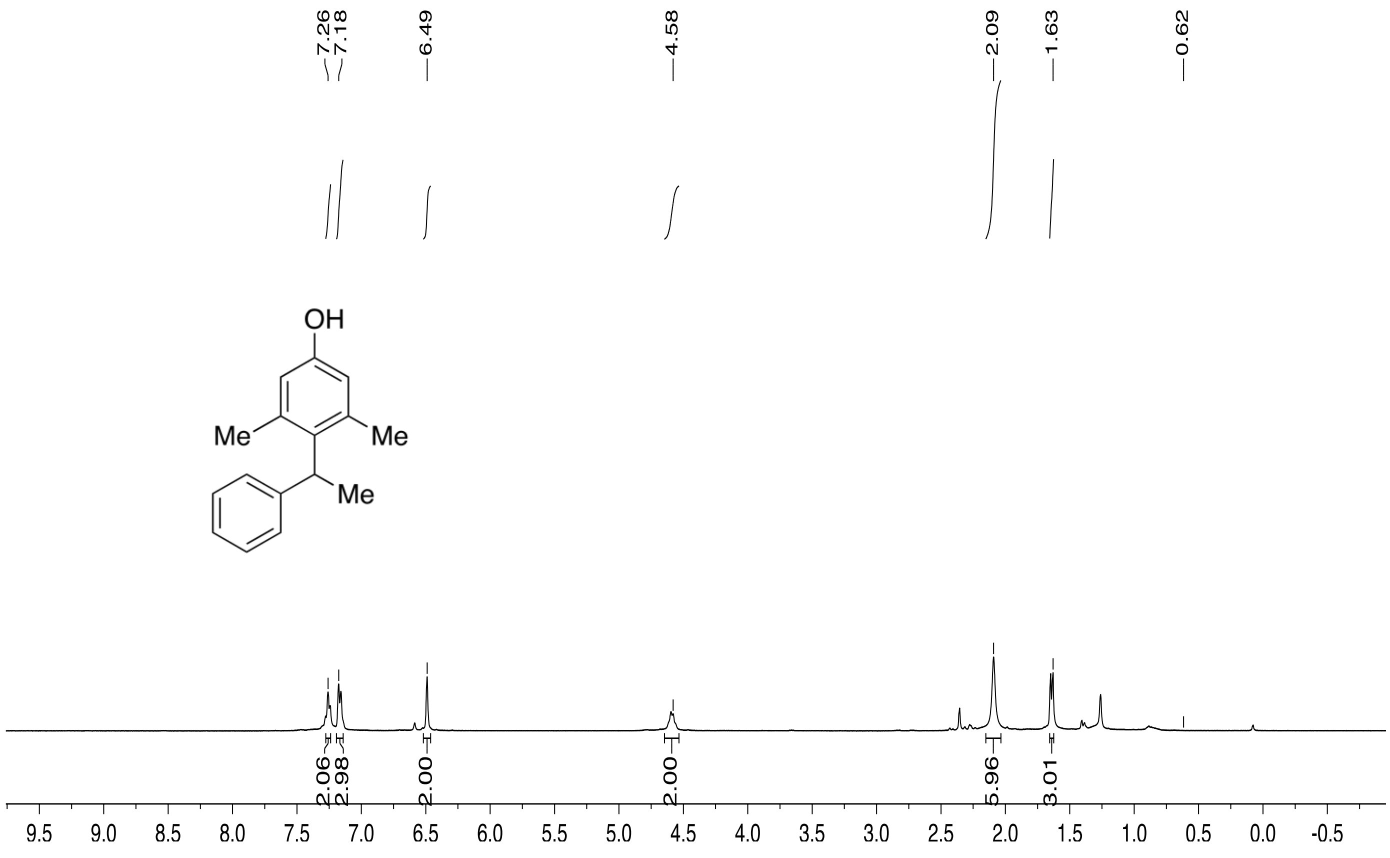
7.26
7.18
6.49
4.58
2.09
1.63
0.62
OH
Me
Me
Me
2.06
2.98
2.00
2.00
5.96
3.01
9.5 9.0 8.5 8.0 7.5 7.0 6.5 6.0 5.5 5.0 4.5 4.0 3.5 3.0 2.5 2.0 1.5 1.0 0.5 0.0 -0.5

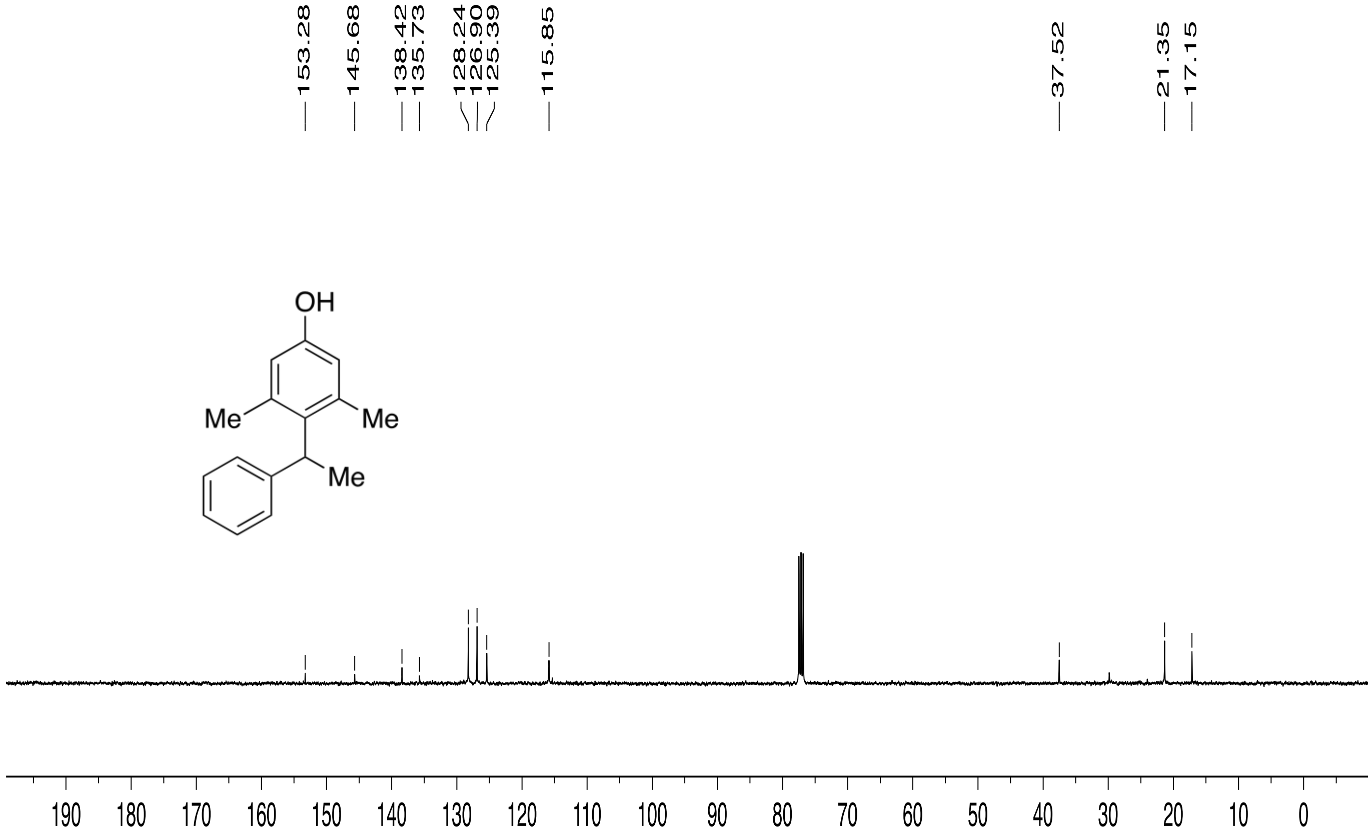
153.28
145.68
138.42
135.73
128.24
126.90
125.39
115.85
37.52
21.35
17.15
OH
Me
Me
Me
190 180 170 160 150 140 130 120 110 100 90 80 70 60 50 40 30 20 10 0

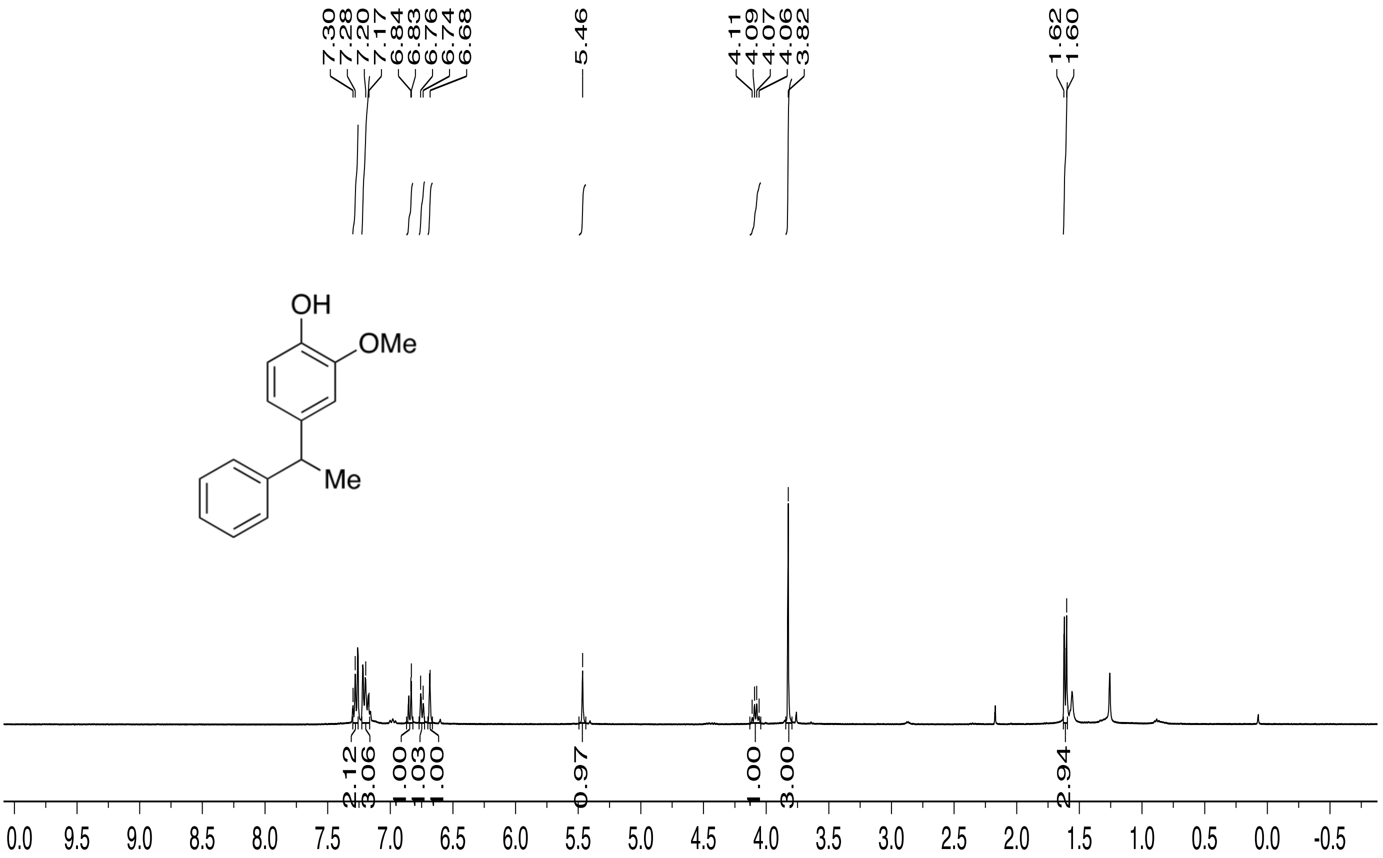
7.30
7.28
7.20
7.17
6.84
6.83
6.76
6.74
6.68
5.46
4.11
4.09
4.07
4.06
3.82
1.62
1.60
OH
OMe
Me
2.12
3.06
1.00
1.03
1.00
0.97
1.00
3.00
2.94

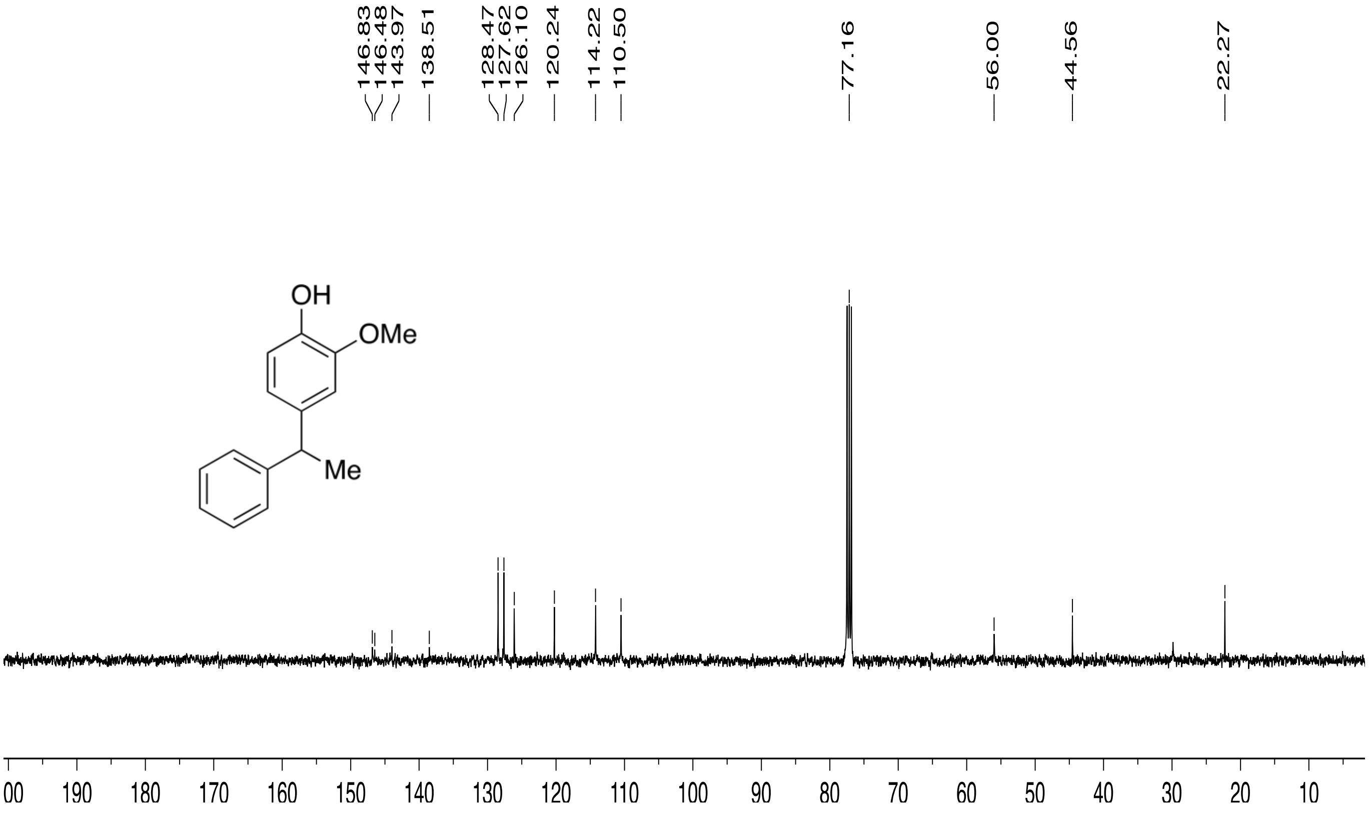
146.83
146.48
143.97
138.51
128.47
127.62
126.10
120.24
114.22
110.50
77.16
56.00
44.56
22.27
OH
OMe
Me

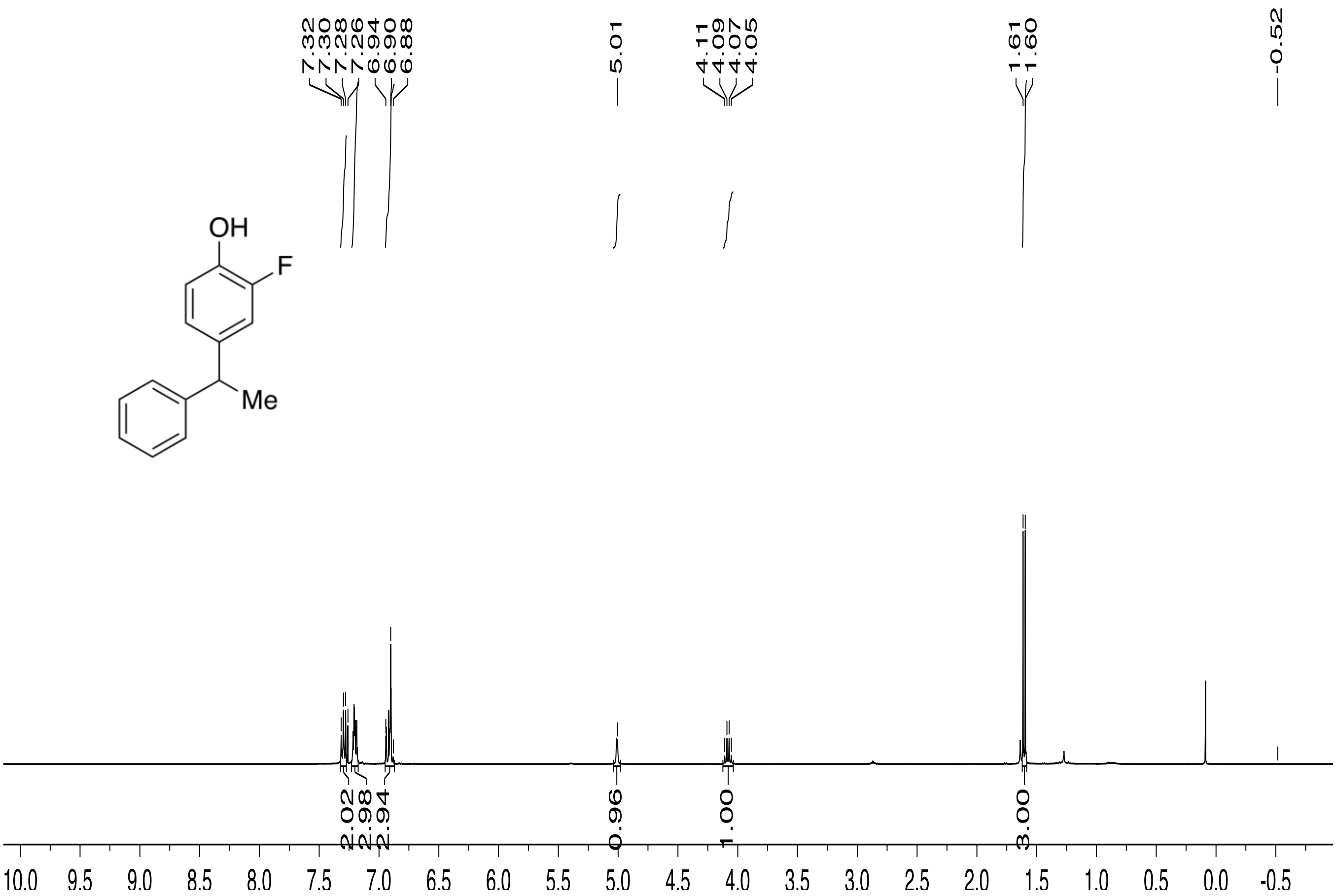

7.32
7.30
7.28
7.26
6.94
6.90
6.88
5.01
4.11
4.09
4.07
4.05
1.61
1.60
-0.52
OH
F
Me
2.02
2.98
2.94
0.96
1.00
3.00
10.0 9.5 9.0 8.5 8.0 7.5 7.0 6.5 6.0 5.5 5.0 4.5 4.0 3.5 3.0 2.5 2.0 1.5 1.0 0.5 0.0 -0.5

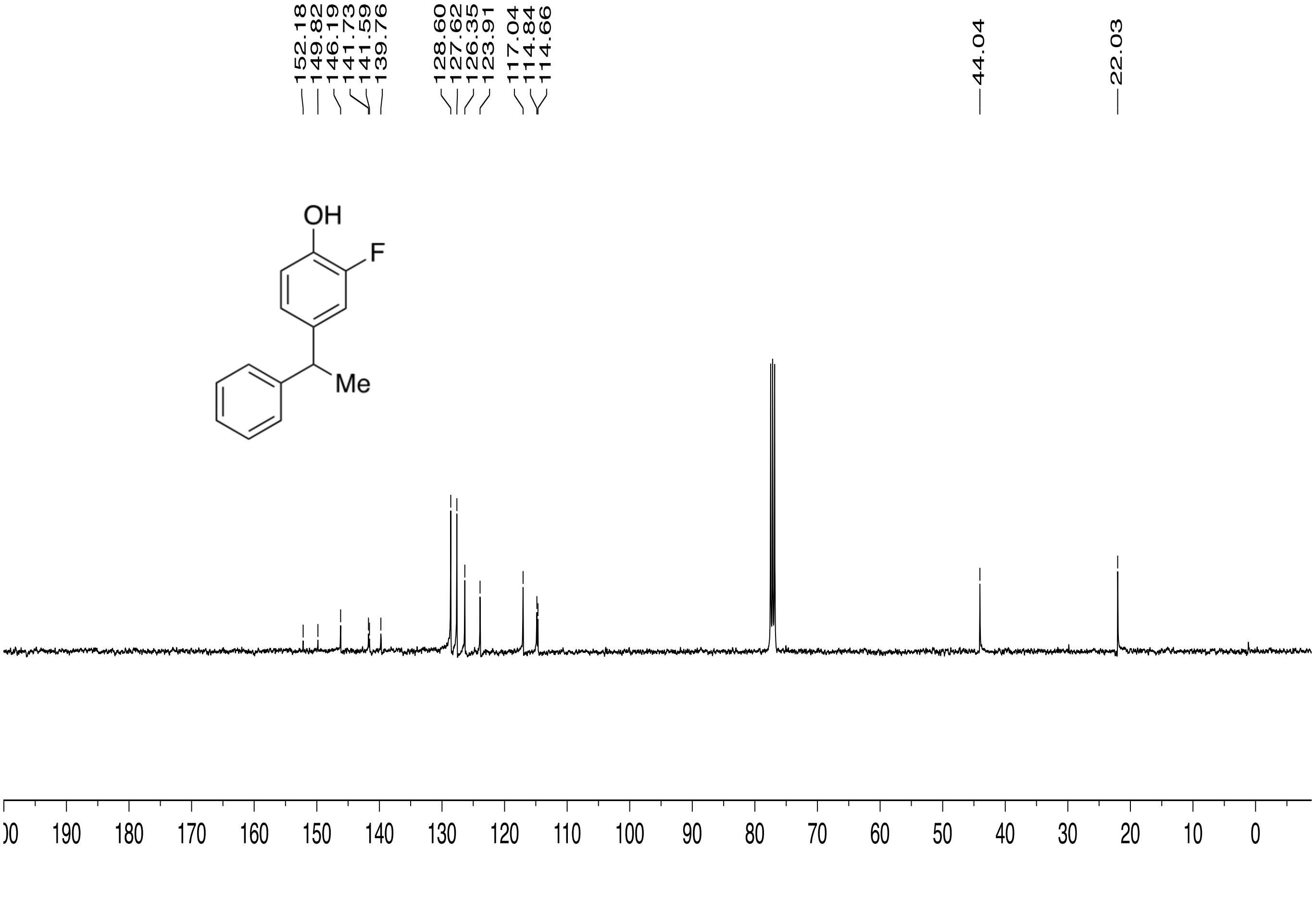

152.18
149.82
146.19
141.73
141.59
139.76
128.60
127.62
126.35
123.91
117.04
114.84
114.66
44.04
22.03
OH
F
Me
190 180 170 160 150 140 130 120 110 100 90 80 70 60 50 40 30 20 10 0

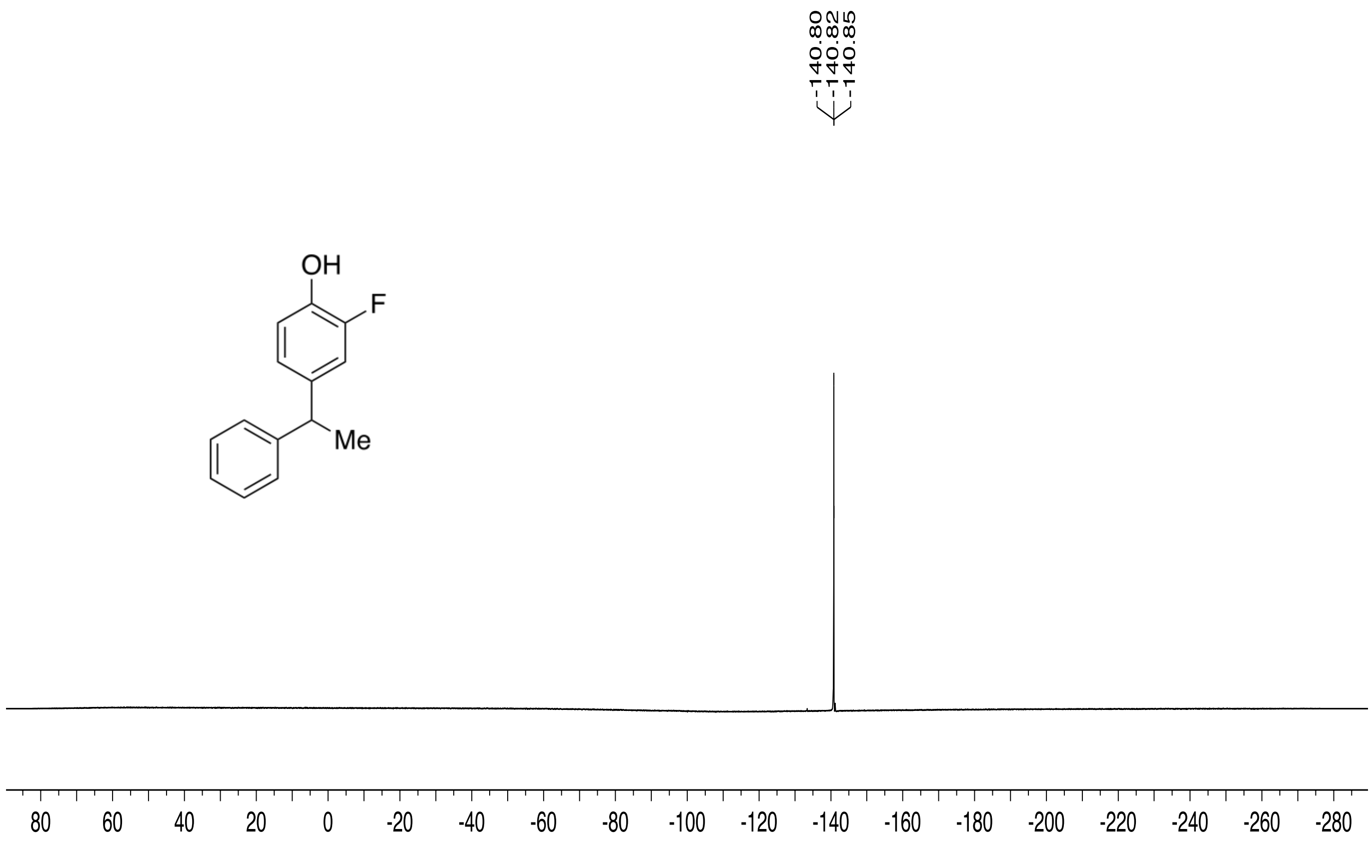
-140.80
-140.82
-140.85
OH
F
Me
80 60 40 20 0 -20 -40 -60 -80 -100 -120 -140 -160 -180 -200 -220 -240 -260 -280

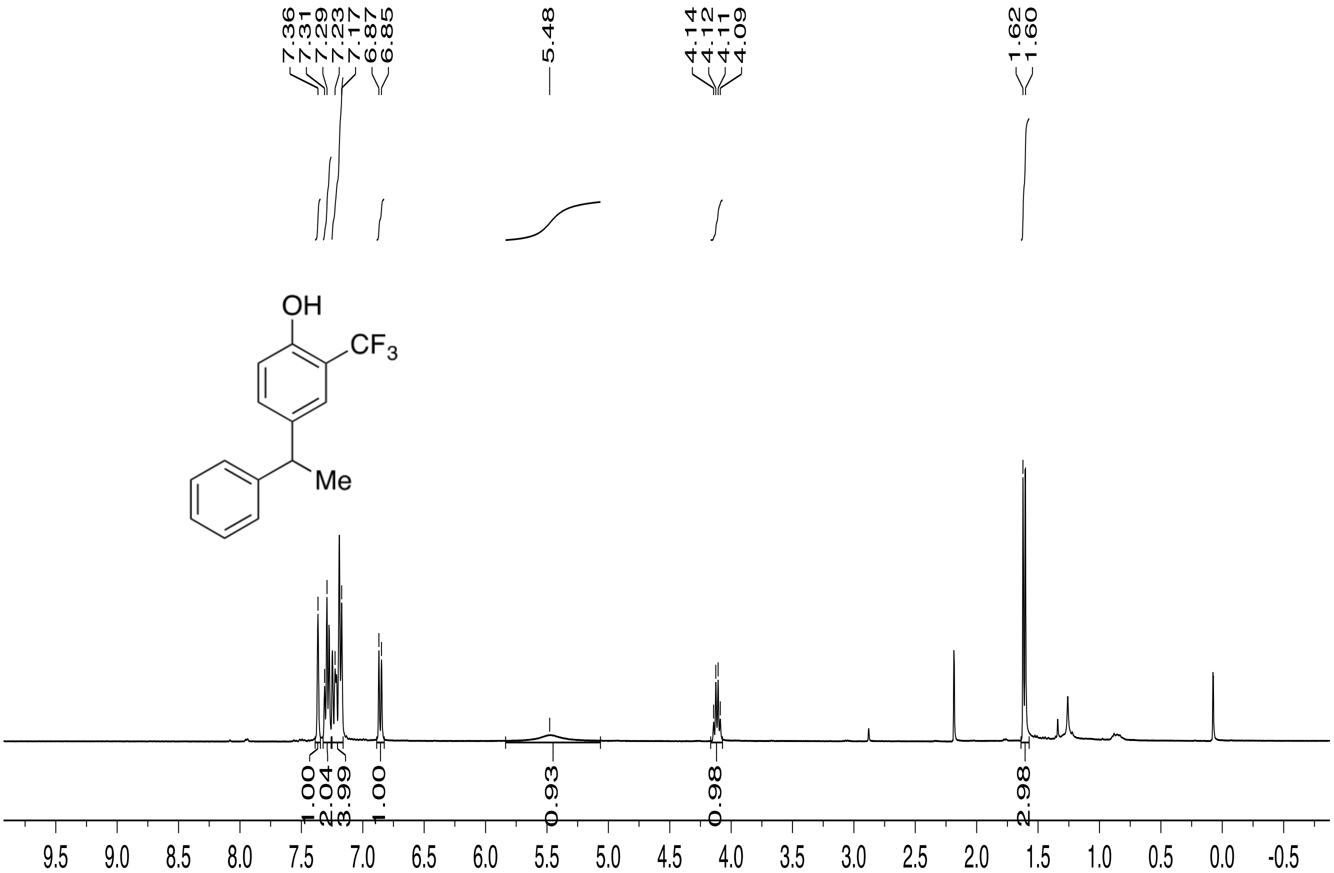
7.36
7.31
7.29
7.23
7.17
6.87
6.85
5.48
4.14
4.12
4.11
4.09
1.62
1.60
OH
CF3
Me
1.00
2.04
3.99
1.00
0.93
0.98
2.98
9.5 9.0 8.5 8.0 7.5 7.0 6.5 6.0 5.5 5.0 4.5 4.0 3.5 3.0 2.5 2.0 1.5 1.0 0.5 0.0 -0.5

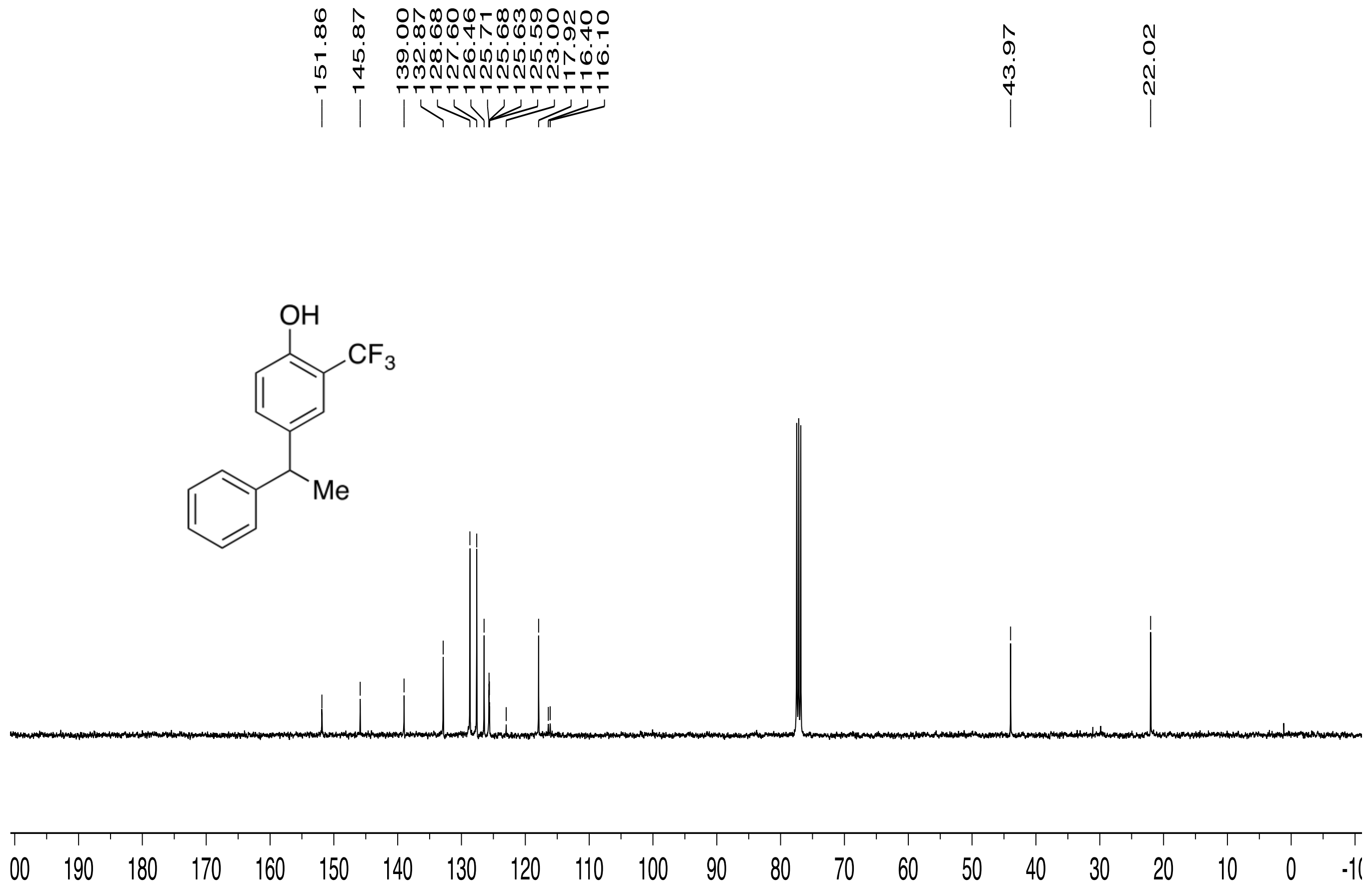
151.86
145.87
139.00
132.87
128.68
127.60
126.46
125.71
125.68
125.63
125.59
123.00
117.92
116.40
116.10
43.97
22.02
OH
CF_3
Me
00 190 180 170 160 150 140 130 120 110 100 90 80 70 60 50 40 30 20 10 0 -1(

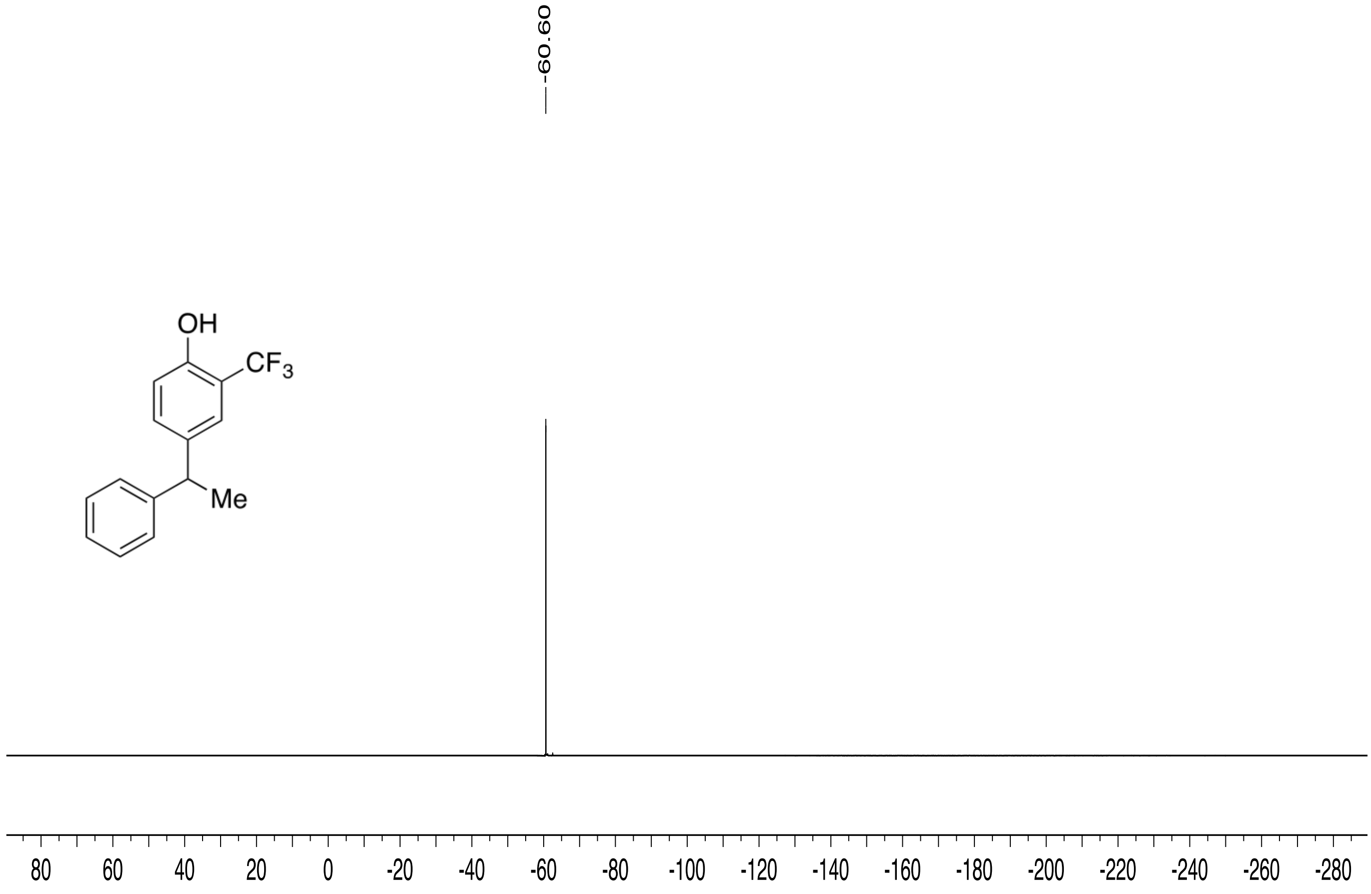
-60.60
OH
CF_3
Me
80 60 40 20 0 -20 -40 -60 -80 -100 -120 -140 -160 -180 -200 -220 -240 -260 -280

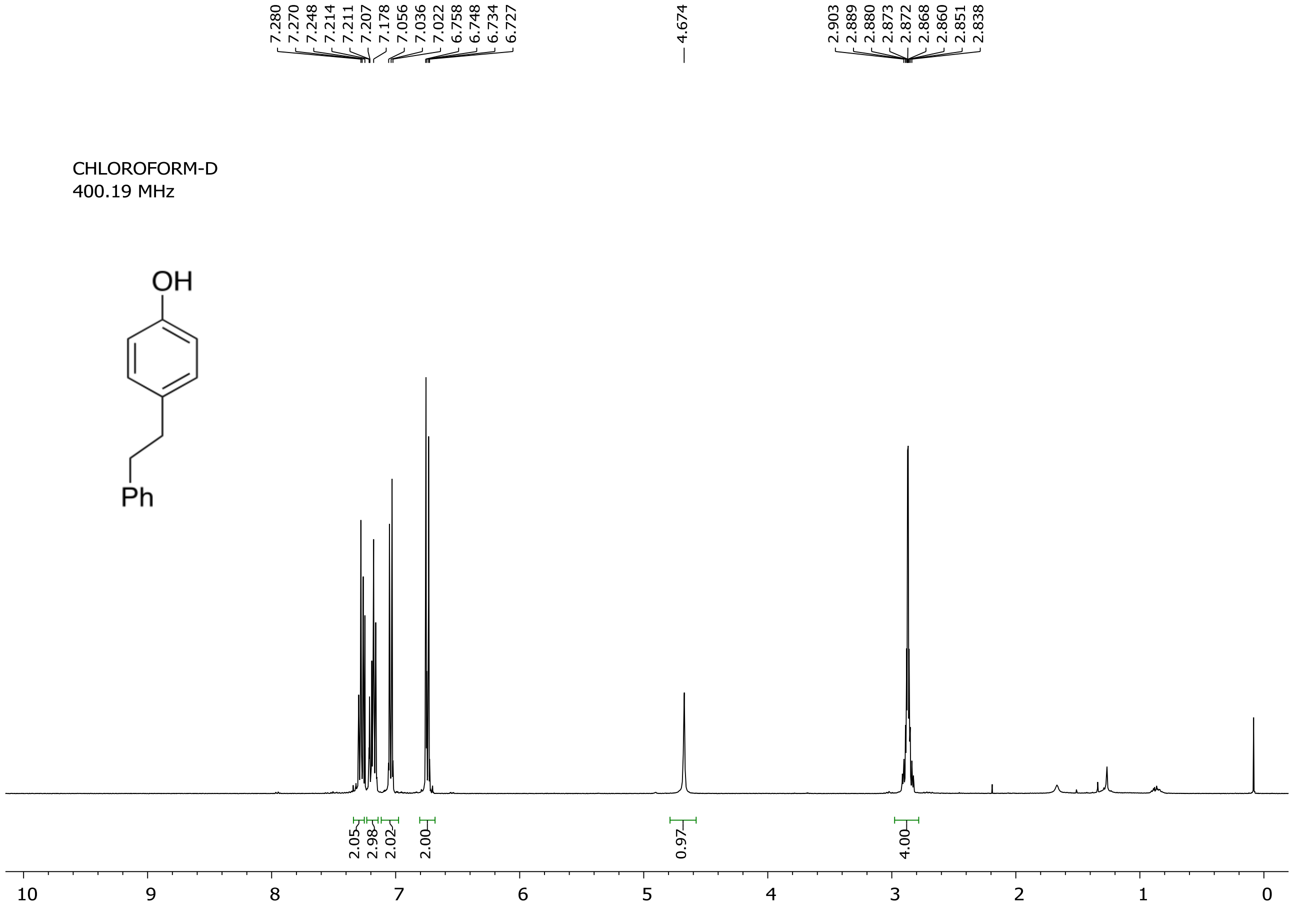
7.280
7.270
7.248
7.214
7.211
7.207
7.178
7.056
7.036
7.022
6.758
6.748
6.734
6.727
4.674
2.903
2.889
2.880
2.873
2.872
2.868
2.860
2.851
2.838
CHLOROFORM-D
400.19 MHz
OH
Ph
2.05
2.98
2.02
2.00
0.97
4.00
10
9
8
7
6
5
4
3
2
1
0

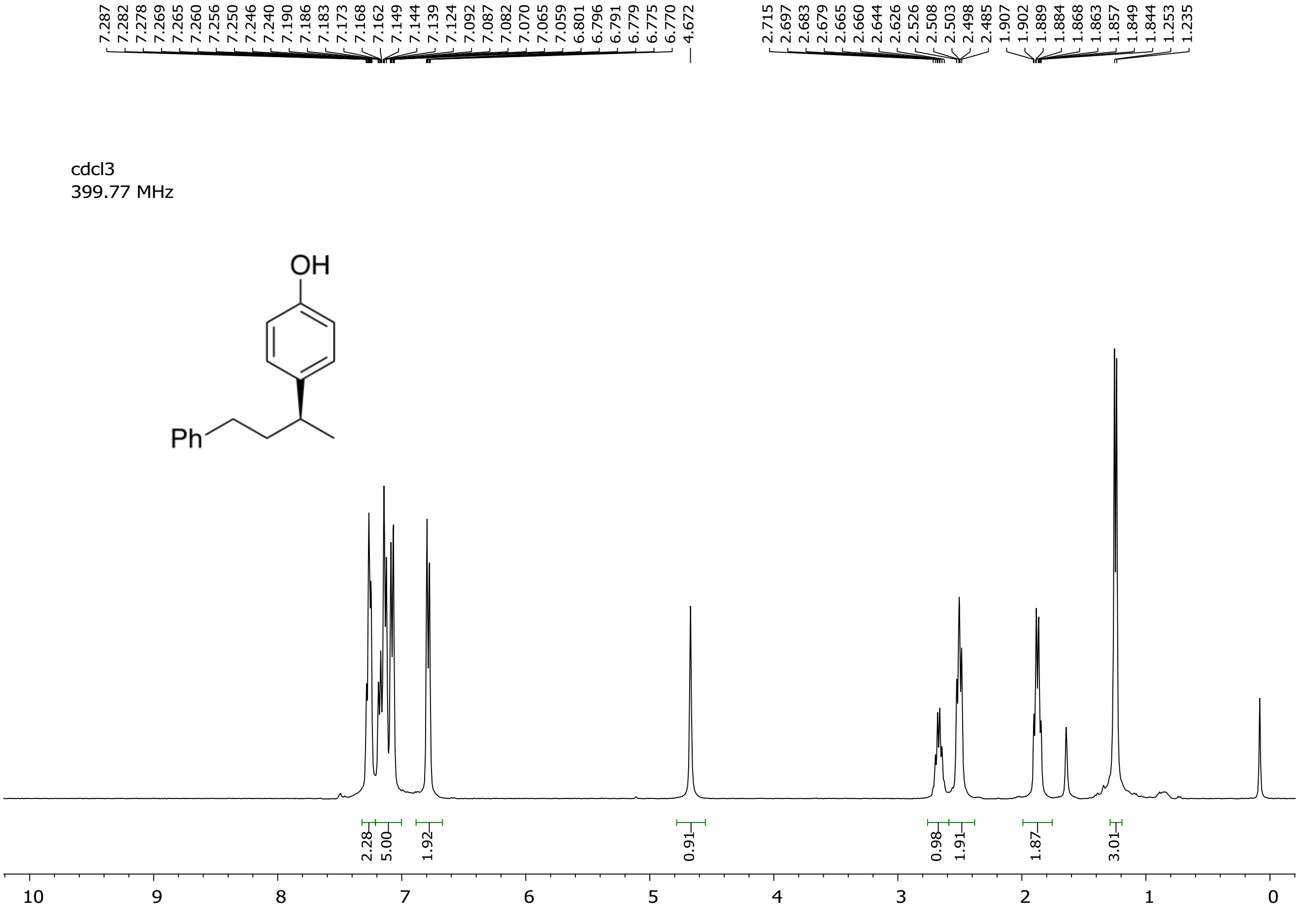
7.287
7.282
7.278
7.269
7.265
7.260
7.256
7.250
7.246
7.240
7.190
7.186
7.183
7.173
7.168
7.162
7.149
7.144
7.139
7.124
7.092
7.087
7.082
7.070
7.065
7.059
6.801
6.796
6.791
6.779
6.775
6.770
4.672
2.715
2.697
2.683
2.679
2.665
2.660
2.644
2.626
2.526
2.508
2.503
2.498
2.485
1.907
1.902
1.889
1.884
1.868
1.863
1.857
1.849
1.844
1.253
1.235
cdcl3
399.77 MHz
OH
Ph
2.28
5.00
1.92
0.91
0.98
1.91
1.87
3.01
10
9
8
7
6
5
4
3
2
1
0

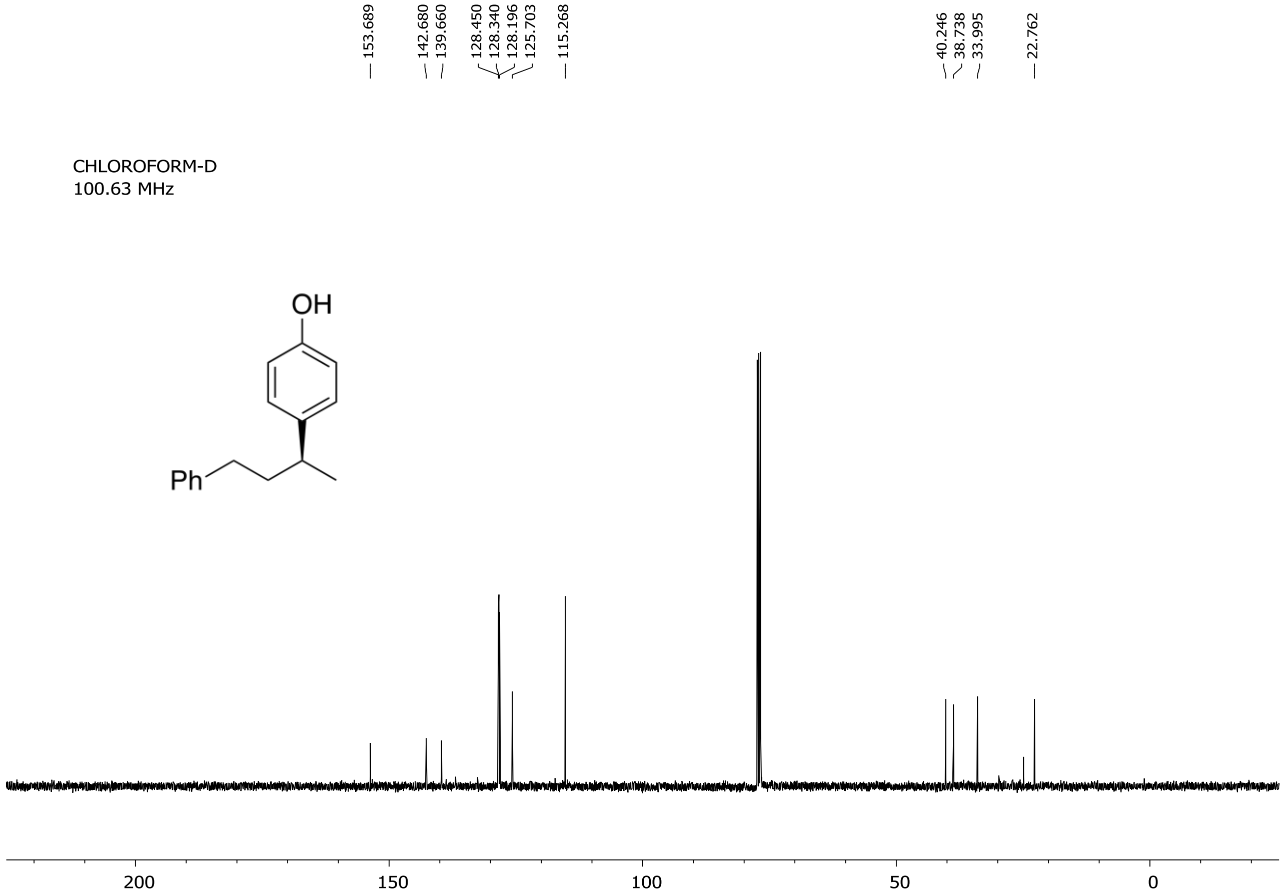
153.689
142.680
139.660
128.450
128.340
128.196
125.703
115.268
40.246
38.738
33.995
22.762
CHLOROFORM-D
100.63 MHz
OH
Ph
200
150
100
50
0

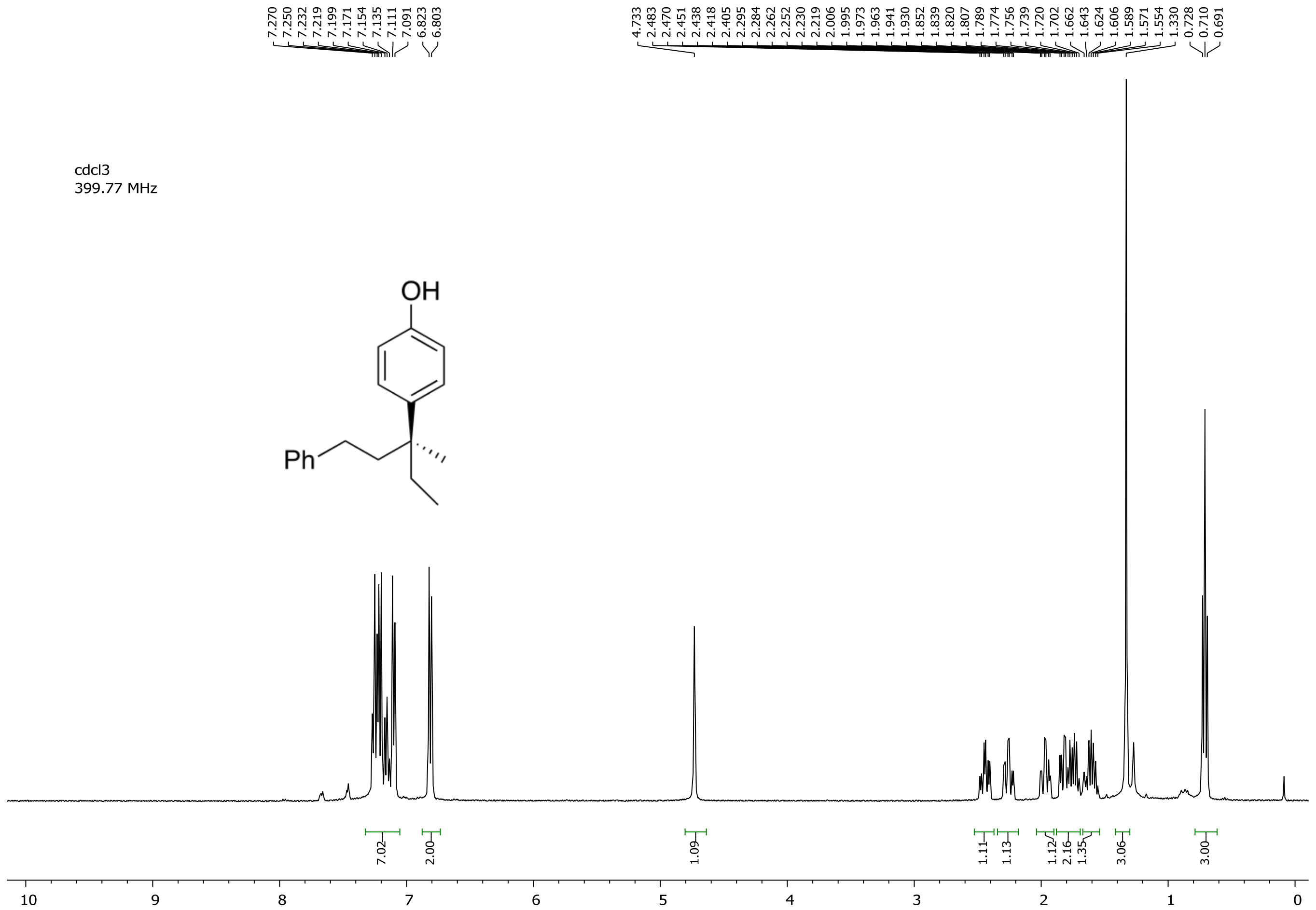
7.270
7.250
7.232
7.219
7.199
7.171
7.154
7.135
7.111
7.091
6.823
6.803
4.733
2.483
2.470
2.451
2.438
2.418
2.405
2.295
2.284
2.262
2.252
2.230
2.219
2.006
1.995
1.973
1.963
1.941
1.930
1.852
1.839
1.820
1.807
1.789
1.774
1.756
1.739
1.720
1.702
1.662
1.643
1.624
1.606
1.589
1.571
1.554
1.330
0.728
0.710
0.691
cdcl3
399.77 MHz
OH
Ph
7.02
2.00
1.09
1.11
1.13
1.12
2.16
1.35
3.06
3.00
10
9
8
7
6
5
4
3
2
1
0

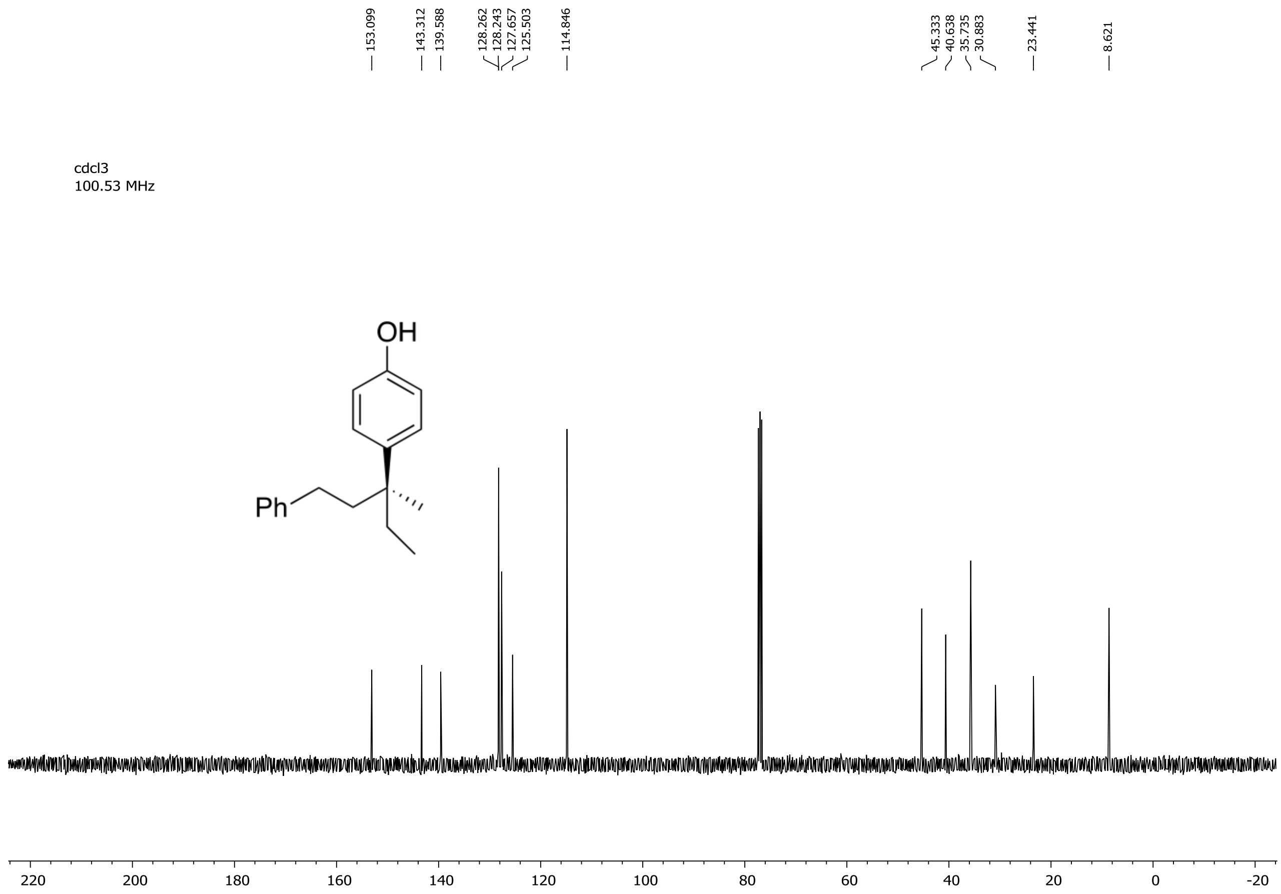
153.099
143.312
139.588
128.262
128.243
127.657
125.503
114.846
45.333
40.638
35.735
30.883
23.441
8.621
cdcl3
100.53 MHz
OH
Ph
220 200 180 160 140 120 100 80 60 40 20 0 -20

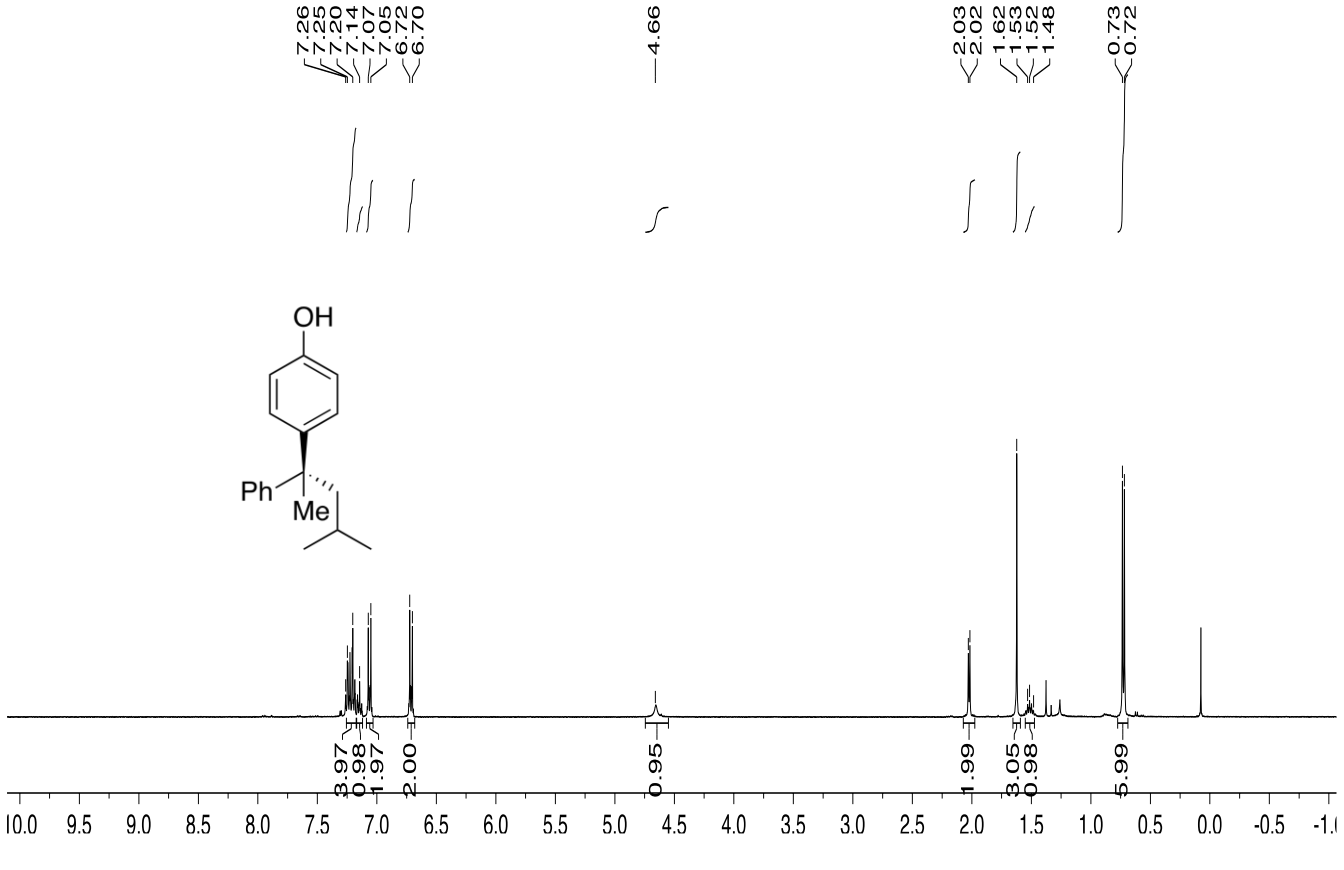
7.26
7.25
7.20
7.14
7.07
7.05
6.72
6.70
4.66
2.03
2.02
1.62
1.53
1.52
1.48
0.73
0.72
OH
Ph
Me
3.97
0.98
1.97
2.00
0.95
1.99
3.05
0.98
5.99
10.0 9.5 9.0 8.5 8.0 7.5 7.0 6.5 6.0 5.5 5.0 4.5 4.0 3.5 3.0 2.5 2.0 1.5 1.0 0.5 0.0 -0.5

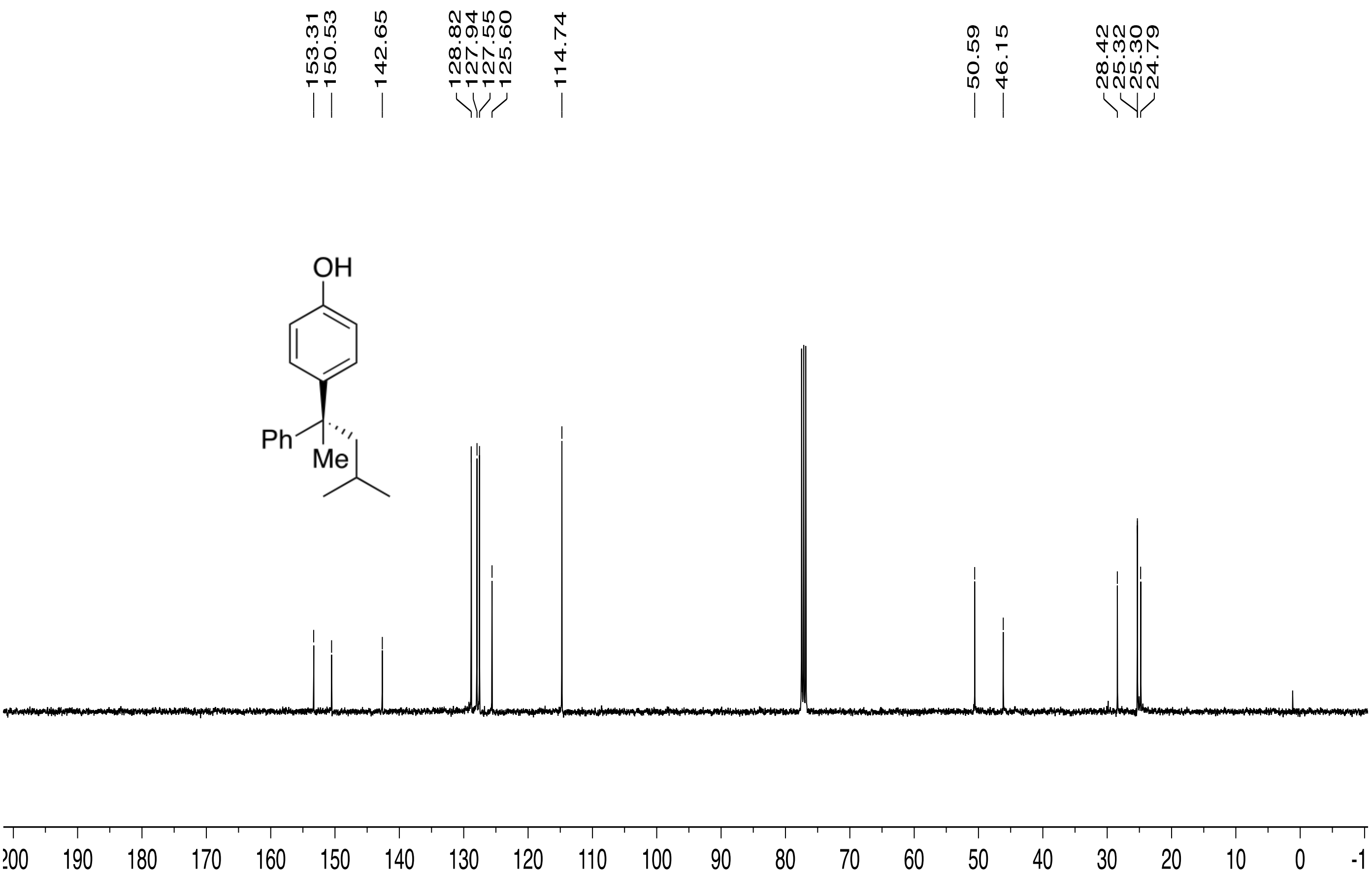

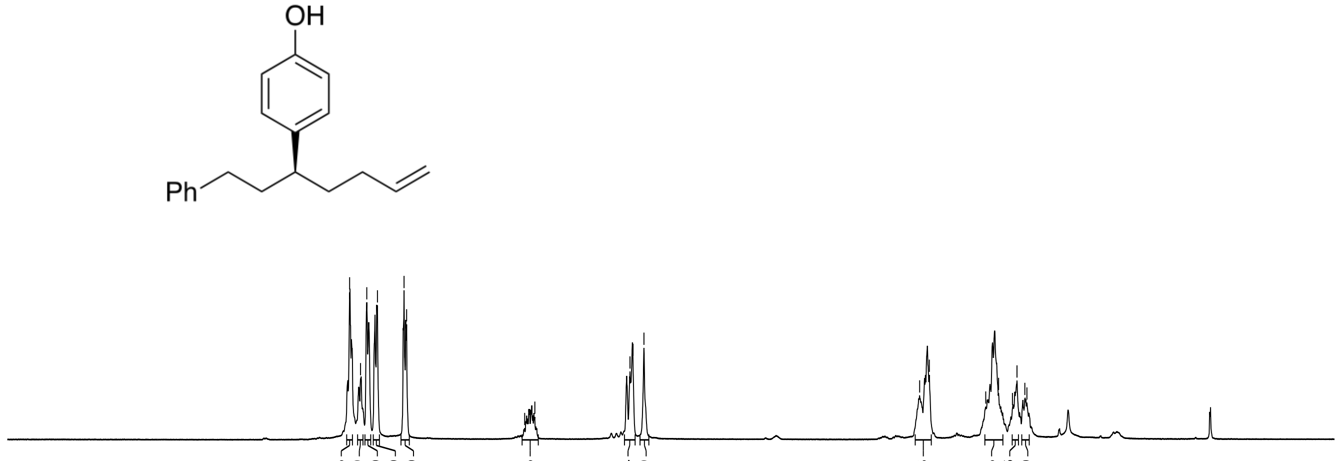

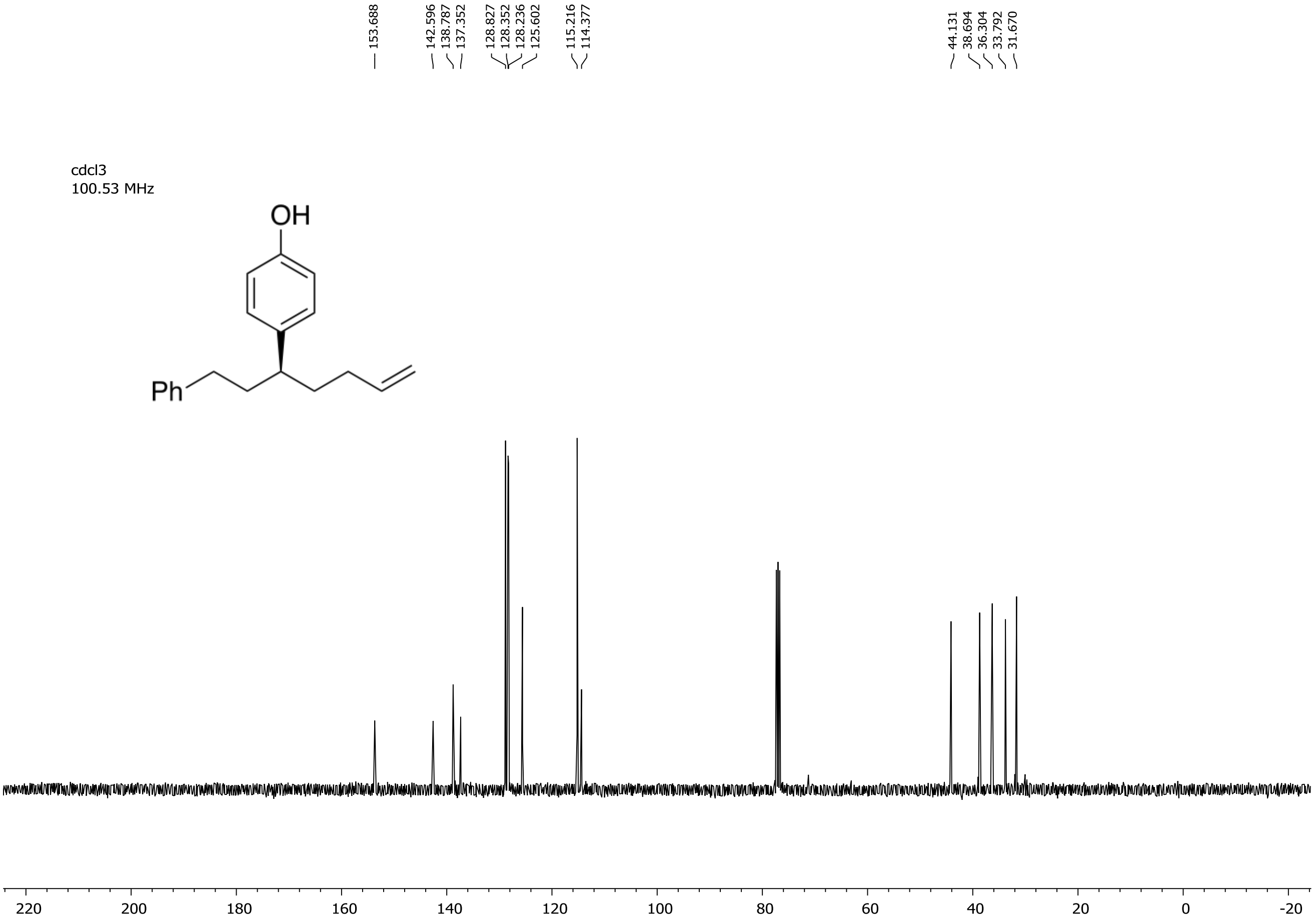
153.688
142.596
138.787
137.352
128.827
128.352
128.236
125.602
115.216
114.377
44.131
38.694
36.304
33.792
31.670
cdcl3
100.53 MHz
OH
Ph

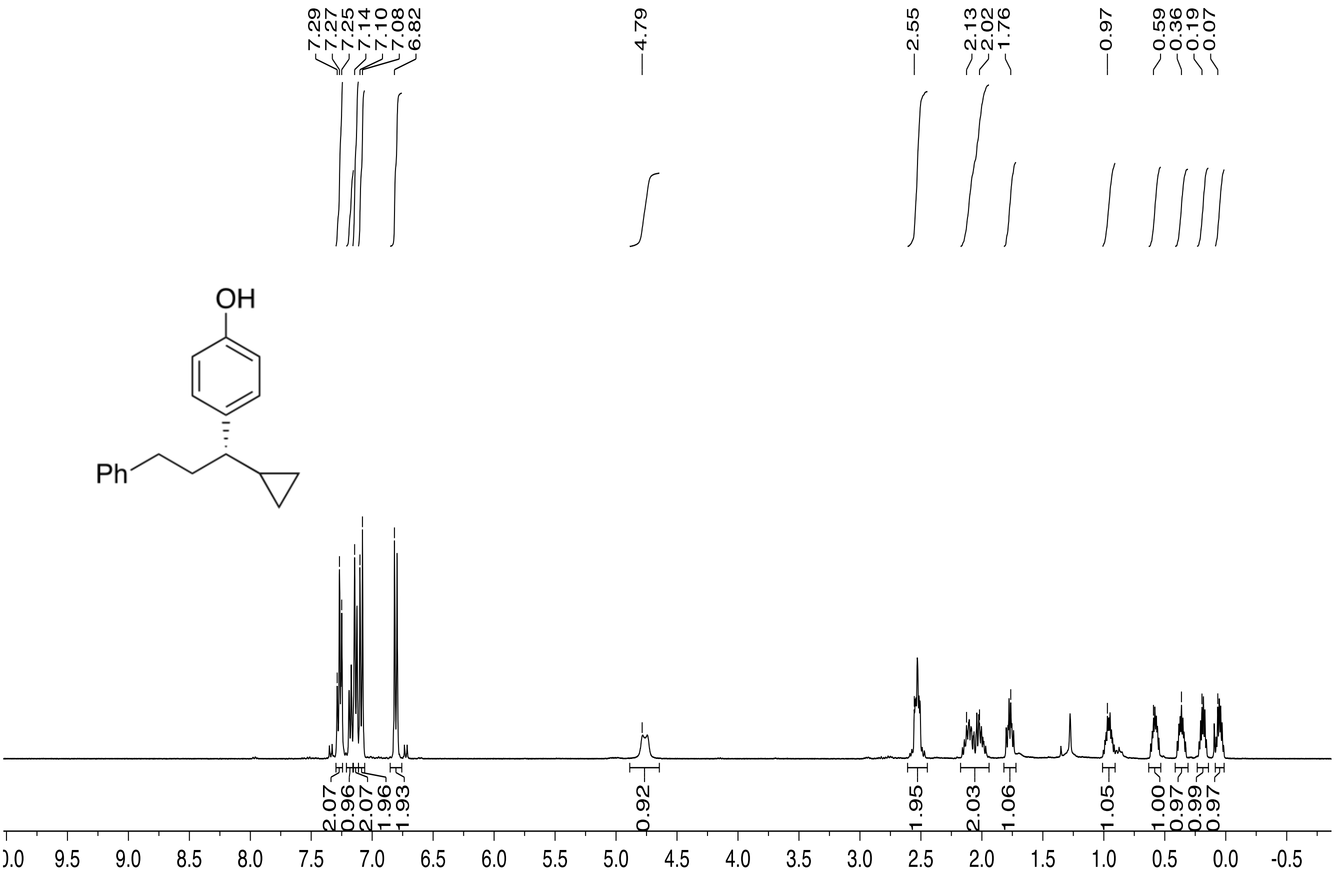
7.29
7.27
7.25
7.14
7.10
7.08
6.82
4.79
2.55
2.13
2.02
1.76
0.97
0.59
0.36
0.19
0.07
OH
Ph
2.07
0.96
2.07
1.96
1.93
0.92
1.95
2.03
1.06
1.05
1.00
0.97
0.99
0.97

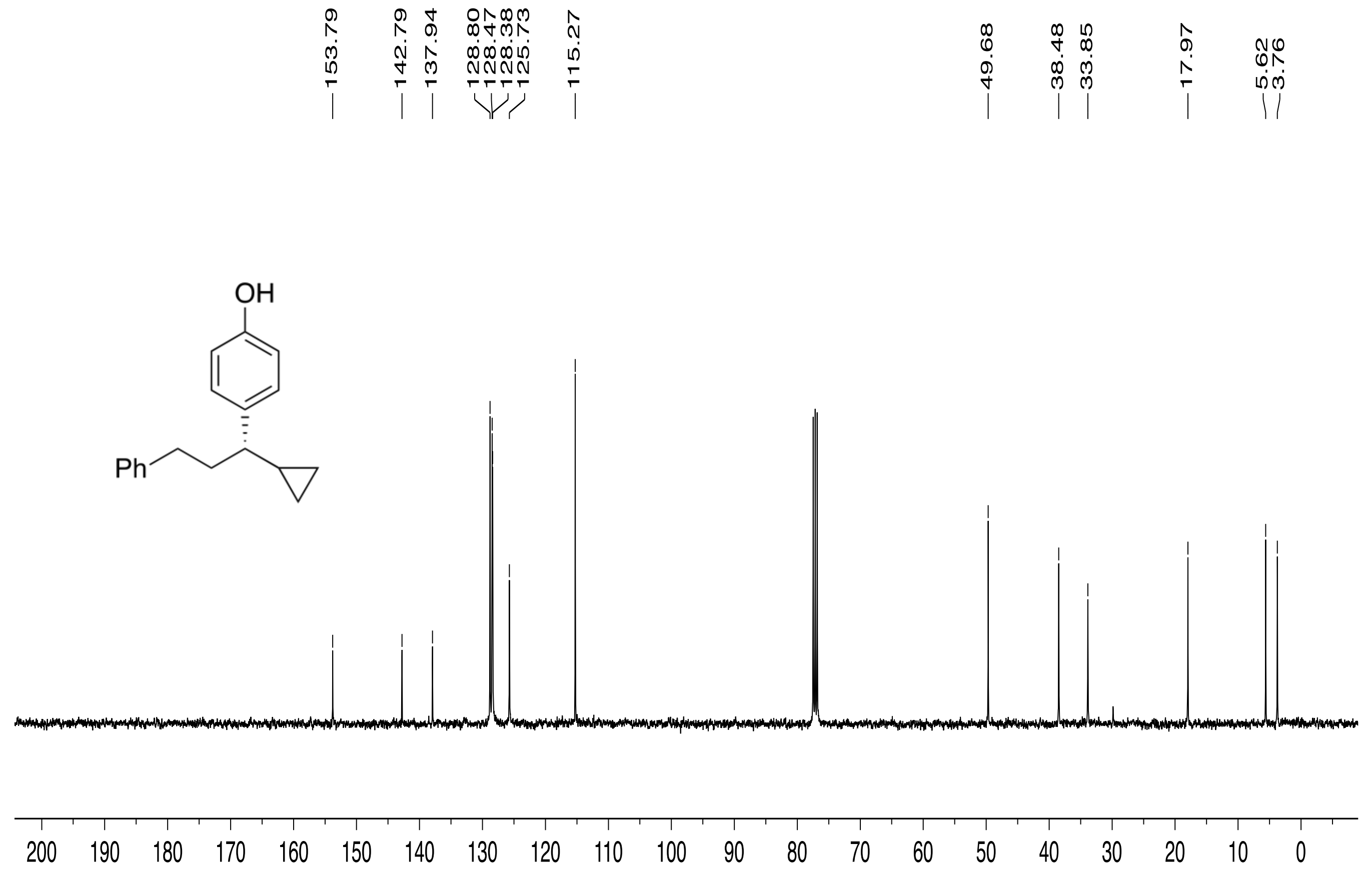
153.79
142.79
137.94
128.80
128.47
128.38
125.73
115.27
49.68
38.48
33.85
17.97
5.62
3.76
OH
Ph
200 190 180 170 160 150 140 130 120 110 100 90 80 70 60 50 40 30 20 10 0

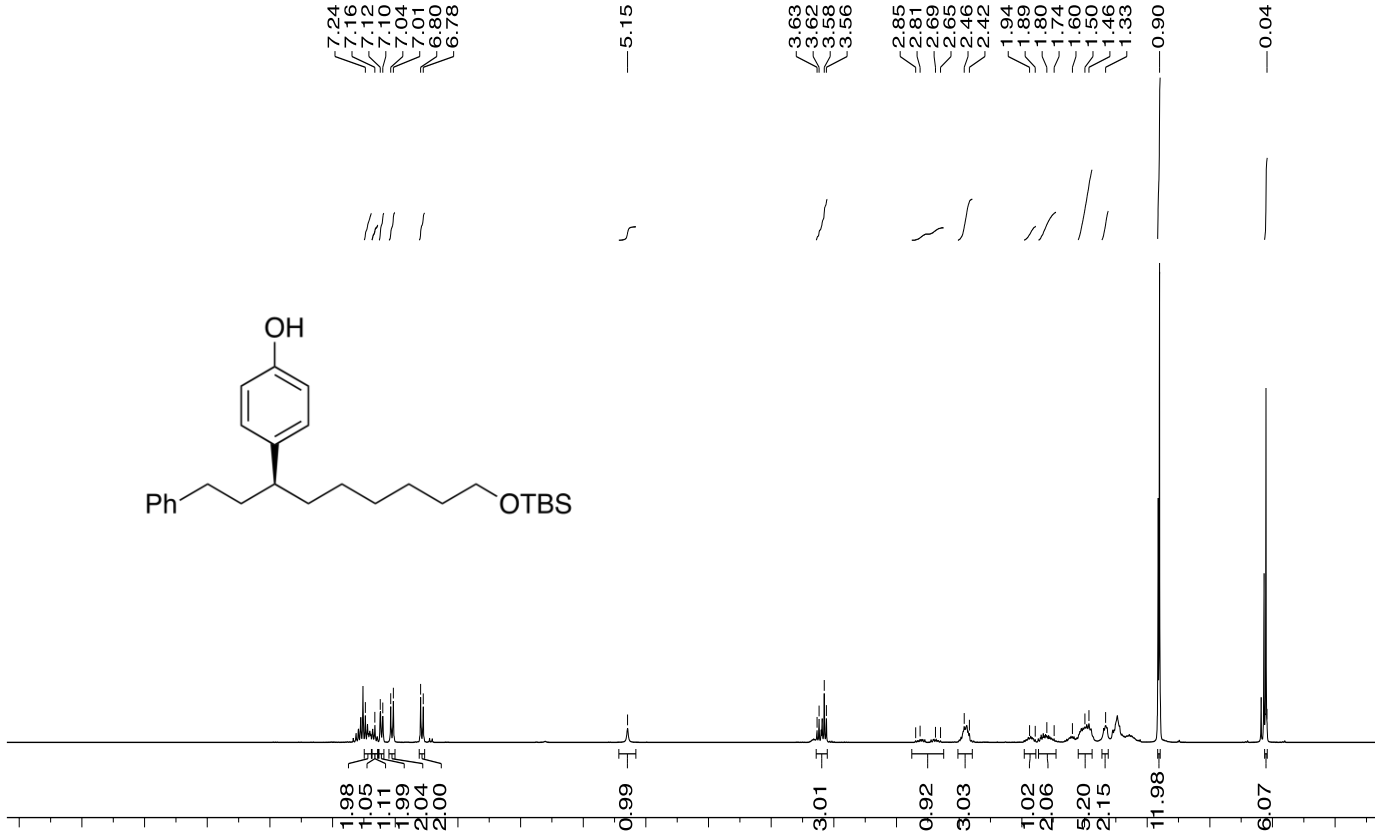
7.24
7.16
7.12
7.10
7.04
7.01
6.80
6.78
5.15
3.63
3.62
3.58
3.56
2.85
2.81
2.69
2.65
2.46
2.42
1.94
1.89
1.80
1.74
1.60
1.50
1.46
1.33
0.90
0.04
OH
Ph
OTBS
1.98
1.05
1.11
1.99
2.04
2.00
0.99
3.01
0.92
3.03
1.02
2.06
5.20
2.15
11.98
6.07
10.0 9.5 9.0 8.5 8.0 7.5 7.0 6.5 6.0 5.5 5.0 4.5 4.0 3.5 3.0 2.5 2.0 1.5 1.0 0.5 0.0 -0.5

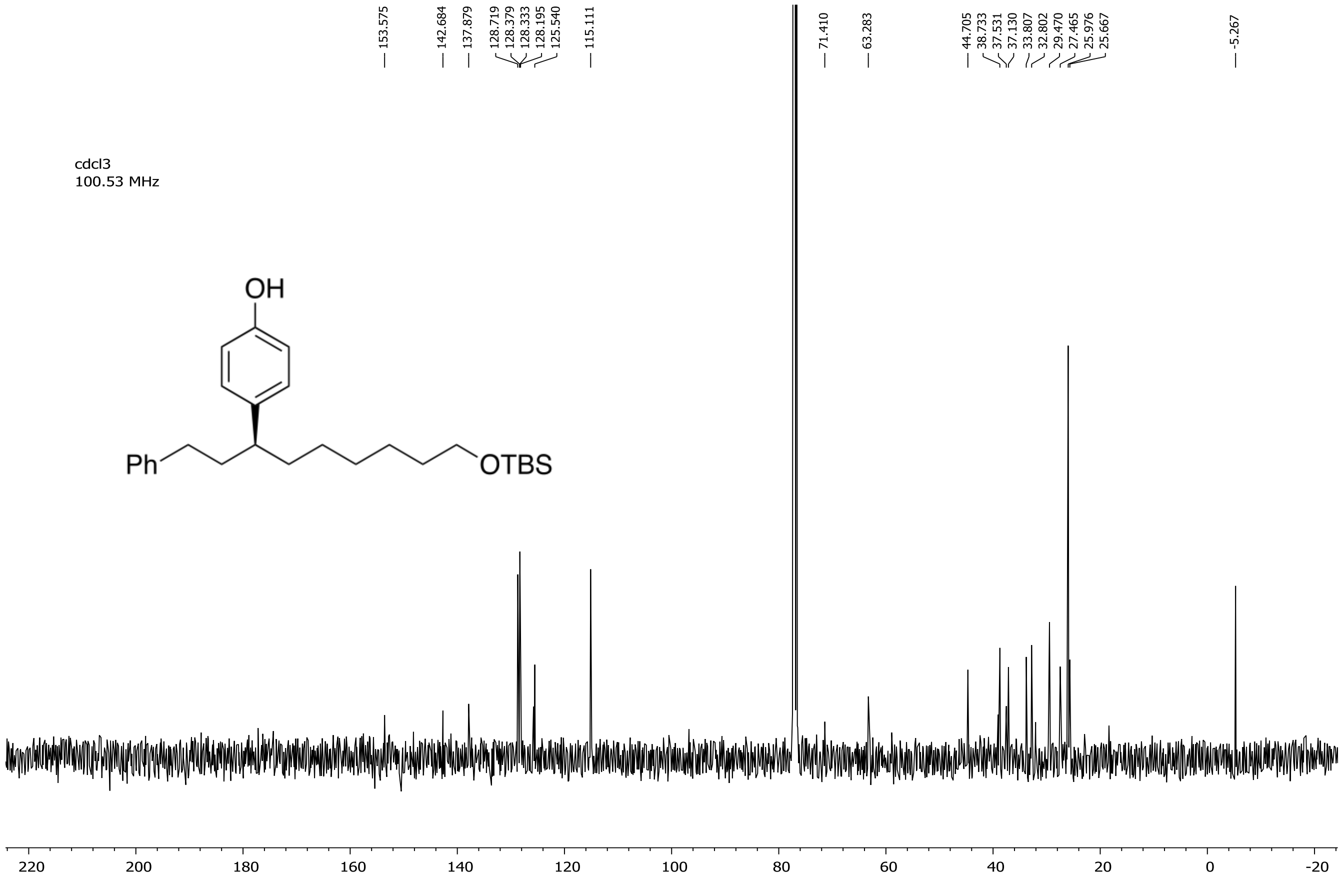
cdcl3
100.53 MHz
OH
Ph
OTBS

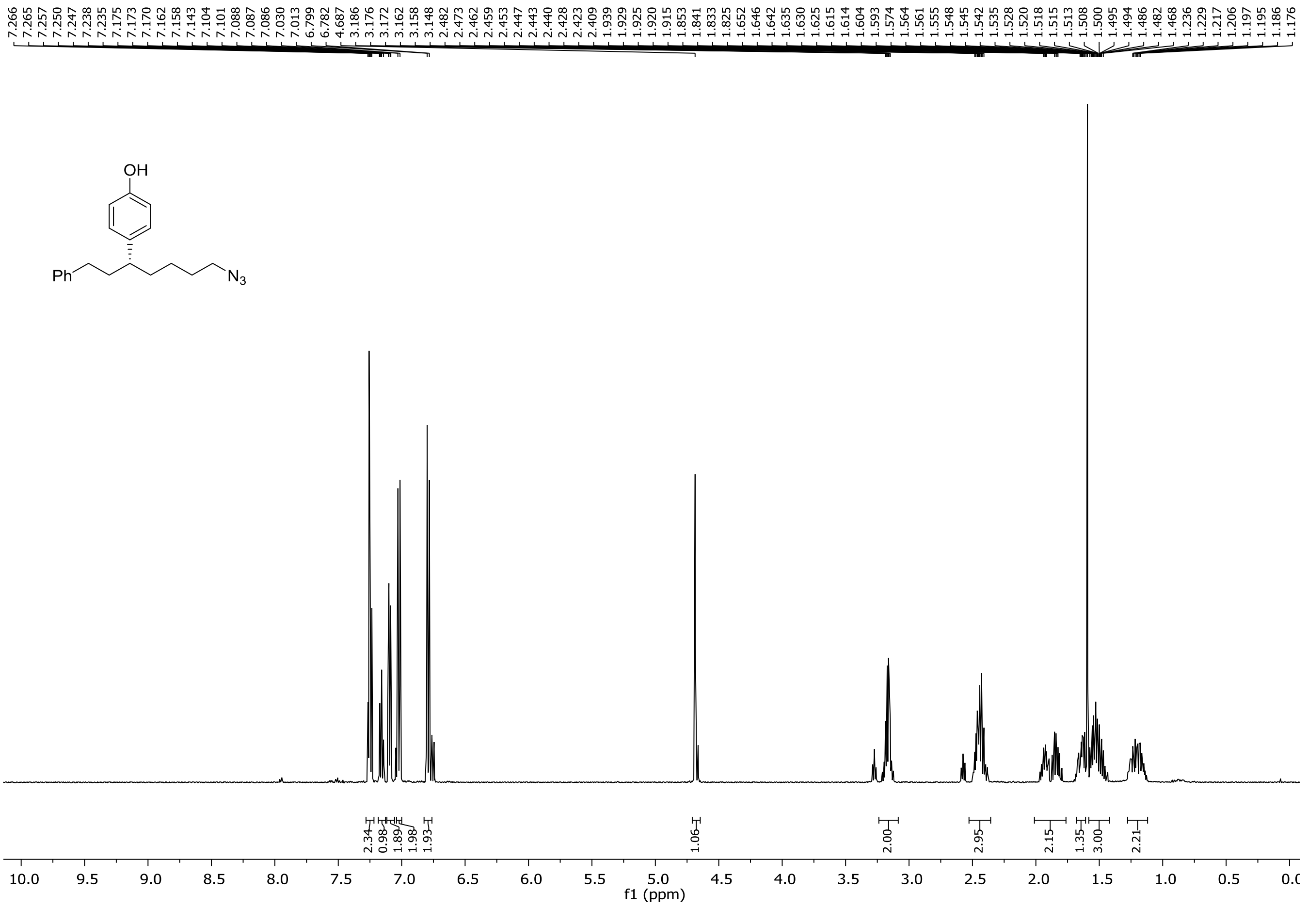
OH
Ph
N_3
f1 (ppm)

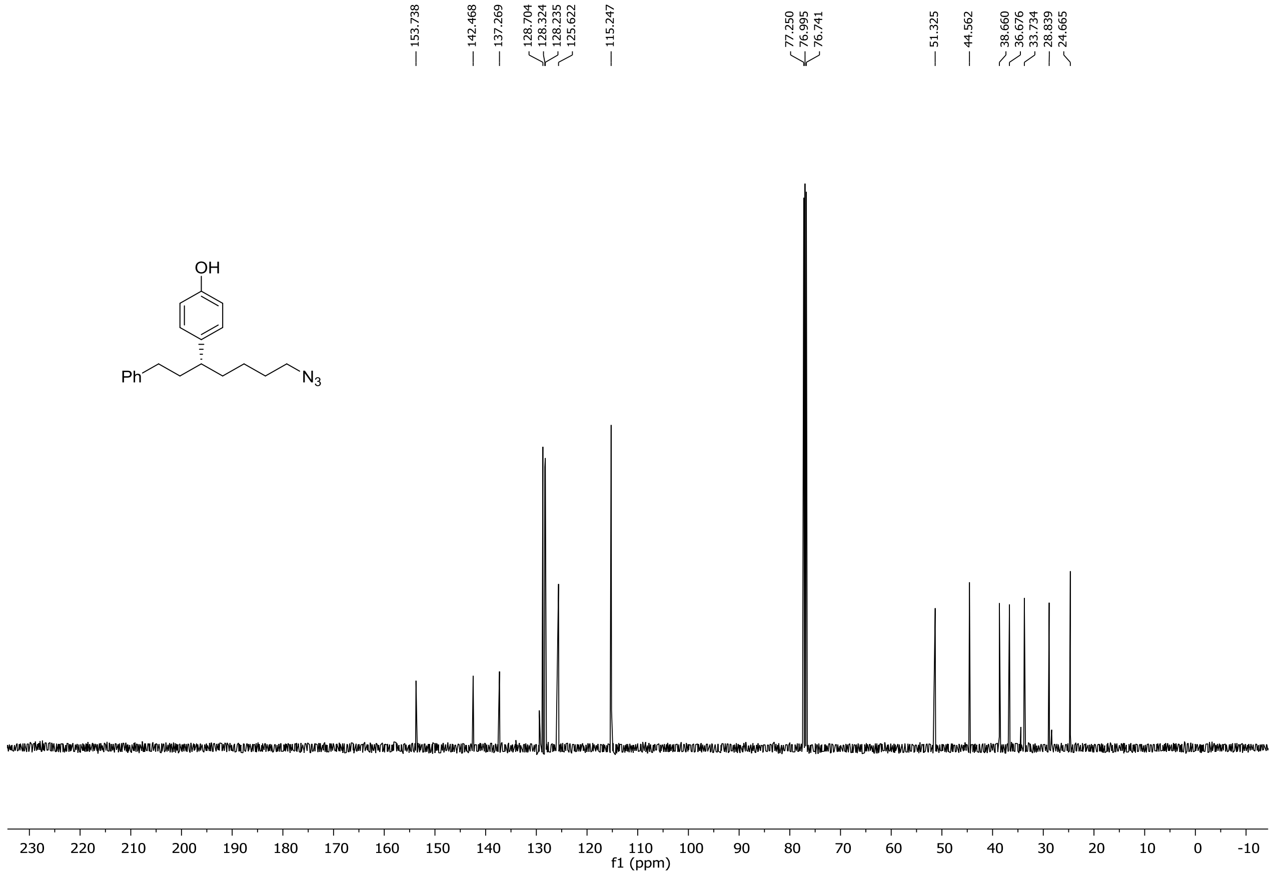
153.738
142.468
137.269
128.704
128.324
128.235
125.622
115.247
77.250
76.995
76.741
51.325
44.562
38.660
36.676
33.734
28.839
24.665
OH
Ph
N3
f1 (ppm)

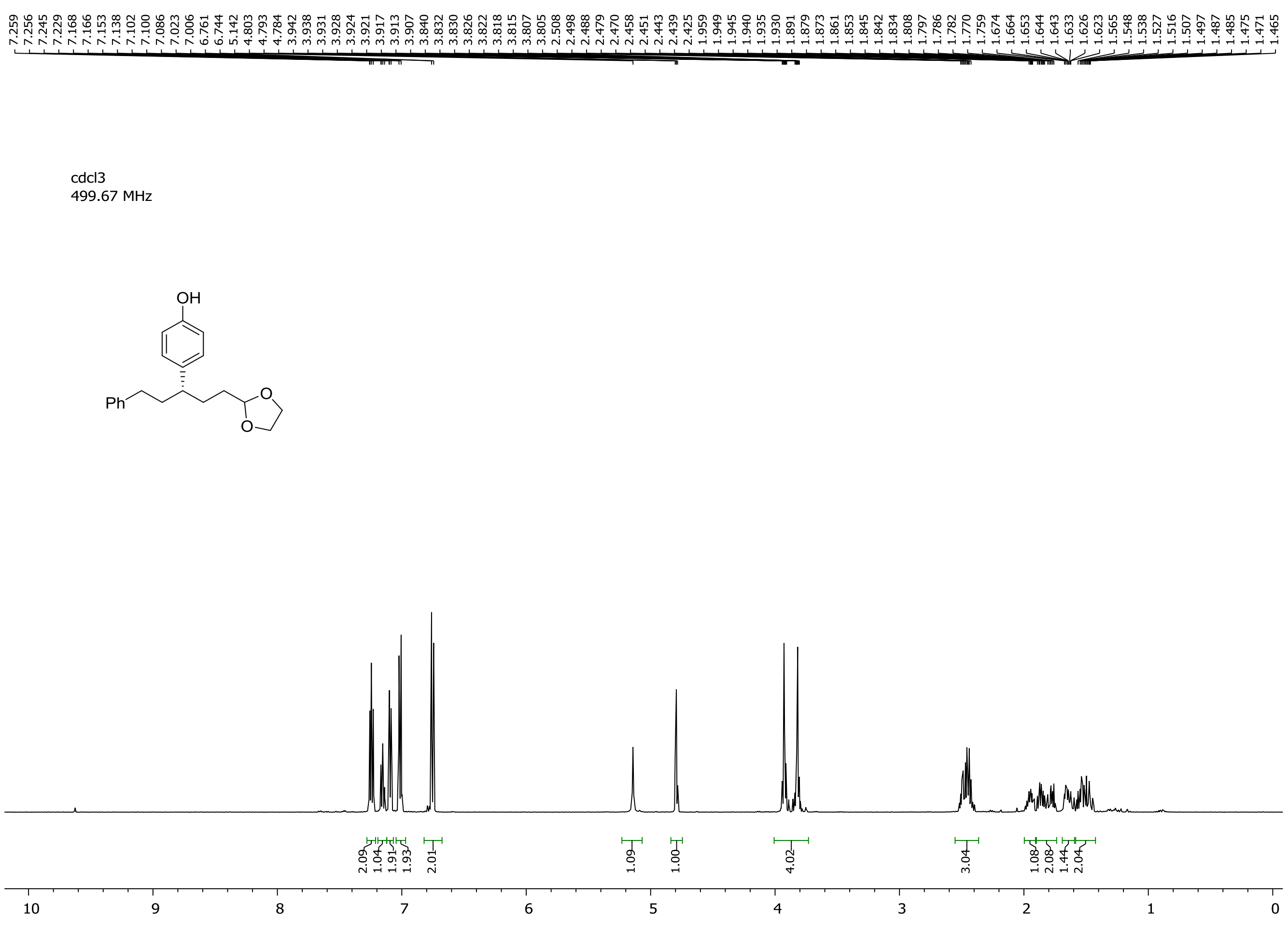
7.259 7.256 7.245 7.229 7.168 7.166 7.153 7.138 7.102 7.100 7.086 7.023 7.006 6.761 6.744 5.142 4.803 4.793 4.784 3.942 3.938 3.931 3.928 3.924 3.921 3.917 3.913 3.907 3.840 3.832 3.830 3.826 3.822 3.818 3.815 3.807 3.805 2.508 2.498 2.488 2.479 2.470 2.458 2.451 2.443 2.439 2.425 1.959 1.949 1.945 1.940 1.935 1.930 1.891 1.879 1.873 1.861 1.853 1.845 1.842 1.834 1.808 1.797 1.786 1.782 1.770 1.759 1.674 1.664 1.653 1.644 1.643 1.633 1.626 1.623 1.565 1.548 1.538 1.527 1.516 1.507 1.497 1.487 1.485 1.475 1.471 1.465
cdcl3
499.67 MHz
OH
Ph
O
O
2.09
1.04
1.91
1.93
2.01
1.09
1.00
4.02
3.04
1.08
2.08
1.44
2.04

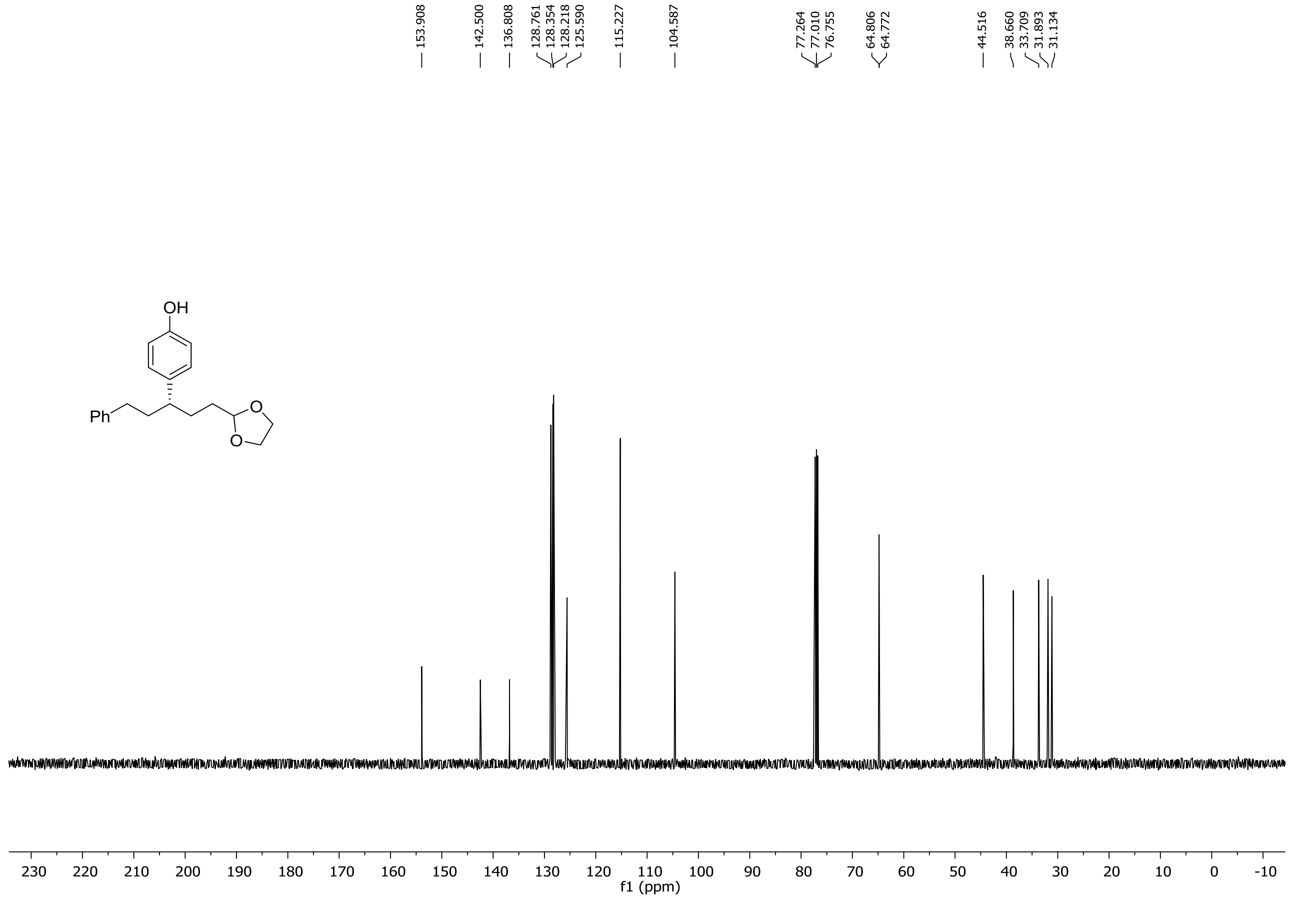
153.908
142.500
136.808
128.761
128.354
128.218
125.590
115.227
104.587
77.264
77.010
76.755
64.806
64.772
44.516
38.660
33.709
31.893
31.134
OH
Ph
O
O
230 220 210 200 190 180 170 160 150 140 130 120 110 100 90 80 70 60 50 40 30 20 10 0 -10
f1 (ppm)

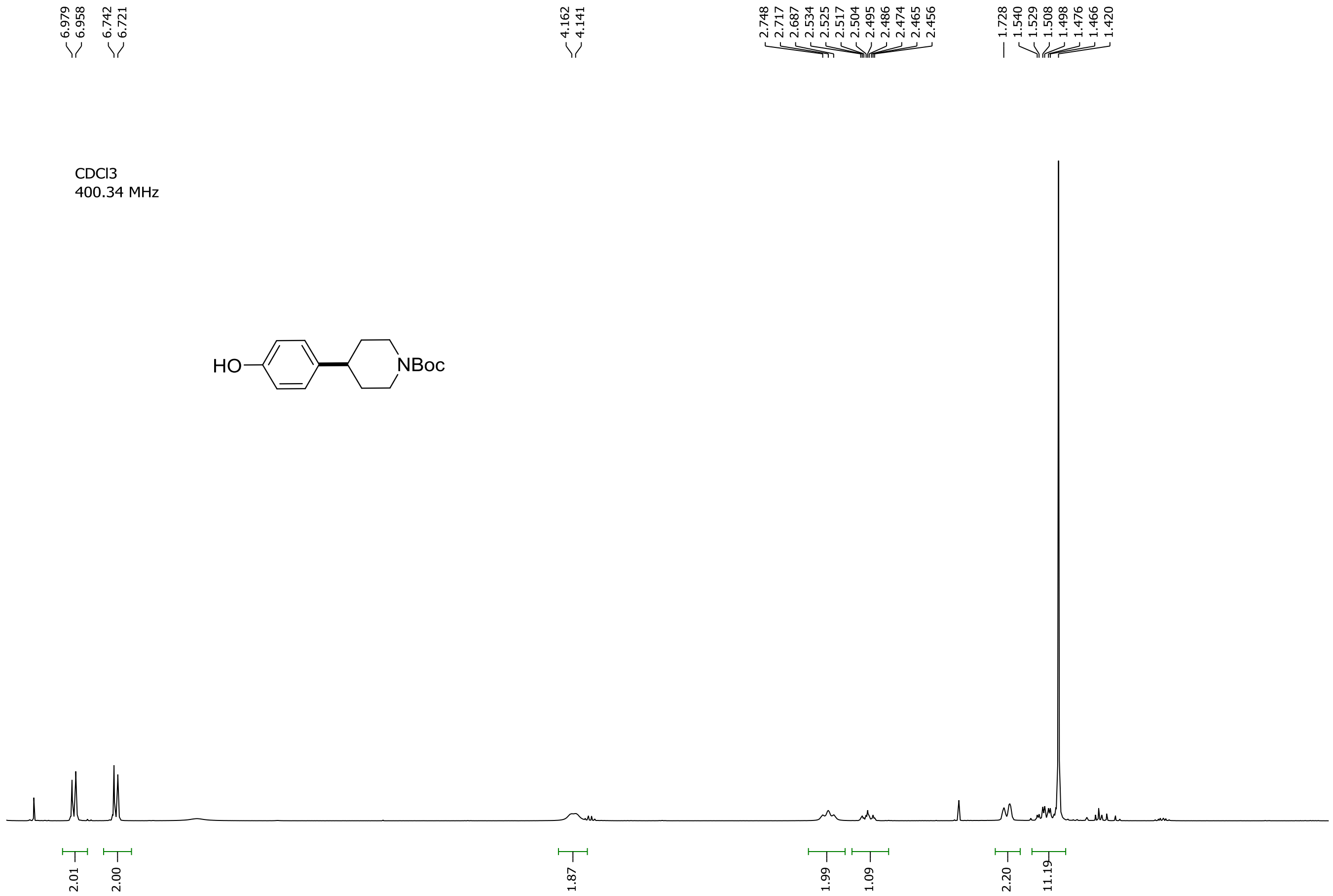
6.979
6.958
6.742
6.721
4.162
4.141
2.748
2.717
2.687
2.534
2.525
2.517
2.504
2.495
2.486
2.474
2.465
2.456
1.728
1.540
1.529
1.508
1.498
1.476
1.466
1.420
CDCl3
400.34 MHz
HO
NBoc
2.01
2.00
1.87
1.99
1.09
2.20
11.19
7 6 5 4 3 2 1 0

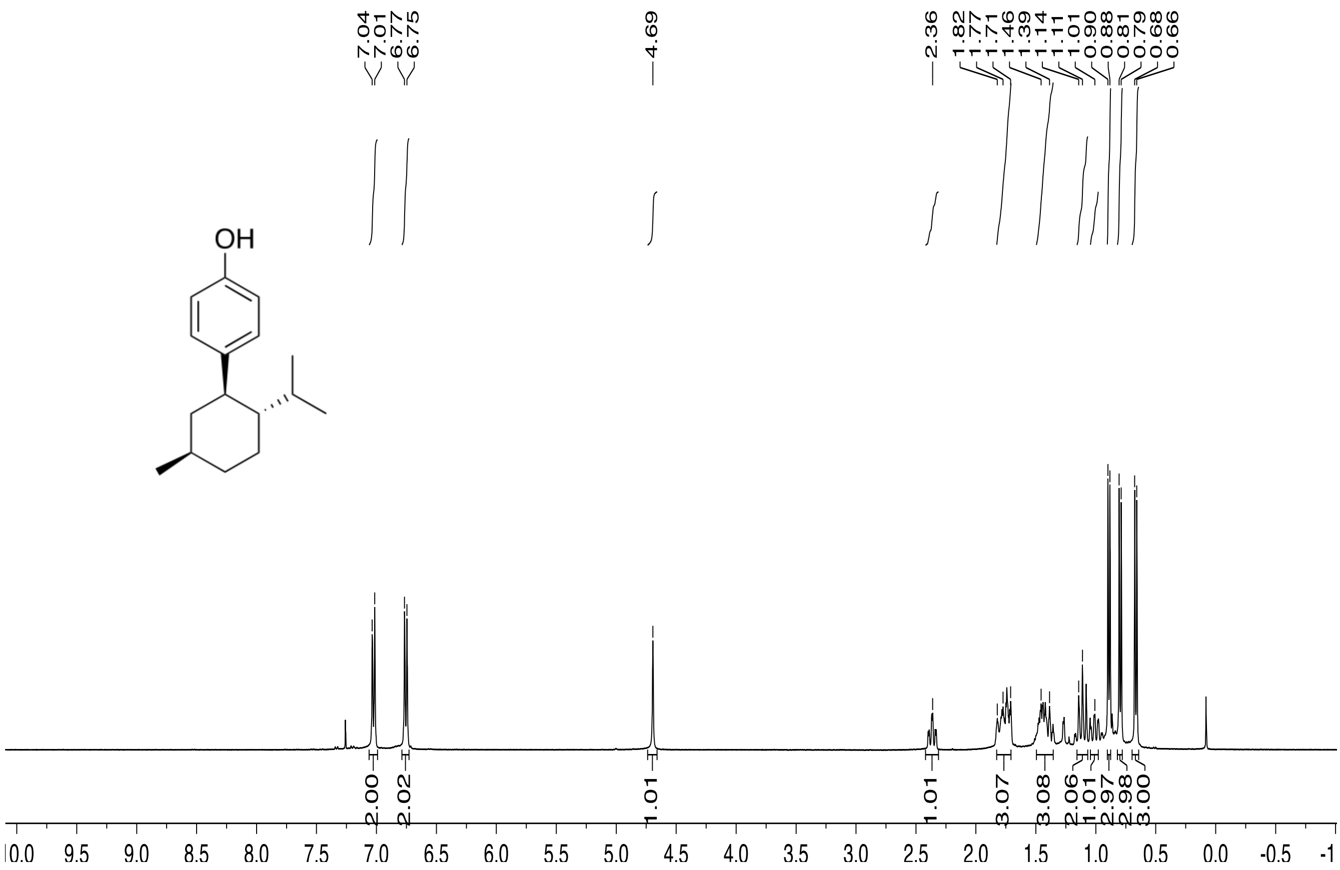
7.04
7.01
6.77
6.75
4.69
2.36
1.82
1.77
1.71
1.46
1.39
1.14
1.11
1.01
0.90
0.88
0.81
0.79
0.68
0.66
OH
2.00
2.02
1.01
1.01
3.07
3.08
2.06
1.01
2.97
2.98
3.00

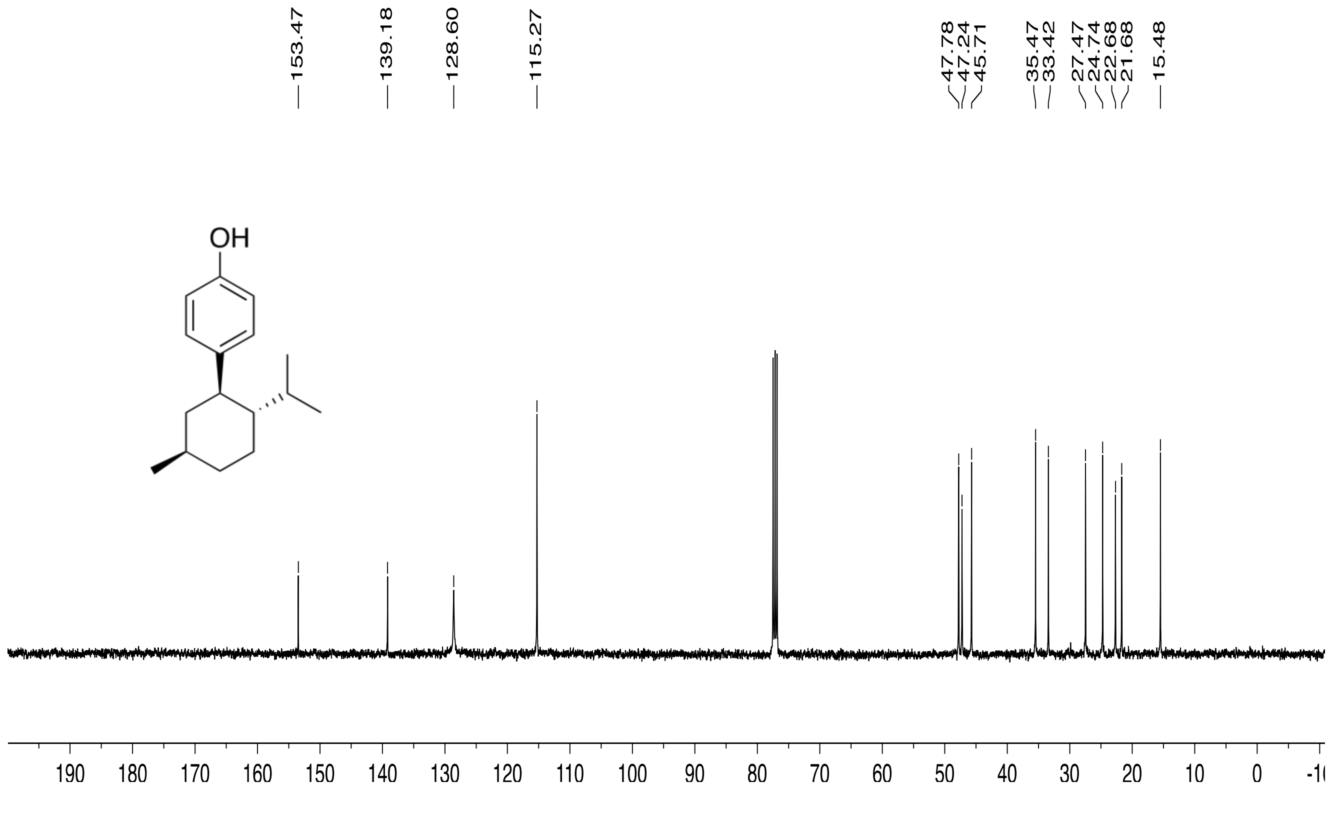
153.47
139.18
128.60
115.27
47.78
47.24
45.71
35.47
33.42
27.47
24.74
22.68
21.68
15.48
OH

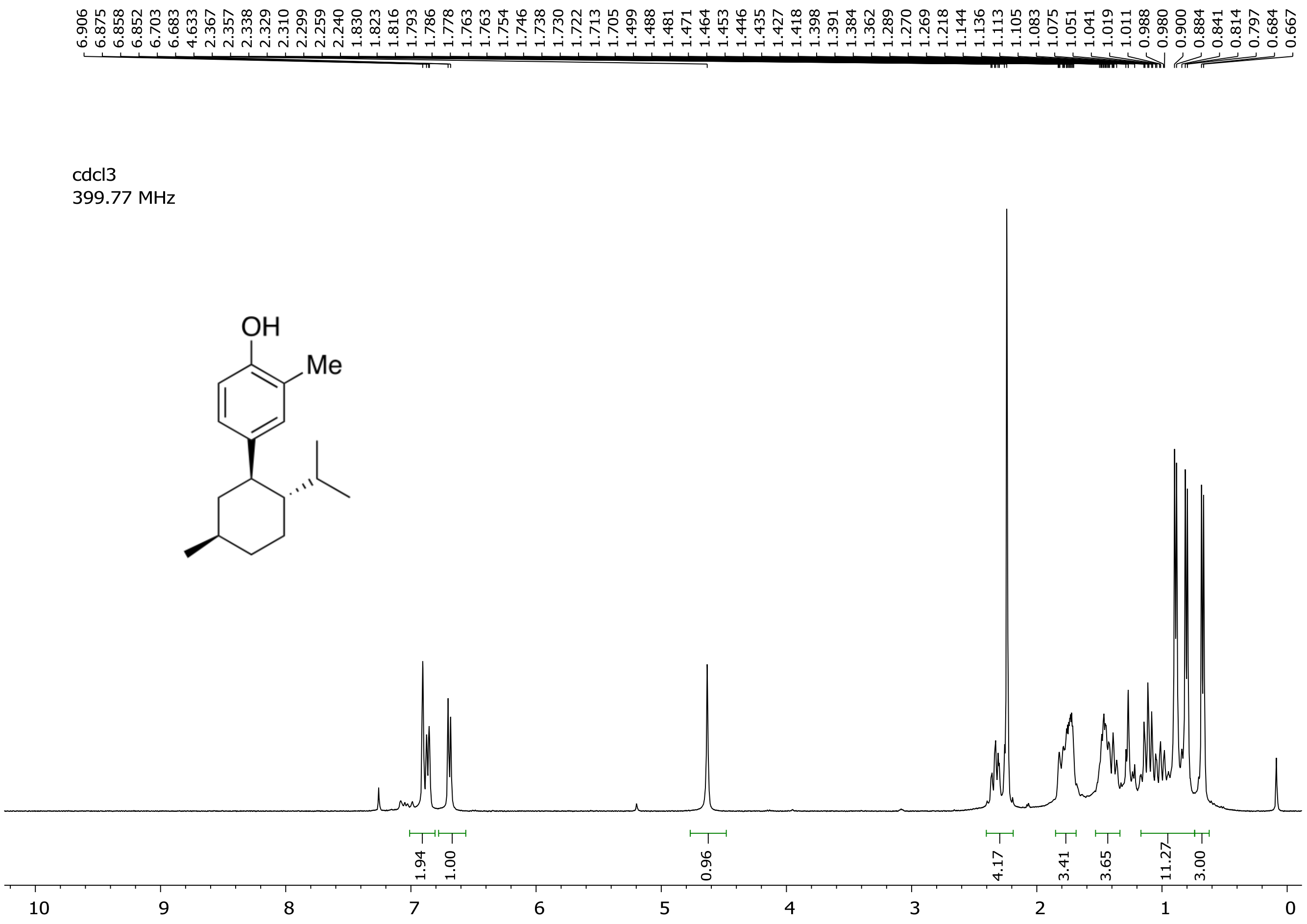
6.906 6.875 6.858 6.852 6.703 6.683 4.633 2.367 2.357 2.338 2.329 2.310 2.299 2.259 2.240 1.830 1.823 1.816 1.793 1.786 1.778 1.763 1.763 1.754 1.746 1.738 1.730 1.722 1.713 1.705 1.499 1.488 1.481 1.471 1.464 1.453 1.446 1.435 1.427 1.418 1.398 1.391 1.384 1.362 1.289 1.270 1.269 1.218 1.144 1.136 1.113 1.105 1.083 1.075 1.051 1.041 1.019 1.011 0.988 0.980 0.900 0.884 0.841 0.814 0.797 0.684 0.667
cdcl3
399.77 MHz
OH
Me
1.94
1.00
0.96
4.17
3.41
3.65
11.27
3.00
10 9 8 7 6 5 4 3 2 1 0

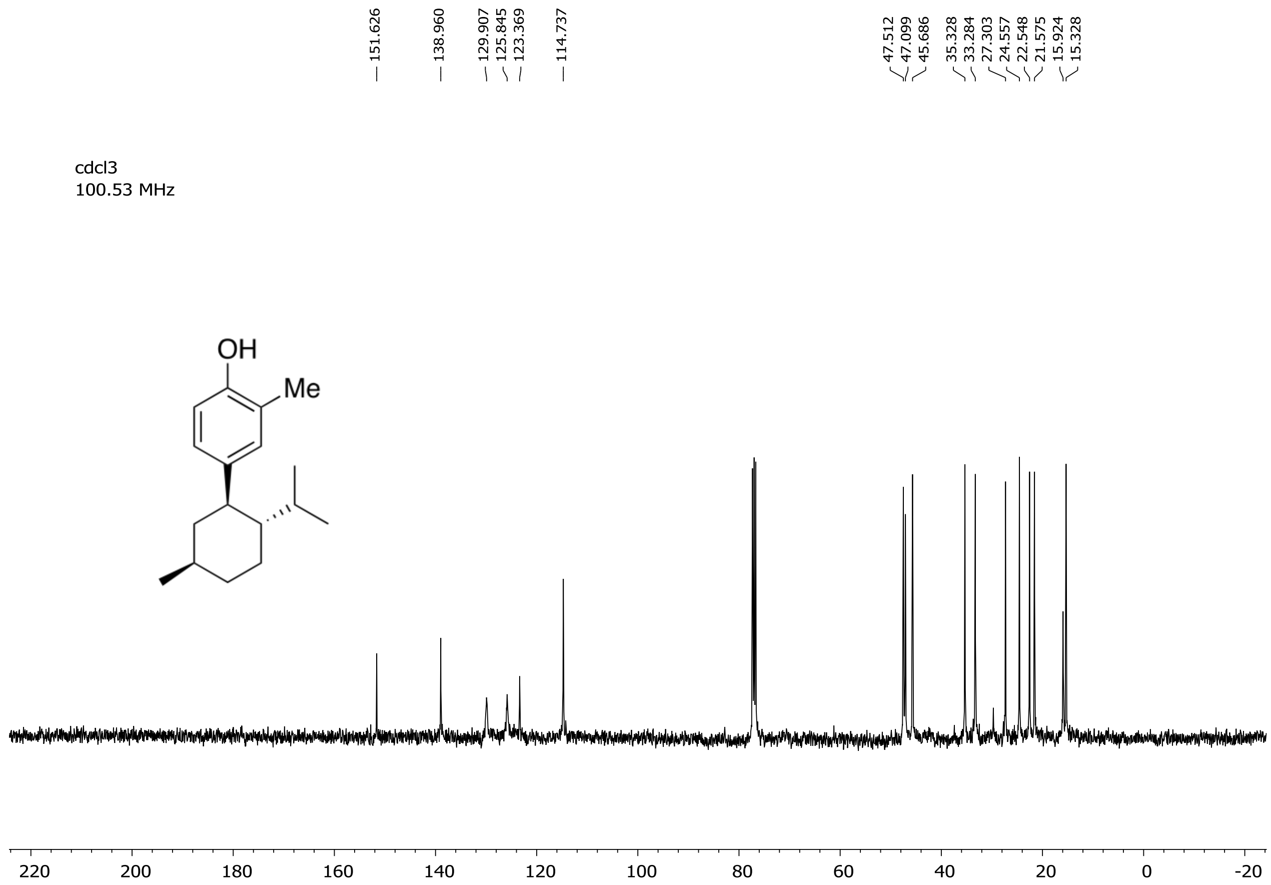
151.626
138.960
129.907
125.845
123.369
114.737
47.512
47.099
45.686
35.328
33.284
27.303
24.557
22.548
21.575
15.924
15.328
cdcl3
100.53 MHz
OH
Me
220 200 180 160 140 120 100 80 60 40 20 0 -20

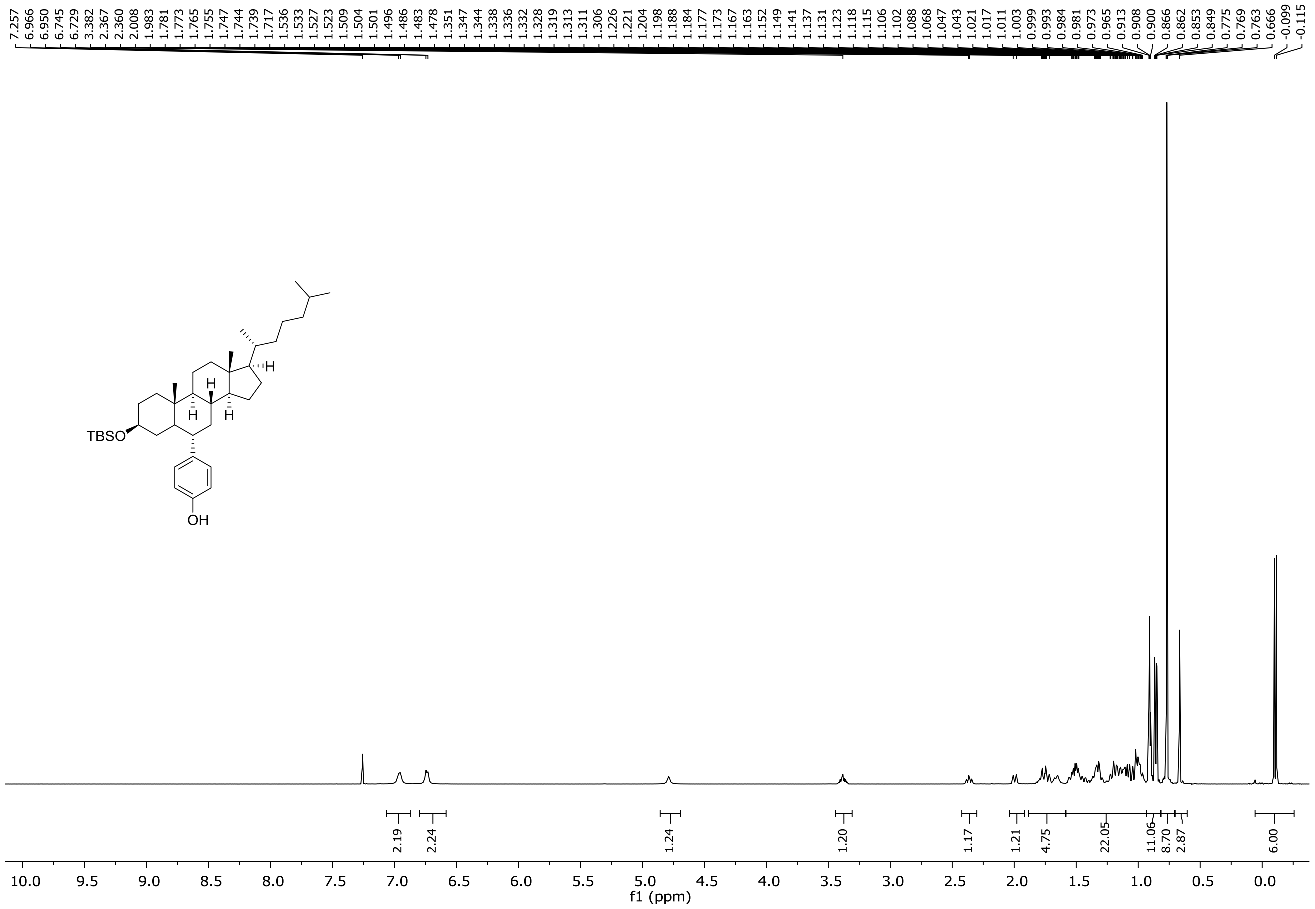
7.257 6.966 6.950 6.745 6.729 3.382 2.367 2.360 2.008 1.983 1.781 1.773 1.765 1.755 1.747 1.744 1.739 1.717 1.536 1.533 1.527 1.523 1.509 1.504 1.501 1.496 1.486 1.483 1.478 1.351 1.347 1.344 1.338 1.336 1.332 1.328 1.319 1.313 1.311 1.306 1.226 1.221 1.204 1.198 1.188 1.184 1.177 1.173 1.167 1.163 1.152 1.149 1.141 1.137 1.131 1.123 1.118 1.115 1.106 1.102 1.088 1.068 1.047 1.043 1.021 1.017 1.011 1.003 0.999 0.993 0.984 0.981 0.973 0.965 0.913 0.908 0.900 0.866 0.862 0.853 0.849 0.775 0.769 0.763 0.666 -0.099 -0.115
TBSO
OH
H
2.19 2.24 1.24 1.20 1.17 1.21 4.75 22.05 11.06 8.70 2.87 6.00
10.0 9.5 9.0 8.5 8.0 7.5 7.0 6.5 6.0 5.5 5.0 4.5 4.0 3.5 3.0 2.5 2.0 1.5 1.0 0.5 0.0
f1 (ppm)

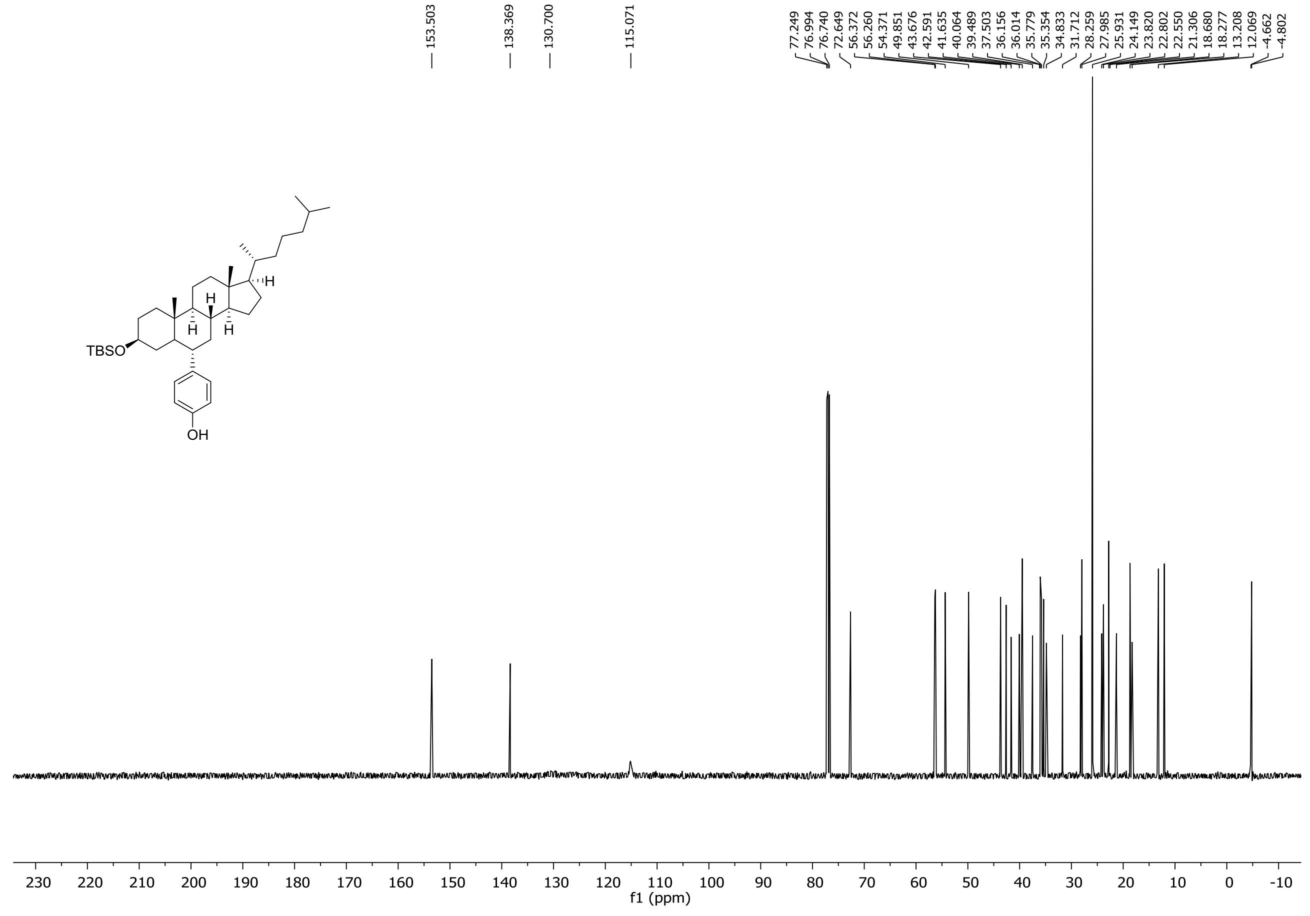
153.503 138.369 130.700 115.071
77.249 76.994 76.740 72.649 56.372 56.260 54.371 49.851 43.676 42.591 41.635 40.064 39.489 37.503 36.156 36.014 35.779 35.354 34.833 31.712 28.259 27.985 25.931 24.149 23.820 22.802 22.550 21.306 18.680 18.277 13.208 12.069 -4.662 -4.802
TBSO
OH
H
230 220 210 200 190 180 170 160 150 140 130 120 110 100 90 80 70 60 50 40 30 20 10 0 -10
f1 (ppm)

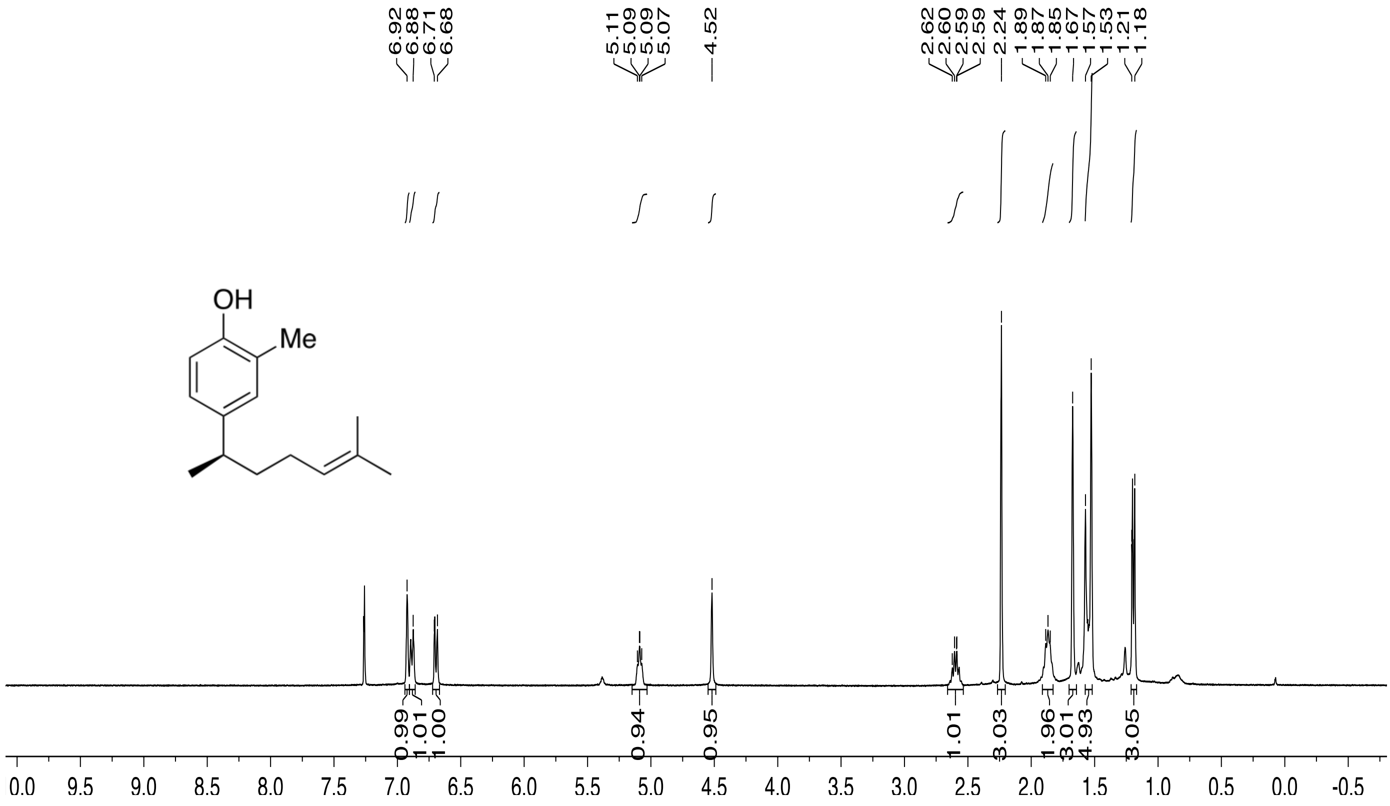

6.92
6.88
6.71
6.68
5.11
5.09
5.09
5.07
4.52
2.62
2.60
2.59
2.59
2.24
1.89
1.87
1.85
1.67
1.57
1.53
1.21
1.18
OH
Me
0.99
1.01
1.00
0.94
0.95
1.01
3.03
1.96
3.01
4.93
3.05

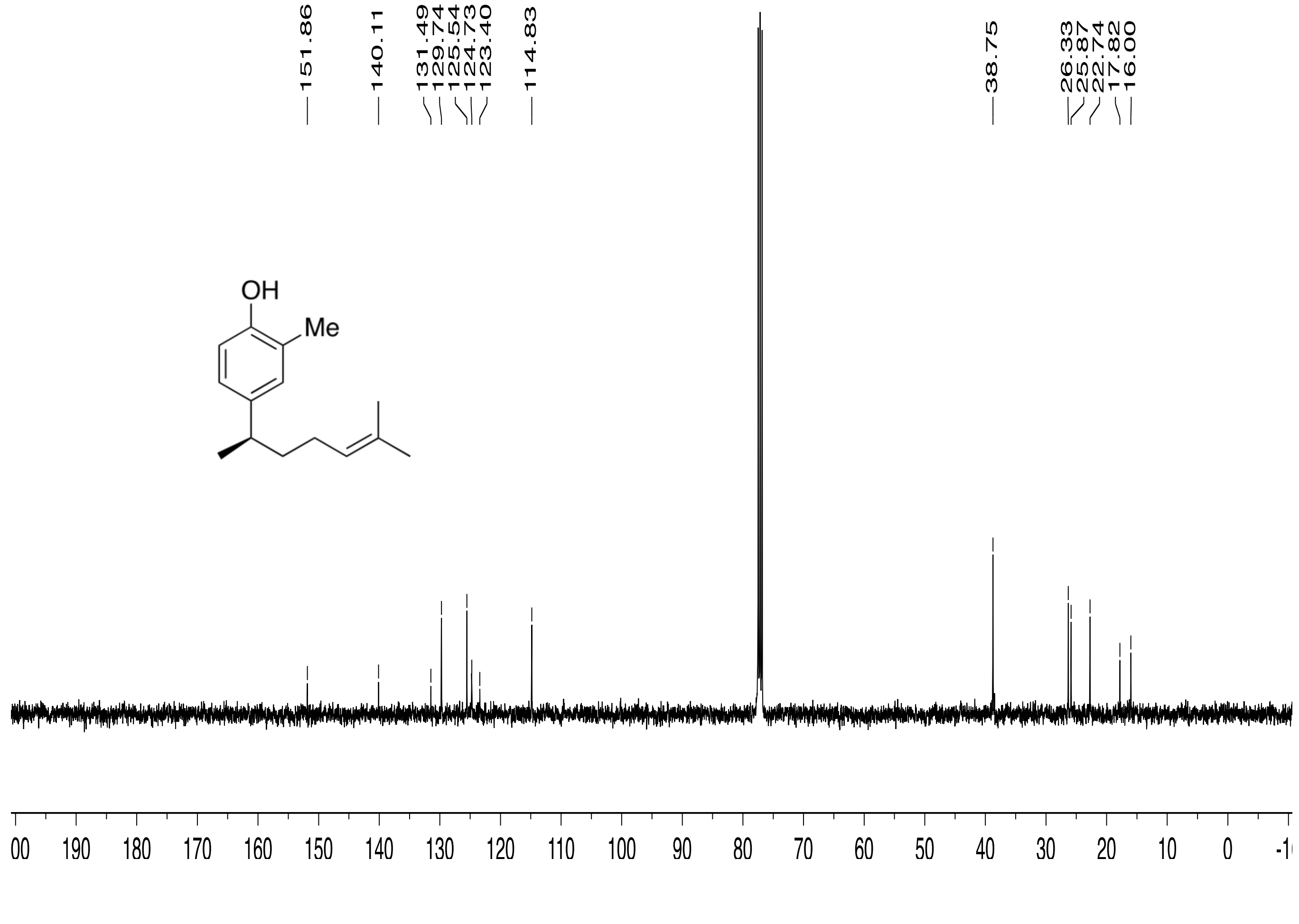

151.86
140.11
131.49
129.74
125.54
124.73
123.40
114.83
38.75
26.33
25.87
22.74
17.82
16.00
OH
Me

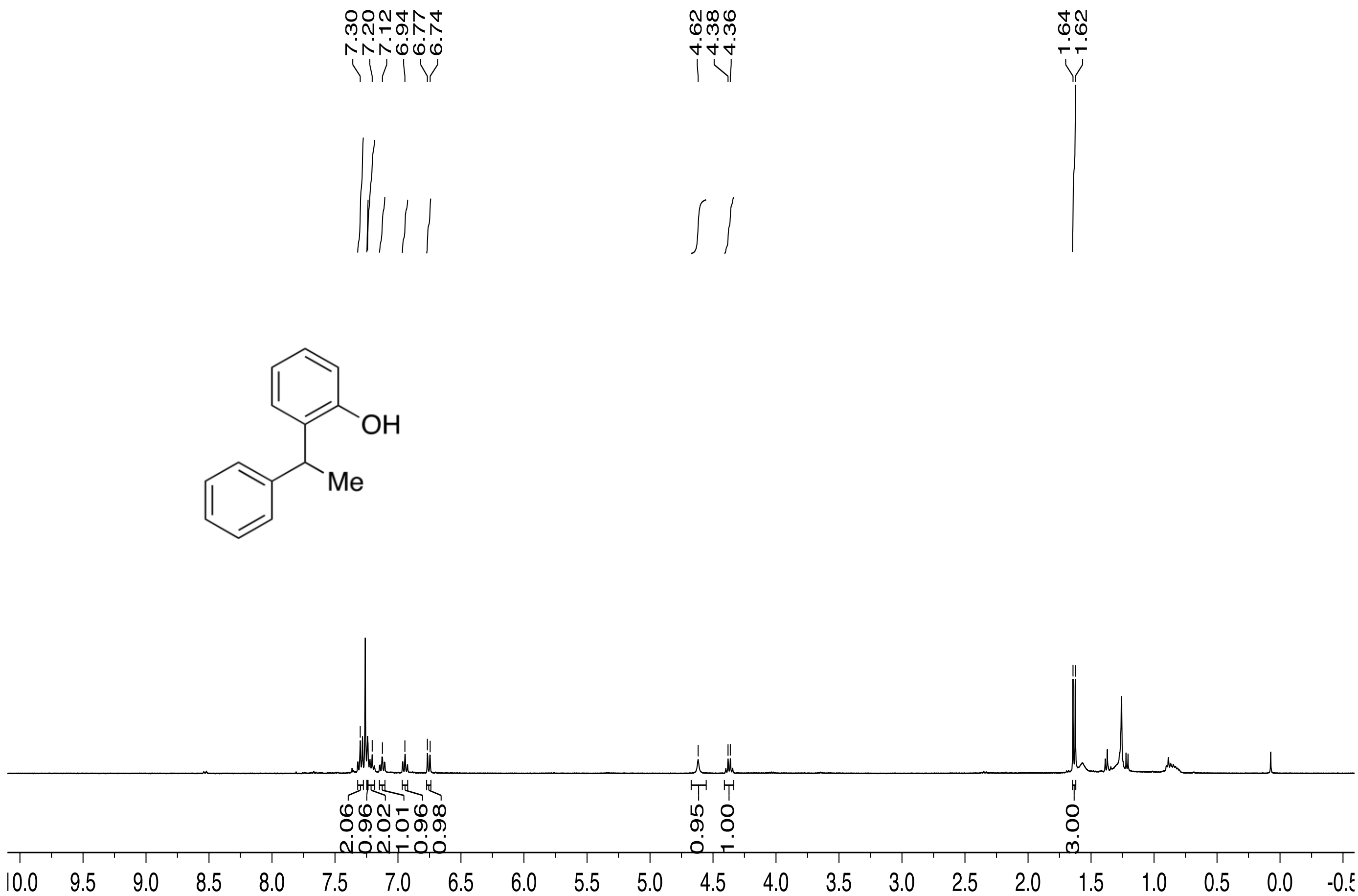
7.30
7.20
7.12
6.94
6.77
6.74
4.62
4.38
4.36
1.64
1.62
OH
Me
2.06
0.96
2.02
1.01
0.96
0.98
0.95
1.00
3.00

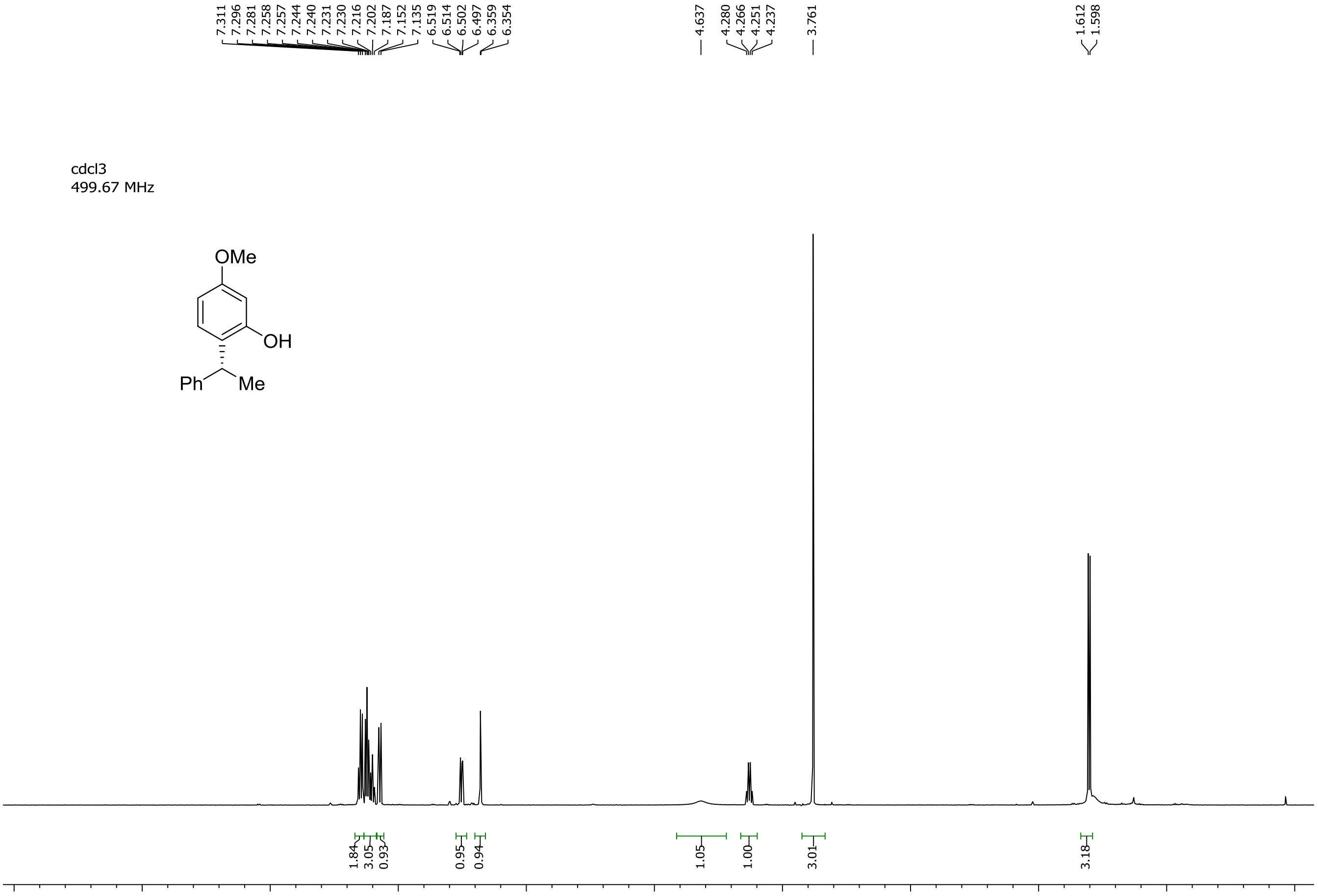
7.311
7.296
7.281
7.258
7.257
7.244
7.240
7.231
7.230
7.216
7.202
7.187
7.152
7.135
6.519
6.514
6.502
6.497
6.359
6.354
4.637
4.280
4.266
4.251
4.237
3.761
1.612
1.598
cdcl3
499.67 MHz
OMe
OH
Ph
Me
1.84
3.05
0.93
0.95
0.94
1.05
1.00
3.01
3.18

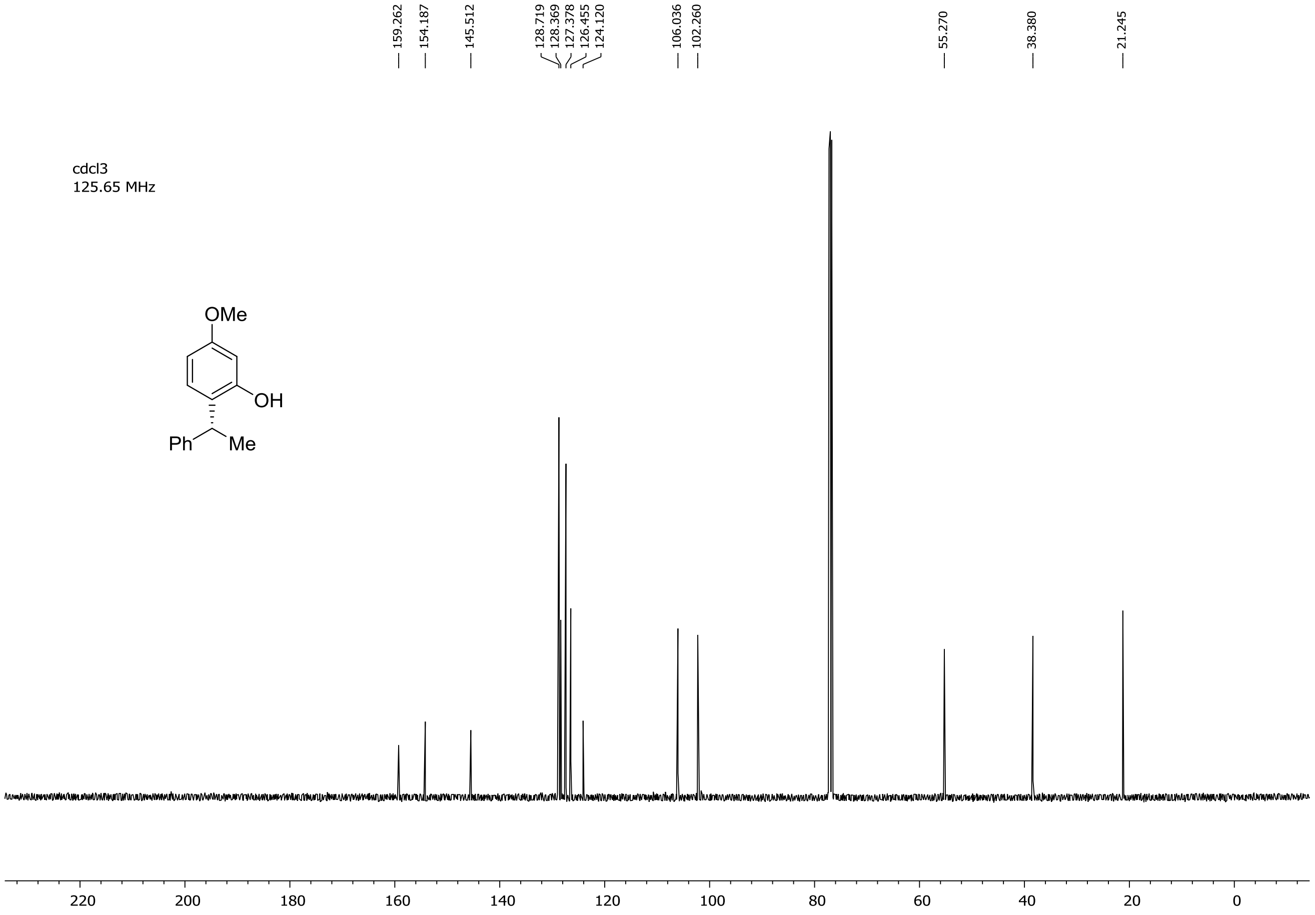
159.262
154.187
145.512
128.719
128.369
127.378
126.455
124.120
106.036
102.260
55.270
38.380
21.245
cdcl3
125.65 MHz
OMe
OH
Ph
Me
220
200
180
160
140
120
100
80
60
40
20
0

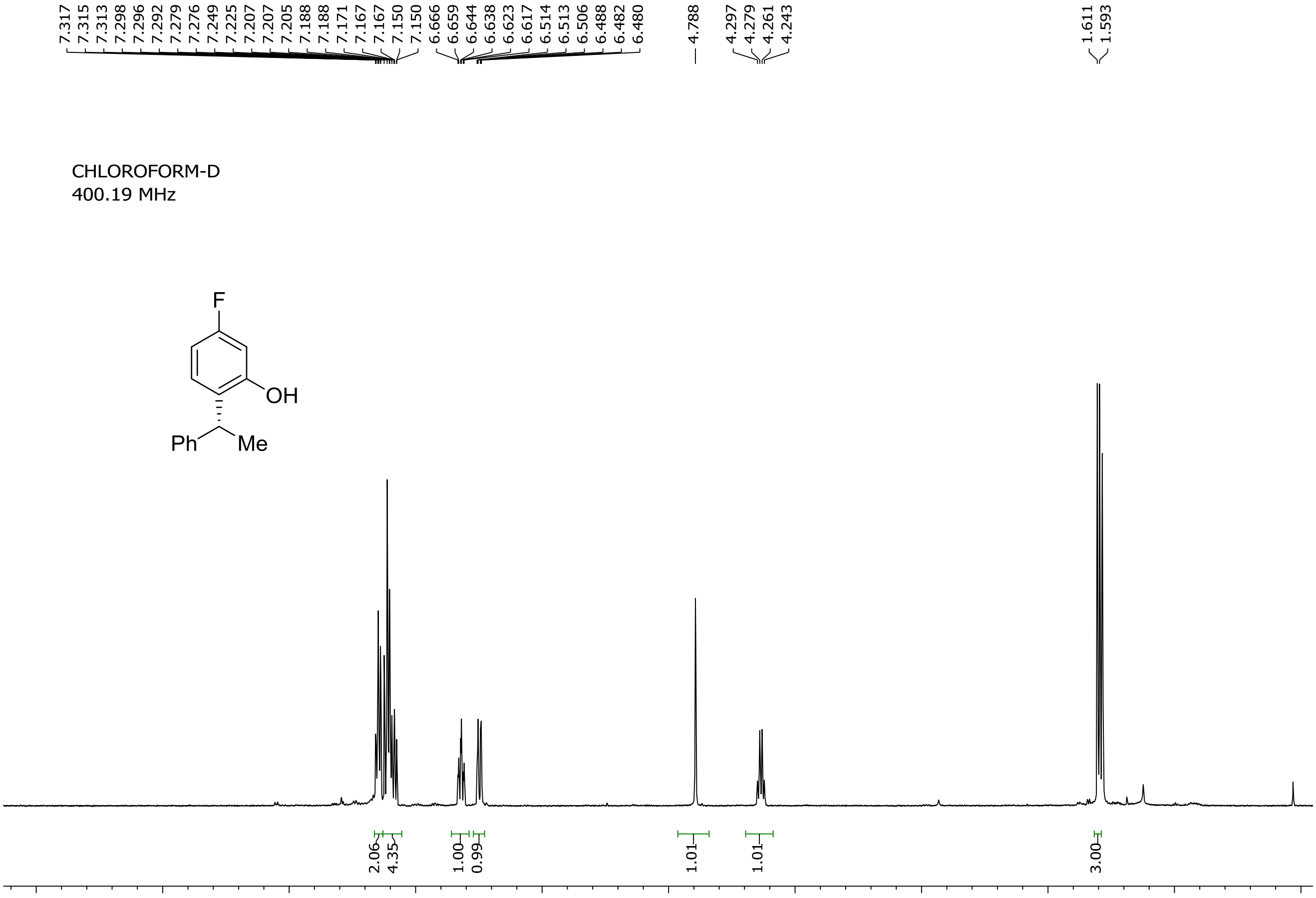
7.317
7.315
7.313
7.298
7.296
7.292
7.279
7.276
7.249
7.225
7.207
7.207
7.205
7.188
7.188
7.171
7.167
7.167
7.150
7.150
6.666
6.659
6.644
6.638
6.623
6.617
6.514
6.513
6.506
6.488
6.482
6.480
4.788
4.297
4.279
4.261
4.243
1.611
1.593
CHLOROFORM-D
400.19 MHz
F
OH
Ph
Me
2.06
4.35
1.00
0.99
1.01
1.01
3.00
10
9
8
7
6
5
4
3
2
1
0

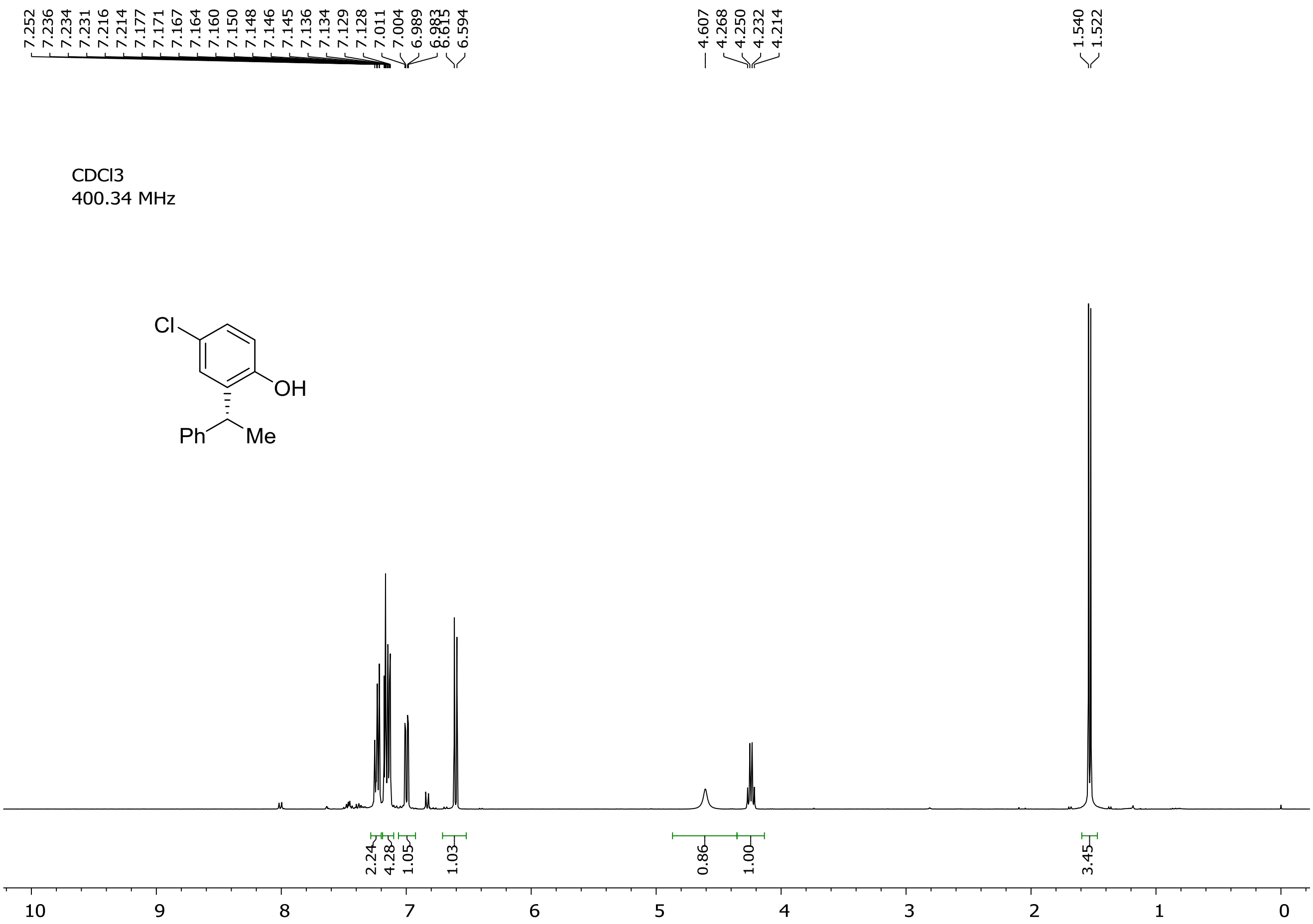
CDCl3
400.34 MHz
Cl
OH
Ph
Me

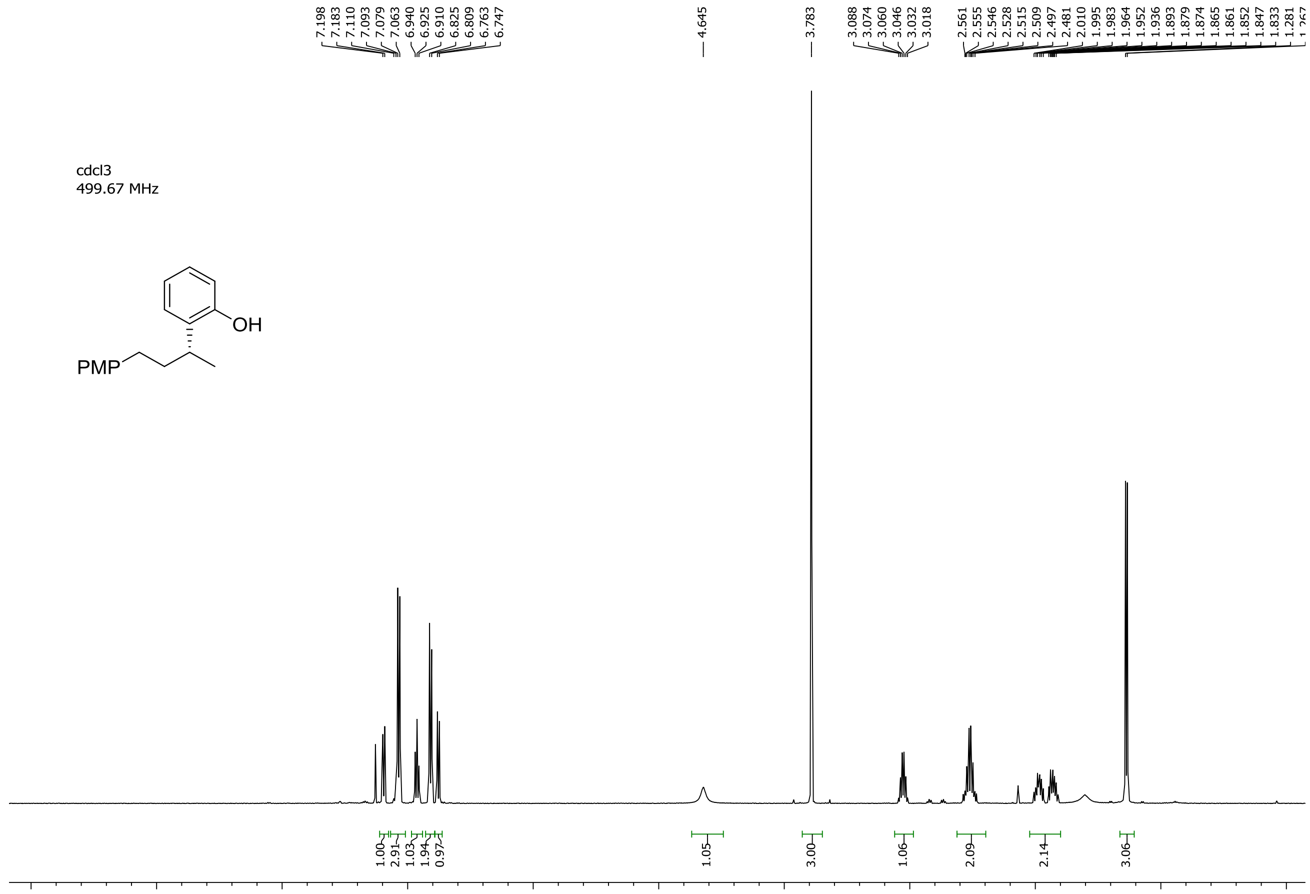
cdcl3
499.67 MHz
OH
PMP

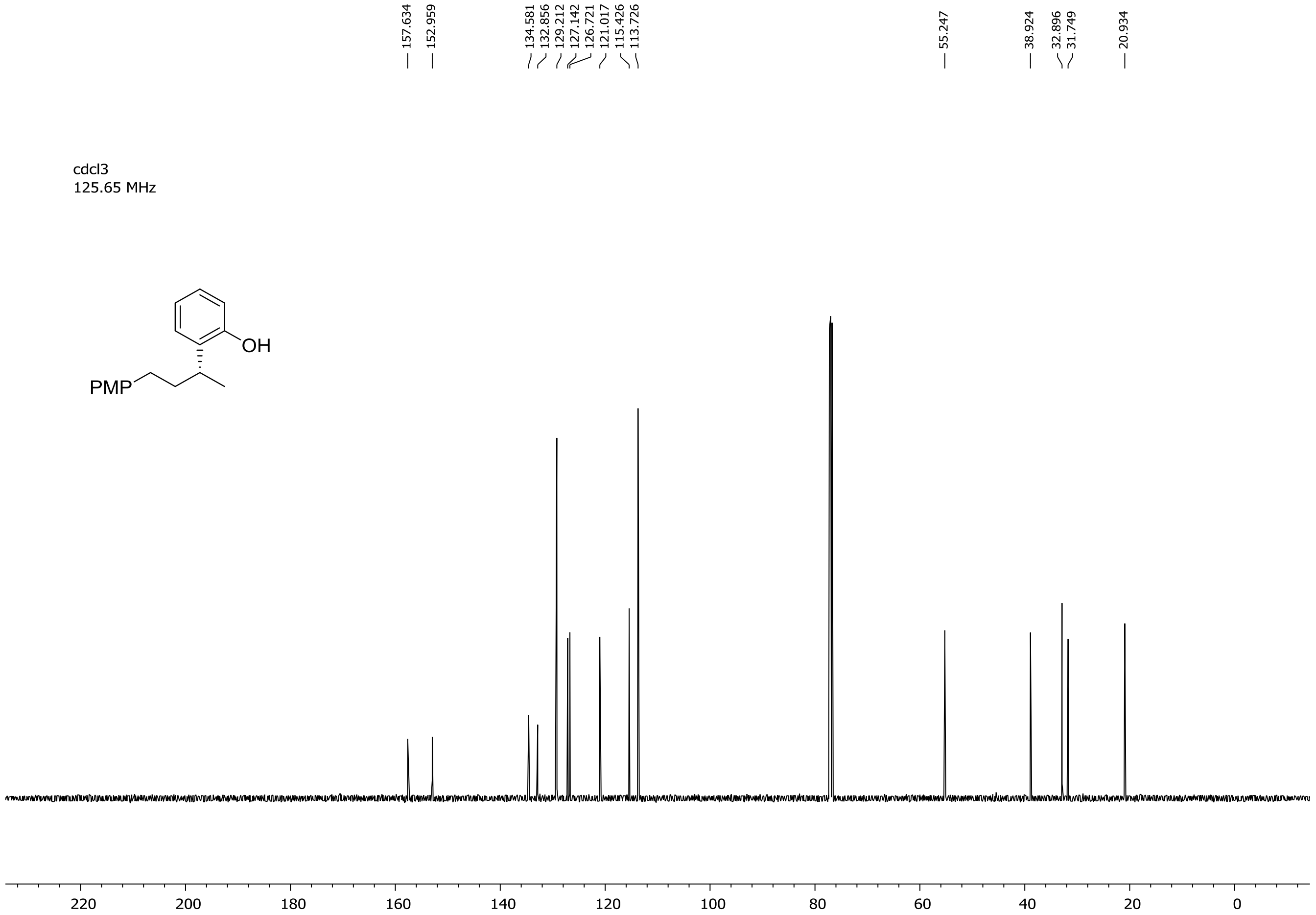
157.634
152.959
134.581
132.856
129.212
127.142
126.721
121.017
115.426
113.726
55.247
38.924
32.896
31.749
20.934
cdcl3
125.65 MHz
OH
PMP

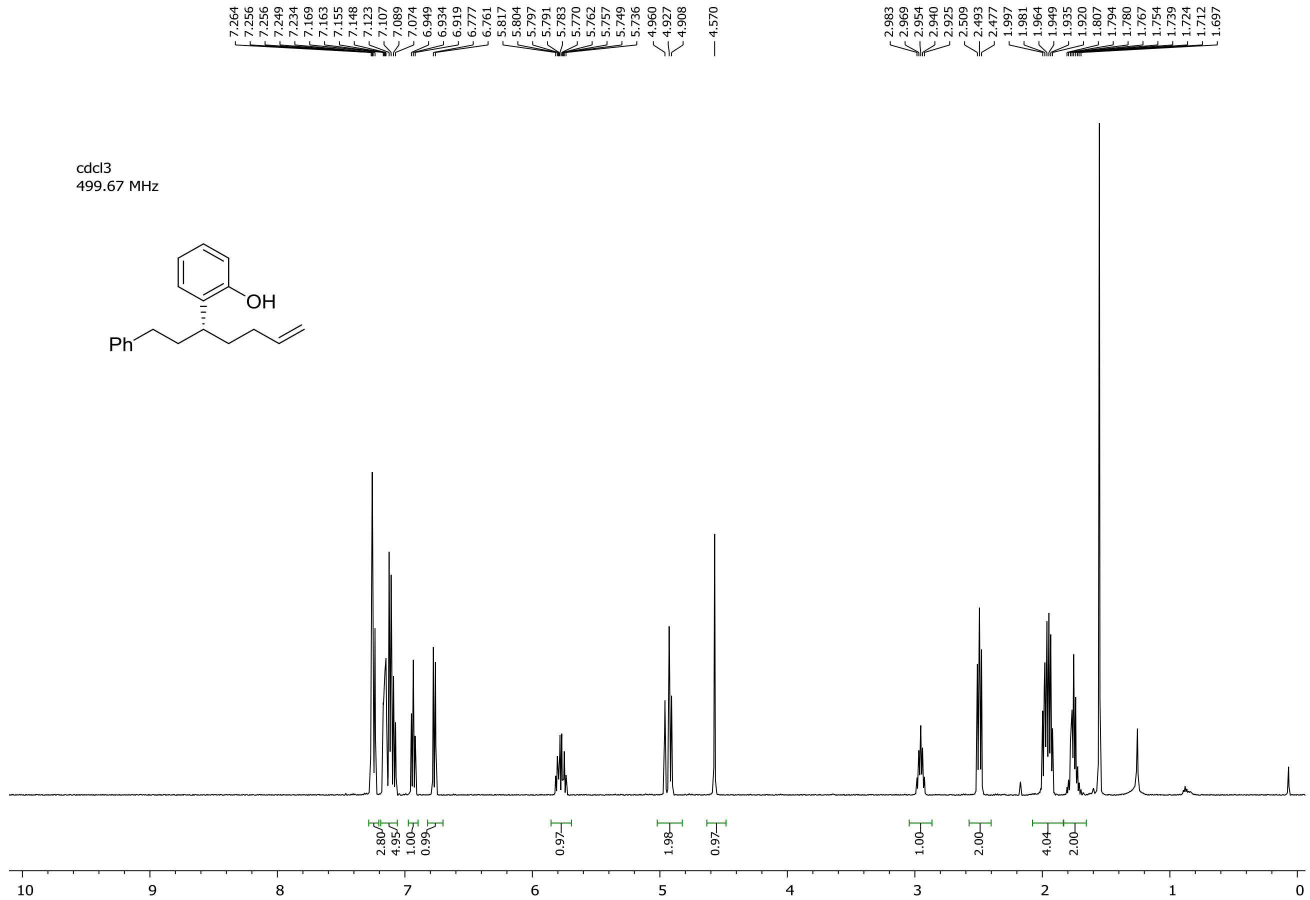
7.264
7.256
7.256
7.249
7.234
7.169
7.163
7.155
7.148
7.123
7.107
7.089
7.074
6.949
6.934
6.919
6.777
6.761
5.817
5.804
5.797
5.791
5.783
5.770
5.762
5.757
5.749
5.736
4.960
4.927
4.908
4.570
2.983
2.969
2.954
2.940
2.925
2.509
2.493
2.477
1.997
1.981
1.964
1.949
1.935
1.920
1.807
1.794
1.780
1.767
1.754
1.739
1.724
1.712
1.697
cdcl3
499.67 MHz
OH
Ph

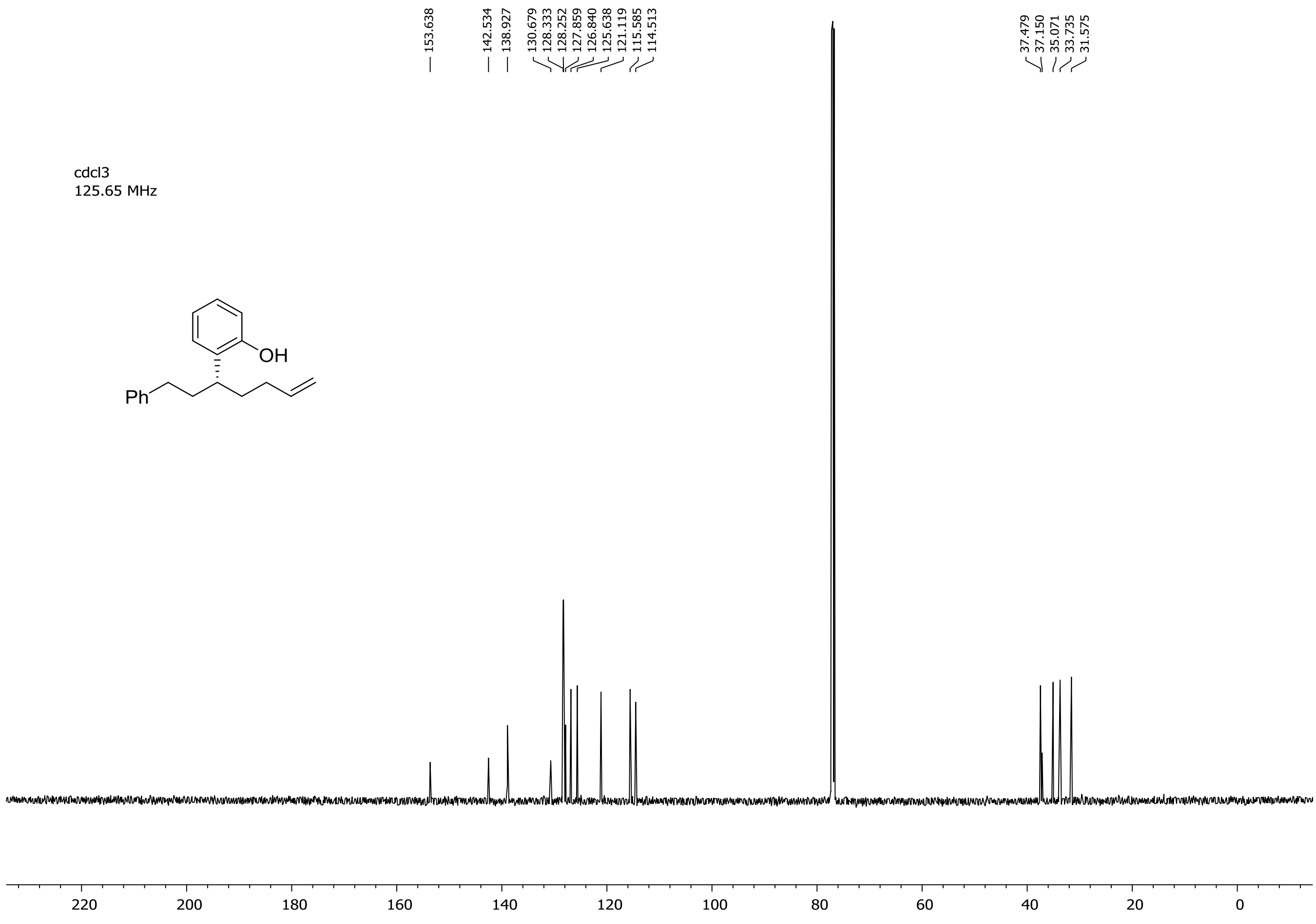

cdcl3
125.65 MHz
OH
Ph
153.638
142.534
138.927
130.679
128.333
128.252
127.859
126.840
125.638
121.119
115.585
114.513
37.479
37.150
35.071
33.735
31.575
220
200
180
160
140
120
100
80
60
40
20
0

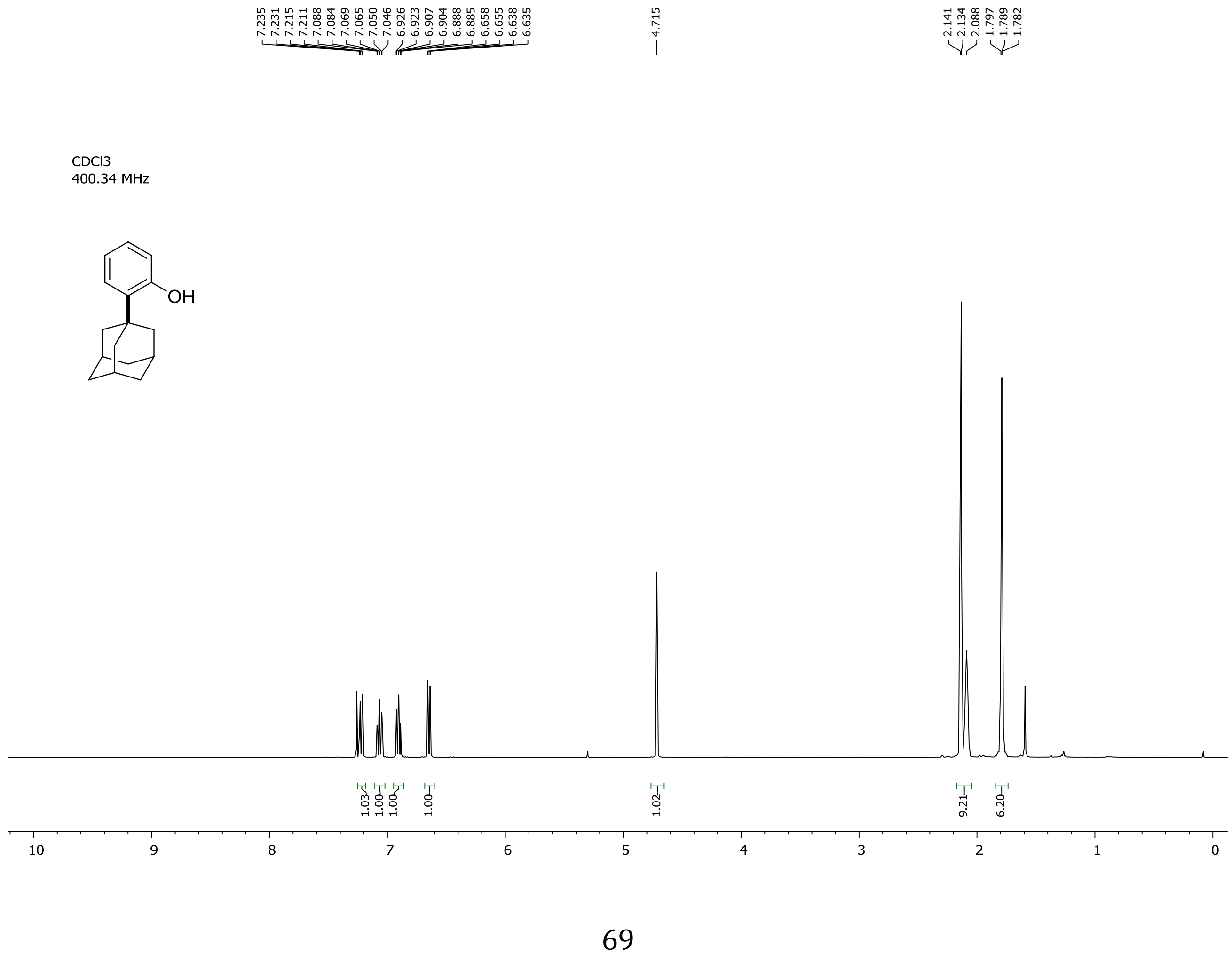

CDCl3
400.34 MHz
OH
7.235
7.231
7.215
7.211
7.088
7.084
7.069
7.065
7.050
7.046
6.926
6.923
6.907
6.904
6.888
6.885
6.655
6.638
6.635
4.715
2.141
2.134
2.088
1.797
1.789
1.782
1.03
1.00
1.00
1.00
1.02
9.21
6.20
10
9
8
7
6
5
4
3
2
1
0

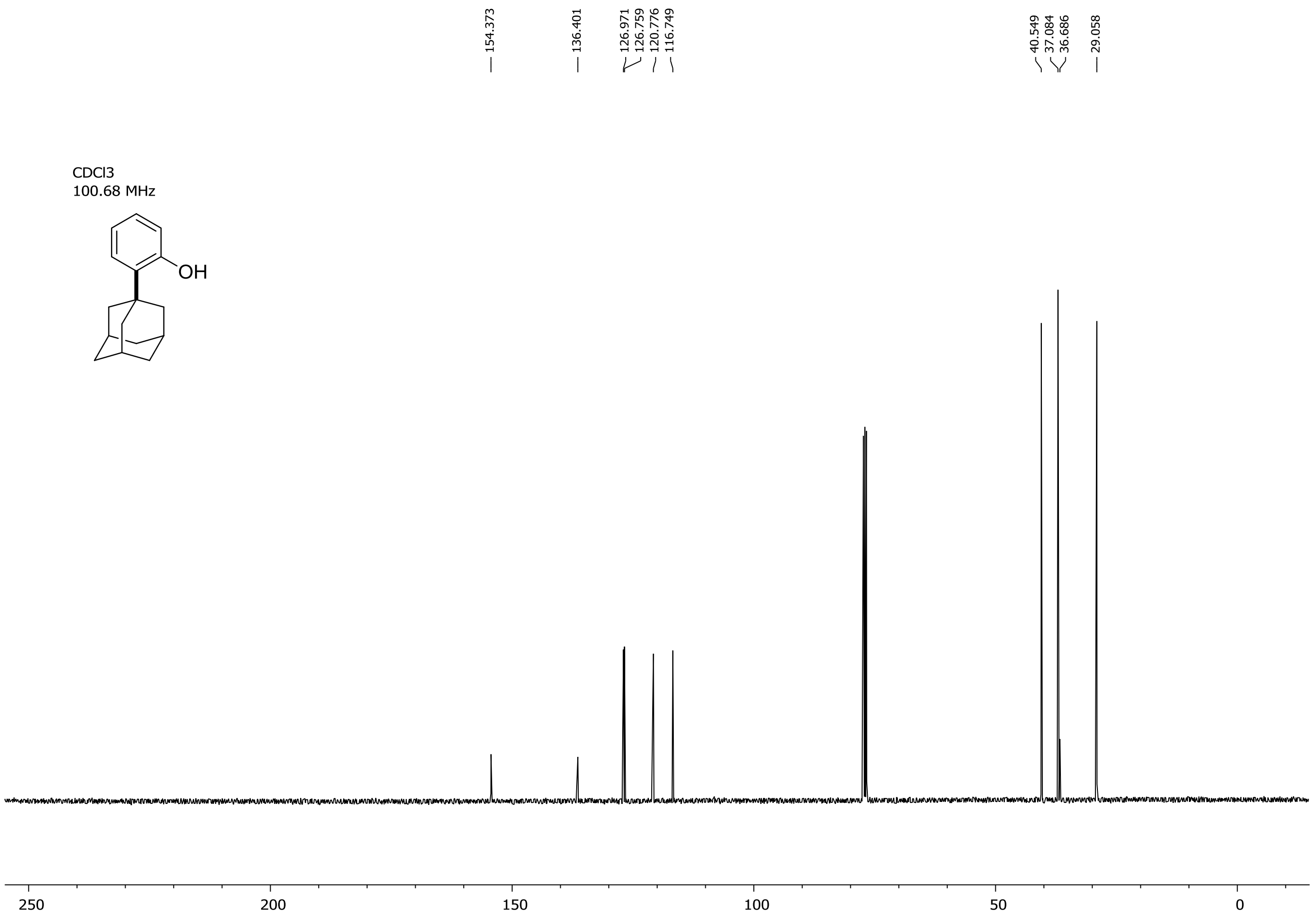
154.373
136.401
126.971
126.759
120.776
116.749
40.549
37.084
36.686
29.058
CDCl3
100.68 MHz
OH
250 200 150 100 50 0

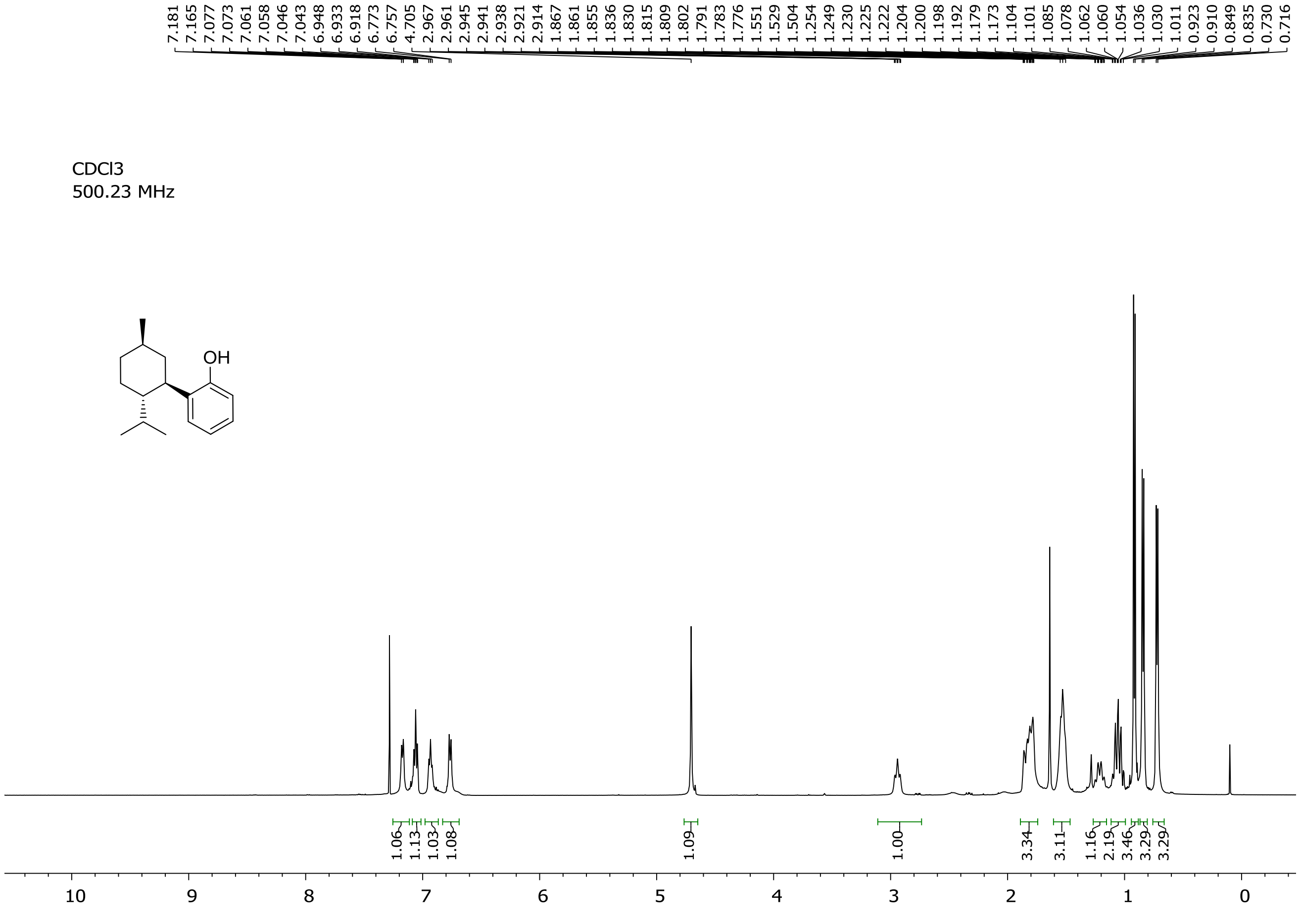
7.181 7.165 7.077 7.073 7.061 7.058 7.046 7.043 6.948 6.933 6.918 6.773 6.757 4.705 2.967 2.961 2.945 2.941 2.938 2.921 2.914 1.867 1.861 1.855 1.836 1.830 1.815 1.809 1.802 1.791 1.783 1.776 1.551 1.529 1.504 1.254 1.249 1.230 1.225 1.222 1.204 1.200 1.198 1.192 1.179 1.173 1.104 1.101 1.085 1.078 1.062 1.060 1.054 1.036 1.030 1.011 0.923 0.910 0.849 0.835 0.730 0.716
CDCl3
500.23 MHz
OH
1.06 1.13 1.03 1.08 1.09 1.00 3.34 3.11 1.16 2.19 3.46 3.29 3.29
10 9 8 7 6 5 4 3 2 1 0

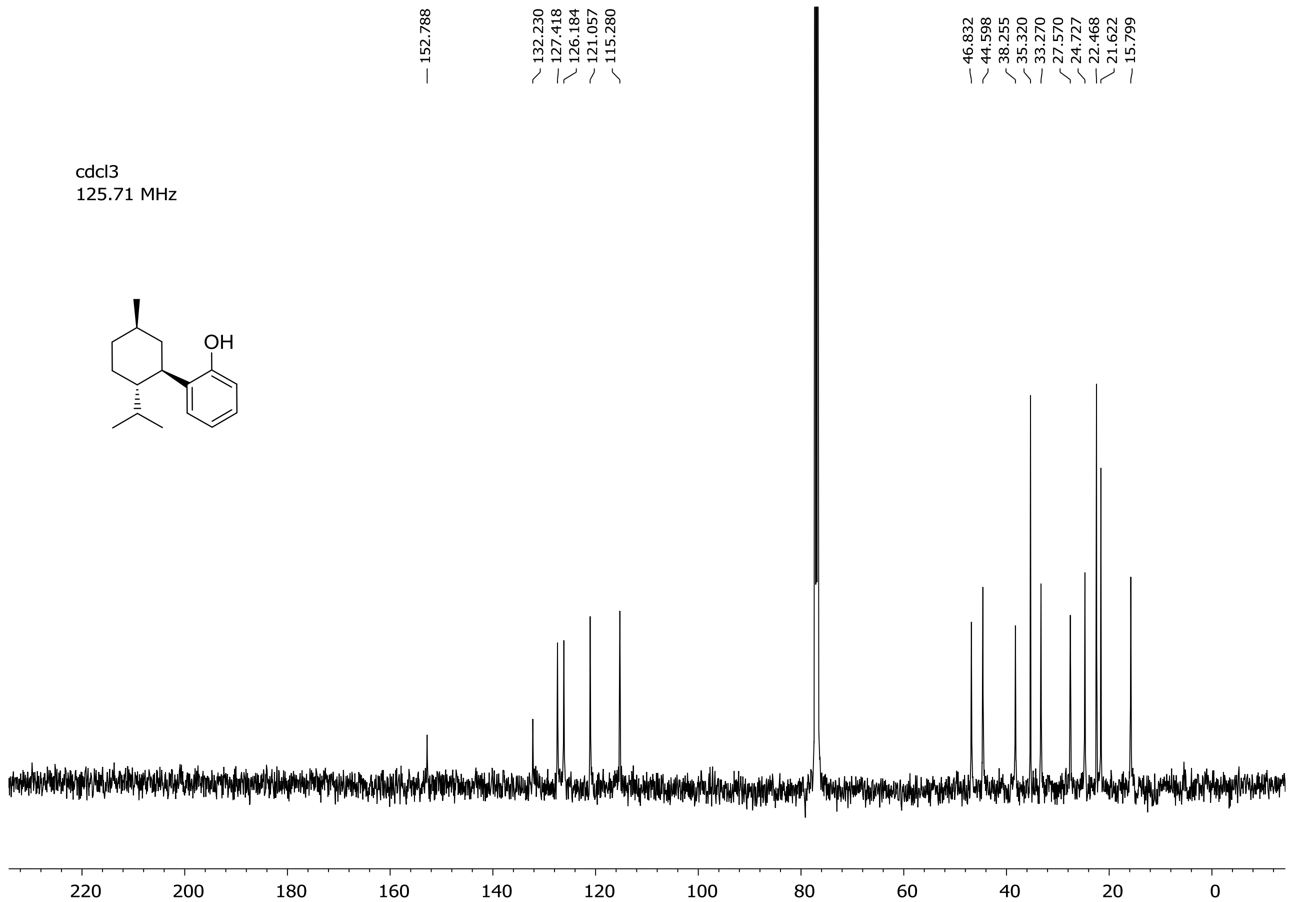
152.788
132.230
127.418
126.184
121.057
115.280
46.832
44.598
38.255
35.320
33.270
27.570
24.727
22.468
21.622
15.799
cdcl3
125.71 MHz
OH
220 200 180 160 140 120 100 80 60 40 20 0

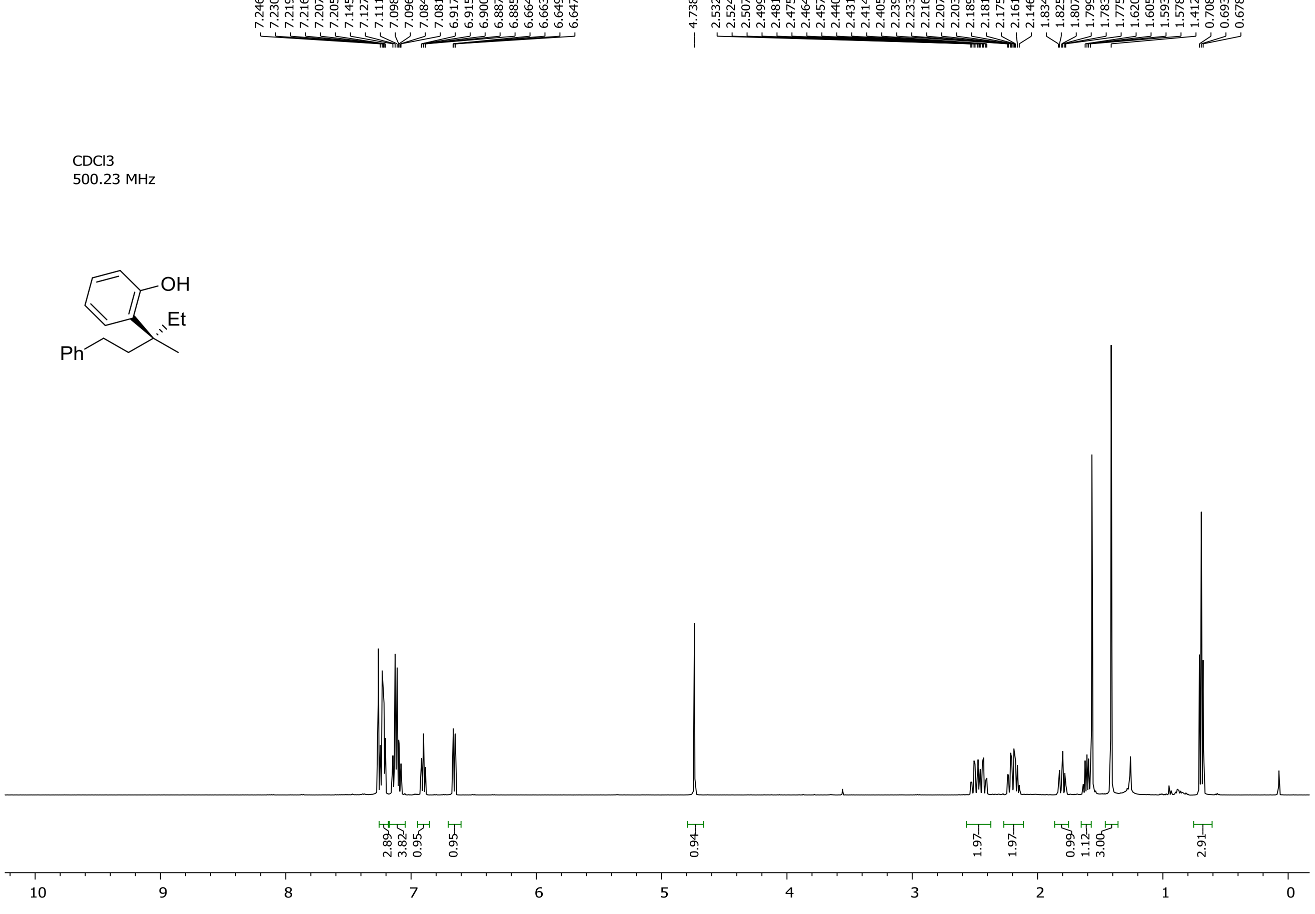
7.246 7.230 7.219 7.216 7.207 7.205 7.145 7.127 7.111 7.098 7.096 7.084 7.081 6.917 6.915 6.900 6.887 6.885 6.664 6.663 6.649 6.647
4.738
2.532 2.524 2.507 2.499 2.481 2.475 2.464 2.457 2.440 2.431 2.414 2.405 2.239 2.233 2.216 2.207 2.203 2.189 2.181 2.175 2.161 2.146 1.834 1.825 1.807 1.799 1.783 1.775 1.620 1.605 1.593 1.578 1.412 0.708 0.693 0.678
CDCl3
500.23 MHz
OH
Et
Ph
2.89 3.82 0.95 0.95 0.94 1.97 1.97 0.99 1.12 3.00 2.91
10 9 8 7 6 5 4 3 2 1 0

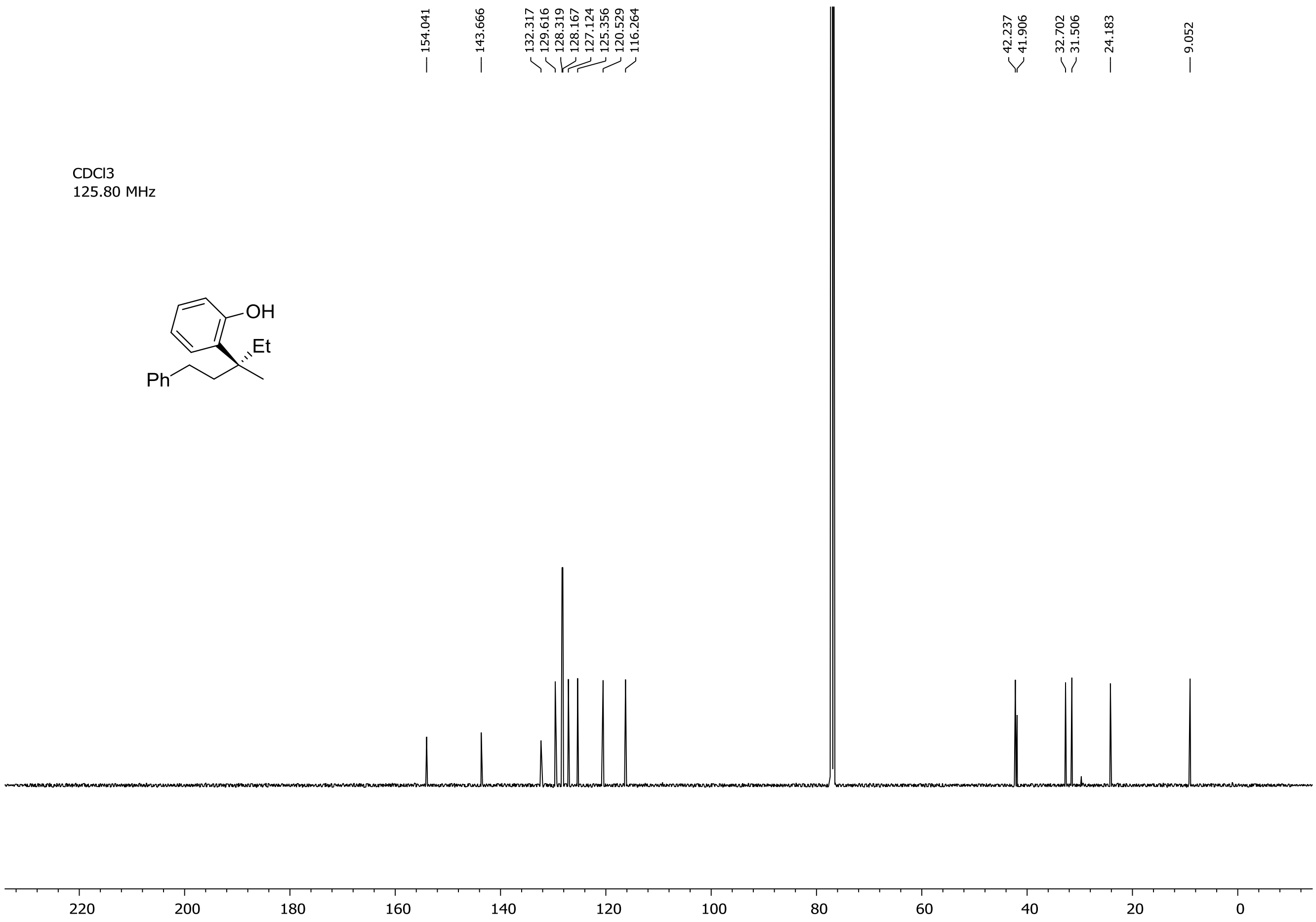
154.041
143.666
132.317
129.616
128.319
128.167
127.124
125.356
120.529
116.264
42.237
41.906
32.702
31.506
24.183
9.052
CDCl3
125.80 MHz
OH
Et
Ph
220
200
180
160
140
120
100
80
60
40
20
0

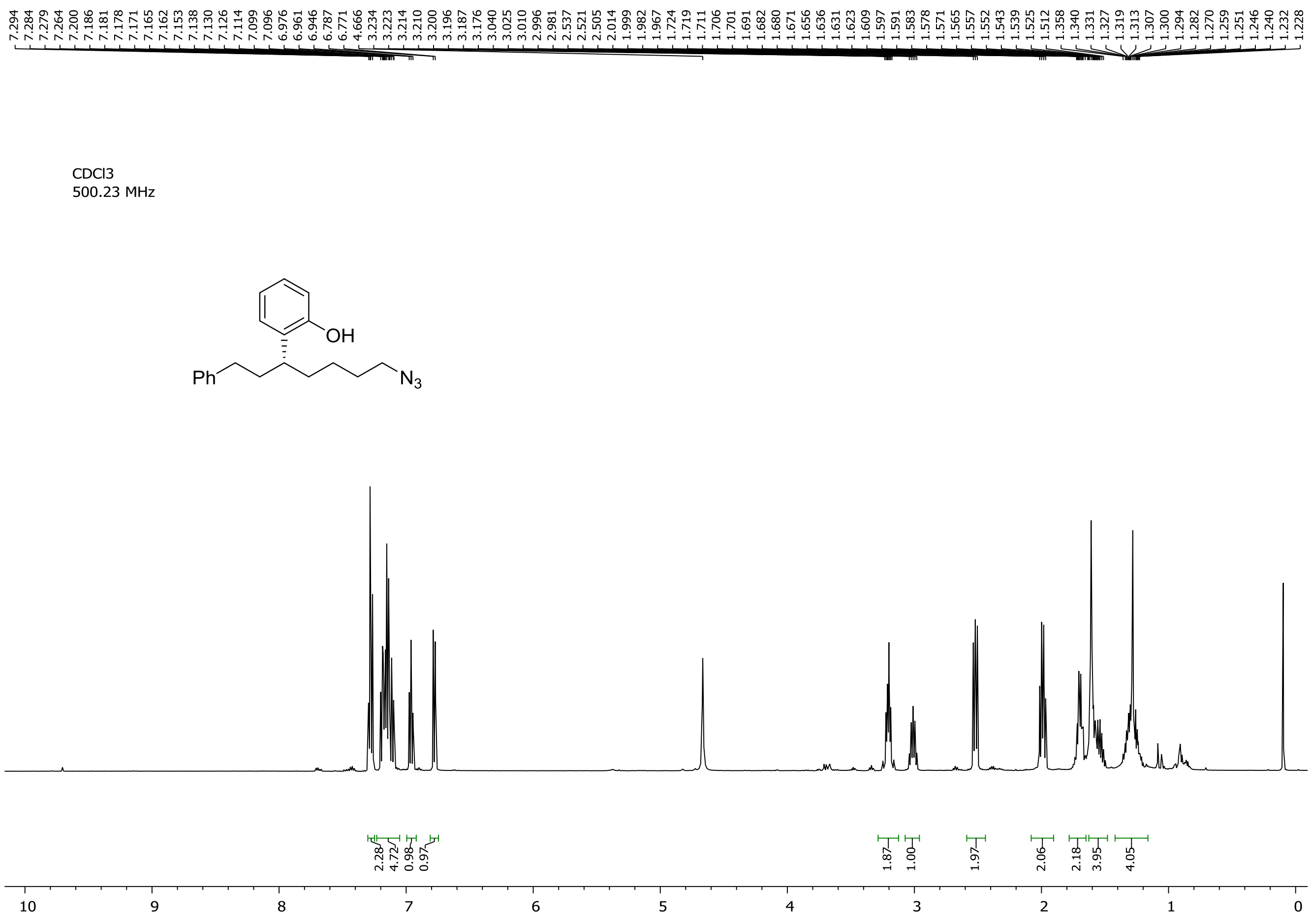
7.294 7.284 7.279 7.264 7.200 7.186 7.181 7.178 7.171 7.165 7.162 7.153 7.138 7.130 7.126 7.114 7.099 7.096 6.976 6.961 6.946 6.787 6.771 4.666 3.234 3.223 3.214 3.210 3.200 3.196 3.187 3.176 3.040 3.025 3.010 2.996 2.981 2.537 2.521 2.505 2.014 1.999 1.982 1.967 1.724 1.719 1.711 1.706 1.701 1.691 1.682 1.680 1.671 1.656 1.636 1.631 1.623 1.609 1.597 1.591 1.583 1.578 1.571 1.565 1.557 1.552 1.543 1.539 1.525 1.512 1.358 1.340 1.331 1.327 1.319 1.313 1.307 1.300 1.294 1.282 1.270 1.259 1.251 1.246 1.240 1.232 1.228
CDCl3
500.23 MHz
OH
Ph
N3
2.28
4.72
0.98
0.97
1.87
1.00
1.97
2.06
2.18
3.95
4.05
10
9
8
7
6
5
4
3
2
1
0

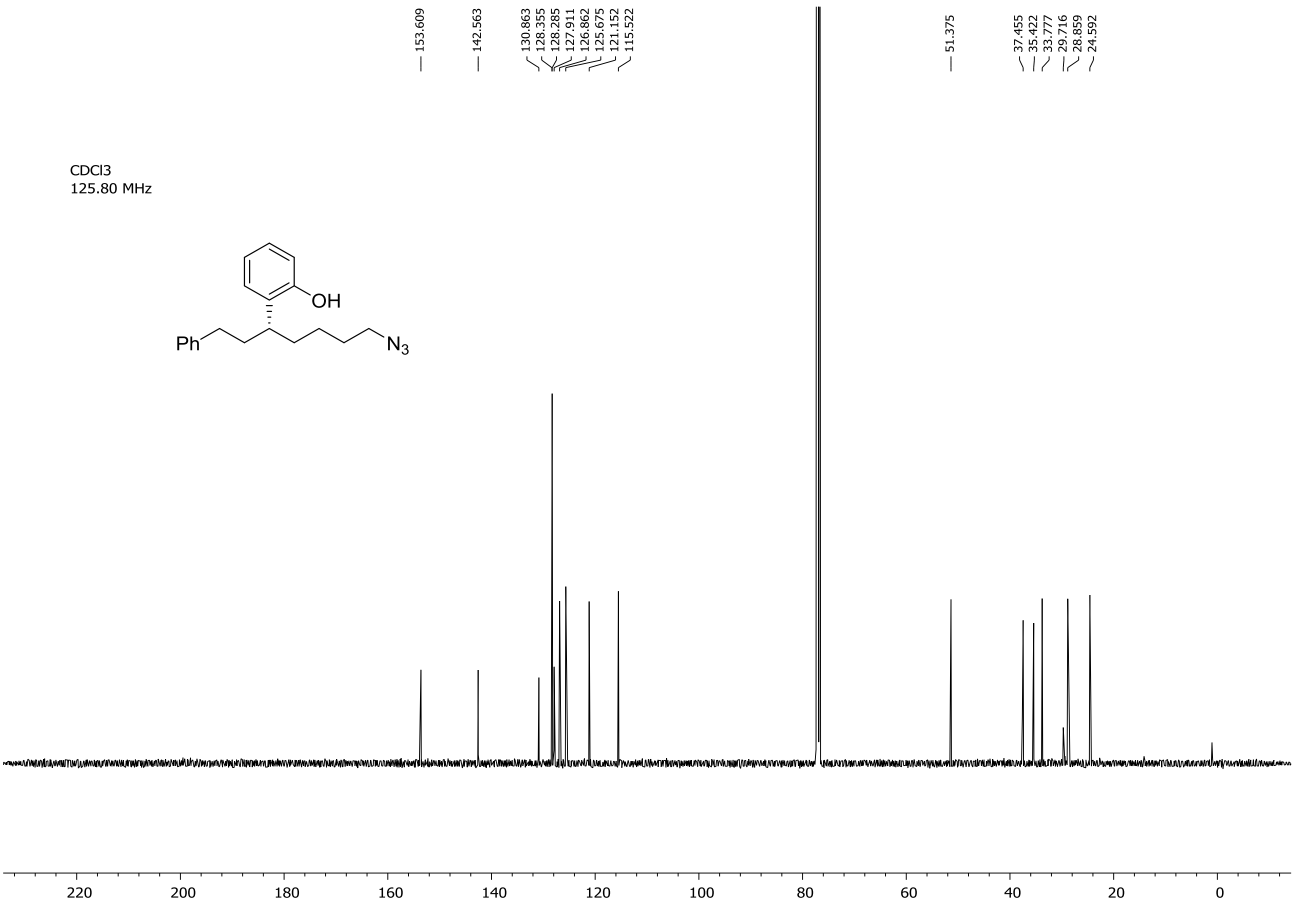
153.609
142.563
130.863
128.355
128.285
127.911
126.862
125.675
121.152
115.522
51.375
37.455
35.422
33.777
29.716
28.859
24.592
CDCl3
125.80 MHz
OH
Ph
N_3
220 200 180 160 140 120 100 80 60 40 20 0

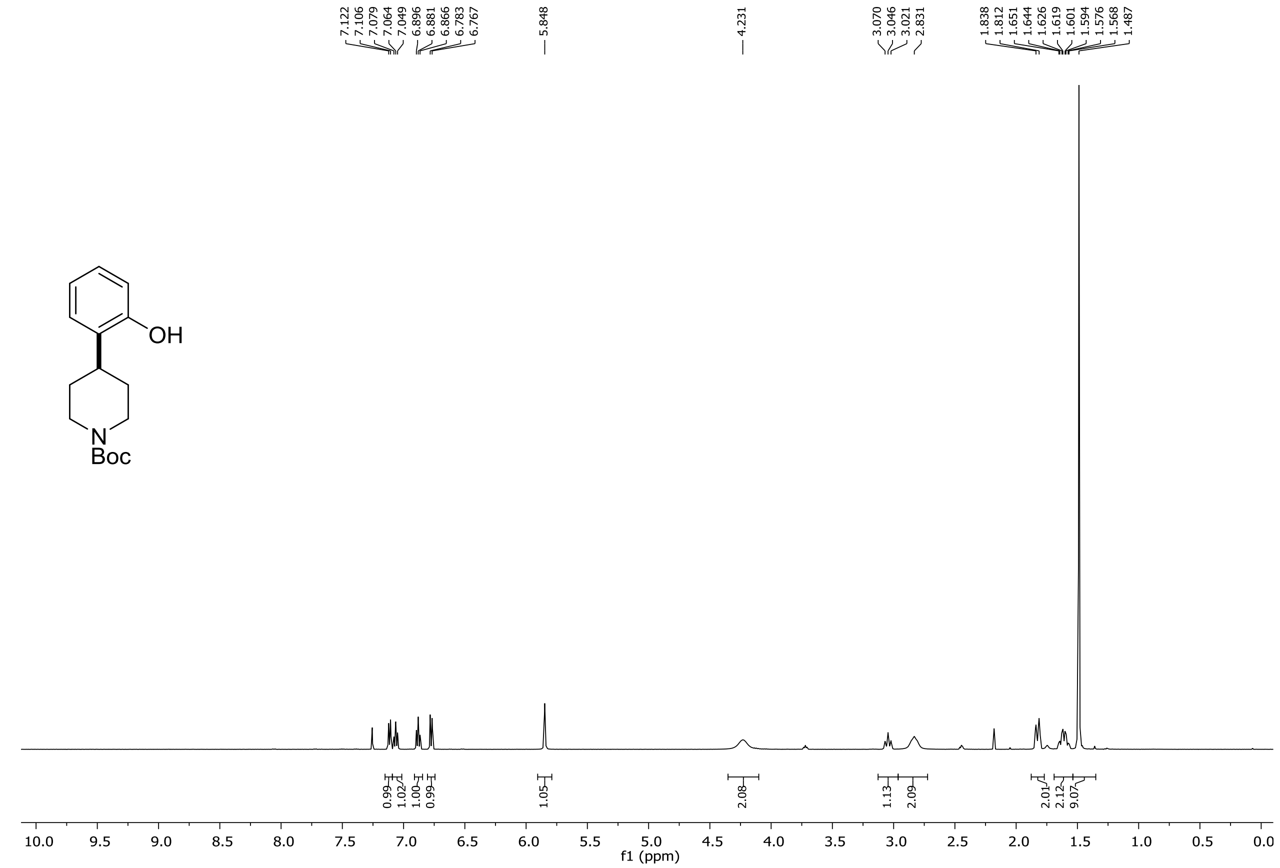
7.122
7.106
7.079
7.064
7.049
6.896
6.881
6.866
6.783
6.767
5.848
4.231
3.070
3.046
3.021
2.831
1.838
1.812
1.651
1.644
1.626
1.619
1.601
1.594
1.576
1.568
1.487
OH
N
Boc
0.99
1.02
1.00
0.99
1.05
2.08
1.13
2.09
2.01
2.12
9.07
10.0 9.5 9.0 8.5 8.0 7.5 7.0 6.5 6.0 5.5 5.0 4.5 4.0 3.5 3.0 2.5 2.0 1.5 1.0 0.5 0.0
f1 (ppm)

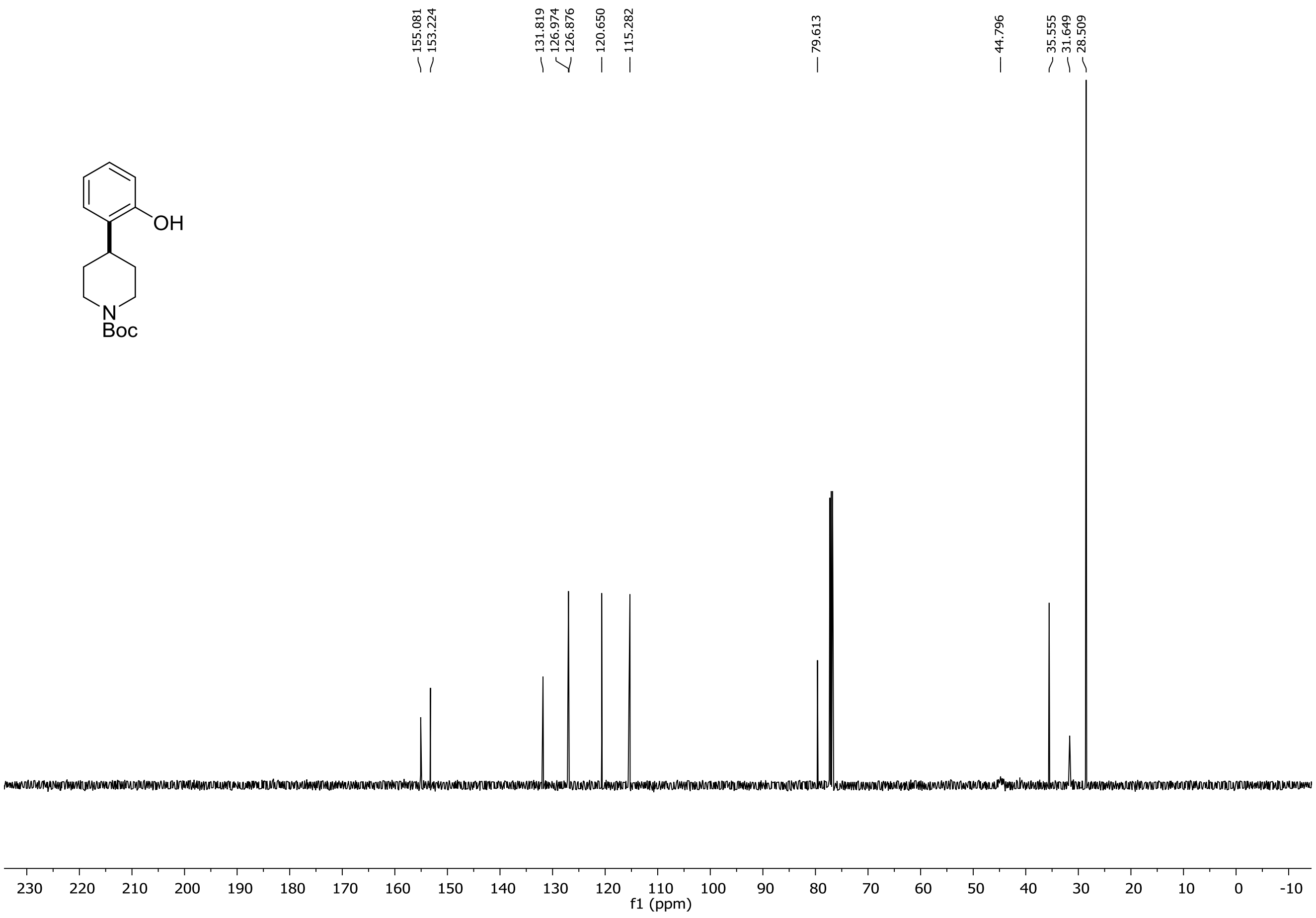

8. References

[1] (a) R. Larouche-Gauthier, T. G. Elford, V. K. Aggarwal, *J. Am. Chem. Soc.* **2011**, *133*, 16794-16797; (b) C. Sandford, R. Rasappan, V. K. Aggarwal, *J. Am. Chem. Soc.* **2015**, *137*, 10100-10103.

[2] D. Noh, H. Chea, J. Ju, J. Yun, *Angew. Chem. Int. Ed.* **2009**, *48*, 6062-6064.

[3] M. J. Hesse, C. P. Butts, C. L. Willis, V. K. Aggarwal, *Angew. Chem. Int. Ed.* **2012**, *51*, 12444-12448.

[4] X.-F. Zhou, Y.-D. Wu, J.-J. Dai, Y.-J. Li, Y. Huang, H.-J. Xu, *RSC Adv.* **2015**, *5*, 46672-46676.

[5] T. G. Driver, J. R. Harris, K. A. Woerpel, *J. Am. Chem. Soc.* **2007**, *129*, 3836-3837.

[6] H. Ito, K. Kubota, *Org. Lett.* **2012**, *14*, 890-893.

[7] (a) A. Bonet, M. Odachowski, D. Leonori, S. Essafi, V. K. Aggarwal, *Nature Chem.* **2014**, *6*, 584-589; (b) V. Ganesh, M. Odachowski, V. K. Aggarwal, *Angew. Chem. Int. Ed.* **2017**, *56*, 9752-9756.

[8] T. G. Elford, S. Nave, R. P. Sonawane, V. K. Aggarwal, *J. Am. Chem. Soc.* **2011**, *133*, 16798-16801.

[9] Z.-X. Wang, W.-M. Shi, H.-Y. Bi, X.-H. Li, G.-F. Su, D.-L. Mo, *J. Org. Chem.* **2016**, *81*, 8014-8021.

[10] S. Y. Lee, A. Villani-Gale, C. C. Eichman, *Org. Lett.* **2016**, *18*, 5034-5037.

[11] J. Wen, H. Qi, X. Kong, L. Chen, X. Yan, *Synthetic Communications* **2014**, *44*, 1893-1903.

[12] U. Azzena, G. Dettori, R. Pireddu, L. Pisano, *Tetrahedron* **2004**, *60*, 1617-1623.

[13] C.-S. Yan, Y. Peng, X.-B. Xu, Y.-W. Wang, *Chemistry – A European Journal* **2012**, *18*, 6039-6048.

[14] Y. Yamashita, J. C. Tellis, G. A. Molander, *Proceedings of the National Academy of Sciences* **2015**, *112*, 12026-12029.

[15] D.-H. Lee, K.-H. Kwon, C. S. Yi, *J. Am. Chem. Soc.* **2012**, *134*, 7325-7328.

[16] Y. Arredondo, M. Moreno-Mañas, R. Pleixats, *Synthetic Communications* **1996**, *26*, 3885-3895.
